# *Campylobacter concisus* Genomospecies 2 Is Better Adapted to the Human Gastrointestinal Tract as Compared with *Campylobacter concisus* Genomospecies 1

**DOI:** 10.3389/fphys.2017.00543

**Published:** 2017-08-03

**Authors:** Yiming Wang, Fang Liu, Xiang Zhang, Heung Kit Leslie Chung, Stephen M. Riordan, Michael C. Grimm, Shu Zhang, Rena Ma, Seul A. Lee, Li Zhang

**Affiliations:** ^1^School of Biotechnology and Biomolecular Sciences, University of New South Wales Sydney, NSW, Australia; ^2^Clinical Research Center, The First Affiliated Hospital of Nanjing Medical University Nanjing, China; ^3^Gastrointestinal and Liver Unit, Prince of Wales Hospital, University of New South Wales Sydney, NSW, Australia; ^4^St George and Sutherland Clinical School, University of New South Wales Sydney, NSW, Australia

**Keywords:** *Campylobacter concisus*, Crohn's disease, inflammatory bowel disease, 23S rRNA, genomospecies

## Abstract

*Campylobacter concisus* was previously shown to be associated with inflammatory bowel disease including Crohn's disease (CD) and ulcerative colitis (UC). *C. concisus* has two genomospecies (GS). This study systematically examined the colonization of GS1 and GS2 *C. concisus* in the human gastrointestinal tract. GS1 and GS2 specific polymorphisms in 23S rRNA gene were identified by comparison of the 23S rRNA genes of 49 *C. concisus* strains. Two newly designed PCR methods, based on the polymorphisms of 23S rRNA gene, were developed and validated. These PCR methods were used to detect and quantify GS1 and GS2 *C. concisus* in 56 oral and enteric samples collected from the gastrointestinal tract of patients with IBD and healthy controls. Meta-analysis of the composition of the isolated GS1 and GS2 *C. concisus* strains in previous studies was also conducted. The quantitative PCR methods revealed that there was more GS2 than GS1 *C. concisus* in samples collected from the upper and lower gastrointestinal tract of both patients with IBD and healthy controls, showing that GS2 *C. concisus* is better adapted to the human gastrointestinal tract. Analysis of GS1 and GS2 composition of isolated *C. concisus* strains in previous studies showed similar findings except that in healthy individuals a significantly lower GS2 than GS1 *C. concisus* strains were isolated from fecal samples, suggesting a potential difference in the *C. concisus* strains or the enteric environment between patients with gastrointestinal diseases and healthy controls. This study provides novel information regarding the adaptation of different genomospecies of *C. concisus* in the human gastrointestinal tract.

## Introduction

*Campylobacter concisus* is a Gram-negative motile bacterium, growing under anaerobic, and microaerophilic conditions in the presence of hydrogen gas (Lastovica et al., 2014; Lee et al., 2014). A number of studies reported an association between *C. concisus* and inflammatory bowel disease (IBD); these studies found that the prevalence of *C. concisus* in the intestinal tissues of patients with IBD was significantly higher than that in the controls (Zhang et al., 2009; Mahendran et al., 2011; Mukhopadhya et al., 2011; Kirk et al., 2016). IBD is a chronic inflammatory condition of the gastrointestinal tract, presenting as two major clinical forms including Crohn's disease (CD) or ulcerative colitis (UC; Sartor and Mazmanian, 2012). In addition to its association with IBD, some studies suggested that *C. concisus* may be involved in diarrheal disease (Lindblom et al., 1995; Kalischuk and Inglis, 2011; Nielsen et al., 2013).

*C. concisus* is part of the oral microbiota, with it being detected in saliva samples of nearly every individual (Zhang et al., 2010). *C. concisus* strains have different abilities in resistance to the enteric environmental factors such as the bile, some were able to colonize the intestinal tract (Ma et al., 2015). Some oral *C. concisus* strains may cause enteric diseases; they were found to induce intestinal epithelial death and production of proinflammatory cytokines (Nielsen et al., 2011; Ismail et al., 2012, 2013). Enteric virulent *C. concisus* strains translocated from the oral cavity to the intestinal tract were suggested to be initiators for a subgroup of IBD (Zhang et al., 2014; Zhang, 2015). *C. concisus* zonula occludens toxin was found to damage intestinal barrier function and enhance the responses of macrophage to other bacterial species (Mahendran et al., 2016), and phospholipase A was shown to damage human cell membrane (Istivan et al., 2004).

*C. concisus* has two genomospecies (GS), which can be consistently differentiated by the sequences of *C. concisus* core-genome, housekeeping genes and a polymerase chain reaction (PCR) targeting the polymorphisms of 23S rRNA gene (Aabenhus et al., 2005; Miller et al., 2012; On et al., 2013; Mahendran et al., 2015; Chung et al., 2016). The *C. concisus* GS1 and GS2 strains are morphologically similar but have genomospecies-specific genes, with some of the genes suggested to play a role in environmental adaptation (Chung et al., 2016). Previous studies suggested that *C. concisus* strains of different genomospecies may vary in their pathogenicity. For example, Engberg et al. examined *C. concisus* strains isolated from diarrheal stool samples and found that bloody diarrhea was present only in individuals infected with GS2 *C. concisus* (Engberg et al., 2005). A study from Kalischuk et al. found that GS2 *C. concisus* strains had a greater mean epithelial invasion and translocation than GS1 strains (Kalischuk and Inglis, 2011). Furthermore, oral *C. concisus* strains that were invasive to intestinal epithelial cells reported by Ismail et al. were GS2 strains (Ismail et al., 2012; Mahendran et al., 2015).

*C. concisus* was previously detected in clinical samples collected from both the upper and lower human gastrointestinal tract such as saliva, intestinal biopsies, and fecal samples (Zhang et al., 2009, 2010; Mahendran et al., 2011; Mukhopadhya et al., 2011; Kirk et al., 2016). The PCR methods used for the detection of *C. concisus* in these studies targeted the 16S rRNA gene, which were unable to differentiate *C. concisus* GS1 and GS2 strains. Therefore, it is not clear whether *C. concisus* previously detected in these clinical samples were GS1 or GS2.

This study aimed to examine the colonization of GS1 and GS2 *C. concisus* in the human gastrointestinal tract. We identified *C. concisus* GS1 and GS2 specific polymorphisms in the 23S rRNA gene. Based on these polymorphisms, we developed *C. concisus* GS1 and GS2 specific PCR methods and quantified GS1 and GS2 *C. concisus* in saliva, intestinal biopsies and fecal samples from patients with IBD and healthy controls. This study found that in the gastrointestinal tract of both patients with IBD and healthy controls, there were more GS2 than GS1 *C. concisus*, showing that GS2 *C. concisus* is better adapted to the environment of the human gastrointestinal tract. The potential clinical relevance was discussed.

## Methods

### Identification of *C. concisus* GS1 and GS2 specific polymorphisms in 23S rRNA gene

In order to design specific PCR methods that can be used for detection and quantification of *C. concisus* GS1 and GS2, we identified GS1 and GS2 specific polymorphisms in 23S rRNA gene. The 23S rRNA gene of 49 *C. concisus* strains was examined, including 13 *C. concisus* strains which had their genomes sequenced in this study and 36 publicly available *C. concisus* genomes with known GS1 and GS2 identities (Tanner et al., 1981; Deshpande et al., 2013; Chung et al., 2016). The 13 *C. concisus* genomes were sequenced using the MiSeq method and their GS1 and GS2 identities were determined using six housekeeping genes and 23rRNA genes as described previously (Chung et al., 2016). The sequences of the 23S rRNA gene and the housekeeping genes of these 13 *C. concisus* strains were submitted to National Center for Biotechnology Information (NCBI) Genbank. The accession numbers for the 23S rRNA gene and the six housekeeping genes were MF351708-MF351720 and MF358606-MF358683, respectively.

The sequences of the 23S rRNA gene of these 49 *C. concisus* strains were compared using MEGA7 (Kumar et al., 2016). From the alignments performed, GS1-specific and GS2-specific polymorphic nucleotides of the 23S rRNA gene were identified. GS1-specific polymorphic nucleotides refer to the nucleotides that are present in the 23S rRNA gene of all GS1 *C. concisus* strains but absent in all GS2 *C. concisus* strains, and *vice versa*.

### Development and validation of two PCR methods for detection and quantification of GS1 and GS2 *C. concisus*

PCR primers were designed by NCBI Primer-BLAST using the sequence of 23S rRNA gene of *C. concisus* strain 13826 (Ye et al., 2012). Four primers were selected. The sequences of these primers were compared to all species in the NCBI non-redundant database using Primer-BLAST, which showed 100% match to *C. concisus* and low similarities to other species. One of the primers had a sequence that was identical to the CON1 primer previously designed by Bastyns et al. (1995). Primers CON23S_GS1_F and CON1 were used to amplify GS1 *C. concisus* (GS1-PCR) with the product size being 234 bp, primers CON23S_GS2_F and CON23S_GS2_R were used to amplify GS2 *C. concisus* (GS2-PCR) with the product size being 90 bp. The sequences of these primers and the PCR thermocycling conditions are listed in Table 1.

**Table 1:** PCR Primers used to amplify the 23S rRNA gene of GS1 and GS2 *C. concisus* strains.

**Target gene**	**Primer name**	**Sequences (5′ → 3′)**	**Product size (bp)**
GS1 23S	CON23S_GS1_F	AGGGTTAGCCGGGTCCTAA	234
	CON1^*^	CAGTATCGGCAATTCGCT	
GS2 23S	CON23S_GS2_F	TGGTAGTGCTGGTCGAAAGG	90
	CON23S_GS2_R	CCGAAGAACTTCACGCACCT	
**CYCLING CONDITION**			
	Hold 95°C, 3 min 0 s		
	Cycling (40 repeats)	Step 1 at 95°C, hold 10 s	
		Step 2 at 61°C, hold 10 s	
		Step 3 at 72°C, hold 30 s	

* *CON1 primer was from reference (Bastyns et al., 1995), the remaining three primers were from this study. GS, genomospecies*.

The specificities of GS1-PCR and GS2-PCR were confirmed using 41 *C. concisus* strains with known GS1 and GS2 identities that were available to us, including the 27 *C. concisus* strains that we previously performed genome sequencing, the 13 genomes that were sequenced in this study and *C. concisus* strain 13826 (Chung et al., 2016). These *C. concisus* strains were cultured on horse blood agar plates and DNA was extracted as previously described (Mahendran et al., 2011; Ismail et al., 2012). An aliquot (5 ng) of the extracted bacterial DNA was subjected to GS1-PCR and GS2-PCR.

The use of the *C. concisus* GS1 and GS2 specific primers designed in this study in quantitative GS1-real time PCR (GS1-rtPCR) and GS2-real time PCR (GS2-rtPCR) was also validated. DNA samples extracted from GS1 strain P14UCO-S2 and GS2 strain 13826, which were chosen randomly, were used to construct the standard curves and the PCR efficiencies were determined by Rotor gene 6000 software (Dhanasekaran et al., 2010). The SYBR Green I method was used for rtPCR and the reagents were purchased from Bioline (NSW, Australia). Each reaction was prepared with 10 μl of 1 × SensiFAST™ SYBR® No-ROX mix, 0.8 μl of 400 nM forward primer, 0.8 μl of 400 nM reverse primer, mixed with 2 μl of serial diluted bacterial DNA template of concentrations ranged between 0.0015 and 15 ng/μl, the volume was topped up to 20 μl with nuclease-free water. Amplifications were performed in a three step rtPCR using the following cycling conditions: 95°C for 3 min, followed by 40 cycles of 95°C for 10 s, 61°C for 10 s, and 72°C for 30 s (Fite et al., 2004). No primer dimers were observed in the melting curve.

The PCR methods developed in this study were then used to detect and quantify GS1 and GS2 *C. concisus* in oral and enteric samples collected from patients with IBD and healthy controls.

### Detection and quantification of GS1 and GS2 *C. concisus* in DNA samples extracted from saliva, intestinal biopsies, and fecal samples

A total of 56 *C. concisus* positive samples collected from the human gastrointestinal tract were used for GS1 and GS2 *C. concisus* quantification, including 27 saliva samples and 29 enteric samples (**Tables 3, 4**). These samples were collected in a previous study (Mahendran et al., 2011). In that study, multiple intestinal biopsies (each at ileum, caecum, colon, and rectum), were collected from each individual and used for *C. concisus* detection by the genus PCR. The *C. concisus* positive intestinal biopsies were used in this study for the quantification of GS1 and GS2 *C. concisus*. Fecal fluids refer to the liquids containing fecal materials in the draining tubes during the colonoscopy procedure. DNA samples of intestinal biopsies were extracted previously (Mahendran et al., 2011). DNA samples of the saliva and fecal fluids were extracted in this study using the ISOLATE fecal DNA kit (Bioline) following the manufacturer's instructions. Azathioprine, mercaptopurine, and 5-aminosalicylic acid were shown to affect *C. concisus* growth, however the samples used in this study were collected from the patients who have not received any treatment (Liu et al., 2017).

GS1-PCR and GS2-PCR methods were used to detect the presence of GS1 and GS2 *C. concisus* in DNA samples extracted from saliva and fecal fluids. PCR reactions had 25 μl in volume, containing 2.5 μl of 10 × DNA polymerase buffer, 2.5 μl of 10 × dNTP, 1.5 μl of 25 mM MgCl_2_, 1 μl of 10 pmol forward primer, 1 μl of 10 pmol reverse primer, 12.5 μl of nuclease-free water, 2 μl of DNA template and 2 μl of *Taq* polymerase. DNA templates used were 5 ng DNA extracted from saliva, 100 ng DNA extracted from intestinal biopsies and 50 ng DNA extracted from fecal fluids. A PCR reaction without bacterial DNA was used as the negative control. Positive PCR products were sequenced as previously described using BigDye^TM^ terminator chemistry (Applied Biosystems, Foster City, USA) (Mahendran et al., 2011). The sequences of the PCR products were compared to the 23S rRNA sequences of the corresponding *C. concisus* strains (Chung et al., 2016).

DNA samples extracted from saliva, fecal samples and intestinal biopsies were then subjected to GS1-rtPCR and GS2-rtPCR for quantification. The results were presented as copy numbers, amplified using 5 ng DNA extracted from saliva, 100 ng DNA extracted from intestinal biopsies and 50 ng DNA extracted from fecal fluids. The copy numbers of amplified *C. concisus* GS1 and GS2 in clinical samples were determined using standard curves that were generated using DNA extracted from GS1 strain P14UCO-S2 and GS2 strain 13826, respectively. Non-template control group was included in each run. For each run, the melting curves of individual samples were checked.

### Meta-analysis of the prevalence of GS1 and GS2 *C. concisus* in isolated oral and enteric *C. concisus* strains in publicly available databases

A literature search was performed in January 2017 and relevant publications were initially identified using the searching terms “*Campylobacter concisus*” in PubMed and Web of Science. The identified publications were then refined using searching terms “*Campylobacter concisus* isolation,” “*Campylobacter concisus* genomospecies,” and “*Campylobacter concisus* genotypic.” The identified publications following the refined search were manually inspected and studies containing defined *C. concisus* genomospecies were included in the analysis. In these studies, amplified fragment length polymorphism (AFLP), PCR targeting 23S rRNA gene, sequences of housekeeping genes and sequences of *C. concisus* core genomes were used for *C. concisus* genomospecies identification (Aabenhus et al., 2005; Engberg et al., 2005; Kalischuk and Inglis, 2011; On et al., 2013; Mahendran et al., 2015; Chung et al., 2016; Nielsen et al., 2016). These methods were consistent in defining GS1 and GS2 *C. concisus* (Aabenhus et al., 2005; Engberg et al., 2005; Kalischuk and Inglis, 2011; On et al., 2013; Mahendran et al., 2015; Chung et al., 2016; Nielsen et al., 2016). Given this, GS1 and GS2 *C. concisus* strains defined by these methods were combined and used for analysis of the prevalence of GS1 and GS2 *C. concisus* strains.

### Statistical analysis

Fisher's exact test (two tailed) was used to compare the prevalence of GS1 and GS2 *C. concisus*. Unpaired *t*-test was used to compare the copy numbers of GS1 and GS2 *C. concisus* DNA. *P* < 0.05 was considered statistically significant.

## Results

### *C. concisus* GS1 and GS2 specific polymorphisms in 23S rRNA gene

In this study, the genomes of 13 *C. concisus* strains were sequenced, of which 11 strains were GS2 and two strains were GS1 based on the phylogenetic trees generated using the 23S rRNA genes and housekeeping genes (Supplementary Figure S1). Comparison of the sequences of the 23S rRNA gene of 49 *C. concisus* strains including those sequenced in this study and publicly available genomes (10 GS1 strains and 39 GS2 strains) revealed 31 GS-specific polymorphisms, at positions 257–258, 269–270, 330, 347, 1,453–1,456, 1,460, 1,477, 1,489, 1,495–1,497, 1,500, 1,513, 1,516–1,518, 1,530, 1,534, 1,540, 1,542–1,543, 1,564, 1,566–1,567, and 1,572–1,573 bp (Table 2, Supplementary Figure S2). Most of the genomospecies-specific polymorphic nucleotides (81%, 25/31) were in the region between 1,453 and 1,575 bp. In addition to the 25 GS-specific nucleotides, this region also contained 22 polymorphic nucleotides that were either strain-specific or occurred in a number of strains. In total, this region contained 47 (38%, 47/123) polymorphic nucleotides (Table 2, Supplementary Figure S2).

**Table 2:** *C. concisus* genomospecies specific nucleotide polymorphisms of 23S rRNA gene.

**Nucleotide position**	**GS1**	**GS2**
257–258	GT	AC
269–270	AC	GT
330	A	G
347	T	C
1,453–1,456	AGCA	GAGG
1,460	T	C
1,477	A	G
1,489	T	C
1,495–1,497	AAG	GGA
1,500	C	T
1,513	G	A
1,516–1,518	CTT	TCC
1,530	A	G
1,534	C	T
1,540	C	T
1,542–1,543	TT	CG
1,564	A	C
1,566–1,567	CG	TA
1,572–1,573	GC	CT

*Genomospecies specific nucleotide polymorphisms of 49 C. concisus strains (10 GS1 strains and 39 GS2 strains) were analyzed using MEGA7. Genomospecies specific nucleotide polymorphisms refer to the polymorphisms that were present in all GS1 strains but absent in all GS2 strains and vice versa. GS, genomospecies. C. concisus strain ATCC 33237 was used as reference strain for nucleotide position*.

### The specificities of GS1-PCR and GS2-PCR and the amplification efficiencies of GS1-rtPCR and GS2-rtPCR

The specificities of GS1-PCR and GS2-PCR were confirmed using DNA extracted from 41 *C. concisus* strains with known GS1 and GS2 identity. GS1-PCR was positive for all 8 GS1 strains and negative for all 33 GS2 strains. GS2-PCR was positive for all 33 GS2 strains and negative for the 8 GS1 strains.

The positive PCR products revealed a single band on agarose gel with the expected sizes (Supplementary Figure S3A). Sequencing the positive PCR products confirmed the identity of *C. concisus* 23S rRNA gene.

The amplification efficiencies of GS1-rtPCR and GS2-rtPCR were 93.4 and 95.4%, respectively. No primer dimer peaks were observed.

### Detection and quantification of GS1 and GS2 *C. concisus* in saliva, fecal samples, and intestinal biopsies from patients with IBD and healthy controls that were positive for both GS1 and GS2 *C. concisus*

The DNA samples extracted from 27 saliva samples, nine fecal samples and 20 intestinal biopsies were all positive for both GS1-PCR and GS2-PCR. These DNA samples were then subjected to GS1-rtPCR and GS2-rtPCR respectively for quantitative measurement of GS1 and GS2 *C. concisus*.

The majority of the saliva DNA samples (25/27, 92.6%) contained significantly higher copy numbers of GS2 than GS1 *C. concisus*, which were seen in both patients with IBD (92.3%, 12/13) and healthy controls (92.9%, 13/14; Table 3). The mean GS2/GS1 ratios in saliva samples from patients with IBD were 16 ± 24 and 22 ± 28, respectively, which was not significantly different (*P* > 0.05).

**Table 3:** Quantification of GS1 and GS2 *C. concisus* in saliva samples from patients with IBD and healthy controls.

**Samples' No**.	**Status**	**Average copy number for GS1**	**Average copy number for GS2**	**Ratio (GS2/GS1)**	***t*-test (*P*-value)**
1	CD	1.78E+06	8.09E+07	45.47	0.0000025
2	CD	4.53E+06	1.73E+07	3.83	0.0002848
3	CD	1.05E+07	8.35E+07	7.92	0.0000034
4	CD	8.61E+04	7.15E+06	83.06	0.0087320
5	CD	7.73E+05	3.11E+06	4.03	0.0001298
6	CD	3.71E+05	1.16E+07	31.25	0.0001030
7	CD	1.19E+07	4.89E+07	4.11	0.0022292
8	CD	1.31E+05	8.21E+05	6.28	0.0154187
9	CD	1.37E+06	3.91E+05	0.28	0.0009157
10	CD	8.04E+06	2.15E+07	2.67	0.0044400
11	UC	2.13E+05	1.68E+06	7.93	0.0000113
12	UC	3.75E+06	8.26E+06	2.20	0.0002831
13	UC	4.34E+05	5.27E+06	12.15	0.0016518
14	HC	1.98E+05	1.24E+07	62.81	0.0000027
15	HC	1.03E+06	8.63E+05	0.83	0.0899524
16	HC	1.45E+05	2.10E+06	14.43	0.0000639
17	HC	4.06E+05	2.51E+06	6.17	0.0006932
18	HC	5.53E+04	6.09E+05	11.02	0.0037420
19	HC	6.85E+05	4.31E+06	6.29	0.0000032
20	HC	1.10E+05	2.50E+06	22.65	0.0003467
21	HC	7.34E+05	1.83E+07	24.91	0.0000337
22	HC	1.59E+05	4.79E+06	30.20	0.0000597
23	HC	2.88E+04	2.90E+06	100.84	0.0007940
24	HC	9.80E+04	6.26E+05	6.38	0.0006584
25	HC	1.47E+05	5.92E+05	4.03	0.0009846
26	HC	1.84E+06	5.14E+06	2.79	0.0003896
27	HC	1.04E+06	1.35E+07	12.89	0.0000683

*Ratio: the average GS2 copy numbers divided by the average GS1 copy numbers of the same sample. All saliva samples carried more copy numbers of GS2 than GS1 C. concisus, except for samples 9 and 15. CD, Crohn's Disease; UC, ulcerative colitis; HC, healthy control*.

All 29 enteric DNA samples (20 intestinal biopsies and nine fecal samples) contained significantly higher copy numbers of GS2 than GS1 *C. concisus* (Table 4). The average GS2/GS1 ratios of both intestinal biopsies and fecal samples between patients with IBD and healthy controls were not significantly different (129 ± 71 vs. 131 ± 119 and 62 ± 44 vs. 52 ± 22, respectively, *P* > 0.05).

**Table 4:** Quantification of GS1 and GS2 *C. concisus* in enteric samples from patients with IBD and healthy controls.

**Samples' No**.	**Site**	**Status**	**Copy number for GS1**	**Copy number for GS2**	**Ratio (GS2/GS1)**	***t*-test (*P*-value)**
1	Colon	CD	1.16E+04	2.76E+06	237.30	0.00001098
2	Rectum	CD	1.59E+04	2.34E+06	146.91	0.00001123
3	Ileum	CD	1.45E+04	2.81E+06	192.81	0.00000572
4	Colon	CD	7.36E+04	1.35E+07	183.07	0.00664986
5	Caecum	CD	6.56E+04	3.71E+06	56.60	0.00000019
6	Ileum	CD	7.09E+04	2.04E+06	28.80	0.00005750
7	Caecum	UC	1.31E+04	1.91E+06	146.35	0.00000690
8	Colon	UC	6.78E+04	1.90E+06	27.98	0.00000374
9	Ileum	UC	1.01E+04	1.27E+06	125.37	0.00000024
10	Rectum	UC	1.43E+04	2.01E+06	140.51	0.00000099
11	Ileum	HC	3.56E+04	2.09E+06	58.84	0.00000055
12	Rectum	HC	1.47E+04	1.40E+06	94.95	0.00000187
13	Ileum	HC	1.53E+04	2.31E+06	150.60	0.00000157
14	Rectum	HC	4.46E+04	2.89E+06	64.90	0.00003156
15	Caecum	HC	9.58E+04	1.01E+07	105.54	0.00017316
16	Ileum	HC	3.30E+04	7.48E+06	226.95	0.02185015
17	Caecum	HC	3.09E+04	2.68E+06	86.53	0.00411064
18	Rectum	HC	8.32E+03	3.56E+06	427.45	0.00124217
19	Colon	HC	4.82E+04	4.07E+06	84.44	0.00010349
20	Rectum	HC	1.19E+05	1.44E+06	12.12	0.00088559
21	Feces	CD	7.49E+05	1.79E+07	23.96	0.00000172
22	Feces	CD	4.03E+05	3.45E+07	85.71	0.00000337
23	Feces	CD	9.51E+04	5.14E+06	54.01	0.00000332
24	Feces	CD	3.51E+05	7.52E+06	21.40	0.00090633
25	Feces	CD	1.46E+05	1.83E+07	124.84	0.00038192
26	Feces	HC	1.82E+05	5.64E+06	30.96	0.00012801
27	Feces	HC	1.26E+05	4.37E+06	34.59	0.00001531
28	Feces	HC	1.12E+05	7.98E+06	71.01	0.00006877
29	Feces	HC	6.98E+05	4.90E+07	70.27	0.00000350

*Ratio: the average GS2 copy numbers divided by the average GS1 copy numbers of the same sample. All enteric samples carried more copy numbers of GS2 than GS1 C. concisus. CD, Crohn's Disease; UC, ulcerative colitis; HC, healthy control*.

**Figure 1:**
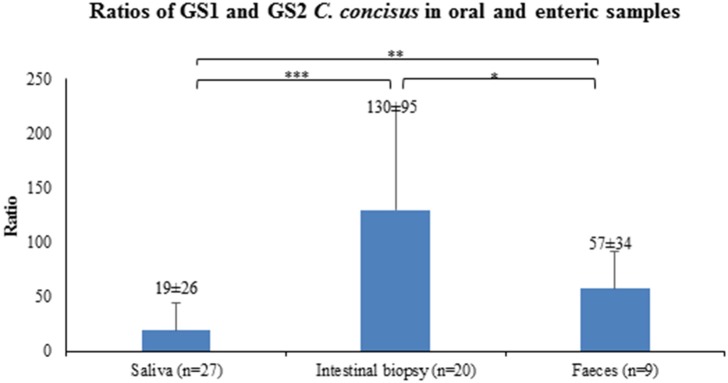
GS2/GS1 *C. concisus* ratios in oral and enteric samples. The copy numbers of GS1 and GS2 *C. concisus* determined by quantitative PCR methods were used to calculate the GS2/GS1 ratios in the oral and enteric samples. The average GS2/GS1 ratio in intestinal biopsy samples was 130 ± 95, which was significantly higher than that of the saliva samples (19 ± 26) and fecal samples (57 ± 34). The GS2/GS1 ratio in fecal samples was 57 ± 34, which was significantly higher than that of the saliva samples (19 ± 26). ^*^Indicates statistical significance (^*^P < 0.05, ^**^P < 0.01, ^***^P < 0.001).

Given that the average GS2/GS1 ratios in saliva, intestinal biopsies and fecal samples between patients with IBD and healthy controls were not significantly different, samples from patients with IBD and controls were combined to compare the GS2/GS1 ratios between samples collected from the upper and lower human gastrointestinal tract. The average GS2/GS1 ratio in the 20 intestinal biopsies was 130 ± 95, which was significantly higher than that of the 27 saliva samples (19 ± 26, *P* < 0.001) and that of the nine fecal samples (57 ± 34, *P* < 0.05). The average GS2/GS1 ratio in the fecal samples was significantly higher than that of the saliva samples (*P* < 0.01; Figure 1).

### Meta-analysis of the prevalence of GS1 and GS2 *C. concisus* in isolated oral and enteric *C. concisus* strains

Initial literature search using term “*Campylobacter concisus*” revealed a total of 149 publications in PubMed and 189 publications in Web of Science. Searching terms “*Campylobacter concisus* isolation” *Campylobacter concisus* genomospecies” and “*Campylobacter concisus* genotypic” were used to further refine the results. According to the criteria described in Methods Section, a total of 260 *C. concisus* strains obtained from seven original research publications were included in the analysis of the prevalence of GS1 and GS2 *C. concisus*, of which 194 strains were isolated from fecal samples, nine strains from intestinal biopsies and 57 strains from the oral cavity (Table 5).

**Table 5:** Prevalence of GS1 and GS2 *C. concisus* strains in isolated oral and enteric *C. concisus* strains from previous studies.

**Clinical condition**	**Origin of isolation**	**Prevalence of strains %**	**Method**	**References**
Gastroenteritis	Oral	GS1 25% (1/4)	APLF	Aabenhus et al., 2005
		GS2 75% (3/4)		
	Fecal	GS1 42% (21/50)		
		GS2 58% (29/50)		
Gastroenteritis	Fecal	GS1 11.8% (2/14)	PCR of 23S rRNA	Kalischuk and Inglis, 2011
		GS2 70.6% (12/14)		
Healthy	Fecal	GS1 80% (4/4)		
		GS2 0% (0/4)		
Gastroenteritis	Fecal	GS1 31% (12/39)	PCR of 23S rRNA	Engberg et al., 2005
		GS2 69% (27/39)		
Healthy	Fecal	GS1 67% (2/3)		
		GS2 33% (1/3)		
Gastroenteritis	Fecal	GS1 33% (9/27)	AFLP	On et al., 2013
		GS2 67% (18/27)		
CD	Oral	GS1 0% (0/1)	PCR of 23S rRNA	Nielsen et al., 2016
		GS2 100% (1/1)		
	Fecal	GS1 37.5% (3/8)		
		GS2 62.5% (5/8)		
Gastroenteritis	Oral	GS1 0% (0/2)		
		GS2 100% (2/2)		
	Fecal	41% (20/49)		
		59% (29/49)		
CD	Intestinal biopsy	GS1 0% (0/3)	Core genome	Chung et al., 2016
		GS2 100% (3/3)		
UC	Intestinal biopsy	GS1 100% (1/1)		
		GS2 0% (0/1)		
Gastroenteritis	Intestinal biopsy	GS1 75% (3/4)		
		GS2 25% (1/4)		
Healthy	Intestinal biopsy	GS1 0% (0/1)		
		GS2 100% (1/1)		
CD	Oral	GS1 44% (8/18)	Housekeeping genes	Mahendran et al., 2015
		GS2 56% (10/18)		
UC	Oral	GS1 20% (3/15)		
		GS2 80% (12/15)		
Healthy	Oral	GS1 41% (7/17)		
		GS2 59% (10/17)		

*The prevalence of GS1 and GS2 C. concisus strains in patients with CD, UC, gastroenteritis and healthy individuals were obtained from previous studies. APLF, amplified fragment length polymorphisms; CD, Crohn's disease; UC, ulcerative colitis*.

**Figure 2:**
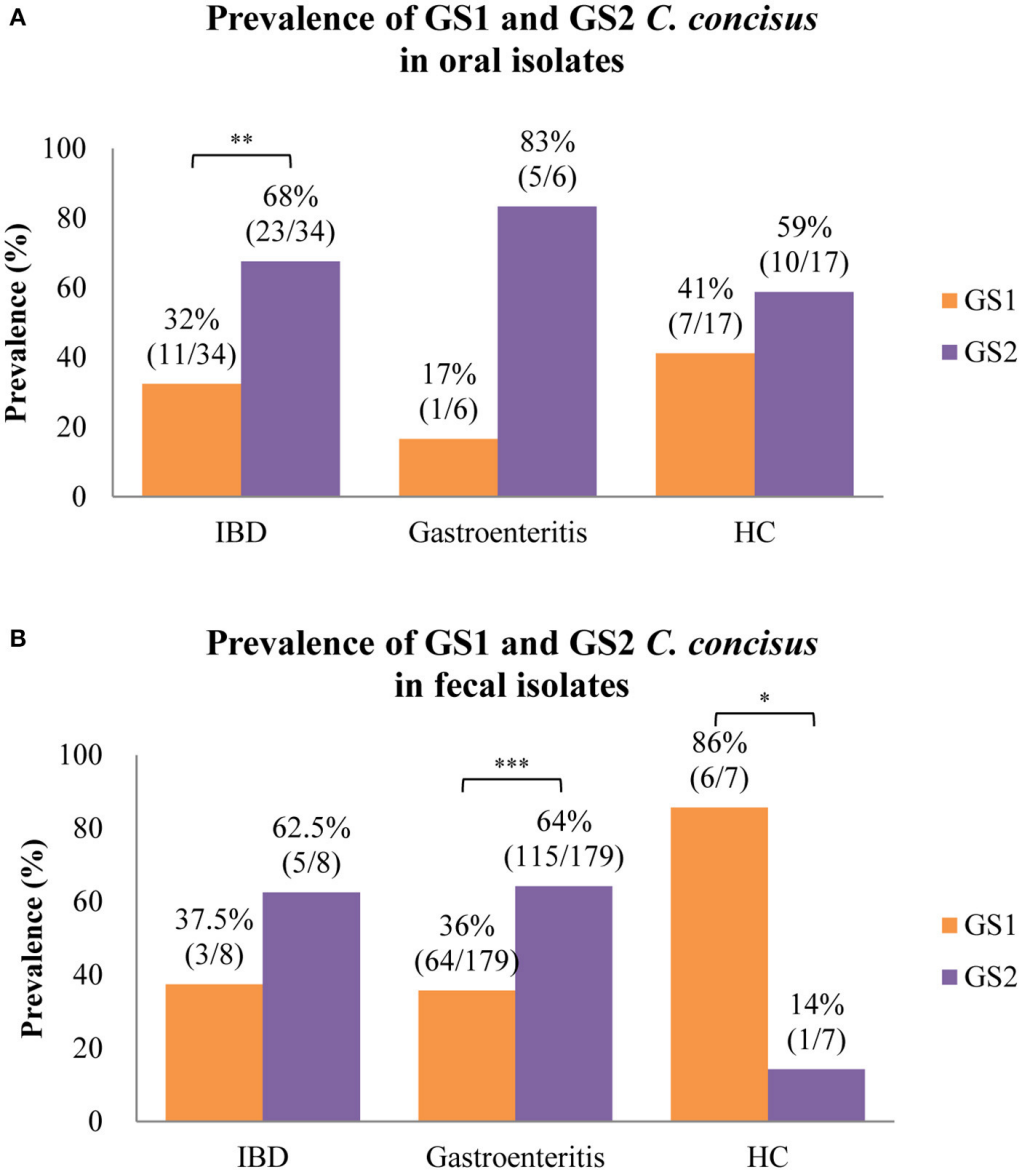
Meta-analysis of the prevalence of GS1 and GS2 *C. concisus* strains isolated from oral and fecal samples in patients with IBD, gastroenteritis, and healthy controls. **(A)** GS2 *C. concisus* has a higher prevalence in the oral cavity of both patients with enteric diseases and healthy controls as compared to GS1 *C. concisus*. **(B)** Most of the strains isolated from fecal samples of patients with enteric diseases were GS2 *C. concisus*, while most of those from healthy controls were GS1 *C. concisus*. IBD, inflammatory bowel disease. ^*^Indicates statistical significance (^*^P < 0.05, ^**^P < 0.01, ^***^P < 0.001). These *C. concisus* strains were reported in previous studies.

Of the *C. concisus* strains isolated from saliva samples, there were more GS2 than GS1 strains and the prevalence of GS2 strains was significantly higher than GS1 strains is strains isolated from patients with IBD (68 vs. 32%, *P* < 0.01; Figure 2A). The prevalence of GS2 and GS1 strains in oral strains isolated from patients with IBD, healthy controls, and gastroenteritis was not significantly different.

The *C. concisus* strains isolated from fecal samples from patients with IBD and gastroenteritis also had more GS2 than GS1 *C. concisus* strains and the prevalence of GS2 in strains isolated from patients with gastroenteritis was significantly higher than the GS1 strains (64 vs. 36%, *P* < 0.001; Figure 2B). There was less GS2 than GS1 in *C. concisus* strains isolated from fecal samples from healthy individuals, and the difference was statistically significant (14 vs. 86%, *P* < 0.05; Figure 2B).

Up until now, only small numbers *C. concisus* strains isolated from intestinal biopsies had known GS1 and GS2 identities, including one strain from a patient with UC, three strains from patients with CD, four strains from patients with gastroenteritis and one strain from a healthy individual (Table 4). Given the small strain numbers, we did not analyse the prevalence of GS1 and GS2 in *C. concisus* strains isolated from intestinal biopsies.

## Discussion

In this study, we systematically examined the colonization of GS1 and GS2 *C. concisus* in the human gastrointestinal tract using both quantitative PCR methods and a meta-analysis of isolated *C. concisus* strains in publicly available publications.

This study firstly identified *C. concisus* GS1 and GS2 specific polymorphic nucleotides in the 23S rRNA gene. In a recent study, we showed that the sequences of *C. concisus* core-genome, six housekeeping genes and 23S rRNA gene consistently divided 36 *C. concisus* strains into two genomospecies (Chung et al., 2016). The finding that the sequences of 23S rRNA gene were able to differentiate GS1 and GS2 *C. concisus* strains suggests that the *C. concisus* 23S rRNA gene contains *C. concisus* genomospecies specific polymorphisms. Indeed, comparison of the sequences of 23S rRNA gene of 49 GS1 and GS2 *C. concisus* strains conducted in this study identified multiple GS1 and GS2 specific polymorphic nucleotides in the 23S rRNA gene, most of which were in the highly polymorphic region 1,453–1,575 bp (Table 2, Supplementary Figure S2). The GS1 and GS2 multiple specific polymorphic nucleotides provide the molecular base of using this gene to assign GS1 and GS2 *C. concisus* strains (Chung et al., 2016).

We then designed and validated specific PCR methods for GS1 and GS2 *C. concisus* detection and quantification. CON1, CON2, and MUC1 are PCR primers designed by Bastyns et al. aiming to detect *C. concisus* by amplification of 23S rRNA gene (Bastyns et al., 1995). A number of studies used these PCR primers to define *C. concisus* genomospecies (Bastyns et al., 1995; Engberg et al., 2005; Kalischuk and Inglis, 2011; Nielsen et al., 2016). In our preliminary experiments, we attempted to use CON1, CON2, and MUC1 primers in rtPCR using SYBR Green I method to quantify GS1 and GS2 *C. concisus*. However, primer dimers were frequently observed when using CON1, CON2, and MUC1 for examination of GS1 and GS2 *C. concisus* in enteric samples, interfering with the interpretation of the results. We therefore designed and validated two new PCR methods for detection of GS1 and GS2 *C. concisus*, respectively. The newly designed PCR methods are specific in differentiating GS1 and GS2 strains, as demonstrated using *C. concisus* strains with known GS1 and GS2 identity. Furthermore, these PCR primers were suitable for rtPCR to quantify GS1 and GS2 *C. concisus* using SYBR Green I method; no primer dimers were seen and the amplification efficiencies were 93.4 and 95.4%, respectively (Supplementary Figure S3B). Using the newly developed PCR methods, we quantified GS1 and GS2 *C. concisus* in the upper and lower human gastrointestinal tract using oral and enteric samples previously collected from patients with IBD and controls.

Using quantitative PCR methods, we found that GS2 *C. concisus* is better adapted the human gastrointestinal tract than GS1 *C. concisus*. The copy numbers of GS2 *C. concisus* were significantly higher than GS1 *C. concisus* in most of the saliva samples and all enteric samples (Tables 3, 4). We previously found that GS2 and GS1 *C. concisus* strains have genomospecies-specific genes; one such gene specific to GS2 strains is aquaporin-Z (Chung et al., 2016). Aquaporin-Z is a bacterial membrane-bound water channel protein, which has been shown to mediate entry or exit of water in response to large changes in extracellular osmolarity (Delamarche et al., 1999). The osmolarity in the human gastrointestinal tract varies greatly and rapidly due to the uptake of large variety of liquids or solid food. The aquaporin-Z water channel may have contributed to the better adaption of GS2 *C. concisus* in the human gastrointestinal tract.

It was found that there were significantly higher GS2/GS1 ratios in intestinal biopsies than the oral cavity and intestinal lumen (Figure 1). This may be due to the environment near the intestinal epithelium being less favorable for GS1 *C. concisus* to grow as compared to the oral cavity and the intestinal lumen. Previously, we found that some *C. concisus* strains belonging to both GS1 and GS2, acquired virulence genes from bacteriophages or plasmids (Mahendran et al., 2013; Liu et al., 2016). Given that *C. concisus* detected in intestinal biopsies was predominantly GS2, GS2 *C. concisus* strains that have virulence genes are more likely to cause enteric diseases involving damaging the intestinal epithelial cells.

We also analyzed the composition of isolated GS1 and GS2 *C. concisus* strains in previously reported studies (Table 4). *C. concisus* strains isolated from the human oral cavity, including both individuals with enteric diseases and healthy individuals, all had more GS2, with the difference in patients with IBD being statistically significant (Figure 2A). Thus, the molecular methods and bacterial isolation consistently detected more GS2 than GS1 *C. concisus* in the human oral cavity. *C. concisus* strains isolated from the fecal samples of patients with IBD and gastroenteritis also had more GS2 than GS1 *C. concisus* strains, consistent with GS2/GS1 ratios detected using molecular methods in the fecal samples from patients with IBD in this study (Figure 2B, Table 4). A significantly higher GS1 than GS2 *C. concisus* was isolated from fecal samples of healthy controls, which was different from the *C. concisus* strains isolated from the oral samples and the detection by molecular methods (Figure 2, Table 4). One possible explanation is that the GS2 *C. concisus* strains in the oral cavity of the healthy individuals are less resistant to the enteric environment as compared to that of the patients with enteric diseases. Alternatively, the enteric environment of patients with IBD and gastroenteritis may be different from that of the healthy controls, the latter may be less favorable to the growth of GS2 *C. concisus* strains which in turn affects the isolation rate of GS2 from fecal samples. Up until now, only three *C. concisus* strains isolated from intestinal tissues of patient with CD had known GS1 and GS2 identities, all were GS2 strains (Table 4). Kirk et al. isolated larger numbers of *C. concisus* strains from intestinal tissues of patients with IBD and healthy controls, however the GS1 and GS2 identities of these strains were not reported (Kirk et al., 2016). Given the small numbers of *C. concisus* strains isolated from intestinal biopsies with known GS1 and GS2 identities, we did not compare their GS 1 and GS2 composition with what was detected by the quantitative PCR methods.

In summary, this study identified *C. concisus* GS1 and GS2 specific polymorphisms in 23S rRNA gene and *C. concisus* GS1- and GS2-specific PCR methods were developed. Using these PCR methods, GS1 and GS2 *C. concisus* in samples collected from the upper and lower human gastrointestinal tract for the first time were quantified. We also analyzed the GS1 and GS2 composition of the isolated *C. concisus* strains in previous studies. Based on the quantitative PCR methods, there was more GS2 than GS1 *C. concisus* in samples collected from the upper and lower gastrointestinal tract of both patients with IBD and healthy controls, showing that GS2 *C. concisus* is better adapted to the human gastrointestinal tract. Analysis of GS1 and GS2 composition of the isolated *C. concisus* strains in previous studies showed similar findings except that in healthy individuals a significantly lower GS2 than GS1 *C. concisus* strains were isolated from fecal samples, suggesting a potential difference in the *C. concisus* strains or the enteric environment between patients with gastrointestinal diseases and healthy controls. This study provides novel information regarding the adaptation of different genomospecies of *C. concisus* in the human gastrointestinal tract.

## Ethics statement

The ethics approval was granted by the Ethics Committees of the University of New SouthWales and the South East Sydney Area Health Service, Australia [HREC 09237/SESIAHS 09/078 and HREC08335/SESIAHS (CHN)07/48]. Written informed consent was obtained from all subjects.

## Author contributions

Conceived and designed the experiments: LZ, YW, FL, SR, MG. Performed the experiments: YW, FL, HC, RM, SL. Analyzed the data: YW, FL XZ, SZ. Wrote the paper: YW, FL, LZ, XZ, HC, SR, MG, SZ, and RM. All authors have approved the final version of the manuscript.

### Conflict of interest statement

The authors declare that the research was conducted in the absence of any commercial or financial relationships that could be construed as a potential conflict of interest.

## Supplementary material

### Availability of data and materials

The dataset supporting the conclusions of this article is included within the article.

Supplementary Figure 1 Phylogenetic trees generated based on 23S rRNA gene and housekeeping genes for 13 *C. concisus* strains sequenced in this study. The phylogenetic trees of 23S rRNA gene **(A)** and six housekeeping genes (*asd, aspA, atpA, glnA, pgi, and tkt*) **(B)** were generated using maximum likelihood method. *C. concisus* strains were consistently divided into two genomospecies (GS). *C. concisus* strains isolated from patients with CD, UC, and healthy controls were colored in red, blue, and green, respectively. *C. concisus* strains ATCC 33237 and 13826 were used as representative strains for GS1 and GS2, respectively. Bootstrap values higher than 70 were shown. *Campylobacter jejuni* strain NCTC 11168 was used as outgroups.

Supplementary Figure 2 Comparison of 23S rRNA gene from 49 *C. concisus* strains. The 23S rRNA gene sequences were compared using MEGA7 and aligned using Clustal Omega (Sievers et al., 2011). GS1 are shaded in gray. Genomospecies specific nucleotide polymorphisms are boxed. PCR primers used in this study are shaded in blue.

Supplementary Figure 3 Validation of GS1-PCR and GS2-PCR specificities and comparison of previously published PCR primers with primers designed in this study in use for GS1-rtPCR and GS2-rtPCR. **(A)** The positive PCR products obtained from GS1-PCR and GS-2 PCR revealed a single band on agarose gel with expected sizes, which were confirmed to be *C. concisus* 23S rRNA gene by sequencing. −, Negative control (No DNA template). S, Sample. +, Positive control (DNA template that carried GS1 or GS2 *C. concisus* DNA) **(B)** Primer dimers were frequently observed in GS1-rtPCR and GS2-rtPCR when previously published primers were used (right). Primer dimers were absent when primers designed in this study were used (left).

## Abbreviations

IBD: inflammatory bowel disease
CD: Crohn's disease
UC: ulcerative colitis
GS: genomospecies
GS1-PCR: Polymerase chain reaction for *C. concisus* genomospecies 1
GS2-PCR: Polymerase chain reaction for *C. concisus* genomospecies 2.

## References

[B1] Aabenhus R. On S. L. Siemer B. L. Permin H. Andersen L. P. (2005). Delineation of *Campylobacter concisus* genomospecies by amplified fragment length polymorphism analysis and correlation of results with clinical data. J. Clin. Microbiol. 43, 5091–5096. 10.1128/JCM.43.10.5091-5096.2005 16207968 PMC1248439

[B2] Bastyns K. Chapelle S. Vandamme P. Goossens H. De Wachter R. (1995). Specific detection of *Campylobacter concisus* by PCR amplification of 23S rDNA areas. Mol. Cell. Probes 9, 247–250. 10.1016/S0890-8508(95)90114-0 7477020

[B3] Chung H. K. Tay A. Octavia S. Chen J. Liu F. Ma R.. (2016). Genome analysis of *Campylobacter concisus* strains from patients with inflammatory bowel disease and gastroenteritis provides new insights into pathogenicity. Sci. Rep 6:38442. 10.1038/srep38442 27910936 PMC5133609

[B4] Delamarche C. Thomas D. Rolland J. P. Froger A. Gouranton J. Svelto M.. (1999). Visualization of AqpZ-mediated water permeability in *Escherichia coli* by cryoelectron microscopy. J. Bacteriol. 181, 4193–4197. 10400575 10.1128/jb.181.14.4193-4197.1999 PMC93919

[B5] Deshpande N. P. Kaakoush N. O. Wilkins M. R. Mitchell H. M. (2013). Comparative genomics of *Campylobacter concisus* isolates reveals genetic diversity and provides insights into disease association. BMC Genomics 14:585. 10.1186/1471-2164-14-585 23984967 PMC3765806

[B6] Dhanasekaran S. Doherty T. M. Kenneth J. Group T. B. T. S. (2010). Comparison of different standards for real-time PCR-based absolute quantification. J. Immunol. Methods 354, 34–39. 10.1016/j.jim.2010.01.004 20109462

[B7] Engberg J. Bang D. D. Aabenhus R. Aarestrup F. M. Fussing V. Gerner-Smidt P. (2005). *Campylobacter concisus*: an evaluation of certain phenotypic and genotypic characteristics. Clin. Microbiol. Infect. 11, 288–295. 10.1111/j.1469-0691.2005.01111.x 15760425

[B8] Fite A. MacFarlane G. T. Cummings J. H. Hopkins M. J. Kong S. C. Furrie E.. (2004). Identification and quantitation of mucosal and faecal desulfovibrios using real time polymerase chain reaction. Gut 53, 523–529. 10.1136/gut.2003.031245 15016746 PMC1774019

[B9] Ismail Y. Lee H. Riordan S. M. Grimm M. C. Zhang L. (2013). The effects of oral and enteric *Campylobacter concisus* strains on expression of TLR4, MD-2, TLR2, TLR5 and COX-2 in HT-29 cells. PLoS ONE 8:e56888. 10.1371/journal.pone.0056888 23437263 PMC3577652

[B10] Ismail Y. Mahendran V. Octavia S. Day A. S. Riordan S. M. Grimm M. C.. (2012). Investigation of the enteric pathogenic potential of oral *Campylobacter concisus* strains isolated from patients with inflammatory bowel disease. PLoS ONE 7:e38217. 10.1371/journal.pone.0038217 22666490 PMC3364211

[B11] Istivan T. S. Coloe P. J. Fry B. N. Ward P. Smith S. C. (2004). Characterization of a haemolytic phospholipase A(2) activity in clinical isolates of *Campylobacter concisus.* J. Med. Microbiol. 53, 483–493. 10.1099/jmm.0.45554-0 15150326

[B12] Kalischuk L. Inglis G. (2011). Comparative genotypic and pathogenic examination of *Campylobacter concisus* isolates from diarrheic and non-diarrheic humans. BMC Microbiol. 11:53. 10.1186/1471-2180-11-53 21406111 PMC3068073

[B13] Kirk K. F. Nielsen H. L. Thorlacius-Ussing O. Nielsen H. (2016). Optimized cultivation of *Campylobacter concisus* from gut mucosal biopsies in inflammatory bowel disease. Gut Pathog. 8, 27. 10.1186/s13099-016-0111-7 27252786 PMC4888738

[B14] Kumar S. Stecher G. Tamura K. (2016). MEGA7: molecular evolutionary genetics analysis version 7.0 for bigger datasets. Mol. Biol. Evol. 33, 1870–1874. 10.1093/molbev/msw054 27004904 PMC8210823

[B15] Lastovica A. J. On S. L. Zhang L. (2014). The Family Campylobacteraceae, in The Prokaryotes: Deltaproteobacteria and Epsilonproteobacteria, eds Rosenberg E. DeLong E. F. Lory S. Stackebrandt E. Thompson F. (Berlin; Heidelberg: Springer), 307–335.

[B16] Lee H. Ma R. Grimm M. C. Riordan S. M. Lan R. Zhong L.. (2014). Examination of the anaerobic growth of *Campylobacter concisus* strains. Int. J. Microbiol. 2014:476047. 10.1155/2014/476047 25214843 PMC4158115

[B17] Lindblom G. Sjogren E. Hansson-Westerberg J. Kaijser B. (1995). Campylobacter upsaliensis, *C. sputorum* sputorum and *C. concisus* as common causes of diarrhoea in Swedish children. Scand. J. Infect Dis. 27, 187–188. 10.3109/00365549509019006 7660089

[B18] Liu F. Lee H. Lan R. Zhang L. (2016). Zonula occludens toxins and their prophages in *Campylobacter* species. Gut. Pathog. 8, 1–11. 10.1186/s13099-016-0125-1 27651834 PMC5025632

[B19] Liu F. Ma R. Riordan S. M. Grimm M. C. Liu L. Wang Y.. (2017). Azathioprine, mercaptopurine, and 5-aminosalicylic acid affect the growth of IBD-associated *Campylobacter* species and other enteric microbes. Front. Microbiol. 8:527. 10.3389/fmicb.2017.00527 28424670 PMC5372805

[B20] Ma R. Sapwell N. Chung H. K. Lee H. Mahendran V. Leong R. W.. (2015). Investigation of the effects of pH and bile on the growth of oral *Campylobacter concisus* strains isolated from patients with inflammatory bowel disease and controls. J. Med. Microbiol. 64, 438–445. 10.1099/jmm.0.000013 25657299

[B21] Mahendran V. Liu F. Riordan S. Grimm M. Tanaka M. Zhang L. (2016). Examination of the effects of *Campylobacter concisus* zonula occludens toxin on intestinal epithelial cells and macrophages. Gut. Pathog. 8, 18. 10.1186/s13099-016-0101-9 27195022 PMC4870807

[B22] Mahendran V. Octavia S. Demirbas O. F. Sabrina S. Ma R. Lan R.. (2015). Delineation of genetic relatedness and population structure of oral and enteric *Campylobacter concisus* strains by analysis of housekeeping genes. Microbiology 161, 1600–1612. 10.1099/mic.0.000112 26002953

[B23] Mahendran V. Riordan S. M. Grimm M. C. Tran T. A. Major J. Kaakoush N. O.. (2011). Prevalence of *Campylobacter* species in adult Crohn's disease and the preferential colonization sites of *Campylobacter* species in the human intestine. PLoS ONE 6:e25417. 10.1371/journal.pone.0025417 21966525 PMC3179513

[B24] Mahendran V. Tan Y. S. Riordan S. M. Grimm M. C. Day A. S. Lemberg D. A.. (2013). The prevalence and polymorphisms of zonula occluden toxin gene in multiple *Campylobacter concisus* strains isolated from saliva of patients with inflammatory bowel disease and controls. PLoS ONE 8:e75525. 10.1371/journal.pone.0075525 24086553 PMC3781098

[B25] Miller W. G. Chapman M. H. Yee E. On S. L. McNulty D. K. Lastovica A. J.. (2012). Multilocus sequence typing methods for the emerging *Campylobacter* Species *C. hyointestinalis, C. lanienae, C. sputorum, C. concisus*, and *C. curvus.* Front. Cell Infect. Microbiol. 2:45. 10.3389/fcimb.2012.00045 22919636 PMC3417633

[B26] Mukhopadhya I. Thomson J. M. Hansen R. Berry S. H. El-Omar E. M. Hold G. L. (2011). Detection of *Campylobacter concisus* and other *Campylobacter* species in colonic biopsies from adults with ulcerative colitis. PLoS ONE 6:e21490. 10.1371/journal.pone.0021490 21738679 PMC3124515

[B27] Nielsen H. Ejlertsen T. Engberg J. Nielsen H. (2013). High incidence of *Campylobacter concisus* in gastroenteritis in North Jutland, Denmark: a population-based study. Clin. Microbiol. Infect. 19, 445–450. 10.1111/j.1469-0691.2012.03852.x 22512739

[B28] Nielsen H. L. Nielsen H. Ejlertsen T. Engberg J. Günzel D. Zeitz M.. (2011). Oral and fecal *Campylobacter concisus* strains perturb barrier function by apoptosis induction in HT-29/B6 intestinal epithelial cells. PLoS ONE 6, e23858. 10.1371/journal.pone.0023858 21887334 PMC3161070

[B29] Nielsen H. L. Nielsen H. Torpdahl M. (2016). Multilocus sequence typing of *Campylobacter concisus* from Danish diarrheic patients. Gut Pathog. 8, 44. 10.1186/s13099-016-0126-0 27688814 PMC5034547

[B30] On S. Siemer B. Chung P. Lastovica A. (2013). Characterisation of *Campylobacter concisus* strains from South Africa using amplified fragment length polymorphism (AFLP) profiling and a genomospecies-specific polymerase chain reaction (PCR) assay: identification of novel genomospecies and correlation with clinical data. Afr. J. Microbiol Res. 7, 1845–1851. 10.5897/AJMR12.2182

[B31] Sartor R. B. Mazmanian S. K. (2012). Intestinal microbes in inflammatory bowel diseases. Am. J. Gastroenterol. Suppl. 1, 15–21. 10.1038/ajgsup.2012.4

[B32] Sievers F. Wilm A. Dineen D. Gibson T. J. Karplus K. Li W. Z.. (2011). Fast, scalable generation of high-quality protein multiple sequence alignments using Clustal Omega. Mol. Syst. Biol. 7, 539. 10.1038/msb.2011.75 21988835 PMC3261699

[B33] Tanner A. C. R. Badger S. Lai C. Listgarten M. A. Visconti R. A. Socransky S. S. (1981). *Wolinella* gen. nov., *WoZinella succinogenes (Vibrio succinogenes* Wolin et al.) comb. nov., and Description of *Bacteroides gracilis* sp. nov., *Wolinella recta* sp. nov., *Campylobacter concisus* sp. nov., and Eikenella corrodens from Humans with Periodontal Disease. Int. J. Syst. Bacteriol. 31, 432–445. 10.1099/00207713-31-4-432

[B34] Ye J. Coulouris G. Zaretskaya I. Cutcutache I. Rozen S. Madden T. L. (2012). Primer-BLAST: a tool to design target-specific primers for polymerase chain reaction. BMC Bioinformatics 13:1. 10.1186/1471-2105-13-134 22708584 PMC3412702

[B35] Zhang L. (2015). Oral *Campylobacter* species: initiators of a subgroup of inflammatory bowel disease? World J. Gastroenterol. 21, 9239–9244. 10.3748/wjg.v21.i31.9239 26309350 PMC4541376

[B36] Zhang L. Budiman V. Day A. S. Mitchell H. Lemberg D. A. Riordan S. M.. (2010). Isolation and detection of *Campylobacter concisus* from saliva of healthy individuals and patients with inflammatory bowel disease. J. Clin. Microbiol. 48, 2965–2967. 10.1128/JCM.02391-09 20519479 PMC2916630

[B37] Zhang L. Lee H. Grimm M. C. Riordan S. M. Day A. S. Lemberg D. A. (2014). *Campylobacter concisus* and inflammatory bowel disease. World J. Gastroenterol. 20, 1259–1267. 10.3748/wjg.v20.i5.1259 24574800 PMC3921508

[B38] Zhang L. Man S. M. Day A. S. Leach S. T. Lemberg D. A. Dutt S.. (2009). Detection and isolation of *Campylobacter* species other than *C. jejuni* from children with Crohn's disease. J. Clin. Microbiol. 47, 453–455. 10.1128/JCM.01949-08 19052183 PMC2643684

