# Supplementary figures and images for: Campylobacter concisus Genomospecies 2 Is Better Adapted to the Human Gastrointestinal Tract as Compared with Campylobacter concisus Genomospecies 1
Source: Front Physiol. 2017 Aug 3;8:543. doi: 10.3389/fphys.2017.00543 (PMC5541300; doi:10.3389/fphys.2017.00543)

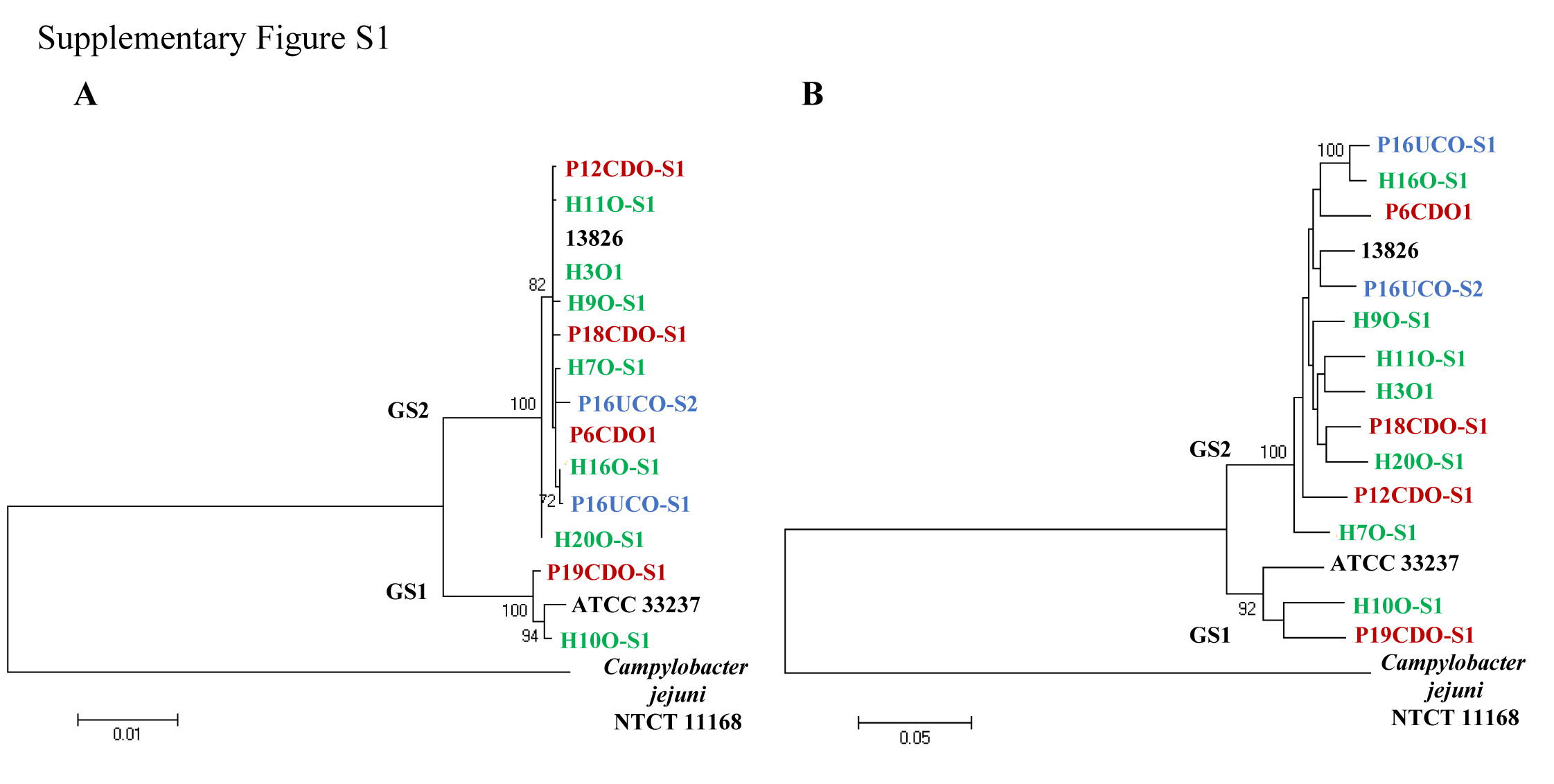

Supplement: Supplementary Figure 1 — Phylogenetic trees generated based on 23S rRNA gene and housekeeping genes for 13 C. concisus strains sequenced in this study. The phylogenetic trees of 23S rRNA gene (A) and six housekeeping genes (asd, aspA, atpA, glnA, pgi, and tkt) (B) were generated using maximum likelihood method. C. concisus strains were consistently divided into two genomospecies (GS). C. concisus strains isolated from patients with CD, UC, and healthy controls were colored in red, blue, and green, respectively. C. concisus strains ATCC 33237 and 13826 were used as representative strains for GS1 and GS2, respectively. Bootstrap values higher than 70 were shown. Campylobacter jejuni strain NCTC 11168 was used as outgroups. [file Image1.tif]

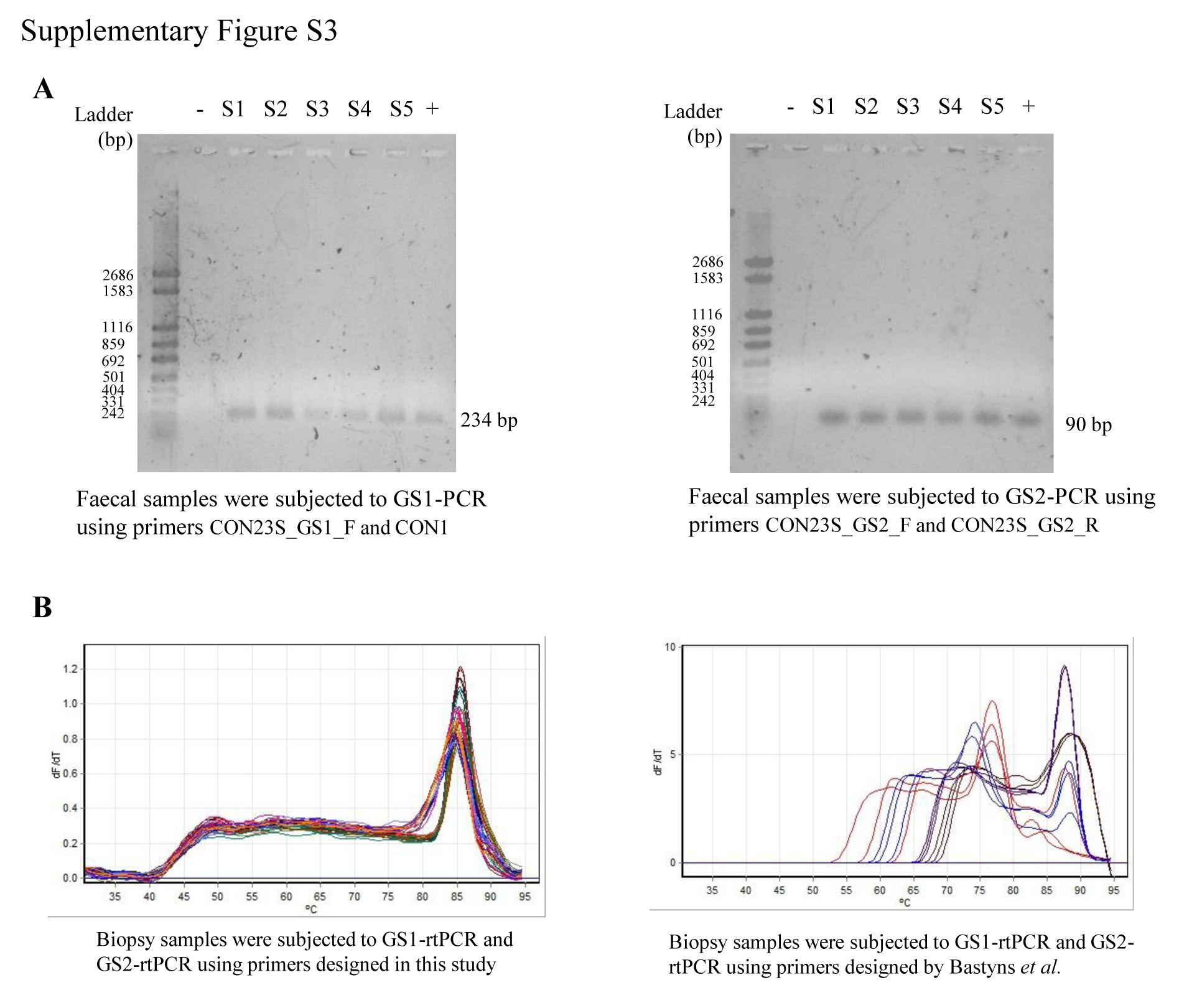

Supplement: Supplementary Figure 3 — Validation of GS1-PCR and GS2-PCR specificities and comparison of previously published PCR primers with primers designed in this study in use for GS1-rtPCR and GS2-rtPCR. (A) The positive PCR products obtained from GS1-PCR and GS-2 PCR revealed a single band on agarose gel with expected sizes, which were confirmed to be C. concisus 23S rRNA gene by sequencing. −, Negative control (No DNA template). S, Sample. +, Positive control (DNA template that carried GS1 or GS2 C. concisus DNA) (B) Primer dimers were frequently observed in GS1-rtPCR and GS2-rtPCR when previously published primers were used (right). Primer dimers were absent when primers designed in this study were used (left). [file Image3.tif]
