# Supplementary material for: Campylobacter concisus Genomospecies 2 Is Better Adapted to the Human Gastrointestinal Tract as Compared with Campylobacter concisus Genomospecies 1
Source: Front Physiol. 2017 Aug 3;8:543. doi: 10.3389/fphys.2017.00543 (PMC5541300; doi:10.3389/fphys.2017.00543)
Supplement: Supplementary Figure 2 — Comparison of 23S rRNA gene from 49 C. concisus strains. The 23S rRNA gene sequences were compared using MEGA7 and aligned using Clustal Omega (Sievers et al., 2011). GS1 are shaded in gray. Genomospecies specific nucleotide polymorphisms are boxed. PCR primers used in this study are shaded in blue. [file Image2.pdf]

Supplementary Figure S2

```
ATCC33237       GTAAGCTACAAAGAGCAAGTGGTGGATGCCTTGGCTAGTAGAGGCGATGAAAGACGTGCC 60
ATCC51562       ---AGCTACAAAGAGCAAGTGGTGGATGCCTTGGCTAGTAGAGGCGATGAAAGACGTGCC 57
H1O1            GTAAGCTACAAAGAGCAAGTGGTGGATGCCTTGGCTAGTAGAGGCGATGAAAGACGTGCC 60
H10O-S1         ---AGCTACAAAGAGCAAGTGGTGGATGCCTTGGCTAGTAGAGGCGATGAAAGACGTGCC 57
H17O-S1         GTAAGCTACAAAGAGCAAGTGGTGGATGCCTTGGCTAGTAGAGGCGATGAAAGACGTGCC 60
H21O-S3         GTAAGCTACAAAGAGCAAGTGGTGGATGCCTTGGCTAGTAGAGGCGATGAAAGACGTGCC 60
P3UCO1          GTAAGCTACAAAGAGCAAGTGGTGGATGCCTTGGCTAGTAGAGGCGATGAAAGACGTGCC 60
P3UCB1          GTAAGCTACAAAGAGCAAGTGGTGGATGCCTTGGCTAGTAGAGGCGATGAAAGACGTGCC 60
P9CDO-S1        ---AGCTACAAAGAGCAAGTGGTGGATGCCTTGGCTAGTAGAGGCGATGAAAGACGTGCC 57
P20CDO-S4       GTAAGCTACAAAGAGCAAGTGGTGGATGCCTTGGCTAGTAGAGGCGATGAAAGACGTGCC 60
13826           GTAAGCTACAAAGAGCAAGTGGTGGATGCCTTGGCTAGTAGAGGCGATGAAAGACGTGCC 60
ATCC51561       ---AGCTACAAAGAGCAAGTGGTGGATGCCTTGGCTAGTAGAGGCGATGAAAGACGTGCC 57
H3O1            ---AGCTACAAAGAGCAAGTGGTGGATGCCTTGGCTAGTAGAGGCGATGAAAGACGTGCC 57
H7O-S1          ---AGCTACAAAGAGCAAGTGGTGGATGCCTTGGCTAGTAGAGGCGATGAAAGACGTGCC 57
H9O-S1          ---AGCTACAAAGAGCAAGTGGTGGATGCCTTGGCTAGTAGAGGCGATGAAAGACGTGCC 57
H9O-S2          GTAAGCTACAAAGAGCAAGTGGTGGATGCCTTGGCTAGTAGAGGCGATGAAAGACGTGCC 60
H11O-S1         ---AGCTACAAAGAGCAAGTGGTGGATGCCTTGGCTAGTAGAGGCGATGAAAGACGTGCC 57
H14O-S1         GTAAGCTACAAAGAGCAAGTGGTGGATGCCTTGGCTAGTAGAGGCGATGAAAGACGTGCC 60
H16O-S1         ---AGCTACAAAGAGCAAGTGGTGGATGCCTTGGCTAGTAGAGGCGATGAAAGACGTGCC 57
H20O-S1         ---AGCTACAAAGAGCAAGTGGTGGATGCCTTGGCTAGTAGAGGCGATGAAAGACGTGCC 57
H21O-S1         GTAAGCTACAAAGAGCAAGTGGTGGATGCCTTGGCTAGTAGAGGCGATGAAAGACGTGCC 60
H21O-S2         GTAAGCTACAAAGAGCAAGTGGTGGATGCCTTGGCTAGTAGAGGCGATGAAAGACGTGCC 60
H21O-S5         GTAAGCTACAAAGAGCAAGTGGTGGATGCCTTGGCTAGTAGAGGCGATGAAAGACGTGCC 60
H22O-S1         GTAAGCTACAAAGAGCAAGTGGTGGATGCCTTGGCTAGTAGAGGCGATGAAAGACGTGCC 60
H23O-S1         GTAAGCTACAAAGAGCAAGTGGTGGATGCCTTGGCTAGTAGAGGCGATGAAAGACGTGCC 60
P2CDO3          GTAAGCTACAAAGAGCAAGTGGTGGATGCCTTGGCTAGTAGAGGCGATGAAAGACGTGCC 60
P2CDO4          GTAAGCTACAAAGAGCAAGTGGTGGATGCCTTGGCTAGTAGAGGCGATGAAAGACGTGCC 60
P2CDO-S6        GTAAGCTACAAAGAGCAAGTGGTGGATGCCTTGGCTAGTAGAGGCGATGAAAGACGTGCC 60
P6CDO1          ---AGCTACAAAGAGCAAGTGGTGGATGCCTTGGCTAGTAGAGGCGATGAAAGACGTGCC 57
P12CDO-S1       ---AGCTACAAAGAGCAAGTGGTGGATGCCTTGGCTAGTAGAGGCGATGAAAGACGTGCC 57
P13UCO-S3       GTAAGCTACAAAGAGCAAGTGGTGGATGCCTTGGCTAGTAGAGGCGATGAAAGACGTGCC 60
P15UCO-S2       GTAAGCTACAAAGAGCAAGTGGTGGATGCCTTGGCTAGTAGAGGCGATGAAAGACGTGCC 60
P16UCO-S1       ---AGCTACAAAGAGCAAGTGGTGGATGCCTTGGCTAGTAGAGGCGATGAAAGACGTGCC 57
P16UCO-S2       ---AGCTACAAAGAGCAAGTGGTGGATGCCTTGGCTAGTAGAGGCGATGAAAGACGTGCC 57
P18CDO-S1       ---AGCTACAAAGAGCAAGTGGTGGATGCCTTGGCTAGTAGAGGCGATGAAAGACGTGCC 57
P20CDO-S1       GTAAGCTACAAAGAGCAAGTGGTGGATGCCTTGGCTAGTAGAGGCGATGAAAGACGTGCC 60
P20CDO-S2       GTAAGCTACAAAGAGCAAGTGGTGGATGCCTTGGCTAGTAGAGGCGATGAAAGACGTGCC 60
P20CDO-S3       GTAAGCTACAAAGAGCAAGTGGTGGATGCCTTGGCTAGTAGAGGCGATGAAAGACGTGCC 60
P21CDO-S1       GTAAGCTACAAAGAGCAAGTGGTGGATGCCTTGGCTAGTAGAGGCGATGAAAGACGTGCC 60
P21CDO-S2       GTAAGCTACAAAGAGCAAGTGGTGGATGCCTTGGCTAGTAGAGGCGATGAAAGACGTGCC 60
P21CDO-S4       GTAAGCTACAAAGAGCAAGTGGTGGATGCCTTGGCTAGTAGAGGCGATGAAAGACGTGCC 60
P24CDO-S2       GTAAGCTACAAAGAGCAAGTGGTGGATGCCTTGGCTAGTAGAGGCGATGAAAGACGTGCC 60
P24CDO-S3       GTAAGCTACAAAGAGCAAGTGGTGGATGCCTTGGCTAGTAGAGGCGATGAAAGACGTGCC 60
P24CDO-S4       GTAAGCTACAAAGAGCAAGTGGTGGATGCCTTGGCTAGTAGAGGCGATGAAAGACGTGCC 60
UNSW1           GTAAGCTACAAAGAGCAAGTGGTGGATGCCTTGGCTAGTAGAGGCGATGAAAGACGTGCC 60
UNSW2           GTAAGCTACAAAGAGCAAGTGGTGGATGCCTTGGCTAGTAGAGGCGATGAAAGACGTGCC 60
UNSW3           GTAAGCTACAAAGAGCAAGTGGTGGATGCCTTGGCTAGTAGAGGCGATGAAAGACGTGCC 60
UNSWCD          ---AGCTACAAAGAGCAAGTGGTGGATGCCTTGGCTAGTAGAGGCGATGAAAGACGTGCC 57
UNSWCS          GTAAGCTACAAAGAGCAAGTGGTGGATGCCTTGGCTAGTAGAGGCGATGAAAGACGTGCC 60
                   *********************************************************
```

Supplementary Figure S2

```
ATCC33237       AGGCTGCGATAAGTCTCGGGGAGCCGTCAAGGGGCTTTGATCCGGGAATTTCTGAATGGG 120
ATCC51562       AGGCTGCGATAAGTCTCGGGGAGCCGTCAAGGGGCTTTGATCCGGGAATTTCTGAATGGG 117
H1O1            AGGCTGCGATAAGTCTCGGGGAGCCGTCAAGGGGCTTTGATCCGGGAATTTCTGAATGGG 120
H10O-S1         AGGCTGCGATAAGTCTCGGGGAGCCGTCAAGGGGCTTTGATCCGGGAATTTCTGAATGGG 117
H17O-S1         AGGCTGCGATAAGTCTCGGGGAGCCGTCAAGGGGCTTTGATCCGGGAATTTCTGAATGGG 120
H21O-S3         AGGCTGCGATAAGTCTCGGGGAGCCGTCAAGGGGCTTTGATCCGGGAATTTCTGAATGGG 120
P3UCO1          AGGCTGCGATAAGTCTCGGGGAGCCGTCAAGGGGCTTTGATCCGGGAATTTCTGAATGGG 120
P3UCB1          AGGCTGCGATAAGTCTCGGGGAGCCGTCAAGGGGCTTTGATCCGGGAATTTCTGAATGGG 120
P9CDO-S1        AGGCTGCGATAAGTCTCGGGGAGCCGTCAAGGGGCTTTGATCCGGGAATTTCTGAATGGG 117
P20CDO-S4       AGGCTGCGATAAGTCTCGGGGAGCCGTCAAGGGGCTTTGATCCGGGAATTTCTGAATGGG 120
13826           AGGCTGCGATAAGTCTCGGGGAGCCGTCAAGGGGCTTTGATCCGGGAATTTCTGAATGGG 120
ATCC51561       AGGCTGCGATAAGTCTCGGGGAGCCGTCAAGGGGCTTTGATCCGGGAATTTCTGAATGGG 117
H3O1            AGGCTGCGATAAGTCTCGGGGAGCCGTCAAGGGGCTTTGATCCGGGAATTTCTGAATGGG 117
H7O-S1          AGGCTGCGATAAGTCTCGGGGAGCCGTCAAGGGGCTTTGATCCGGGAATTTCTGAATGGG 117
H9O-S1          AGGCTGCGATAAGTCTCGGGGAGCCGTCAAGGGGCTTTGATCCGGGAATTTCTGAATGGG 117
H9O-S2          AGGCTGCGATAAGTCTCGGGGAGCCGTCAAGGGGCTTTGATCCGGGAATTTCTGAATGGG 120
H11O-S1         AGGCTGCGATAAGTCTCGGGGAGCCGTCAAGGGGCTTTGATCCGGGAATTTCTGAATGGG 117
H14O-S1         AGGCTGCGATAAGTCTCGGGGAGCCGTCAAGGGGCTTTGATCCGGGAATTTCTGAATGGG 120
H16O-S1         AGGCTGCGATAAGTCTCGGGGAGCCGTCAAGGGGCTTTGATCCGGGAATTTCTGAATGGG 117
H20O-S1         AGGCTGCGATAAGTCTCGGGGAGCCGTCAAGGGGCTTTGATCCGGGAATTTCTGAATGGG 117
H21O-S1         AGGCTGCGATAAGTCTCGGGGAGCCGTCAAGGGGCTTTGATCCGGGAATTTCTGAATGGG 120
H21O-S2         AGGCTGCGATAAGTCTCGGGGAGCCGTCAAGGGGCTTTGATCCGGGAATTTCTGAATGGG 120
H21O-S5         AGGCTGCGATAAGTCTCGGGGAGCCGTCAAGGGGCTTTGATCCGGGAATTTCTGAATGGG 120
H22O-S1         AGGCTGCGATAAGTCTCGGGGAGCCGTCAAGGGGCTTTGATCCGGGAATTTCTGAATGGG 120
H23O-S1         AGGCTGCGATAAGTCTCGGGGAGCCGTCAAGGGGCTTTGATCCGGGAATTTCTGAATGGG 120
P2CDO3          AGGCTGCGATAAGTCTCGGGGAGCCGTCAAGGGGCTTTGATCCGGGAATTTCTGAATGGG 120
P2CDO4          AGGCTGCGATAAGTCTCGGGGAGCCGTCAAGGGGCTTTGATCCGGGAATTTCTGAATGGG 120
P2CDO-S6        AGGCTGCGATAAGTCTCGGGGAGCCGTCAAGGGGCTTTGATCCGGGAATTTCTGAATGGG 120
P6CDO1          AGGCTGCGATAAGTCTCGGGGAGCCGTCAAGGGGCTTTGATCCGGGAATTTCTGAATGGG 117
P12CDO-S1       AGGCTGCGATAAGTCTCGGGGAGCCGTCAAGGGGCTTTGATCCGGGAATTTCTGAATGGG 117
P13UCO-S3       AGGCTGCGATAAGTCTCGGGGAGCCGTCAAGGGGCTTTGATCCGGGAATTTCTGAATGGG 120
P15UCO-S2       AGGCTGCGATAAGTCTCGGGGAGCCGTCAAGGGGCTTTGATCCGGGAATTTCTGAATGGG 120
P16UCO-S1       AGGCTGCGATAAGTCTCGGGGAGCCGTCAAGGGGCTTTGATCCGGGAATTTCTGAATGGG 117
P16UCO-S2       AGGCTGCGATAAGTCTCGGGGAGCCGTCAAGGGGCTTTGATCCGGGAATTTCTGAATGGG 117
P18CDO-S1       AGGCTGCGATAAGTCTCGGGGAGCCGTCAAGGGGCTTTGATCCGGGAATTTCTGAATGGG 117
P20CDO-S1       AGGCTGCGATAAGTCTCGGGGAGCCGTCAAGGGGCTTTGATCCGGGAATTTCTGAATGGG 120
P20CDO-S2       AGGCTGCGATAAGTCTCGGGGAGCCGTCAAGGGGCTTTGATCCGGGAATTTCTGAATGGG 120
P20CDO-S3       AGGCTGCGATAAGTCTCGGGGAGCCGTCAAGGGGCTTTGATCCGGGAATTTCTGAATGGG 120
P21CDO-S1       AGGCTGCGATAAGTCTCGGGGAGCCGTCAAGGGGCTTTGATCCGGGAATTTCTGAATGGG 120
P21CDO-S2       AGGCTGCGATAAGTCTCGGGGAGCCGTCAAGGGGCTTTGATCCGGGAATTTCTGAATGGG 120
P21CDO-S4       AGGCTGCGATAAGTCTCGGGGAGCCGTCAAGGGGCTTTGATCCGGGAATTTCTGAATGGG 120
P24CDO-S2       AGGCTGCGATAAGTCTCGGGGAGCCGTCAAGGGGCTTTGATCCGGGAATTTCTGAATGGG 120
P24CDO-S3       AGGCTGCGATAAGTCTCGGGGAGCCGTCAAGGGGCTTTGATCCGGGAATTTCTGAATGGG 120
P24CDO-S4       AGGCTGCGATAAGTCTCGGGGAGCCGTCAAGGGGCTTTGATCCGGGAATTTCTGAATGGG 120
UNSW1           AGGCTGCGATAAGTCTCGGGGAGCCGTCAAGGGGCTTTGATCCGGGAATTTCTGAATGGG 120
UNSW2           AGGCTGCGATAAGTCTCGGGGAGCCGTCAAGGGGCTTTGATCCGGGAATTTCTGAATGGG 120
UNSW3           AGGCTGCGATAAGTCTCGGGGAGCCGTCAAGGGGCTTTGATCCGGGAATTTCTGAATGGG 120
UNSWCD          AGGCTGCGATAAGTCTCGGGGAGCCGTCAAGGGGCTTTGATCCGGGAATTTCTGAATGGG 117
UNSWCS          AGGCTGCGATAAGTCTCGGGGAGCCGTCAAGGGGCTTTGATCCGGGAATTTCTGAATGGG 120
                ************************************************************
```

Supplementary Figure S2

```
ATCC33237       GCAACCCAGTTAAGCGCGAGCTTAACTACCTAAT-ATGGAGCGAACGAGGGGAATTGAAA 179
ATCC51562       GCAACCCAGTTAAGCGTGAGCTTAACTACCTAAT-ATGGAGCGAACGAGGGGAATTGAAA 176
H1O1            GCAACCCAACTAATAGAGATATTAGTTACCGTATAACGGAGCGAACGAGGGGAATTGAAA 180
H10O-S1         GCAACCCAGTTAAGCGCGAGTTTAACTACCTAAT-ATGGAGCGAACGAGGGGAATTGAAA 176
H17O-S1         GCAACCCAACTAATAGAGATATTAGTTACCGTATAACGGAGCGAACGAGGGGAATTGAAA 180
H21O-S3         GCAACCCAACTAATAGAGATATTAGTTACCGTATAACGGAGCGAACGAGGGGAATTGAAA 180
P3UCO1          GCAACCCAGTTAAGCGCGAGCTTAACTACCTAAT-ATGGAGCGAACGAGGGGAATTGAAA 179
P3UCB1          GCAACCCAGTTAAGCGCGAGCTTAACTACCTAAT-ATGGAGCGAACGAGGGGAATTGAAA 179
P9CDO-S1        GCAACCCAGTTAAGCGCGAGCTTAACTACCTAAT-ATGGAGCGAACGAGGGGAATTGAAA 176
P20CDO-S4       GCAACCCAGTTAAGCGCGAGCTTAACTACCTAAT-ATGGAGCGAACGAGGGGAATTGAAA 179
13826           GCAACCCAACTAATAGAGATATTAGTTACCGTATAACGGAGCGAACGAGGGGAATTGAAA 180
ATCC51561       GCAACCCAACTAATAGAGATATTAGTTACCGTATAACGGAGCGAACGAGGGGAATTGAAA 177
H3O1            GCAACCCAACTAATAGAGATATTAGTTACCGTATAACGGAGCGAACGAGGGGAATTGAAA 177
H7O-S1          GCAACCCAACTAATAGAGATATTAGTTACCGTATAACGGAGCGAACGAGGGGAATTGAAA 177
H9O-S1          GCAACCCAACTAATAGAGATATTAGTTACCGTATAACGGAGCGAACGAGGGGAATTGAAA 177
H9O-S2          GCAACCCAACTAATAGAGATATTAGTTACCGTATAACGGAGCGAACGAGGGGAATTGAAA 180
H11O-S1         GCAACCCAACTAATAGAGATATTAGTTACCGTATAACGGAGCGAACGAGGGGAATTGAAA 177
H14O-S1         GCAACCCAGTTAAGCGCGAGCTTAACTACCTAAT-ATGGAGCGAACGAGGGGAATTGAAA 179
H16O-S1         GCAACCCAACTAATAGAGATATTAGTTACCGTATAACGGAGCGAACGAGGGGAATTGAAA 177
H20O-S1         GCAACCCAACTAATAGAGATATTAGTTACCGTATAACGGAGCGAACGAGGGGAATTGAAA 177
H21O-S1         GCAACCCAACTAATAGAGATATTAGTTACCGTATAACGGAGCGAACGAGGGGAATTGAAA 180
H21O-S2         GCAACCCAACTAATAGAGATATTAGTTACCGTATAACGGAGCGAACGAGGGGAATTGAAA 180
H21O-S5         GCAACCCAACTAATAGAGATATTAGTTACCGTATAACGGAGCGAACGAGGGGAATTGAAA 180
H22O-S1         GCAACCCAACTAATAGAGATATTAGTTACCGTATAACGGAGCGAACGAGGGGAATTGAAA 180
H23O-S1         GCAACCCAACTAATAGAGATATTAGTTACCGTATAACGGAGCGAACGAGGGGAATTGAAA 180
P2CDO3          GCAACCCAACTAATAGAGATATTAGTTACCGTATAACGGAGCGAACGAGGGGAATTGAAA 180
P2CDO4          GCAACCCAACTAATAGAGATATTAGTTACCGTATAACGGAGCGAACGAGGGGAATTGAAA 180
P2CDO-S6        GCAACCCAACTAATAGAGATATTAGTTACCGTATAACGGAGCGAACGAGGGGAATTGAAA 180
P6CDO1          GCAACCCAACTAATAGAGATATTAGTTACCGTATAACGGAGCGAACGAGGGGAATTGAAA 177
P12CDO-S1       GCAACCCAACTAATAGAGATATTAGTTACCGTATAACGGAGCGAACGAGGGGAATTGAAA 177
P13UCO-S3       GCAACCCAACTAATAGAGATATTAGTTACCGTATAACGGAGCGAACGAGGGGAATTGAAA 180
P15UCO-S2       GCAACCCAACTAATAGAGATATTAGTTACCGTATAACGGAGCGAACGAGGGGAATTGAAA 180
P16UCO-S1       GCAACCCAACTAATAGAGATATTAGTTACCGTATAACGGAGCGAACGAGGGGAATTGAAA 177
P16UCO-S2       GCAACCCAACTAATAGAGATATTAGTTACCGTATAACGGAGCAAACGAGGGGAATTGAAA 177
P18CDO-S1       GCAACCCAACTAATAGAGATATTAGTTACCGTATAACGGAGCGAACGAGGGGAATTGAAA 177
P20CDO-S1       GCAACCCAACTAATAGAGATATTAGTTACCGTATAACGGAGCGAACGAGGGGAATTGAAA 180
P20CDO-S2       GCAACCCAACTAATAGAGATATTAGTTACCGTATAACGGAGCGAACGAGGGGAATTGAAA 180
P20CDO-S3       GCAACCCAACTAATAGAGATATTAGTTACCGTATAACGGAGCGAACGAGGGGAATTGAAA 180
P21CDO-S1       GCAACCCAGTTAAGCGCGAGCTTAACTACCTAAT-ATGGAGCGAACGAGGGGAATTGAAA 179
P21CDO-S2       GCAACCCAACTAATAGAGATATTAGTTACCGTATAACGGAGCGAACGAGGGGAATTGAAA 180
P21CDO-S4       GCAACCCAACTAATAGAGATATTAGTTACCGTATAACGGAGCGAACGAGGGGAATTGAAA 180
P24CDO-S2       GCAACCCAACTAATAGAGATATTAGTTACCGTATAACGGAGCGAACGAGGGGAATTGAAA 180
P24CDO-S3       GCAACCCAACTAATAGAGATATTAGTTACCGTATAACGGAGCGAACGAGGGGAATTGAAA 180
P24CDO-S4       GCAACCCAACTAATAGAGATATTAGTTACCGTATAACGGAGCGAACGAGGGGAATTGAAA 180
UNSW1           GCAACCCAACTAATAGAGATATTAGTTACCGTATAACGGAGCGAACGAGGGGAATTGAAA 180
UNSW2           GCAACCCAACTAATAGAGATATTAGTTACCGTATAACGGAGCGAACGAGGGGAATTGAAA 180
UNSW3           GCAACCCAACTAATAGAGATATTAGTTACCGTATAACGGAGCGAACGAGGGGAATTGAAA 180
UNSWCD          GCAACCCAACTAATAGAGATATTAGTTACCGTATAACGGGGCGAACGAGGGGAATTGAAA 177
UNSWCS          GCAACCCAACTAATAGAGATATTAGTTACCGTATAACGGAGCGAACGAGGGGAATTGAAA 180
                ********  ***  * **  ***  ****  ** * ** ** *****************
```

Supplementary Figure S2

```
ATCC33237       CATCTTAGTACCCTCAGGAAAAGAAATCAAAAGAGATTACGCTAGTAGCGGCGAGCGAAC 239
ATCC51562       CATCTTAGTACCCTCAGGAAAAGAAATCAAAAGAGATTACGCTAGTAGCGGCGAGCGAAC 236
H1O1            CATCTTAGTACCCTCAGGAAAAGAAATCAAAAGAGATTACGCTAGTAGCGGCGAGCGAAC 240
H10O-S1         CATCTTAGTACCCTCAGGAAAAGAAATCAAAAGAGATTACGCTAGTAGCGGCGAGCGAAC 236
H17O-S1         CATCTTAGTACCCTCAGGAAAAGAAATCAAAAGAGATTACGCTAGTAGCGGCGAGCGAAC 240
H21O-S3         CATCTTAGTACCCTCAGGAAAAGAAATCAAAAGAGATTACGCTAGTAGCGGCGAGCGAAC 240
P3UCO1          CATCTTAGTACCCTCAGGAAAAGAAATCAAAAGAGATTACGCTAGTAGCGGCGAGCGAAC 239
P3UCB1          CATCTTAGTACCCTCAGGAAAAGAAATCAAAAGAGATTACGCTAGTAGCGGCGAGCGAAC 239
P9CDO-S1        CATCTTAGTACCCTCAGGAAAAGAAATCAAAAGAGATTACGCTAGTAGCGGCGAGCGAAC 236
P20CDO-S4       CATCTTAGTACCCTCAGGAAAAGAAATCAAAAGAGATTACGCTAGTAGCGGCGAGCGAAC 239
13826           CATCTTAGTACCCTCAGGAAAAGAAATCAAAAGAGATTACGCTAGTAGCGGCGAGCGAAC 240
ATCC51561       CATCTTAGTACCCTCAGGAAAAGAAATCAAAAGAGATTACGCTAGTAGCGGCGAGCGAAC 237
H3O1            CATCTTAGTACCCTCAGGAAAAGAAATCAAAAGAGATTACGCTAGTAGCGGCGAGCGAAC 237
H7O-S1          CATCTTAGTACCCTCAGGAAAAGAAATCAAAAGAGATTACGCTAGTAGCGGCGAGCGAAC 237
H9O-S1          CATCTTAGTACCCTCAGGAAAAGAAATCAAAAGAGATTACGCTAGTAGCGGCGAGCGAAC 237
H9O-S2          CATCTTAGTACCCTCAGGAAAAGAAATCAAAAGAGATTACGCTAGTAGCGGCGAGCGAAC 240
H11O-S1         CATCTTAGTACCCTCAGGAAAAGAAATCAAAAGAGATTACGCTAGTAGCGGCGAGCGAAC 237
H14O-S1         CATCTTAGTACCCTCAGGAAAAGAAATCAAAAGAGATTACGCTAGTAGCGGCGAGCGAAC 239
H16O-S1         CATCTTAGTACCCTCAGGAAAAGAAATCAAAAGAGATTACGCTAGTAGCGGCGAGCGAAC 237
H20O-S1         CATCTTAGTACCCTCAGGAAAAGAAATCAAAAGAGATTACGCTAGTAGCGGCGAGCGAAC 237
H21O-S1         CATCTTAGTACCCTCAGGAAAAGAAATCAAAAGAGATTACGCTAGTAGCGGCGAGCGAAC 240
H21O-S2         CATCTTAGTACCCTCAGGAAAAGAAATCAAAAGAGATTACGCTAGTAGCGGCGAGCGAAC 240
H21O-S5         CATCTTAGTACCCTCAGGAAAAGAAATCAAAAGAGATTACGCTAGTAGCGGCGAGCGAAC 240
H22O-S1         CATCTTAGTACCCTCAGGAAAAGAAATCAAAAGAGATTACGCTAGTAGCGGCGAGCGAAC 240
H23O-S1         CATCTTAGTACCCTCAGGAAAAGAAATCAAAAGAGATTACGCTAGTAGCGGCGAGCGAAC 240
P2CDO3          CATCTTAGTACCCTCAGGAAAAGAAATCAAAAGAGATTACGCTAGTAGCGGCGAGCGAAC 240
P2CDO4          CATCTTAGTACCCTCAGGAAAAGAAATCAAAAGAGATTACGCTAGTAGCGGCGAGCGAAC 240
P2CDO-S6        CATCTTAGTACCCTCAGGAAAAGAAATCAAAAGAGATTACGCTAGTAGCGGCGAGCGAAC 240
P6CDO1          CATCTTAGTACCCTCAGGAAAAGAAATCAAAAGAGATTACGCTAGTAGCGGCGAGCGAAC 237
P12CDO-S1       CATCTTAGTACCCTCAGGAAAAGAAATCAAAAGAGATTACGCTAGTAGCGGCGAGCGAAC 237
P13UCO-S3       CATCTTAGTACCCTCAGGAAAAGAAATCAAAAGAGATTACGCTAGTAGCGGCGAGCGAAC 240
P15UCO-S2       CATCTTAGTACCCTCAGGAAAAGAAATCAAAAGAGATTACGCTAGTAGCGGCGAGCGAAC 240
P16UCO-S1       CATCTTAGTACCCTCAGGAAAAGAAATCAAAAGAGATTACGCTAGTAGCGGCGAGCGAAC 237
P16UCO-S2       CATCTTAGTACCCTCAGGAAAAGAAATCAAAAGAGATTACGCTAGTAGCGGCGAGCGAAC 237
P18CDO-S1       CATCTTAGTACCCTCAGGAAAAGAAATCAAAAGAGATTACGCTAGTAGCGGCGAGCGAAC 237
P20CDO-S1       CATCTTAGTACCCTCAGGAAAAGAAATCAAAAGAGATTACGCTAGTAGCGGCGAGCGAAC 240
P20CDO-S2       CATCTTAGTACCCTCAGGAAAAGAAATCAAAAGAGATTACGCTAGTAGCGGCGAGCGAAC 240
P20CDO-S3       CATCTTAGTACCCTCAGGAAAAGAAATCAAAAGAGATTACGCTAGTAGCGGCGAGCGAAC 240
P21CDO-S1       CATCTTAGTACCCTCAGGAAAAGAAATCAAAAGAGATTACGCTAGTAGCGGCGAGCGAAC 239
P21CDO-S2       CATCTTAGTACCCTCAGGAAAAGAAATCAAAAGAGATTACGCTAGTAGCGGCGAGCGAAC 240
P21CDO-S4       CATCTTAGTACCCTCAGGAAAAGAAATCAAAAGAGATTACGCTAGTAGCGGCGAGCGAAC 240
P24CDO-S2       CATCTTAGTACCCTCAGGAAAAGAAATCAAAAGAGATTACGCTAGTAGCGGCGAGCGAAC 240
P24CDO-S3       CATCTTAGTACCCTCAGGAAAAGAAATCAAAAGAGATTACGCTAGTAGCGGCGAGCGAAC 240
P24CDO-S4       CATCTTAGTACCCTCAGGAAAAGAAATCAAAAGAGATTACGCTAGTAGCGGCGAGCGAAC 240
UNSW1           CATCTTAGTACCCTCAGGAAAAGAAATCAAAAGAGATTACGCTAGTAGCGGCGAGCGAAC 240
UNSW2           CATCTTAGTACCCTCAGGAAAAGAAATCAAAAGAGATTACGCTAGTAGCGGCGAGCGAAC 240
UNSW3           CATCTTAGTACCCTCAGGAAAAGAAATCAAAAGAGATTACGCTAGTAGCGGCGAGCGAAC 240
UNSWCD          CATCTTAGTACCCTCAGGAAAAGAAATCAAAAGAGATTACGCTAGTAGCGGCGAGCGAAC 237
UNSWCS          CATCTTAGTACCCTCAGGAAAAGAAATCAAAAGAGATTACGCTAGTAGCGGCGAGCGAAC 240
                ************************************************************
```

Supplementary Figure S2

```
ATCC33237        GCGTAAGAGGGCAAACCGTTAGTTTACTAACGGGGTTGTAGGACTGCAATATAGACTAAA 299
ATCC51562        GCGTAAGAGGGCAAACCGTTAGTTTACTAACGGGGTTGTAGGACTGCAATATAGACTAAA 296
H1O1             GCGTAAGAGGGCAAACCGTTAGTTTACTAACGGGGTTGTAGGACTGCAATATAGACTAAA 300
H10O-S1          GCGTAAGAGGGCAAACCGTTAGTTTACTAACGGGGTTGTAGGACTGCAATATAGACTAAA 296
H17O-S1          GCGTAAGAGGGCAAACCGTTAGTTTACTAACGGGGTTGTAGGACTGCAATATAGACTAAA 300
H21O-S3          GCGTAAGAGGGCAAACCGTTAGTTTACTAACGGGGTTGTAGGACTGCAATATAGACTAAA 300
P3UCO1           GCGTAAGAGGGCAAACCGTTAGTTTACTAACGGGGTTGTAGGACTGCAATATAGACTAAA 299
P3UCB1           GCGTAAGAGGGCAAACCGTTAGTTTACTAACGGGGTTGTAGGACTGCAATATAGACTAAA 299
P9CDO-S1         GCGTAAGAGGGCAAACCGTTAGTTTACTAACGGGGTTGTAGGACTGCGATATAGACTAAA 296
P20CDO-S4        GCGTAAGAGGGCAAACCGTTAGTTTACTAACGGGGTTGTAGGACTGCAATATAGACTAAA 299
13826            GCGTAAGAGGGCAAACCACTAGTTTACTAGTGGGGTTGTAGGACTGCAATATAGACTAAA 300
ATCC51561        GCGTAAGAGGGCAAACCACTAGTTTACTAGTGGGGTTGTAGGACTGCGATATAGACTAAA 297
H3O1             GCGTAAGAGGGCAAACCACTAGTTTACTAGTGGGGTTGTAGGACTGCAATATAGACTAAA 297
H7O-S1           GCGTAAGAGGGCAAACCACTAGTTTACTAGTGGGGTTGTAGGACTGCAATATAGACTAAA 297
H9O-S1           GCGTAAGAGGGCAAACCACTAGTTTACTAGTGGGGTTGTAGGACTGCAATATAGACTAAA 297
H9O-S2           GCGTAAGAGGGCAAACCACTAGTTTACTAGTGGGGTTGTAGGACTGCGATATAGACTAAA 300
H11O-S1          GCGTAAGAGGGCAAACCACTAGTTTACTAGTGGGGTTGTAGGACTGCAATATAGACTAAA 297
H14O-S1          GCGTAAGAGGGCAAACCACTAGTTTACTAGTGGGGTTGTAGGACTGCAATATAGACTAAA 299
H16O-S1          GCGTAAGAGGGCAAACCACTAGTTTACTAGTGGGGTTGTAGGACTGCAATATAGACTAAA 297
H20O-S1          GCGTAAGAGGGCAAACCACTAGTTTACTAGTGGGGTTGTAGGACTGCAATATAGACTAAA 297
H21O-S1          GCGTAAGAGGGCAAACCACTAGTTTACTAGTGGGGTTGTAGGACTGCAATATAGACTAAA 300
H21O-S2          GCGTAAGAGGGCAAACCACTAGTTTACTAGTGGGGTTGTAGGACTGCAATATAGACTAAA 300
H21O-S5          GTGTAAGAGGGCAAACCACTAGTTTACTAGTGGGGTTGTAGGACTGCAATATAGACTAAA 300
H22O-S1          GCGTAAGAGGGCAAACCACTAGTTTACTAGTGGGGTTGTAGGACTGCAATATAGACTAAA 300
H23O-S1          GCGTAAGAGGGCAAACCACTAGTTTACTAGTGGGGTTGTAGGACTGCAATATAGACTAAA 300
P2CDO3           GCGTAAGAGGGCAAACCACTAGTTTACTAGTGGGGTTGTAGGACTGCAATATAGACTAAA 300
P2CDO4           GCGTAAGAGGGCAAACCACTAGTTTACTAGTGGGGTTGTAGGACTGCAATATAGACTAAA 300
P2CDO-S6         GCGTAAGAGGGCAAACCACTAGTTTACTAGTGGGGTTGTAGGACTGCAATATAGACTAAA 300
P6CDO1           GCGTAAGAGGGCAAACCACTAGTTTACTAGTGGGGTTGTAGGACTGCAATATAGACTAAA 297
P12CDO-S1        GCGTAAGAGGGCAAACCACTAGTTTACTAGTGGGGTTGTAGGACTGCAATATAGACTAAA 297
P13UCO-S3        GCGTAAGAGGGCAAACCACTAGTTTACTAGTGGGGTTGTAGGACTGCAATATAGACTAAA 300
P15UCO-S2        GCGTAAGAGGGCAAACCACTAGTTTACTAGTGGGGTTGTAGGACTGCAATATAGACTAAA 300
P16UCO-S1        GCGTAAGAGGGCAAACCACTAGTTTACTAGTGGGGTTGTAGGACTGCAATATAGACTAAA 297
P16UCO-S2        GCGTAAGAGGGCAAACCACTAGTTTACTAGTGGGGTTGTAGGACTGCAATATAGACTAAA 297
P18CDO-S1        GCGTAAGAGGGCAAACCACTAGTTTACTAGTGGGGTTGTAGGACTGCAATATAGACTAAA 297
P20CDO-S1        GCGTAAGAGGGCAAACCACTAGTTTACTAGTGGGGTTGTAGGACTGCAATATAGACTAAA 300
P20CDO-S2        GCGTAAGAGGGCAAACCACTAGTTTACTAGTGGGGTTGTAGGACTGCAATATAGACTAAA 300
P20CDO-S3        GCGTAAGAGGGCAAACCACTAGTTTACTAGTGGGGTTGTAGGACTGCAATATAGACTAAA 300
P21CDO-S1        GCGTAAGAGGGCAAACCACTAGTTTACTAGTGGGGTTGTAGGACTGCAATATAGACTAAA 299
P21CDO-S2        GCGTAAGAGGGCAAACCACTAGTTTACTAGTGGGGTTGTAGGACTGCAATATAGACTAAA 300
P21CDO-S4        GCGTAAGAGGGCAAACCACTAGTTTACTAGTGGGGTTGTAGGACTGCAATATAGACTAAA 300
P24CDO-S2        GCGTAAGAGGGCAAACCACTAGTTTACTAGTGGGGTTGTAGGACTGCAATATAGACTAAA 300
P24CDO-S3        GCGTAAGAGGGCAAACCACTAGTTTACTAGTGGGGTTGTAGGACTGCAATATAGACTAAA 300
P24CDO-S4        GCGTAAGAGGGCAAACCACTAGTTTACTAGTGGGGTTGTAGGACTGCAATATAGACTAAA 300
UNSW1            GCGTAAGAGGGCAAACCACTAGTTTACTAGTGGGGTTGTAGGACTGCAATATAGACTAAA 300
UNSW2            GCGTAAGAGGGCAAACCACTAGTTTACTAGTGGGGTTGTAGGACTGCAATATAGACTAAA 300
UNSW3            GCGTAAGAGGGCAAACCACTAGTTTACTAGTGGGGTTGTAGGACTGCAATATAGACTAAA 300
UNSWCD           GCGTAAGAGGGCAAACCACTAGTTTACTAGTGGGGTTGTAGGACTGCAATATAGACTAAA 297
UNSWCS           GCGTAAGAGGGCAAACCACTAGTTTACTAGTGGGGTTGTAGGACTGCAATATAGACTAAA 300
                 * ***************  **********  **************** ************
```

Supplementary Figure S2

```
ATCC33237       CTTAGCTAATAGAATAATTTGGAAAGATTAAGCATAGAGGGTGATACTCCCGTATATGAA 359
ATCC51562       CTTAGCTAATAGAATAATCTGGAAAGATTAAGCATAGAGGGTGATACTCCCGTATATGAA 356
H1O1            CTTAGCTAATAGAATAATCTGGAAAGATTAAGCGTAGAGGGTGATACTCCCGTATATGAA 360
H10O-S1         CTTAGCTAATAGAATAATTTGGAAAGATTAAGCATAGAGGGTGATACTCCCGTATATGAA 356
H17O-S1         CTTAGCTAATAGAATAATCTGGAAAGATTAAGCATAGAGGGTGATACTCCCGTATATGAA 360
H21O-S3         CTTAGCTAATAGAATAATCTGGAAAGATTAAGCATAGAGGGTGATACTCCCGTATATGAA 360
P3UCO1          CTTAGCTAATAGAATAACCTGGAAAGGTTGAGCGTAGAGGGTGATACTCCCGTATATGAA 359
P3UCB1          CTTAGCTAATAGAATAACCTGGAAAGGTTGAGCGTAGAGGGTGATACTCCCGTATATGAA 359
P9CDO-S1        CTTAGCTAATAGAATAATCTGGAAAGATTAAGCATAGAGGGTGATACTCCCGTATATGAA 356
P20CDO-S4       CTTAGCTAATAGAACAACCTGGAAAGGTTGAGCATAGAGGGTGATACTCCCGTATATGAA 359
13826           CTTAGCTAATAGAACAACCTGGAAAGGTTAGGCACAGAGGGTGATACCCCCGTATATGAA 360
ATCC51561       CTTAGCTAATAGAACAACCTGGAAAGGTTGGGCACAGAGGGTGATACCCCCGTATATGAA 357
H3O1            CTTAGCTAATAGAACAACCTGGAAAGGTTAGGCACAGAGGGTGATACCCCCGTATATGAA 357
H7O-S1          CTTAGCTAATAGAACGACCTGGAAAGGTTAGGCACAGAGGGTGATACCCCCGTATATGAA 357
H9O-S1          CTTAGCTAATAGAATAACCTGGAAAGGTTAGGCACAGAGGGTGATACCCCCGTATATGAA 357
H9O-S2          CTTAGCTAATAGAACAACCTGGAAAGGTTGGGCACAGAGGGTGATACCCCCGTATATGAA 360
H11O-S1         CTTAGCTAATAGAACAACCTGGAAAGGTTAGGCACAGAGGGTGATACCCCCGTATATGAA 357
H14O-S1         CTTAGCTAATAGAACGACCTGGAAAGGTTAGGCACAGAGGGTGATACCCCCGTATATGAA 359
H16O-S1         CTTAGCTAATAGAACGACCTGGAAAGGTTAGGCACAGAGGGTGATACCCCCGTATATGAA 357
H20O-S1         CTTAGCTAATAGAACAACCTGGAAAGGTTAGGCACAGAGGGTGATACCCCCGTATATGAA 357
H21O-S1         CTTAGCTAATAGAACAACCTGGAAAGGTTAGGCACAGAGGGTGATACCCCCGTATATGAA 360
H21O-S2         CTTAGCTAATAGAACAACCTGGAAAGGTTAGGCACAGAGGGTGATACCCCCGTATATGAA 360
H21O-S5         CTTAGCTAATAGAACGACCTGGAAAGGTTAGGCACAGAGGGTGATACCCCCGTATATGAA 360
H22O-S1         CTTAGCTAATAGAACGACCTGGAAAGGTTAGGCACAGAGGGTGATACCCCCGTATATGAA 360
H23O-S1         CTTAGCTAATAGAACGACCTGGAAAGGTTAGGCACAGAGGGTGATACCCCCGTATATGAA 360
P2CDO3          CTTAGCTAATAGAACGACCTGGAAAGGTTAGGCACAGAGGGTGATACCCCCGTATATGAA 360
P2CDO4          CTTAGCTAATAGAACAACCTGGAAAGGTTAGGCACAGAGGGTGATACCCCCGTATATGAA 360
P2CDO-S6        CTTAGCTAATAGAACGACCTGGAAAGGTTAGGCACAGAGGGTGATACCCCCGTATATGAA 360
P6CDO1          CTTAGCTAATAGAACGACCTGGAAAGGTTAGGCACAGAGGGTGATACCCCCGTATATGAA 357
P12CDO-S1       CTTAGCTAATAGAACAACCTGGAAAGGTTAGGCACAGAGGGTGATACCCCCGTATATGAA 357
P13UCO-S3       CTTAGCTAATAGAACGACCTGGAAAGGTTAGGCACAGAGGGTGATACCCCCGTATATGAA 360
P15UCO-S2       CTTAGCTAATAGAACGACCTGGAAAGGTTAGGCACAGAGGGTGATACCCCCGTATATGAA 360
P16UCO-S1       CTTAGCTAATAGAACGACCTGGAAAGGTTAGGCACAGAGGGTGATACCCCCGTATATGAA 357
P16UCO-S2       CTTAGCTAATAGAACGACCTGGAAAGGTTAGGCACAGAGGGTGATACCCCCGTATATGAA 357
P18CDO-S1       CTTAGCTAATAGAACAACCTGGAAAGGTTGGGCACAGAGGGTGATACCCCCGTATATGAA 357
P20CDO-S1       CTTAGCTAATAGAACAACCTGGAAAGGTTAGGCACAGAGGGTGATACCCCCGTATATGAA 360
P20CDO-S2       CTTAGCTAATAGAATAACCTGGAAAGGTTAGGCACAGAGGGTGATACCCCCGTATATGAA 360
P20CDO-S3       CTTAGCTAATAGAACAACCTGGAAAGGTTAGGCACAGAGGGTGATACCCCCGTATATGAA 360
P21CDO-S1       CTTAGCTAATAGAACGACCTGGAAAGGTTAGGCACAGAGGGTGATACCCCCGTATATGAA 359
P21CDO-S2       CTTAGCTAATAGAATAACTTGGAAAGGTTAGGCATAGAGGGTGATACCCCCGTATATGAA 360
P21CDO-S4       CTTAGCTAATAGAACAACCTGGAAAGGTTAGGCACAGAGGGTGATACCCCCGTATATGAA 360
P24CDO-S2       CTTAGCTAATAGAACGACCTGGAAAGGTTAGGCACAGAGGGTGATACCCCCGTATATGAA 360
P24CDO-S3       CTTAGCTAATAGAACGACCTGGAAAGGTTAGGCACAGAGGGTGATACCCCCGTATATGAA 360
P24CDO-S4       CTTAGCTAATAGAACGACCTGGAAAGGTTAGGCACAGAGGGTGATACCCCCGTATATGAA 360
UNSW1           CTTAGCTAATAGAACAACCTGGAAAGGTTAGGCACAGAGGGTGATACCCCCGTATATGAA 360
UNSW2           CTTAGCTAATAGAACAACCTGGAAAGGTTAGGCACAGAGGGTGATACCCCCGTATATGAA 360
UNSW3           CTTAGCTAATAGAACAACCTGGAAAGGTTAGGCACAGAGGGTGATACCCCCGTATATGAA 360
UNSWCD          CTTAGCTAATAGAACGACCTGGAAAGGTTAGGCACAGAGGGTGATACCCCCGTATATGAA 357
UNSWCS          CTTAGCTAATAGAACAACCTGGAAAGGTTAGGCACAGAGGGTGATACCCCCGTATATGAA 360
                **************  *  ******* **  **  ************ ************
```

Supplementary Figure S2

```
ATCC33237       AGCTTTGTTTTACTTAGCAGTATCCTGAGTAGGGCGGAACACGTGATATTCTGTCTGAAG 419
ATCC51562       AGCTTTGTTTTACTTAGCAGTATCCTGAGTAGGGCGGAACACGTGATATTCTGTCTGAAG 416
H1O1            AGCTTTGTTTTACTTAGCAGTATCCTGAGTAGGGCGGAACACGTGATATTCTGTCTGAAG 420
H10O-S1         AGCTTTGTTTTACTTAGCAGTATCCTGAGTAGGGCGGAACACGTGATATTCTGTCTGAAG 416
H17O-S1         AGCTTTGTTTTACTTAGCAGTATCCTGAGTAGGGCGGAACACGTGATATTCTGTCTGAAG 420
H21O-S3         AGCTTTGTTTTACTTAGCAGTATCCTGAGTAGGGCGGAACACGTGATATTCTGTCTGAAG 420
P3UCO1          AGCTTTGTTTTACTTAGCAGTATCCTGAGTAGGGCGGAACACGTGATATTCTGTCTGAAG 419
P3UCB1          AGCTTTGTTTTACTTAGCAGTATCCTGAGTAGGGCGGAACACGTGATATTCTGTCTGAAG 419
P9CDO-S1        AGCTTTGTTTTACTTAGCAGTATCCTGAGTAGGGCGGAACACGTGATATTCTGTCTGAAG 416
P20CDO-S4       AGCTTTGTTTTACTTAGCAGTATCCTGAGTAGGGCGGAACACGTGATATTCTGTCTGAAG 419
13826           AGCTTTGTTTTACTTAGCAGTATCCTGAGTAGGGCGGAACACGTGATATTCTGTCTGAAG 420
ATCC51561       AGCTTTGTTTTACTTAGCAGTATCCTGAGTAGGGCGGAACACGTGATATTCTGTCTGAAG 417
H3O1            AGCTTTGTTTTACTTAGCAGTATCCTGAGTAGGGCGGAACACGTGATATTCTGTCTGAAG 417
H7O-S1          AGCTTTGTTTTACTTAGCAGTATCCTGAGTAGGGCGGAACACGTGATATTCTGTCTGAAG 417
H9O-S1          AGCTTTGTTTTACTTAGCAGTATCCTGAGTAGGGCGGAACACGTGATATTCTGTCTGAAG 417
H9O-S2          AGCTTTGTTTTACTTAGCAGTATCCTGAGTAGGGCGGAACACGTGATATTCTGTCTGAAG 420
H11O-S1         AGCTTTGTTTTACTTAGCAGTATCCTGAGTAGGGCGGAACACGTGATATTCTGTCTGAAG 417
H14O-S1         AGCTTTGTTTTACTTAGCAGTATCCTGAGTAGGGCGGAACACGTGATATTCTGTCTGAAG 419
H16O-S1         AGCTTTGTTTTACTTAGCAGTATCCTGAGTAGGGCGGAACACGTGATATTCTGTCTGAAG 417
H20O-S1         AGCTTTGTTTTACTTAGCAGTATCCTGAGTAGGGCGGAACACGTGATATTCTGTCTGAAG 417
H21O-S1         AGCTTTGTTTTACTTAGCAGTATCCTGAGTAGGGCGGAACACGTGATATTCTGTCTGAAG 420
H21O-S2         AGCTTTGTTTTACTTAGCAGTATCCTGAGTAGGGCGGAACACGTGATATTCTGTCTGAAG 420
H21O-S5         AGCTTTGTTTTACTTAGCAGTATCCTGAGTAGGGCGGAACACGTGATATTCTGTCTGAAG 420
H22O-S1         AGCTTTGTTTTACTTAGCAGTATCCTGAGTAGGGCGGAACACGTGATATTCTGTCTGAAG 420
H23O-S1         AGCTTTGTTTTACTTAGCAGTATCCTGAGTAGGGCGGAACACGTGATATTCTGTCTGAAG 420
P2CDO3          AGCTTTGTTTTACTTAGCAGTATCCTGAGTAGGGCGGAACACGTGATATTCTGTCTGAAG 420
P2CDO4          AGCTTTGTTTTACTTAGCAGTATCCTGAGTAGGGCGGAACACGTGATATTCTGTCTGAAG 420
P2CDO-S6        AGCTTTGTTTTACTTAGCAGTATCCTGAGTAGGGCGGAACACGTGATATTCTGTCTGAAG 420
P6CDO1          AGCTTTGTTTTACTTAGCAGTATCCTGAGTAGGGCGGAACACGTGATATTCTGTCTGAAG 417
P12CDO-S1       AGCTTTGTTTTACTTAGCAGTATCCTGAGTAGGGCGGAACACGTGATATTCTGTCTGAAG 417
P13UCO-S3       AGCTTTGTTTTACTTAGCAGTATCCTGAGTAGGGCGGAACACGTGATATTCTGTCTGAAG 420
P15UCO-S2       AGCTTTGTTTTACTTAGCAGTATCCTGAGTAGGGCGGAACACGTGATATTCTGTCTGAAG 420
P16UCO-S1       AGCTTTGTTTTACTTAGCAGTATCCTGAGTAGGGCGGAACACGTGATATTCTGTCTGAAG 417
P16UCO-S2       AGCTTTGTTTTACTTAGCAGTATCCTGAGTAGGGCGGAACACGTGATATTCTGTCTGAAG 417
P18CDO-S1       AGCTTTGTTTTACTTAGCAGTATCCTGAGTAGGGCGGAACACGTGATATTCTGTCTGAAG 417
P20CDO-S1       AGCTTTGTTTTACTTAGCAGTATCCTGAGTAGGGCGGAACACGTGATATTCTGTCTGAAG 420
P20CDO-S2       AGCTTTGTTTTACTTAGCAGTATCCTGAGTAGGGCGGAACACGTGATATTCTGTCTGAAG 420
P20CDO-S3       AGCTTTGTTTTACTTAGCAGTATCCTGAGTAGGGCGGAACACGTGATATTCTGTCTGAAG 420
P21CDO-S1       AGCTTTGTTTTACTTAGCAGTATCCTGAGTAGGGCGGAACACGTGATATTCTGTCTGAAG 419
P21CDO-S2       AGCTTTGTTTTACTTAGCAGTATCCTGAGTAGGGCGGAACACGTGATATTCTGTCTGAAG 420
P21CDO-S4       AGCTTTGTTTTACTTAGCAGTATCCTGAGTAGGGCGGAACACGTGATATTCTGTCTGAAG 420
P24CDO-S2       AGCTTTGTTTTACTTAGCAGTATCCTGAGTAGGGCGGAACACGTGATATTCTGTCTGAAG 420
P24CDO-S3       AGCTTTGTTTTACTTAGCAGTATCCTGAGTAGGGCGGAACACGTGATATTCTGTCTGAAG 420
P24CDO-S4       AGCTTTGTTTTACTTAGCAGTATCCTGAGTAGGGCGGAACACGTGATATTCTGTCTGAAG 420
UNSW1           AGCTTTGTTTTACTTAGCAGTATCCTGAGTAGGGCGGAACACGTGATATTCTGTCTGAAG 420
UNSW2           AGCTTTGTTTTACTTAGCAGTATCCTGAGTAGGGCGGAACACGTGATATTCTGTCTGAAG 420
UNSW3           AGCTTTGTTTTACTTAGCAGTATCCTGAGTAGGGCGGAACACGTGATATTCTGTCTGAAG 420
UNSWCD          AGCTTTGTTTTACTTAGCAGTATCCTGAGTAGGGCGGAACACGTGATATTCTGTCTGAAG 417
UNSWCS          AGCTTTGTTTTACTTAGCAGTATCCTGAGTAGGGCGGAACACGTGATATTCTGTCTGAAG 420
                ************************************************************
```

Supplementary Figure S2

ATCC33237	CTGGGTAGACCACTATCCAACCCTAAATACTACTACTAGACCGATAGTGCACAAGTACCG	479
ATCC51562	CTGGGTAGACCACTATCCAACCCTAAATACTACTACTAGACCGATAGTGCACAAGTACCG	476
H1O1	CTGGGTAGACCACTATCCAACCCTAAATACTACTACTAGACCGATAGTGCACAAGTACCG	480
H10O-S1	CTGGGTAGACCACTATCCAACCCTAAATACTACTACTAGACCGATAGTGCACAAGTACCG	476
H17O-S1	CTGGGTAGACCACTATCCAACCCTAAATACTACTACTAGACCGATAGTGCACAAGTACCG	480
H21O-S3	CTGGGTAGACCACTATCCAACCCTAAATACTACTACTAGACCGATAGTGCACAAGTACCG	480
P3UCO1	CTGGGTAGACCACTATCCAACCCTAAATACTACTACTAGACCGATAGTGCACAAGTACCG	479
P3UCB1	CTGGGTAGACCACTATCCAACCCTAAATACTACTACTAGACCGATAGTGCACAAGTACCG	479
P9CDO-S1	CTGGGTAGACCACTATCCAACCCTAAATACTACTACTAGACCGATAGTGCACAAGTACCG	476
P20CDO-S4	CTGGGTAGACCACTATCCAACCCTAAATACTACTACTAGACCGATAGTGCACAAGTACCG	479
13826	CTGGGTAGACCACTATCCAACCCTAAATACTACTACTAGACCGATAGTGCACAAGTACCG	480
ATCC51561	CTGGGTAGACCACTATCCAACCCTAAATACTACTACTAGACCGATAGTGCACAAGTACCG	477
H3O1	CTGGGTAGACCACTATCCAACCCTAAATACTACTACTAGACCGATAGTGCACAAGTACCG	477
H7O-S1	CTGGGTAGACCACTATCCAACCCTAAATACTACTACTAGACCGATAGTGCACAAGTACCG	477
H9O-S1	CTGGGTAGACCACTATCCAACCCTAAATACTACTACTAGACCGATAGTGCACAAGTACCG	477
H9O-S2	CTGGGTAGACCACTATCCAACCCTAAATACTACTACTAGACCGATAGTGCACAAGTACCG	480
H11O-S1	CTGGGTAGACCACTATCCAACCCTAAATACTACTACTAGACCGATAGTGCACAAGTACCG	477
H14O-S1	CTGGGTAGACCACTATCCAACCCTAAATACTACTACTAGACCGATAGTGCACAAGTACCG	479
H16O-S1	CTGGGTAGACCACTATCCAACCCTAAATACTACTACTAGACCGATAGTGCACAAGTACCG	477
H20O-S1	CTGGGTAGACCACTATCCAACCCTAAATACTACTACTAGACCGATAGTGCACAAGTACCG	477
H21O-S1	CTGGGTAGACCACTATCCAACCCTAAATACTACTACTAGACCGATAGTGCACAAGTACCG	480
H21O-S2	CTGGGTAGACCACTATCCAACCCTAAATACTACTACTAGACCGATAGTGCACAAGTACCG	480
H21O-S5	CTGGGTAGACCACTATCCAACCCTAAATACTACTACTAGACCGATAGTGCACAAGTACCG	480
H22O-S1	CTGGGTAGACCACTATCCAACCCTAAATACTACTACTAGACCGATAGTGCACAAGTACCG	480
H23O-S1	CTGGGTAGACCACTATCCAACCCTAAATACTACTACTAGACCGATAGTGCACAAGTACCG	480
P2CDO3	CTGGGTAGACCACTATCCAACCCTAAATACTACTACTAGACCGATAGTGCACAAGTACCG	480
P2CDO4	CTGGGTAGACCACTATCCAACCCTAAATACTACTACTAGACCGATAGTGCACAAGTACCG	480
P2CDO-S6	CTGGGTAGACCACTATCCAACCCTAAATACTACTACTAGACCGATAGTGCACAAGTACCG	480
P6CDO1	CTGGGTAGACCACTATCCAACCCTAAATACTACTACTAGACCGATAGTGCACAAGTACCG	477
P12CDO-S1	CTGGGTAGACCACTATCCAACCCTAAATACTACTACTAGACCGATAGTGCACAAGTACCG	477
P13UCO-S3	CTGGGTAGACCACTATCCAACCCTAAATACTACTACTAGACCGATAGTGCACAAGTACCG	480
P15UCO-S2	CTGGGTAGACCACTATCCAACCCTAAATACTACTACTAGACCGATAGTGCACAAGTACCG	480
P16UCO-S1	CTGGGTAGACCACTATCCAACCCTAAATACTACTACTAGACCGATAGTGCACAAGTACCG	477
P16UCO-S2	CTGGGTAGACCACTATCCAACCCTAAATACTACTACTAGACCGATAGTGCACAAGTACCG	477
P18CDO-S1	CTGGGTAGACCACTATCCAACCCTAAATACTACTACTAGACCGATAGTGCACAAGTACCG	477
P20CDO-S1	CTGGGTAGACCACTATCCAACCCTAAATACTACTACTAGACCGATAGTGCACAAGTACCG	480
P20CDO-S2	CTGGGTAGACCACTATCCAACCCTAAATACTACTACTAGACCGATAGTGCACAAGTACCG	480
P20CDO-S3	CTGGGTAGACCACTATCCAACCCTAAATACTACTACTAGACCGATAGTGCACAAGTACCG	480
P21CDO-S1	CTGGGTAGACCACTATCCAACCCTAAATACTACTACTAGACCGATAGTGCACAAGTACCG	479
P21CDO-S2	CTGGGTAGACCACTATCCAACCCTAAATACTACTACTAGACCGATAGTGCACAAGTACCG	480
P21CDO-S4	CTGGGTAGACCACTATCCAACCCTAAATACTACTACTAGACCGATAGTGCACAAGTACCG	480
P24CDO-S2	CTGGGTAGACCACTATCCAACCCTAAATACTACTACTAGACCGATAGTGCACAAGTACCG	480
P24CDO-S3	CTGGGTAGACCACTATCCAACCCTAAATACTACTACTAGACCGATAGTGCACAAGTACCG	480
P24CDO-S4	CTGGGTAGACCACTATCCAACCCTAAATACTACTACTAGACCGATAGTGCACAAGTACCG	480
UNSW1	CTGGGTAGACCACTATCCAACCCTAAATACTACTACTAGACCGATAGTGCACAAGTACCG	480
UNSW2	CTGGGTAGACCACTATCCAACCCTAAATACTACTACTAGACCGATAGTGCACAAGTACCG	480
UNSW3	CTGGGTAGACCACTATCCAACCCTAAATACTACTACTAGACCGATAGTGCACAAGTACCG	480
UNSWCD	CTGGGTAGACCACTATCCAACCCTAAATACTACTACTAGACCGATAGTGCACAAGTACCG	477
UNSWCS	CTGGGTAGACCACTATCCAACCCTAAATACTACTACTAGACCGATAGTGCACAAGTACCG	480
	**	

Supplementary Figure S2

```
ATCC33237      TGAGGGAAAGGTGAAAAGAACTGAGGTGATCAGAGTGAAATAGAACCTGAAACCATTTGC 539
ATCC51562      TGAGGGAAAGGTGAAAAGAACTGAGGTGATCAGAGTGAAATAGAACCTGAAACCATTTGC 536
H1O1           TGAGGGAAAGGTGAAAAGAACTGAGGTGATCAGAGTGAAATAGAACCTGAAACCATTTGC 540
H10O-S1        TGAGGGAAAGGTGAAAAGAACTGAGGTGATCAGAGTGAAATAGAACCTGAAACCATTTGC 536
H17O-S1        TGAGGGAAAGGTGAAAAGAACTGAGGTGATCAGAGTGAAATAGAACCTGAAACCATTTGC 540
H21O-S3        TGAGGGAAAGGTGAAAAGAACTGAGGTGATCAGAGTGAAATAGAACCTGAAACCATTTGC 540
P3UCO1         TGAGGGAAAGGTGAAAAGAACTGAGGTGATCAGAGTGAAATAGAACCTGAAACCATTTGC 539
P3UCB1         TGAGGGAAAGGTGAAAAGAACTGAGGTGATCAGAGTGAAATAGAACCTGAAACCATTTGC 539
P9CDO-S1       TGAGGGAAAGGTGAAAAGAACTGAGGTGATCAGAGTGAAATAGAACCTGAAACCATTTGC 536
P20CDO-S4      TGAGGGAAAGGTGAAAAGAACTGAGGTGATCAGAGTGAAATAGAACCTGAAACCATTTGC 539
13826          TGAGGGAAAGGTGAAAAGAACTGAGGTGATCAGAGTGAAATAGAACCTGAAACCATTTGC 540
ATCC51561      TGAGGGAAAGGTGAAAAGAACTGAGGTGATCAGAGTGAAATAGAACCTGAAACCATTTGC 537
H3O1           TGAGGGAAAGGTGAAAAGAACTGAGGTGATCAGAGTGAAATAGAACCTGAAACCATTTGC 537
H7O-S1         TGAGGGAAAGGTGAAAAGAACTGAGGTGATCAGAGTGAAATAGAACCTGAAACCATTTGC 537
H9O-S1         TGAGGGAAAGGTGAAAAGAACTGAGGTGATCAGAGTGAAATAGAACCTGAAACCATTTGC 537
H9O-S2         TGAGGGAAAGGTGAAAAGAACTGAGGTGATCAGAGTGAAATAGAACCTGAAACCATTTGC 540
H11O-S1        TGAGGGAAAGGTGAAAAGAACTGAGGTGATCAGAGTGAAATAGAACCTGAAACCATTTGC 537
H14O-S1        TGAGGGAAAGGTGAAAAGAACTGAGGTGATCAGAGTGAAATAGAACCTGAAACCATTTGC 539
H16O-S1        TGAGGGAAAGGTGAAAAGAACTGAGGTGATCAGAGTGAAATAGAACCTGAAACCATTTGC 537
H20O-S1        TGAGGGAAAGGTGAAAAGAACTGAGGTGATCAGAGTGAAATAGAACCTGAAACCATTTGC 537
H21O-S1        TGAGGGAAAGGTGAAAAGAACTGAGGTGATCAGAGTGAAATAGAACCTGAAACCATTTGC 540
H21O-S2        TGAGGGAAAGGTGAAAAGAACTGAGGTGATCAGAGTGAAATAGAACCTGAAACCATTTGC 540
H21O-S5        TGAGGGAAAGGTGAAAAGAACTGAGGTGATCAGAGTGAAATAGAACCTGAAACCATTTGC 540
H22O-S1        TGAGGGAAAGGTGAAAAGAACTGAGGTGATCAGAGTGAAATAGAACCTGAAACCATTTGC 540
H23O-S1        TGAGGGAAAGGTGAAAAGAACTGAGGTGATCAGAGTGAAATAGAACCTGAAACCATTTGC 540
P2CDO3         TGAGGGAAAGGTGAAAAGAACTGAGGTGATCAGAGTGAAATAGAACCTGAAACCATTTGC 540
P2CDO4         TGAGGGAAAGGTGAAAAGAACTGAGGTGATCAGAGTGAAATAGAACCTGAAACCATTTGC 540
P2CDO-S6       TGAGGGAAAGGTGAAAAGAACTGAGGTGATCAGAGTGAAATAGAACCTGAAACCATTTGC 540
P6CDO1         TGAGGGAAAGGTGAAAAGAACTGAGGTGATCAGAGTGAAATAGAACCTGAAACCATTTGC 537
P12CDO-S1      TGAGGGAAAGGTGAAAAGAACTGAGGTGATCAGAGTGAAATAGAACCTGAAACCATTTGC 537
P13UCO-S3      TGAGGGAAAGGTGAAAAGAACTGAGGTGATCAGAGTGAAATAGAACCTGAAACCATTTGC 540
P15UCO-S2      TGAGGGAAAGGTGAAAAGAACTGAGGTGATCAGAGTGAAATAGAACCTGAAACCATTTGC 540
P16UCO-S1      TGAGGGAAAGGTGAAAAGAACTGAGGTGATCAGAGTGAAATAGAACCTGAAACCATTTGC 537
P16UCO-S2      TGAGGGAAAGGTGAAAAGAACTGAGGTGATCAGAGTGAAATAGAACCTGAAACCATTTGC 537
P18CDO-S1      TGAGGGAAAGGTGAAAAGAACTGAGGTGATCAGAGTGAAATAGAACCTGAAACCATTTGC 537
P20CDO-S1      TGAGGGAAAGGTGAAAAGAACTGAGGTGATCAGAGTGAAATAGAACCTGAAACCATTTGC 540
P20CDO-S2      TGAGGGAAAGGTGAAAAGAACTGAGGTGATCAGAGTGAAATAGAACCTGAAACCATTTGC 540
P20CDO-S3      TGAGGGAAAGGTGAAAAGAACTGAGGTGATCAGAGTGAAATAGAACCTGAAACCATTTGC 540
P21CDO-S1      TGAGGGAAAGGTGAAAAGAACTGAGGTGATCAGAGTGAAATAGAACCTGAAACCATTTGC 539
P21CDO-S2      TGAGGGAAAGGTGAAAAGAACTGAGGTGATCAGAGTGAAATAGAACCTGAAACCATTTGC 540
P21CDO-S4      TGAGGGAAAGGTGAAAAGAACTGAGGTGATCAGAGTGAAATAGAACCTGAAACCATTTGC 540
P24CDO-S2      TGAGGGAAAGGTGAAAAGAACTGAGGTGATCAGAGTGAAATAGAACCTGAAACCATTTGC 540
P24CDO-S3      TGAGGGAAAGGTGAAAAGAACTGAGGTGATCAGAGTGAAATAGAACCTGAAACCATTTGC 540
P24CDO-S4      TGAGGGAAAGGTGAAAAGAACTGAGGTGATCAGAGTGAAATAGAACCTGAAACCATTTGC 540
UNSW1          TGAGGGAAAGGTGAAAAGAACTGAGGTGATCAGAGTGAAATAGAACCTGAAACCATTTGC 540
UNSW2          TGAGGGAAAGGTGAAAAGAACTGAGGTGATCAGAGTGAAATAGAACCTGAAACCATTTGC 540
UNSW3          TGAGGGAAAGGTGAAAAGAACTGAGGTGATCAGAGTGAAATAGAACCTGAAACCATTTGC 540
UNSWCD         TGAGGGAAAGGTGAAAAGAACTGAGGTGATCAGAGTGAAATAGAACCTGAAACCATTTGC 537
UNSWCS         TGAGGGAAAGGTGAAAAGAACTGAGGTGATCAGAGTGAAATAGAACCTGAAACCATTTGC 540
               ************************************************************
```

Supplementary Figure S2

```
ATCC33237       TTACAATCATTCAGAGCCCTATGATTTATCAGGGTGATGGACTGCCTTTTGCATAATGAG 599
ATCC51562       TTACAATCATTCAGAGCCCTATGATTTATCAGGGTGATGGACTGCCTTTTGCATAATGAG 596
H1O1            TTACAATCATTCAGAGCCCTATGATTTATCAGGGTGATGGACTGCCTTTTGCATAATGAG 600
H10O-S1         TTACAATCATTCAGAGCCCTATGATTTATCAGGGTGATGGACTGCCTTTTGCATAATGAG 596
H17O-S1         TTACAATCATTCAGAGCCCTATGATTTATCAGGGTGATGGACTGCCTTTTGCATAATGAG 600
H21O-S3         TTACAATCATTCAGAGCCCTATGATTTATCAGGGTGATGGACTGCCTTTTGCATAATGAG 600
P3UCO1          TTACAATCATTCAGAGCCCTATGATTTATCAGGGTGATGGACTGCCTTTTGCATAATGAG 599
P3UCB1          TTACAATCATTCAGAGCCCTATGATTTATCAGGGTGATGGACTGCCTTTTGCATAATGAG 599
P9CDO-S1        TTACAATCATTCAGAGCCCTATGATTTATCAGGGTGATGGACTGCCTTTTGCATAATGAG 596
P20CDO-S4       TTACAATCATTCAGAGCCCTATGATTTATCAGGGTGATGGACTGCCTTTTGCATAATGAG 599
13826           TTACAATCATTCAGAGCCCTATGATTTATCAGGGTGATGGACTGCCTTTTGCATAATGAG 600
ATCC51561       TTACAATCATTCAGAGCCCTATGATTTATCAGGGTGATGGACTGCCTTTTGCATAATGAG 597
H3O1            TTACAATCATTCAGAGCCCTATGATTTATCAGGGTGATGGACTGCCTTTTGCATAATGAG 597
H7O-S1          TTACAATCATTCAGAGCCCTATGATTTATCAGGGTGATGGACTGCCTTTTGCATAATGAG 597
H9O-S1          TTACAATCATTCAGAGCCCTATGATTTATCAGGGTGATGGACTGCCTTTTGCATAATGAG 597
H9O-S2          TTACAATCATTCAGAGCCCTATGATTTATCAGGGTGATGGACTGCCTTTTGCATAATGAG 600
H11O-S1         TTACAATCATTCAGAGCCCTATGATTTATCAGGGTGATGGACTGCCTTTTGCATAATGAG 597
H14O-S1         TTACAATCATTCAGAGCCCTATGATTTATCAGGGTGATGGACTGCCTTTTGCATAATGAG 599
H16O-S1         TTACAATCATTCAGAGCCCTATGATTTATCAGGGTGATGGACTGCCTTTTGCATAATGAG 597
H20O-S1         TTACAATCATTCAGAGCCCTATGATTTATCAGGGTGATGGACTGCCTTTTGCATAATGAG 597
H21O-S1         TTACAATCATTCAGAGCCCTATGATTTATCAGGGTGATGGACTGCCTTTTGCATAATGAG 600
H21O-S2         TTACAATCATTCAGAGCCCTATGATTTATCAGGGTGATGGACTGCCTTTTGCATAATGAG 600
H21O-S5         TTACAATCATTCAGAGCCCTATGATTTATCAGGGTGATGGACTGCCTTTTGCATAATGAG 600
H22O-S1         TTACAATCATTCAGAGCCCTATGATTTATCAGGGTGATGGACTGCCTTTTGCATAATGAG 600
H23O-S1         TTACAATCATTCAGAGCCCTATGATTTATCAGGGTGATGGACTGCCTTTTGCATAATGAG 600
P2CDO3          TTACAATCATTCAGAGCCCTATGATTTATCAGGGTGATGGACTGCCTTTTGCATAATGAG 600
P2CDO4          TTACAATCATTCAGAGCCCTATGATTTATCAGGGTGATGGACTGCCTTTTGCATAATGAG 600
P2CDO-S6        TTACAATCATTCAGAGCCCTATGATTTATCAGGGTGATGGACTGCCTTTTGCATAATGAG 600
P6CDO1          TTACAATCATTCAGAGCCCTATGATTTATCAGGGTGATGGACTGCCTTTTGCATAATGAG 597
P12CDO-S1       TTACAATCATTCAGAGCCCTATGATTTATCAGGGTGATGGACTGCCTTTTGCATAATGAG 597
P13UCO-S3       TTACAATCATTCAGAGCCCTATGATTTATCAGGGTGATGGACTGCCTTTTGCATAATGAG 600
P15UCO-S2       TTACAATCATTCAGAGCCCTATGATTTATCAGGGTGATGGACTGCCTTTTGCATAATGAG 600
P16UCO-S1       TTACAATCATTCAGAGCCCTATGATTTATCAGGGTGATGGACTGCCTTTTGCATAATGAG 597
P16UCO-S2       TTACAATCATTCAGAGCCCTATGATTTATCAGGGTGATGGACTGCCTTTTGCATAATGAG 597
P18CDO-S1       TTACAATCATTCAGAGCCCTATGATTTATCAGGGTGATGGACTGCCTTTTGCATAATGAG 597
P20CDO-S1       TTACAATCATTCAGAGCCCTATGATTTATCAGGGTGATGGACTGCCTTTTGCATAATGAG 600
P20CDO-S2       TTACAATCATTCAGAGCCCTATGATTTATCAGGGTGATGGACTGCCTTTTGCATAATGAG 600
P20CDO-S3       TTACAATCATTCAGAGCCCTATGATTTATCAGGGTGATGGACTGCCTTTTGCATAATGAG 600
P21CDO-S1       TTACAATCATTCAGAGCCCTATGATTTATCAGGGTGATGGACTGCCTTTTGCATAATGAG 599
P21CDO-S2       TTACAATCATTCAGAGCCCTATGATTTATCAGGGTGATGGACTGCCTTTTGCATAATGAG 600
P21CDO-S4       TTACAATCATTCAGAGCCCTATGATTTATCAGGGTGATGGACTGCCTTTTGCATAATGAG 600
P24CDO-S2       TTACAATCATTCAGAGCCCTATGATTTATCAGGGTGATGGACTGCCTTTTGCATAATGAG 600
P24CDO-S3       TTACAATCATTCAGAGCCCTATGATTTATCAGGGTGATGGACTGCCTTTTGCATAATGAG 600
P24CDO-S4       TTACAATCATTCAGAGCCCTATGATTTATCAGGGTGATGGACTGCCTTTTGCATAATGAG 600
UNSW1           TTACAATCATTCAGAGCCCTATGATTTATCAGGGTGATGGACTGCCTTTTGCATAATGAG 600
UNSW2           TTACAATCATTCAGAGCCCTATGATTTATCAGGGTGATGGACTGCCTTTTGCATAATGAG 600
UNSW3           TTACAATCATTCAGAGCCCTATGATTTATCAGGGTGATGGACTGCCTTTTGCATAATGAG 600
UNSWCD          TTACAATCATTCAGAGCCCTATGATTTATCAGGGTGATGGACTGCCTTTTGCATAATGAG 597
UNSWCS          TTACAATCATTCAGAGCCCTATGATTTATCAGGGTGATGGACTGCCTTTTGCATAATGAG 600
                ************************************************************
```

Supplementary Figure S2

```
ATCC33237       CCTGCGAGTTGTGATGTCTGGCAAGGTTAAGGAAACCCGGAGCCGTAGCGAAAGCGAGTC 659
ATCC51562       CCTGCGAGTTGTGATGTCTGGCAAGGTTAAGGAAACCCGGAGCCGTAGCGAAAGCGAGTC 656
H1O1            CCTGCGAGTTGTGATGTCTGGCAAGGTTAAGGAAACACGGAGCCATAGCGAAAGCGAGTC 660
H10O-S1         CCTGCGAGTTGTGATGTCTGGCAAGGTTAAGGAAACCCGGAGCCGTAGCGAAAGCGAGTC 656
H17O-S1         CCTGCGAGTTGTGATGTCTGGCGAGGTTAAGGAAACCCGGAGCCGTAGCGAAAGCGAGTC 660
H21O-S3         CCTGCGAGTTGTGATGTCTGGCGAGGTTAAGGAAACCCGGAGCCGTAGCGAAAGCGAGTC 660
P3UCO1          CCTGCGAGTTGTGATGTCTGGCGAGGTTAAGGAAACCCGGAGCCGTAGCGAAAGCGAGTC 659
P3UCB1          CCTGCGAGTTGTGATGTCTGGCGAGGTTAAGGAAACCCGGAGCCGTAGCGAAAGCGAGTC 659
P9CDO-S1        CCTGCGAGTTGTGATGTCTGGCAAGGTTAAGGAAACCCGGAGCCGTAGCGAAAGCGAGTC 656
P20CDO-S4       CCTGCGAGTTGTGATGTCTGGCAAGGTTAAGGAAACCCGGAGCCGTAGCGAAAGCAAGTC 659
13826           CCTGCGAGTTGTGATGTCTGGCGAGGTTAAGGAAACCCGGAGCCGTAGCGAAAGCGAGTC 660
ATCC51561       CCTGCGAGTTGTGATGTCTGGCGAGGTTAAGGAAACCCGGAGCCGTAGCGAAAGCGAGTC 657
H3O1            CCTGCGAGTTGTGATGTCTGGCGAGGTTAAGGAAACCCGGAGCCGTAGCGAAAGCGAGTC 657
H7O-S1          CCTGCGAGTTGTGATGTCTGGCAAGGTTAAGGAAACCCGGAGCCGTAGCGAAAGCGAGTC 657
H9O-S1          CCTGCGAGTTGTGATGTCTGGCGAGGTTAAGGAAACCCGGAGCCGTAGCGAAAGCGAGTC 657
H9O-S2          CCTGCGAGTTGTGATGTCTGGCGAGGTTAAGGAAACCCGGAGCCGTAGCGAAAGCGAGTC 660
H11O-S1         CCTGCGAGTTGTGATGTCTGGCGAGGTTAAGGAAACCCGGAGCCGTAGCGAAAGCGAGTC 657
H14O-S1         CCTGCGAGTTGTGATGTCTGGCGAGGTTAAGGAAACCCGGAGCCGTAGCGAAAGCGAGTC 659
H16O-S1         CCTGCGAGTTGTGATGTCTGGCGAGGTTAAGGAAACCCGGAGCCGTAGCGAAAGCGAGTC 657
H20O-S1         CCTGCGAGTTGTGATGTCTGGCAAGGTTAAGGAAACCCGGAGCCGTAGCGAAAGCGAGTC 657
H21O-S1         CCTGCGAGTTGTGATGTCTGGCGAGGTTAAGGAAACCCGGAGCCGTAGCGAAAGCGAGTC 660
H21O-S2         CCTGCGAGTTGTGATGTCTGGCGAGGTTAAGGAAACCCGGAGCCGTAGCGAAAGCGAGTC 660
H21O-S5         CCTGCGAGTTGTGATGTCTGGCGAGGTTAAGGAAACCCGGAGCCGTAGCGAAAGCGAGTT 660
H22O-S1         CCTGCGAGTTGTGATGTCTGGCGAGGTTAAGGAAACCCGGAGCCGTAGCGAAAGCGAGTC 660
H23O-S1         CCTGCGAGTTGTGATGTCTGGCGAGGTTAAGGAAACCCGGAGCCGTAGCGAAAGCGAGTC 660
P2CDO3          CCTGCGAGTTGTGATGTCTGGCGAGGTTAAGGAAACCCGGAGCCGTAGCGAAAGCGAGTC 660
P2CDO4          CCTGCGAGTTGTGATGTCTGGCGAGGTTAAGGAAACCCGGAGCCGTAGCGAAAGCGAGTC 660
P2CDO-S6        CCTGCGAGTTGTGATGTCTGGCGAGGTTAAGGAAACCCGGAGCCGTAGCGAAAGCGAGTC 660
P6CDO1          CCTGCGAGTTGTGATGTCTGGCGAGGTTAAGGAAACCCGGAGCCGTAGCGAAAGCGAGTC 657
P12CDO-S1       CCTGCGAGTTGTGATGTCTGGCGAGGTTAAGGAAACCCGGAGCCGTAGCGAAAGCGAGTC 657
P13UCO-S3       CCTGCGAGTTGTGATGTCTGGCGAGGTTAAGGAAACCCGGAGCCGTAGCGAAAGCGAGTC 660
P15UCO-S2       CCTGCGAGTTGTGATGTCTGGCGAGGTTAAGGAAACCCGGAGCCGTAGCGAAAGCGAGTC 660
P16UCO-S1       CCTGCGAGTTGTGATGTCTGGCGAGGTTAAGGAAACCCGGAGCCGTAGCGAAAGCGAGTC 657
P16UCO-S2       CCTGCGAGTTGTGATGTCTGGCGAGGTTAAGGAAACCCGGAGCCGTAGCGAAAGCGAGTC 657
P18CDO-S1       CCTGCGAGTTGTGATGTCTGGCGAGGTTAAGGAAACCCGGAGCCGTAGCGAAAGCGAGTC 657
P20CDO-S1       CCTGCGAGTTGTGATGTCTGGCGAGGTTAAGGAAACCCGGAGCCGTAGCGAAAGCGAGTC 660
P20CDO-S2       CCTGCGAGTTGTGATGTCTGGCGAGGTTAAGGAAACCCGGAGCCGTAGCGAAAGCGAGTC 660
P20CDO-S3       CCTGCGAGTTGTGATGTCTGGCGAGGTTAAGGAAACCCGGAGCCGTAGCGAAAGCGAGTC 660
P21CDO-S1       CCTGCGAGTTGTGATGTCTGGCGAGGTTAAGGAAACCCGGAGCCGTAGCGAAAGCGAGTC 659
P21CDO-S2       CCTGCGAGTTGTGATGTCTGGCAAGGTTAAGGAAACCCGGAGCCGTAGCGAAAGCGAGTC 660
P21CDO-S4       CCTGCGAGTTGTGATGTCTGGCAAGGTTAAGGAAACCCGGAGCCGTAGCGAAAGCGAGTC 660
P24CDO-S2       CCTGCGAGTTGTGATGTCTGGCGAGGTTAAGGAAACCCGGAGCCGTAGCGAAAGCGAGTC 660
P24CDO-S3       CCTGCGAGTTGTGATGTCTGGCGAGGTTAAGGAAACCCGGAGCCGTAGCGAAAGCGAGTC 660
P24CDO-S4       CCTGCGAGTTGTGATGTCTGGCGAGGTTAAGGAAACCCGGAGCCGTAGCGAAAGCGAGTC 660
UNSW1           CCTGCGAGTTGTGATGTCTGGCGAGGTTAAGGAAACCCGGAGCCGTAGCGAAAGCGAGTC 660
UNSW2           CCTGCGAGTTGTGATGTCTGGCGAGGTTAAGGAAACCCGGAGCCGTAGCGAAAGCGAGTC 660
UNSW3           CCTGCGAGTTGTGATGTCTGGCAAGGTTAAGGAAACCCGGAGCCGTAGCGAAAGCGAGTC 660
UNSWCD          CCTGCGAGTTGTGATGTCTGGCAAGGTTAAGGAAACCCGGAGCCGTAGCGAAAGCGAGTC 657
UNSWCS          CCTGCGAGTTGTGATGTCTGGCGAGGTTAAGGAAACCCGGAGCCGTAGCGAAAGCGAGTC 660
                ********************** ************* ******* ********** ***
```

Supplementary Figure S2

```
ATCC33237       TTAATAGGGCGTTTAGTCAGACGTTGCAGACCCGAAACGATGTGATCTATCCATGAGCAG 719
ATCC51562       TTAATAGGGCGTTTAGTCAGACGTTGCAGACCCGAAACGATGTGATCTATCCATGAGCAG 716
H1O1            TTAATAGGGCGTTTAGTCAGACGTTGCAGACCCGAAACGATGTGATCTATCCATGAGCAG 720
H10O-S1         TTAATAGGGCGTTTAGTCAGACGTTGCAGACCCGAAACGATGTGATCTATCCATGAGCAG 716
H17O-S1         TTAATAGGGCGTTTAGTCAGACGTTGCAGACCCGAAACGATGTGATCTATCCATGAGCAG 720
H21O-S3         TTAATAGGGCGTTTAGTCAGACGTTGCAGACCCGAAACGATGTGATCTATCCATGAGCAG 720
P3UCO1          TTAATAGGGCGTTTAGTCAGACGTTGCAGACCCGAAACGATGTGATCTATCCATGAGCAG 719
P3UCB1          TTAATAGGGCGTTTAGTCAGACGTTGCAGACCCGAAACGATGTGATCTATCCATGAGCAG 719
P9CDO-S1        TTAATAGGGCGTTTAGTCAGACGTTGCAGACCCGAAACGATGTGATCTATCCATGAGCAG 716
P20CDO-S4       TTAATAGGGCGTTTAGTCAGACGTTGCAGACCCGAAACGATGTGATCTATCCATGAGCAG 719
13826           TTAATAGGGCGTTTAGTCAGACGTTGCAGACCCGAAACGATGTGATCTATCCATGAGCAG 720
ATCC51561       TTAATAGGGCGTTTAGTCAGACGTTGCAGACCCGAAACGATGTGATCTATCCATGAGCAG 717
H3O1            TTAATAGGGCGTTTAGTCAGACGTTGCAGACCCGAAACGATGTGATCTATCCATGAGCAG 717
H7O-S1          TTAATAGGGCGTTTAGTCAGACGTTGCAGACCCGAAACGATGTGATCTATCCATGAGCAG 717
H9O-S1          TTAATAGGGCGTTTAGTCAGACGTTGCAGACCCGAAACGATGTGATCTATCCATGAGCAG 717
H9O-S2          TTAATAGGGCGTTTAGTCAGACGTTGCAGACCCGAAACGATGTGATCTATCCATGAGCAG 720
H11O-S1         TTAATAGGGCGTTTAGTCAGACGTTGCAGACCCGAAACGATGTGATCTATCCATGAGCAG 717
H14O-S1         TTAATAGGGCATTTAGTCAGACGTTGCAGACCCGAAACGATGTGATCTATCCATGAGCAG 719
H16O-S1         TTAATAGGGCGTTTAGTCAGACGTTGCAGACCCGAAACGATGTGATCTATCCATGAGCAG 717
H20O-S1         TTAATAGGGCGTTTAGTCAGACGTTGCAGACCCGAAACGATGTGATCTATCCATGAGCAG 717
H21O-S1         TTAATAGGGCGTTTAGTCAGACGTTGCAGACCCGAAACGATGTGATCTATCCATGAGCAG 720
H21O-S2         TTAATAGGGCGTTTAGTCAGACGTTGCAGACCCGAAACGATGTGATCTATCCATGAGCAG 720
H21O-S5         TTAATAGGGCGTTTAGTCAGACGTTGCAGACCCGAAACGATGTGATCTATCCATGAGCAG 720
H22O-S1         TTAATAGGGCGTTTAGTCAGACGTTGCAGACCCGAAACGATGTGATCTATCCATGAGCAG 720
H23O-S1         TTAATAGGGCGTTTAGTCAGACGTTGCAGACCCGAAACGATGTGATCTATCCATGAGCAG 720
P2CDO3          TTAATAGGGCGTTTAGTCAGACGTTGCAGACCCGAAACGATGTGATCTATCCATGAGCAG 720
P2CDO4          TTAATAGGGCGTTTAGTCAGACGTTGCAGACCCGAAACGATGTGATCTATCCATGAGCAG 720
P2CDO-S6        TTAATAGGGCGTTTAGTCAGACGTTGCAGACCCGAAACGATGTGATCTATCCATGAGCAG 720
P6CDO1          TTAATAGGGCGTTTAGTCAGACGTTGCAGACCCGAAACGATGTGATCTATCCATGAGCAG 717
P12CDO-S1       TTAATAGGGCGTTTAGTCAGACGTTGCAGACCCGAAACGATGTGATCTATCCATGAGCAG 717
P13UCO-S3       TTAATAGGGCGTTTAGTCAGACGTTGCAGACCCGAAACGATGTGATCTATCCATGAGCAG 720
P15UCO-S2       TTAATAGGGCATTTAGTCAGACGTTGCAGACCCGAAACGATGTGATCTATCCATGAGCAG 720
P16UCO-S1       TTAATAGGGCATTTAGTCAGACGTTGCAGACCCGAAACGATGTGATCTATCCATGAGCAG 717
P16UCO-S2       TTAATAGGGCGTTTAGTCAGACGTTGCAGACCCGAAACGATGTGATCTATCCATGAGCAG 717
P18CDO-S1       TTAATAGGGCGTTTAGTCAGACGTTGCAGACCCGAAACGATGTGATCTATCCATGAGCAG 717
P20CDO-S1       TTAATAGGGCGTTTAGTCAGACGTTGCAGACCCGAAACGATGTGATCTATCCATGAGCAG 720
P20CDO-S2       TTAATAGGGCGTTTAGTCAGACGTTGCAGACCCGAAACGATGTGATCTATCCATGAGCAG 720
P20CDO-S3       TTAATAGGGCGTTTAGTCAGACGTTGCAGACCCGAAACGATGTGATCTATCCATGAGCAG 720
P21CDO-S1       TTAATAGGGCATTTAGTCAGACGTTGCAGACCCGAAACGATGTGATCTATCCATGAGCAG 719
P21CDO-S2       TTAATAGGGCGTTTAGTCAGACGTTGCAGACCCGAAACGATGTGATCTATCCATGAGCAG 720
P21CDO-S4       TTAATAGGGCGTTTAGTCAGACGTTGCAGACCCGAAACGATGTGATCTATCCATGAGCAG 720
P24CDO-S2       TTAATAGGGCGTTTAGTCAGACGTTGCAGACCCGAAACGATGTGATCTATCCATGAGCAG 720
P24CDO-S3       TTAATAGGGCGTTTAGTCAGACGTTGCAGACCCGAAACGATGTGATCTATCCATGAGCAG 720
P24CDO-S4       TTAATAGGGCGTTTAGTCAGACGTTGCAGACCCGAAACGATGTGATCTATCCATGAGCAG 720
UNSW1           TTAATAGGGCGTTTAGTCAGACGTTGCAGACCCGAAACGATGTGATCTATCCATGAGCAG 720
UNSW2           TTAATAGGGCGTTTAGTCAGACGTTGCAGACCCGAAACGATGTGATCTATCCATGAGCAG 720
UNSW3           TTAATAGGGCGTTTAGTCAGACGTTGCAGACCCGAAACGATGTGATCTATCCATGAGCAG 720
UNSWCD          TTAATAGGGCGTTTAGTCAGACGTTGCAGACCCGAAACGATGTGATCTATCCATGAGCAG 717
UNSWCS          TTAATAGGGCGTTTAGTCAGACGTTGCAGACCCGAAACGATGTGATCTATCCATGAGCAG 720
                ********** *************************************************
```

Supplementary Figure S2

```
ATCC33237       GTTGAAACCGGTGTAAGAGCCGGTGGAGGACCGAACCCGCTAGCGTTGAAAAGCTATGGG 779
ATCC51562       GTTGAAACCGGTGTAAGAGCCGGTGGAGGACCGAACCCGCTAGCGTTGAAAAGCTATGGG 776
H1O1            GTTGAAACCGGTGTAAGAGCCGGTGGAGGACCGAACCCGCTAGCGTTGAAAAGCTATGGG 780
H10O-S1         GTTGAAACCGGTGTAAGAGCCGGTGGAGGACCGAACCCGCTAGCGTTGAAAAGCTATGGG 776
H17O-S1         GTTGAAACCGGTGTAAGAGCCGGTGGAGGACCGAACCCGCTAGCGTTGAAAAGCTATGGG 780
H21O-S3         GTTGAAACCGGTGTAAGAGCCGGTGGAGGACCGAACCCGCTAGCGTTGAAAAGCTATGGG 780
P3UCO1          GTTGAAACCGGTGTAAGAGCCGGTGGAGGACCGAACCCGCTAGCGTTGAAAAGCTATGGG 779
P3UCB1          GTTGAAACCGGTGTAAGAGCCGGTGGAGGACCGAACCCGCTAGCGTTGAAAAGCTATGGG 779
P9CDO-S1        GTTGAAACCGGTGTAAGAGCCGGTGGAGGACCGAACCCGCTAGCGTTGAAAAGCTATGGG 776
P20CDO-S4       GTTGAAACCGGTGTAAGAGCCGGTGGAGGACCGAACCCGCTAGCGTTGAAAAGCTATGGG 779
13826           GTTGAAACCGGTGTAAGAGCCGGTGGAGGACCGAACCCGCTAGCGTTGAAAAGCTATGGG 780
ATCC51561       GTTGAAACCGGTGTAAGAGCCGGTGGAGGACCGAACCCGCTAGCGTTGAAAAGCTATGGG 777
H3O1            GTTGAAACCGGTGTAAGAGCCGGTGGAGGACCGAACCCGCTAGCGTTGAAAAGCTATGGG 777
H7O-S1          GTTGAAACCGGTGTAAGAGCCGGTGGAGGACCGAACCCGCTAGCGTTGAAAAGCTATGGG 777
H9O-S1          GTTGAAACCGGTGTAAGAGCCGGTGGAGGACCGAACCCGCTAGCGTTGAAAAGCTATGGG 777
H9O-S2          GTTGAAACCGGTGTAAGAGCCGGTGGAGGACCGAACCCGCTAGCGTTGAAAAGCTATGGG 780
H11O-S1         GTTGAAACCGGTGTAAGAGCCGGTGGAGGACCGAACCCGCTAGCGTTGAAAAGCTATGGG 777
H14O-S1         GTTGAAACCGGTGTAAGAGCCGGTGGAGGACCGAACCCGCTAGCGTTGAAAAGCTATGGG 779
H16O-S1         GTTGAAACCGGTGTAAGAGCCGGTGGAGGACCGAACCCGCTAGCGTTGAAAAGCTATGGG 777
H20O-S1         GTTGAAACCGGTGTAAGAGCCGGTGGAGGACCGAACCCGCTAGCGTTGAAAAGCTATGGG 777
H21O-S1         GTTGAAACCGGTGTAAGAGCCGGTGGAGGACCGAACCCGCTAGCGTTGAAAAGCTATGGG 780
H21O-S2         GTTGAAACCGGTGTAAGAGCCGGTGGAGGACCGAACCCGCTAGCGTTGAAAAGCTATGGG 780
H21O-S5         GTTGAAACCGGTGTAAGAGCCGGTGGAGGACCGAACCCGCTAGCGTTGAAAAGCTATGGG 780
H22O-S1         GTTGAAACCGGTGTAAGAGCCGGTGGAGGACCGAACCCGCTAGCGTTGAAAAGCTATGGG 780
H23O-S1         GTTGAAACCGGTGTAAGAGCCGGTGGAGGACCGAACCCGCTAGCGTTGAAAAGCTATGGG 780
P2CDO3          GTTGAAACCGGTGTAAGAACCGGCGGAGGACCGAACCCGCTAGCGTTGAAAAGCTATGGG 780
P2CDO4          GTTGAAACCGGTGTAAGAGCCGGTGGAGGACCGAACCCGCTAGCGTTGAAAAGCTATGGG 780
P2CDO-S6        GTTGAAACCGGTGTAAGAACCGGCGGAGGACCGAACCCGCTAGCGTTGAAAAGCTATGGG 780
P6CDO1          GTTGAAACCGGTGTAAGAGCCGGTGGAGGACCGAACCCGCTAGCGTTGAAAAGCTATGGG 777
P12CDO-S1       GTTGAAACCGGTGTAAGAGCCGGTGGAGGACCGAACCCGCTAGCGTTGAAAAGCTATGGG 777
P13UCO-S3       GTTGAAACCGGTGTAAGAGCCGGTGGAGGACCGAACCCGCTAGCGTTGAAAAGCTATGGG 780
P15UCO-S2       GTTGAAACCGGTGTAAGAGCCGGTGGAGGACCGAACCCGCTAGCGTTGAAAAGCTATGGG 780
P16UCO-S1       GTTGAAACCGGTGTAAGAGCCGGTGGAGGACCGAACCCGCTAGCGTTGAAAAGCTATGGG 777
P16UCO-S2       GTTGAAACCGGTGTAAGAGCCGGTGGAGGACCGAACCCGCTAGCGTTGAAAAGCTATGGG 777
P18CDO-S1       GTTGAAACCGGTGTAAGAGCCGGTGGAGGACCGAACCCGCTAGCGTTGAAAAGCTATGGG 777
P20CDO-S1       GTTGAAACCGGTGTAAGAGCCGGTGGAGGACCGAACCCGCTAGCGTTGAAAAGCTATGGG 780
P20CDO-S2       GTTGAAACCGGTGTAAGAGCCGGTGGAGGACCGAACCCGCTAGCGTTGAAAAGCTATGGG 780
P20CDO-S3       GTTGAAACCGGTGTAAGAGCCGGTGGAGGACCGAACCCGCTAGCGTTGAAAAGCTATGGG 780
P21CDO-S1       GTTGAAACCGGTGTAAGAGCCGGTGGAGGACCGAACCCGCTAGCGTTGAAAAGCTATGGG 779
P21CDO-S2       GTTGAAACCGGTGTAAGAGCCGGTGGAGGACCGAACCCGCTAGCGTTGAAAAGCTATGGG 780
P21CDO-S4       GTTGAAACCGGTGTAAGAGCCGGTGGAGGACCGAACCCGCTAGCGTTGAAAAGCTATGGG 780
P24CDO-S2       GTTGAAACCGGTGTAAGAGCCGGTGGAGGACCGAACCCGCTAGCGTTGAAAAGCTATGGG 780
P24CDO-S3       GTTGAAACCGGTGTAAGAGCCGGTGGAGGACCGAACCCGCTAGCGTTGAAAAGCTATGGG 780
P24CDO-S4       GTTGAAACCGGTGTAAGAGCCGGTGGAGGACCGAACCCGCTAGCGTTGAAAAGCTATGGG 780
UNSW1           GTTGAAACCGGTGTAAGAGCCGGTGGAGGACCGAACCCGCTAGCGTTGAAAAGCTATGGG 780
UNSW2           GTTGAAACCGGTGTAAGAGCCGGTGGAGGACCGAACCCGCTAGCGTTGAAAAGCTATGGG 780
UNSW3           GTTGAAACCGGTGTAAGAGCCGGTGGAGGACCGAACCCGCTAGCGTTGAAAAGCTATGGG 780
UNSWCD          GTTGAAACCGGTGTAAGAGCCGGTGGAGGACCGAACCCGCTAGCGTTGAAAAGCTATGGG 777
UNSWCS          GTTGAAACCGGTGTAAGAGCCGGTGGAGGACCGAACCCGCTAGCGTTGAAAAGCTATGGG 780
                ****************** **** ************************************
```

Supplementary Figure S2

ATCC33237	ATGACTTGTGGATAGGGGTGAAAGGCCAATCAAACATCGTGATAGCTGGTTCTCTCCGAA	839
ATCC51562	ATGACTTGTGGATAGGGGTGAAAGGCCAATCAAACATCGTGATAGCTGGTTCTCTCCGAA	836
H1O1	ATGACTTGTGGATAGGGGTGAAAGGCCAATCAAACATCGTGATAGCTGGTTCTCTCCGAA	840
H10O-S1	ATGACTTGTGGATAGGGGTGAAAGGCCAATCAAACATCGTGATAGCTGGTTCTCTCCGAA	836
H17O-S1	ATGACTTGTGGATAGGGGTGAAAGGCCAATCAAACATCGTGATAGCTGGTTCTCTCCGAA	840
H21O-S3	ATGACTTGTGGATAGGGGTGAAAGGCCAATCAAACATCGTGATAGCTGGTTCTCTCCGAA	840
P3UCO1	ATGACTTGTGGATAGGGGTGAAAGGCCAATCAAACATCGTGATAGCTGGTTCTCTCCGAA	839
P3UCB1	ATGACTTGTGGATAGGGGTGAAAGGCCAATCAAACATCGTGATAGCTGGTTCTCTCCGAA	839
P9CDO-S1	ATGACTTGTGGATAGGGGTGAAAGGCCAATCAAACATCGTGATAGCTGGTTCTCTCCGAA	836
P20CDO-S4	ATGACTTGTGGATAGGGGTGAAAGGCCAATCAAACATCGTGATAGCTGGTTCTCTCCGAA	839
13826	ATGACTTGTGGATAGGGGTGAAAGGCCAATCAAACATCGTGATAGCTGGTTCTCTCCGAA	840
ATCC51561	ATGACTTGTGGATAGGGGTGAAAGGCCAATCAAACATCGTGATAGCTGGTTCTCTCCGAA	837
H3O1	ATGACTTGTGGATAGGGGTGAAAGGCCAATCAAACATCGTGATAGCTGGTTCTCTCCGAA	837
H7O-S1	ATGACTTGTGGATAGGGGTGAAAGGCCAATCAAACATCGTGATAGCTGGTTCTCTCCGAA	837
H9O-S1	ATGACTTGTGGATAGGGGTGAAAGGCCAATCAAACATCGTGATAGCTGGTTCTCTCCGAA	837
H9O-S2	ATGACTTGTGGATAGGGGTGAAAGGCCAATCAAACATCGTGATAGCTGGTTCTCTCCGAA	840
H11O-S1	ATGACTTGTGGATAGGGGTGAAAGGCCAATCAAACATCGTGATAGCTGGTTCTCTCCGAA	837
H14O-S1	ATGACTTGTGGATAGGGGTGAAAGGCCAATCAAACATCGTGATAGCTGGTTCTCTCCGAA	839
H16O-S1	ATGACTTGTGGATAGGGGTGAAAGGCCAATCAAACATCGTGATAGCTGGTTCTCTCCGAA	837
H20O-S1	ATGACTTGTGGATAGGGGTGAAAGGCCAATCAAACATCGTGATAGCTGGTTCTCTCCGAA	837
H21O-S1	ATGACTTGTGGATAGGGGTGAAAGGCCAATCAAACATCGTGATAGCTGGTTCTCTCCGAA	840
H21O-S2	ATGACTTGTGGATAGGGGTGAAAGGCCAATCAAACATCGTGATAGCTGGTTCTCTCCGAA	840
H21O-S5	ATGACTTGTGGATAGGGGTGAAAGGCCAATCAAACATCGTGATAGCTGGTTCTCTCCGAA	840
H22O-S1	ATGACTTGTGGATAGGGGTGAAAGGCCAATCAAACATCGTGATAGCTGGTTCTCTCCGAA	840
H23O-S1	ATGACTTGTGGATAGGGGTGAAAGGCCAATCAAACATCGTGATAGCTGGTTCTCTCCGAA	840
P2CDO3	ATGACTTGTGGATAGGGGTGAAAGGCCAATCAAACATCGTGATAGCTGGTTCTCTCCGAA	840
P2CDO4	ATGACTTGTGGATAGGGGTGAAAGGCCAATCAAACATCGTGATAGCTGGTTCTCTCCGAA	840
P2CDO-S6	ATGACTTGTGGATAGGGGTGAAAGGCCAATCAAACATCGTGATAGCTGGTTCTCTCCGAA	840
P6CDO1	ATGACTTGTGGATAGGGGTGAAAGGCCAATCAAACATCGTGATAGCTGGTTCTCTCCGAA	837
P12CDO-S1	ATGACTTGTGGATAGGGGTGAAAGGCCAATCAAACATCGTGATAGCTGGTTCTCTCCGAA	837
P13UCO-S3	ATGACTTGTGGATAGGGGTGAAAGGCCAATCAAACATCGTGATAGCTGGTTCTCTCCGAA	840
P15UCO-S2	ATGACTTGTGGATAGGGGTGAAAGGCCAATCAAACATCGTGATAGCTGGTTCTCTCCGAA	840
P16UCO-S1	ATGACTTGTGGATAGGGGTGAAAGGCCAATCAAACATCGTGATAGCTGGTTCTCTCCGAA	837
P16UCO-S2	ATGACTTGTGGATAGGGGTGAAAGGCCAATCAAACATCGTGATAGCTGGTTCTCTCCGAA	837
P18CDO-S1	ATGACTTGTGGATAGGGGTGAAAGGCCAATCAAACATCGTGATAGCTGGTTCTCTCCGAA	837
P20CDO-S1	ATGACTTGTGGATAGGGGTGAAAGGCCAATCAAACATCGTGATAGCTGGTTCTCTCCGAA	840
P20CDO-S2	ATGACTTGTGGATAGGGGTGAAAGGCCAATCAAACATCGTGATAGCTGGTTCTCTCCGAA	840
P20CDO-S3	ATGACTTGTGGATAGGGGTGAAAGGCCAATCAAACATCGTGATAGCTGGTTCTCTCCGAA	840
P21CDO-S1	ATGACTTGTGGATAGGGGTGAAAGGCCAATCAAACATCGTGATAGCTGGTTCTCTCCGAA	839
P21CDO-S2	ATGACTTGTGGATAGGGGTGAAAGGCCAATCAAACATCGTGATAGCTGGTTCTCTCCGAA	840
P21CDO-S4	ATGACTTGTGGATAGGGGTGAAAGGCCAATCAAACATCGTGATAGCTGGTTCTCTCCGAA	840
P24CDO-S2	ATGACTTGTGGATAGGGGTGAAAGGCCAATCAAACATCGTGATAGCTGGTTCTCTCCGAA	840
P24CDO-S3	ATGACTTGTGGATAGGGGTGAAAGGCCAATCAAACATCGTGATAGCTGGTTCTCTCCGAA	840
P24CDO-S4	ATGACTTGTGGATAGGGGTGAAAGGCCAATCAAACATCGTGATAGCTGGTTCTCTCCGAA	840
UNSW1	ATGACTTGTGGATAGGGGTGAAAGGCCAATCAAACATCGTGATAGCTGGTTCTCTCCGAA	840
UNSW2	ATGACTTGTGGATAGGGGTGAAAGGCCAATCAAACATCGTGATAGCTGGTTCTCTCCGAA	840
UNSW3	ATGACTTGTGGATAGGGGTGAAAGGCCAATCAAACATCGTGATAGCTGGTTCTCTCCGAA	840
UNSWCD	ATGACTTGTGGATAGGGGTGAAAGGCCAATCAAACATCGTGATAGCTGGTTCTCTCCGAA	837
UNSWCS	ATGACTTGTGGATAGGGGTGAAAGGCCAATCAAACATCGTGATAGCTGGTTCTCTCCGAA	840
	**	

Supplementary Figure S2

```
ATCC33237      ATATATTTAGGTATAGCGTCATGTAGTAACACTGGGGGGTAGAGCACTGAATGGGCTAGG 899
ATCC51562      ATATATTTAGGTATAGCGTCATGTAGTAACACTGGGGGGTAGAGCACTGAATGGGCTAGG 896
H1O1           ATATATTTAGGTATAGCGTCATGTAGTAACACTAGGGGGTAGAGCACTGAATGGGCTAGG 900
H10O-S1        ATATATTTAGGTATAGCGTCATGTAGTAACACTAGGGGGTAGAGCACTGAATGGGCTAGG 896
H17O-S1        ATATATTTAGGTATAGCGTCATGTAGTAACACTAGGGGGTAGAGCACTGAATGGGCTAGG 900
H21O-S3        ATATATTTAGGTATAGCGTCATGTAGTAACACTAGGGGGTAGAGCACTGAATGGGCTAGG 900
P3UCO1         ATATATTTAGGTATAGCGTCATGTAGTAACACTAGGGGGTAGAGCACTGAATGGGCTAGG 899
P3UCB1         ATATATTTAGGTATAGCGTCATGTAGTAACACTAGGGGGTAGAGCACTGAATGGGCTAGG 899
P9CDO-S1       ATATATTTAGGTATAGCGTCATGTAGTAACACTAGGGGGTAGAGCACTGAATGGGCTAGG 896
P20CDO-S4      ATATATTTAGGTATAGCGTCATGTAGTAACACTAGGGGGTAGAGCACTGAATGGGCTAGG 899
13826          ATATATTTAGGTATAGCGTCATGTAGTAACACTAGGGGGTAGAGCACTGAATGGGCTAGG 900
ATCC51561      ATATATTTAGGTATAGCGTCATGTAGTAACACTAGGGGGTAGAGCACTGAATGGGCTAGG 897
H3O1           ATATATTTAGGTATAGCGTCATGTAGTAACACTAGGGGGTAGAGCACTGAATGGGCTAGG 897
H7O-S1         ATATATTTAGGTATAGCGTCATGTAGTAACACTAGGGGGTAGAGCACTGAATGGGCTAGG 897
H9O-S1         ATATATTTAGGTATAGCGTCATGTAGTAACACTAGGGGGTAGAGCACTGAATGGGCTAGG 897
H9O-S2         ATATATTTAGGTATAGCGTCATGTAGTAACACTAGGGGGTAGAGCACTGAATGGGCTAGG 900
H11O-S1        ATATATTTAGGTATAGCGTCATGTAGTAACACTAGGGGGTAGAGCACTGAATGGGCTAGG 897
H14O-S1        ATATATTTAGGTATAGCGTCATGTAGTAACACTAGGGGGTAGAGCACTGAATGGGCTAGG 899
H16O-S1        ATATATTTAGGTATAGCGTCATGTAGTAACACTAGGGGGTAGAGCACTGAATGGGCTAGG 897
H20O-S1        ATATATTTAGGTATAGCGTCATGTAGTAACACTAGGGGGTAGAGCACTGAATGGGCTAGG 897
H21O-S1        ATATATTTAGGTATAGCGTCATGTAGTAACACTAGGGGGTAGAGCACTGAATGGGCTAGG 900
H21O-S2        ATATATTTAGGTATAGCGTCATGTAGTAACACTAGGGGGTAGAGCACTGAATGGGCTAGG 900
H21O-S5        ATATATTTAGGTATAGCGTCATGTAGTAACACTAGGGGGTAGAGCACTGAATGGGCTAGG 900
H22O-S1        ATATATTTAGGTATAGCGTCATGTAGTAACACTAGGGGGTAGAGCACTGAATGGGCTAGG 900
H23O-S1        ATATATTTAGGTATAGCGTCATGTAGTAACACTAGGGGGTAGAGCACTGAATGGGCTAGG 900
P2CDO3         ATATATTTAGGTATAGCGTCATGTAGTAACACTAGGGGGTAGAGCACTGAATGGGCTAGG 900
P2CDO4         ATATATTTAGGTATAGCGTCATGTAGTAACACTAGGGGGTAGAGCACTGAATGGGCTAGG 900
P2CDO-S6       ATATATTTAGGTATAGCGTCATGTAGTAACACTAGGGGGTAGAGCACTGAATGGGCTAGG 900
P6CDO1         ATATATTTAGGTATAGCGTCATGTAGTAACACTAGGGGGTAGAGCACTGAATGGGCTAGG 897
P12CDO-S1      ATATATTTAGGTATAGCGTCATGTAGTAACACTAGGGGGTAGAGCACTGAATGGGCTAGG 897
P13UCO-S3      ATATATTTAGGTATAGCGTCATGTAGTAACACTAGGGGGTAGAGCACTGAATGGGCTAGG 900
P15UCO-S2      ATATATTTAGGTATAGCGTCATGTAGTAACACTAGGGGGTAGAGCACTGAATGGGCTAGG 900
P16UCO-S1      ATATATTTAGGTATAGCGTCATGTAGTAACACTAGGGGGTAGAGCACTGAATGGGCTAGG 897
P16UCO-S2      ATATATTTAGGTATAGCGTCATGTAGTAACACTAGGGGGTAGAGCACTGAATGGGCTAGG 897
P18CDO-S1      ATATATTTAGGTATAGCGTCATGTAGTAACACTAGGGGGTAGAGCACTGAATGGGCTAGG 897
P20CDO-S1      ATATATTTAGGTATAGCGTCATGTAGTAACACTAGGGGGTAGAGCACTGAATGGGCTAGG 900
P20CDO-S2      ATATATTTAGGTATAGCGTCATGTAGTAACACTAGGGGGTAGAGCACTGAATGGGCTAGG 900
P20CDO-S3      ATATATTTAGGTATAGCGTCATGTAGTAACACTAGGGGGTAGAGCACTGAATGGGCTAGG 900
P21CDO-S1      ATATATTTAGGTATAGCGTCATGTAGTAACACTAGGGGGTAGAGCACTGAATGGGCTAGG 899
P21CDO-S2      ATATATTTAGGTATAGCGTCATGTAGTAACACTAGGGGGTAGAGCACTGAATGGGCTAGG 900
P21CDO-S4      ATATATTTAGGTATAGCGTCATGTAGTAACACTAGGGGGTAGAGCACTGAATGGGCTAGG 900
P24CDO-S2      ATATATTTAGGTATAGCGTCATGTAGTAACACTAGGGGGTAGAGCACTGAATGGGCTAGG 900
P24CDO-S3      ATATATTTAGGTATAGCGTCATGTAGTAACACTAGGGGGTAGAGCACTGAATGGGCTAGG 900
P24CDO-S4      ATATATTTAGGTATAGCGTCATGTAGTAACACTAGGGGGTAGAGCACTGAATGGGCTAGG 900
UNSW1          ATATATTTAGGTATAGCGTCATGTAGTAACACTAGGGGGTAGAGCACTGAATGGGCTAGG 900
UNSW2          ATATATTTAGGTATAGCGTCATGTAGTAACACTAGGGGGTAGAGCACTGAATGGGCTAGG 900
UNSW3          ATATATTTAGGTATAGCGTCATGTAGTAACACTAGGGGGTAGAGCACTGAATGGGCTAGG 900
UNSWCD         ATATATTTAGGTATAGCGTCATGTAGTAACACTAGGGGGTAGAGCACTGAATGGGCTAGG 897
UNSWCS         ATATATTTAGGTATAGCGTCATGTAGTAACACTAGGGGGTAGAGCACTGAATGGGCTAGG 900
               ********************************* **************************
```

Supplementary Figure S2

```
ATCC33237         GCATACACCAATGTACCAAACCCTATCAAACTCCGAATACCTAGTGTGTAATCATGGCAG 959
ATCC51562         GCATACACCAATGTACCAAACCCTATCAAACTCCGAATACCTAGTGTGTAATCATGGCAG 956
H1O1              GCATACACCAATGTACCAAACCCTATCAAACTCCGAATACCTAGTGTGTAATCATGGCAG 960
H10O-S1           GCATACACCAATGTACCAAACCCTATCAAACTCCGAATACCTAGTGTGTAATCATGGCAG 956
H17O-S1           GCATACACCAATGTACCAAACCCTATCAAACTCCGAATACCTAGTGTGTAATCATGGCAG 960
H21O-S3           GCATACACCAATGTACCAAACCCTATCAAACTCCGAATACCTAGTGTGTAATCATGGCAG 960
P3UCO1            GCATACACCAATGTACCAAACCCTATCAAACTCCGAATACCTAGTGTGTAATCATGGCAG 959
P3UCB1            GCATACACCAATGTACCAAACCCTATCAAACTCCGAATACCTAGTGTGTAATCATGGCAG 959
P9CDO-S1          GCATACACCAATGTACCAAACCCTATCAAACTCCGAATACCTAGTGTGTAATCATGGCAG 956
P20CDO-S4         GCATACACCAATGTACCAAACCCTATCAAACTCCGAATACCTAGTGTGTAATCATGGCAG 959
13826             GCATACACCAATGTACCAAACCCTATCAAACTCCGAATACCTAGTGTGTAATCATGGCAG 960
ATCC51561         GCATACACCAATGTACCAAACCCTATCAAACTCCGAATACCTAGTGTGTAATCATGGCAG 957
H3O1              GCATACACCAATGTACCAAACCCTATCAAACTCCGAATACCTAGTGTGTAATCATGGCAG 957
H7O-S1            GCATACACCAATGTACCAAACCCTATCAAACTCCGAATACCTAGTGTGTAATCATGGCAG 957
H9O-S1            GCATACACCAATGTACCAAACCCTATCAAACTCCGAATACCTAGTGTGTAATCATGGCAG 957
H9O-S2            GCATACACCAATGTACCAAACCCTATCAAACTCCGAATACCTAGTGTGTAATCATGGCAG 960
H11O-S1           GCATACACCAATGTACCAAACCCTATCAAACTCCGAATACCTAGTGTGTAATCATGGCAG 957
H14O-S1           GCATACACCAATGTACCAAACCCTATCAAACTCCGAATACCTAGTGTGTAATCATGGCAG 959
H16O-S1           GCATACACCAATGTACCAAACCCTATCAAACTCCGAATACCTAGTGTGTAATCATGGCAG 957
H20O-S1           GCATACACCAATGTACCAAACCCTATCAAACTCCGAATACCTAGTGTGTAATCATGGCAG 957
H21O-S1           GCATACACCAATGTACCAAACCCTATCAAACTCCGAATACCTAGTGTGTAATCATGGCAG 960
H21O-S2           GCATACACCAATGTACCAAACCCTATCAAACTCCGAATACCTAGTGTGTAATCATGGCAG 960
H21O-S5           GCATACACCAATGTACCAAACCCTATCAAACTCCGAATACCTAGTGTGTAATCATGGCAG 960
H22O-S1           GCATACACCAATGTACCAAACCCTATCAAACTCCGAATACCTAGTGTGTAATCATGGCAG 960
H23O-S1           GCATACACCAATGTACCAAACCCTATCAAACTCCGAATACCTAGTGTGTAATCATGGCAG 960
P2CDO3            GCATACACCAATGTACCAAACCCTATCAAACTCCGAATACCTAGTGTGTAATCATGGCAG 960
P2CDO4            GCATACACCAATGTACCAAACCCTATCAAACTCCGAATACCTAGTGTGTAATCATGGCAG 960
P2CDO-S6          GCATACACCAATGTACCAAACCCTATCAAACTCCGAATACCTAGTGTGTAATCATGGCAG 960
P6CDO1            GCATACACCAATGTACCAAACCCTATCAAACTCCGAATACCTAGTGTGTAATCATGGCAG 957
P12CDO-S1         GCATACACCAATGTACCAAACCCTATCAAACTCCGAATACCTAGTGTGTAATCATGGCAG 957
P13UCO-S3         GCATACACCAATGTACCAAACCCTATCAAACTCCGAATACCTAGTGTGTAATCATGGCAG 960
P15UCO-S2         GCATACACCAATGTACCAAACCCTATCAAACTCCGAATACCTAGTGTGTAATCATGGCAG 960
P16UCO-S1         GCATACACCAATGTACCAAACCCTATCAAACTCCGAATACCTAGTGTGTAATCATGGCAG 957
P16UCO-S2         GCATACACCAATGTACCAAACCCTATCAAACTCCGAATACCTAGTGTGTAATCATGGCAG 957
P18CDO-S1         GCATACACCAATGTACCAAACCCTATCAAACTCCGAATACCTAGTGTGTAATCATGGCAG 957
P20CDO-S1         GCATACACCAATGTACCAAACCCTATCAAACTCCGAATACCTAGTGTGTAATCATGGCAG 960
P20CDO-S2         GCATACACCAATGTACCAAACCCTATCAAACTCCGAATACCTAGTGTGTAATCATGGCAG 960
P20CDO-S3         GCATACACCAATGTACCAAACCCTATCAAACTCCGAATACCTAGTGTGTAATCATGGCAG 960
P21CDO-S1         GCATACACCAATGTACCAAACCCTATCAAACTCCGAATACCTAGTGTGTAATCATGGCAG 959
P21CDO-S2         GCATACACCAATGTACCAAACCCTATCAAACTCCGAATACCTAGTGTGTAATCATGGCAG 960
P21CDO-S4         GCATACACCAATGTACCAAACCCTATCAAACTCCGAATACCTAGTGTGTAATCATGGCAG 960
P24CDO-S2         GCATACACCAATGTACCAAACCCTATCAAACTCCGAATACCTAGTGTGTAATCATGGCAG 960
P24CDO-S3         GCATACACCAATGTACCAAACCCTATCAAACTCCGAATACCTAGTGTGTAATCATGGCAG 960
P24CDO-S4         GCATACACCAATGTACCAAACCCTATCAAACTCCGAATACCTAGTGTGTAATCATGGCAG 960
UNSW1             GCATACACCAATGTACCAAACCCTATCAAACTCCGAATACCTAGTGTGTAATCATGGCAG 960
UNSW2             GCATACACCAATGTACCAAACCCTATCAAACTCCGAATACCTAGTGTGTAATCATGGCAG 960
UNSW3             GCATACACCAATGTACCAAACCCTATCAAACTCCGAATACCTAGTGTGTAATCATGGCAG 960
UNSWCD            GCATACACCAATGTACCAAACCCTATCAAACTCCGAATACCTAGTGTGTAATCATGGCAG 957
UNSWCS            GCATACACCAATGTACCAAACCCTATCAAACTCCGAATACCTAGTGCGTAATCATGGCAG 960
                  ********************************************** *************
```

Supplementary Figure S2

```
ATCC33237       TCAGGCGGCGAGTGATAAAATCCGTCGTCGAGAGGGGAACAACCCAGACTAACAGCTAAG 1019
ATCC51562       TCAGGCGGCGAGTGATAAAATCCGTCGTCGAGAGGGGAACAACCCAGACTAACAGCTAAG 1016
H1O1            TCAGGCGGCGAGTGATAAAATCCGTCGTCGAGAGGGGAACAACCCAGACTAACAGCTAAG 1020
H10O-S1         TCAGGCGGCGAGTGATAAAATCCGTCGTCGAGAGGGGAACAACCCAGACTAACAGCTAAG 1016
H17O-S1         TCAGGCGGCGAGTGATAAAATCCGTCGTCGAGAGGGGAACAACCCAGACTAACAGCTAAG 1020
H21O-S3         TCAGGCGGCGAGTGATAAAATCCGTCGTCGAGAGGGGAACAACCCAGACTAACAGCTAAG 1020
P3UCO1          TCAGGCGGCGAGTGATAAAATCCGTCGTCGAGAGGGGAACAACCCAGACTAACAGCTAAG 1019
P3UCB1          TCAGGCGGCGAGTGATAAAATCCGTCGTCGAGAGGGGAACAACCCAGACTAACAGCTAAG 1019
P9CDO-S1        TCAGGCGGCGAGTGATAAAATCCGTCGTCGAGAGGGGAACAACCCAGACTAACAGCTAAG 1016
P20CDO-S4       TCAGGCGGCGAGTGATAAAATCCGTCGTCGAGAGGGGAACAACCCAGACTAACAGCTAAG 1019
13826           TCAGGCGGCGAGTGATAAAATCCGTCGTCGAGAGGGGAACAACCCAGACTAACAGCTAAG 1020
ATCC51561       TCAGGCGGCGAGTGATAAAATCCGTCGTCGAGAGGGGAACAACCCAGACTAACAGCTAAG 1017
H3O1            TCAGGCGGCGAGTGATAAAATCCGTCGTCGAGAGGGGAACAACCCAGACTAACAGCTAAG 1017
H7O-S1          TCAGGCGGCGAGTGATAAAATCCGTCGTCGAGAGGGGAACAACCCAGACTAACAGCTAAG 1017
H9O-S1          TCAGGCGGCGAGTGATAAAATCCGTCGTCGAGAGGGGAACAACCCAGACTAACAGCTAAG 1017
H9O-S2          TCAGGCGGCGAGTGATAAAATCCGTCGTCGAGAGGGGAACAACCCAGACTAACAGCTAAG 1020
H11O-S1         TCAGGCGGCGAGTGATAAAATCCGTCGTCGAGAGGGGAACAACCCAGACTAACAGCTAAG 1017
H14O-S1         TCAGGCGGCGAGTGATAAAATCCGTCGTCGAGAGGGGAACAACCCAGACTAACAGCTAAG 1019
H16O-S1         TCAGGCGGCGAGTGATAAAATCCGTCGTCGAGAGGGGAACAACCCAGACTAACAGCTAAG 1017
H20O-S1         TCAGGCGGCGAGTGATAAAATCCGTCGTCGAGAGGGGAACAACCCAGACTAACAGCTAAG 1017
H21O-S1         TCAGGCGGCGAGTGATAAAATCCGTCGTCGAGAGGGGAACAACCCAGACTAACAGCTAAG 1020
H21O-S2         TCAGGCGGCGAGTGATAAAATCCGTCGTCGAGAGGGGAACAACCCAGACTAACAGCTAAG 1020
H21O-S5         TCAGGCGGCGAGTGATAAAATCCGTCGTCGAGAGGGGAACAACCCAGACTAACAGCTAAG 1020
H22O-S1         TCAGGCGGCGAGTGATAAAATCCGTCGTCGAGAGGGGAACAACCCAGACTAACAGCTAAG 1020
H23O-S1         TCAGGCGGCGAGTGATAAAATCCGTCGTCGAGAGGGGAACAACCCAGACTAACAGCTAAG 1020
P2CDO3          TCAGGCGGCGAGTGATAAAATCCGTCGTCGAGAGGGGAACAACCCAGACTAACAGCTAAG 1020
P2CDO4          TCAGGCGGCGAGTGATAAAATCCGTCGTCGAGAGGGGAACAACCCAGACTAACAGCTAAG 1020
P2CDO-S6        TCAGGCGGCGAGTGATAAAATCCGTCGTCGAGAGGGGAACAACCCAGACTAACAGCTAAG 1020
P6CDO1          TCAGGCGGCGAGTGATAAAATCCGTCGTCGAGAGGGGAACAACCCAGACTAACAGCTAAG 1017
P12CDO-S1       TCAGGCGGCGAGTGATAAAATCCGTCGTCGAGAGGGGAACAACCCAGACTAACAGCTAAG 1017
P13UCO-S3       TCAGGCGGCGAGTGATAAAATCCGTCGTCGAGAGGGGAACAACCCAGACTAACAGCTAAG 1020
P15UCO-S2       TCAGGCGGCGAGTGATAAAATCCGTCGTCGAGAGGGGAACAACCCAGACTAACAGCTAAG 1020
P16UCO-S1       TCAGGCGGCGAGTGATAAAATCCGTCGTCGAGAGGGGAACAACCCAGACTAACAGCTAAG 1017
P16UCO-S2       TCAGGCGGCGAGTGATAAAATCCGTCGTCGAGAGGGGAACAACCCAGACTAACAGCTAAG 1017
P18CDO-S1       TCAGGCGGCGAGTGATAAAATCCGTCGTCGAGAGGGGAACAACCCAGACTAACAGCTAAG 1017
P20CDO-S1       TCAGGCGGCGAGTGATAAAATCCGTCGTCGAGAGGGGAACAACCCAGACTAACAGCTAAG 1020
P20CDO-S2       TCAGGCGGCGAGTGATAAAATCCGTCGTCGAGAGGGGAACAACCCAGACTAACAGCTAAG 1020
P20CDO-S3       TCAGGCGGCGAGTGATAAAATCCGTCGTCGAGAGGGGAACAACCCAGACTAACAGCTAAG 1020
P21CDO-S1       TCAGGCGGCGAGTGATAAAATCCGTCGTCGAGAGGGGAACAACCCAGACTAACAGCTAAG 1019
P21CDO-S2       TCAGGCGGCGAGTGATAAAATCCGTCGTCGAGAGGGGAACAACCCAGACTAACAGCTAAG 1020
P21CDO-S4       TCAGGCGGCGAGTGATAAAATCCGTCGTCGAGAGGGGAACAACCCAGACTAACAGCTAAG 1020
P24CDO-S2       TCAGGCGGCGAGTGATAAAATCCGTCGTCGAGAGGGGAACAACCCAGACTAACAGCTAAG 1020
P24CDO-S3       TCAGGCGGCGAGTGATAAAATCCGTCGTCGAGAGGGGAACAACCCAGACTAACAGCTAAG 1020
P24CDO-S4       TCAGGCGGCGAGTGATAAAATCCGTCGTCGAGAGGGGAACAACCCAGACTAACAGCTAAG 1020
UNSW1           TCAGGCGGCGAGTGATAAAATCCGTCGTCGAGAGGGGAACAACCCAGACTAACAGCTAAG 1020
UNSW2           TCAGGCGGCGAGTGATAAAATCCGTCGTCGAGAGGGGAACAACCCAGACTAACAGCTAAG 1020
UNSW3           TCAGGCGGCGAGTGATAAAATCCGTCGTCGAGAGGGGAACAACCCAGACTAACAGCTAAG 1020
UNSWCD          TCAGGCGGCGAGTGATAAAATCCGTCGTCGAGAGGGGAACAACCCAGACTAACAGCTAAG 1017
UNSWCS          TCAGGCGGCGAGTGATAAAATCCGTCGTCGAGAGGGGAACAACCCAGACTAACAGCTAAG 1020
                ************************************************************
```

Supplementary Figure S2

ATCC33237	GTCCCTAAATCTCATTTAAGTGGAAAACGATGTGGAGTTACTTAAACAACCAGGAGGTTG	1079
ATCC51562	GTCCCTAAATCTCATTTAAGTGGAAAACGATGTGGAGTTACTTAAACAACCAGGAGGTTG	1076
H1O1	GTCCCTAAATCTCATTTAAGTGGAAAACGATGTGGAGTTACTTAAACAACCAGGAGGTTG	1080
H10O-S1	GTCCCTAAATCTCATTTAAGTGGAAAACGATGTGGAGTTACTTAAACAACCAGGAGGTTG	1076
H17O-S1	GTCCCTAAATCTCATTTAAGTGGAAAACGATGTGGAGTTACTTAAACAACCAGGAGGTTG	1080
H21O-S3	GTCCCTAAATCTCATTTAAGTGGAAAACGATGTGGAGTTACTTAAACAACCAGGAGGTTG	1080
P3UCO1	GTCCCTAAATCTCATTTAAGTGGAAAACGATGTGGAGTTACTTAAACAACCAGGAGGTTG	1079
P3UCB1	GTCCCTAAATCTCATTTAAGTGGAAAACGATGTGGAGTTACTTAAACAACCAGGAGGTTG	1079
P9CDO-S1	GTCCCTAAATCTCATTTAAGTGGAAAACGATGTGGAGTTACTTAAACAACCAGGAGGTTG	1076
P20CDO-S4	GTCCCTAAATCTCATTTAAGTGGAAAACGATGTGGAGTTACTTAAACAACCAGGAGGTTG	1079
13826	GTCCCTAAATCTCATTTAAGTGGAAAACGATGTGGAGTTACTTAAACAACCAGGAGGTTG	1080
ATCC51561	GTCCCTAAATCTCATTTAAGTGGAAAACGATGTGGAGTTACTTAAACAACCAGGAGGTTG	1077
H3O1	GTCCCTAAATCTCATTTAAGTGGAAAACGATGTGGAGTTACTTAAACAACCAGGAGGTTG	1077
H7O-S1	GTCCCTAAATCTCATTTAAGTGGAAAACGATGTGGAGTTACTTAAACAACCAGGAGGTTG	1077
H9O-S1	GTCCCTAAATCTCATTTAAGTGGAAAACGATGTGGAGTTACTTAAACAACCAGGAGGTTG	1077
H9O-S2	GTCCCTAAATCTCATTTAAGTGGAAAACGATGTGGAGTTACTTAAACAACCAGGAGGTTG	1080
H11O-S1	GTCCCTAAATCTCATTTAAGTGGAAAACGATGTGGAGTTACTTAAACAACCAGGAGGTTG	1077
H14O-S1	GTCCCTAAATCTCATTTAAGTGGAAAACGATGTGGAGTTACTTAAACAACCAGGAGGTTG	1079
H16O-S1	GTCCCTAAATCTCATTTAAGTGGAAAACGATGTGGAGTTACTTAAACAACCAGGAGGTTG	1077
H20O-S1	GTCCCTAAATCTCATTTAAGTGGAAAACGATGTGGAGTTACTTAAACAACCAGGAGGTTG	1077
H21O-S1	GTCCCTAAATCTCATTTAAGTGGAAAACGATGTGGAGTTACTTAAACAACCAGGAGGTTG	1080
H21O-S2	GTCCCTAAATCTCATTTAAGTGGAAAACGATGTGGAGTTACTTAAACAACCAGGAGGTTG	1080
H21O-S5	GTCCCTAAATCTCATTTAAGTGGAAAACGATGTGGAGTTACTTAAACAACCAGGAGGTTG	1080
H22O-S1	GTCCCTAAATCTCATTTAAGTGGAAAACGATGTGGAGTTACTTAAACAACCAGGAGGTTG	1080
H23O-S1	GTCCCTAAATCTCATTTAAGTGGAAAACGATGTGGAGTTACTTAAACAACCAGGAGGTTG	1080
P2CDO3	GTCCCTAAATCTCATTTAAGTGGAAAACGATGTGGAGTTACTTAAACAACCAGGAGGTTG	1080
P2CDO4	GTCCCTAAATCTCATTTAAGTGGAAAACGATGTGGAGTTACTTAAACAACCAGGAGGTTG	1080
P2CDO-S6	GTCCCTAAATCTCATTTAAGTGGAAAACGATGTGGAGTTACTTAAACAACCAGGAGGTTG	1080
P6CDO1	GTCCCTAAATCTCATTTAAGTGGAAAACGATGTGGAGTTACTTAAACAACCAGGAGGTTG	1077
P12CDO-S1	GTCCCTAAATCTCATTTAAGTGGAAAACGATGTGGAGTTACTTAAACAACCAGGAGGTTG	1077
P13UCO-S3	GTCCCTAAATCTCATTTAAGTGGAAAACGATGTGGAGTTACTTAAACAACCAGGAGGTTG	1080
P15UCO-S2	GTCCCTAAATCTCATTTAAGTGGAAAACGATGTGGAGTTACTTAAACAACCAGGAGGTTG	1080
P16UCO-S1	GTCCCTAAATCTCATTTAAGTGGAAAACGATGTGGAGTTACTTAAACAACCAGGAGGTTG	1077
P16UCO-S2	GTCCCTAAATCTCATTTAAGTGGAAAACGATGTGGAGTTACTTAAACAACCAGGAGGTTG	1077
P18CDO-S1	GTCCCTAAATCTCATTTAAGTGGAAAACGATGTGGAGTTACTTAAACAACCAGGAGGTTG	1077
P20CDO-S1	GTCCCTAAATCTCATTTAAGTGGAAAACGATGTGGAGTTACTTAAACAACCAGGAGGTTG	1080
P20CDO-S2	GTCCCTAAATCTCATTTAAGTGGAAAACGATGTGGAGTTACTTAAACAACCAGGAGGTTG	1080
P20CDO-S3	GTCCCTAAATCTCATTTAAGTGGAAAACGATGTGGAGTTACTTAAACAACCAGGAGGTTG	1080
P21CDO-S1	GTCCCTAAATCTCATTTAAGTGGAAAACGATGTGGAGTTACTTAAACAACCAGGAGGTTG	1079
P21CDO-S2	GTCCCTAAATCTCATTTAAGTGGAAAACGATGTGGAGTTACTTAAACAACCAGGAGGTTG	1080
P21CDO-S4	GTCCCTAAATCTCATTTAAGTGGAAAACGATGTGGAGTTACTTAAACAACCAGGAGGTTG	1080
P24CDO-S2	GTCCCTAAATCTCATTTAAGTGGAAAACGATGTGGAGTTACTTAAACAACCAGGAGGTTG	1080
P24CDO-S3	GTCCCTAAATCTCATTTAAGTGGAAAACGATGTGGAGTTACTTAAACAACCAGGAGGTTG	1080
P24CDO-S4	GTCCCTAAATCTCATTTAAGTGGAAAACGATGTGGAGTTACTTAAACAACCAGGAGGTTG	1080
UNSW1	GTCCCTAAATCTCATTTAAGTGGAAAACGATGTGGAGTTACTTAAACAACCAGGAGGTTG	1080
UNSW2	GTCCCTAAATCTCATTTAAGTGGAAAACGATGTGGAGTTACTTAAACAACCAGGAGGTTG	1080
UNSW3	GTCCCTAAATCTCATTTAAGTGGAAAACGATGTGGAGTTACTTAAACAACCAGGAGGTTG	1080
UNSWCD	GTCCCTAAATCTCATTTAAGTGGAAAACGATGTGGAGTTACTTAAACAACCAGGAGGTTG	1077
UNSWCS	GTCCCTAAATCTCATTTAAGTGGAAAACGATGTGGAGTTACTTAAACAACCAGGAGGTTG	1080
	**	

Supplementary Figure S2

```
ATCC33237       GCTTAGAAGCAGCCATCCTTTAAAGAAAGCGTAATAGCTCACTGGTCTAGTGATTCTGCG 1139
ATCC51562       GCTTAGAAGCAGCCATCCTTTAAAGAAAGCGTAATAGCTCACTGGTCTAGTGATTCTGCG 1136
H1O1            GCTTAGAAGCAGCCATCCTTTAAAGAAAGCGTAATAGCTCACTGGTCTAGTGATTCTGCG 1140
H10O-S1         GCTTAGAAGCAGCCATCCTTTAAAGAAAGCGTAATAGCTCACTGGTCTAGTGATTCTGCG 1136
H17O-S1         GCTTAGAAGCAGCCATCCTTTAAAGAAAGCGTAATAGCTCACTGGTCTAGTGATTCTGCG 1140
H21O-S3         GCTTAGAAGCAGCCATCCTTTAAAGAAAGCGTAATAGCTCACTGGTCTAGTGATTCTGCG 1140
P3UCO1          GCTTAGAAGCAGCCATCCTTTAAAGAAAGCGTAATAGCTCACTGGTCTAGTGATTCTGCG 1139
P3UCB1          GCTTAGAAGCAGCCATCCTTTAAAGAAAGCGTAATAGCTCACTGGTCTAGTGATTCTGCG 1139
P9CDO-S1        GCTTAGAAGCAGCCATCCTTTAAAGAAAGCGTAATAGCTCACTGGTCTAGTGATTCTGCG 1136
P20CDO-S4       GCTTAGAAGCAGCCATCCTTTAAAGAAAGCGTAATAGCTCACTGGTCTAGTGATTCTGCG 1139
13826           GCTTAGAAGCAGCCATCCTTTAAAGAAAGCGTAATAGCTCACTGGTCTAGTGATTCTGCG 1140
ATCC51561       GCTTAGAAGCAGCCATCCTTTAAAGAAAGCGTAATAGCTCACTGGTCTAGTGATTCTGCG 1137
H3O1            GCTTAGAAGCAGCCATCCTTTAAAGAAAGCGTAATAGCTCACTGGTCTAGTGATTCTGCG 1137
H7O-S1          GCTTAGAAGCAGCCATCCTTTAAAGAAAGCGTAATAGCTCACTGGTCTAGTGATTCTGCG 1137
H9O-S1          GCTTAGAAGCAGCCATCCTTTAAAGAAAGCGTAATAGCTCACTGGTCTAGTGATTCTGCG 1137
H9O-S2          GCTTAGAAGCAGCCATCCTTTAAAGAAAGCGTAATAGCTCACTGGTCTAGTGATTCTGCG 1140
H11O-S1         GCTTAGAAGCAGCCATCCTTTAAAGAAAGCGTAATAGCTCACTGGTCTAGTGATTCTGCG 1137
H14O-S1         GCTTAGAAGCAGCCATCCTTTAAAGAAAGCGTAATAGCTCACTGGTCTAGTGATTCTGCG 1139
H16O-S1         GCTTAGAAGCAGCCATCCTTTAAAGAAAGCGTAATAGCTCACTGGTCTAGTGATTCTGCG 1137
H20O-S1         GCTTAGAAGCAGCCATCCTTTAAAGAAAGCGTAATAGCTCACTGGTCTAGTGATTCTGCG 1137
H21O-S1         GCTTAGAAGCAGCCATCCTTTAAAGAAAGCGTAATAGCTCACTGGTCTAGTGATTCTGCG 1140
H21O-S2         GCTTAGAAGCAGCCATCCTTTAAAGAAAGCGTAATAGCTCACTGGTCTAGTGATTCTGCG 1140
H21O-S5         GCTTAGAAGCAGCCATCCTTTAAAGAAAGCGTAATAGCTCACTGGTCTAGTGATTCTGCG 1140
H22O-S1         GCTTAGAAGCAGCCATCCTTTAAAGAAAGCGTAATAGCTCACTGGTCTAGTGATTCTGCG 1140
H23O-S1         GCTTAGAAGCAGCCATCCTTTAAAGAAAGCGTAATAGCTCACTGGTCTAGTGATTCTGCG 1140
P2CDO3          GCTTAGAAGCAGCCATCCTTTAAAGAAAGCGTAATAGCTCACTGGTCTAGTGATTCTGCG 1140
P2CDO4          GCTTAGAAGCAGCCATCCTTTAAAGAAAGCGTAATAGCTCACTGGTCTAGTGATTCTGCG 1140
P2CDO-S6        GCTTAGAAGCAGCCATCCTTTAAAGAAAGCGTAATAGCTCACTGGTCTAGTGATTCTGCG 1140
P6CDO1          GCTTAGAAGCAGCCATCCTTTAAAGAAAGCGTAATAGCTCACTGGTCTAGTGATTCTGCG 1137
P12CDO-S1       GCTTAGAAGCAGCCATCCTTTAAAGAAAGCGTAATAGCTCACTGGTCTAGTGATTCTGCG 1137
P13UCO-S3       GCTTAGAAGCAGCCATCCTTTAAAGAAAGCGTAATAGCTCACTGGTCTAGTGATTCTGCG 1140
P15UCO-S2       GCTTAGAAGCAGCCATCCTTTAAAGAAAGCGTAATAGCTCACTGGTCTAGTGATTCTGCG 1140
P16UCO-S1       GCTTAGAAGCAGCCATCCTTTAAAGAAAGCGTAATAGCTCACTGGTCTAGTGATTCTGCG 1137
P16UCO-S2       GCTTAGAAGCAGCCATCCTTTAAAGAAAGCGTAATAGCTCACTGGTCTAGTGATTCTGCG 1137
P18CDO-S1       GCTTAGAAGCAGCCATCCTTTAAAGAAAGCGTAATAGCTCACTGGTCTAGTGATTCTGCG 1137
P20CDO-S1       GCTTAGAAGCAGCCATCCTTTAAAGAAAGCGTAATAGCTCACTGGTCTAGTGATTCTGCG 1140
P20CDO-S2       GCTTAGAAGCAGCCATCCTTTAAAGAAAGCGTAATAGCTCACTGGTCTAGTGATTCTGCG 1140
P20CDO-S3       GCTTAGAAGCAGCCATCCTTTAAAGAAAGCGTAATAGCTCACTGGTCTAGTGATTCTGCG 1140
P21CDO-S1       GCTTAGAAGCAGCCATCCTTTAAAGAAAGCGTAATAGCTCACTGGTCTAGTGATTCTGCG 1139
P21CDO-S2       GCTTAGAAGCAGCCATCCTTTAAAGAAAGCGTAATAGCTCACTGGTCTAGTGATTCTGCG 1140
P21CDO-S4       GCTTAGAAGCAGCCATCCTTTAAAGAAAGCGTAATAGCTCACTGGTCTAGTGATTCTGCG 1140
P24CDO-S2       GCTTAGAAGCAGCCATCCTTTAAAGAAAGCGTAATAGCTCACTGGTCTAGTGATTCTGCG 1140
P24CDO-S3       GCTTAGAAGCAGCCATCCTTTAAAGAAAGCGTAATAGCTCACTGGTCTAGTGATTCTGCG 1140
P24CDO-S4       GCTTAGAAGCAGCCATCCTTTAAAGAAAGCGTAATAGCTCACTGGTCTAGTGATTCTGCG 1140
UNSW1           GCTTAGAAGCAGCCATCCTTTAAAGAAAGCGTAATAGCTCACTGGTCTAGTGATTCTGCG 1140
UNSW2           GCTTAGAAGCAGCCATCCTTTAAAGAAAGCGTAATAGCTCACTGGTCTAGTGATTCTGCG 1140
UNSW3           GCTTAGAAGCAGCCATCCTTTAAAGAAAGCGTAATAGCTCACTGGTCTAGTGATTCTGCG 1140
UNSWCD          GCTTAGAAGCAGCCATCCTTTAAAGAAAGCGTAATAGCTCACTGGTCTAGTGATTCTGCG 1137
UNSWCS          GCTTAGAAGCAGCCATCCTTTAAAGAAAGCGTAATAGCTCACTGGTCTAGTGATTCTGCG 1140
                ************************************************************
```

Supplementary Figure S2

```
ATCC33237       CGGAAAATATAACGGGGCTAAAATGAGTACCGAAGCTTTAGACTTAGTTTTACTAAGTGG 1199
ATCC51562       CGGAAAATATAACGGGGCTAAAATGAGTACCGAAGCTTTAGACTTAGTTTTACTAAGTGG 1196
H1O1            CGGAAAATATAACGGGGCTAAAATGAGTACCGAAGCTTTAGACTTAGTTTTACTAAGTGG 1200
H10O-S1         CGGAAAATATAACGGGGCTAAAATGAGTACCGAAGCTTTAGACTTAGTTTTACTAAGTGG 1196
H17O-S1         CGGAAAATATAACGGGGCTAAAATGAGTACCGAAGCTTTAGACTTAGTTTTACTAAGTGG 1200
H21O-S3         CGGAAAATATAACGGGGCTAAAATGAGTACCGAAGCTTTAGACTTAGTTTTACTAAGTGG 1200
P3UCO1          CGGAAAATATAACGGGGCTAAAATGAGTACCGAAGCTTTAGACTTAGTTTTACTAAGTGG 1199
P3UCB1          CGGAAAATATAACGGGGCTAAAATGAGTACCGAAGCTTTAGACTTAGTTTTACTAAGTGG 1199
P9CDO-S1        CGGAAAATATAACGGGGCTAAAATGAGTACCGAAGCTTTAGACTTAGTTTTACTAAGTGG 1196
P20CDO-S4       CGGAAAATATAACGGGGCTAAAATGAGTACCGAAGCTTTAGACTTAGTTTTACTAAGTGG 1199
13826           CGGAAAATATAACGGGGCTAAAATGAGTACCGAAGCTTTAGACTTAGTTTTACTAAGTGG 1200
ATCC51561       CGGAAAATATAACGGGGCTAAAATGAGTACCGAAGCTTTAGACTTAGTTTTACTAAGTGG 1197
H3O1            CGGAAAATATAACGGGGCTAAAATGAGTACCGAAGCTTTAGACTTAGTTTTACTAAGTGG 1197
H7O-S1          CGGAAAATATAACGGGGCTAAAATGAGTACCGAAGCTTTAGACTTAGTTTTACTAAGTGG 1197
H9O-S1          CGGAAAATATAACGGGGCTAAAATGAGTACCGAAGCTTTAGACTTAG-TTTACTAAGTGG 1196
H9O-S2          CGGAAAATATAACGGGGCTAAAATGAGTACCGAAGCTTTAGACTTAGTTTTACTAAGTGG 1200
H11O-S1         CGGAAAATATAACGGGGCTAAAATGAGTACCGAAGCTTTAGACTTAGTTTTACTAAGTGG 1197
H14O-S1         CGGAAAATATAACGGGGCTAAAATGAGTACCGAAGCTTTAGACTTAGTTTTACTAAGTGG 1199
H16O-S1         CGGAAAATATAACGGGGCTAAAATGAGTACCGAAGCTTTAGACTTAGTTTTACTAAGTGG 1197
H20O-S1         CGGAAAATATAACGGGGCTAAAATGAGTACCGAAGCTTTAGACTTAGTTTTACTAAGTGG 1197
H21O-S1         CGGAAAATATAACGGGGCTAAAATGAGTACCGAAGCTTTAGACTTAGTTTTACTAAGTGG 1200
H21O-S2         CGGAAAATATAACGGGGCTAAAATGAGTACCGAAGCTTTAGACTTAGTTTTACTAAGTGG 1200
H21O-S5         CGGAAAATATAACGGGGCTAAAATGAGTACCGAAGCTTTAGACTTAGTTTTACTAAGTGG 1200
H22O-S1         CGGAAAATATAACGGGGCTAAAATGAGTACCGAAGCTTTAGACTTAGTTTTACTAAGTGG 1200
H23O-S1         CGGAAAATATAACGGGGCTAAAATGAGTACCGAAGCTTTAGACTTAG-TTTACTAAGTGG 1199
P2CDO3          CGGAAAATATAACGGGGCTAAAATGAGTACCGAAGCTTTAGACTTAGTTTTACTAAGTGG 1200
P2CDO4          CGGAAAATATAACGGGGCTAAAATGAGTACCGAAGCTTTAGACTTAGTTTTACTAAGTGG 1200
P2CDO-S6        CGGAAAATATAACGGGGCTAAAATGAGTACCGAAGCTTTAGACTTAGTTTTACTAAGTGG 1200
P6CDO1          CGGAAAATATAACGGGGCTAAAATGAGTACCGAAGCTTTAGACTTAGTTTTACTAAGTGG 1197
P12CDO-S1       CGGAAAATATAACGGGGCTAAAATGAGTACCGAAGCTTTAGACTTAGTTTTACTAAGTAG 1197
P13UCO-S3       CGGAAAATATAACGGGGCTAAAATGAGTACCGAAGCTTTAGACTTAG-TTTACTAAGTGG 1199
P15UCO-S2       CGGAAAATATAACGGGGCTAAAATGAGTACCGAAGCTTTAGACTTAGTTTTACTAAGTGG 1200
P16UCO-S1       CGGAAAATATAACGGGGCTAAAATGAGTACCGAAGCTTTAGACTTAGTTTTACTAAGTGG 1197
P16UCO-S2       CGGAAAATATAACGGGGCTAAAATGAGTACCGAAGCTTTAGACTTAG-TTTACTAAGTGG 1196
P18CDO-S1       CGGAAAATATAACGGGGCTAAAATGAGTACCGAAGCTTTAGACTTAGTTTTACTAAGTGG 1197
P20CDO-S1       CGGAAAATATAACGGGGCTAAAATGAGTACCGAAGCTTTAGACTTAG-TTTACTAAGTGG 1199
P20CDO-S2       CGGAAAATATAACGGGGCTAAAATGAGTACCGAAGCTTTAGACTTAGTTTTACTAAGTGG 1200
P20CDO-S3       CGGAAAATATAACGGGGCTAAAATGAGTACCGAAGCTTTAGACTTAG-TTTACTAAGTGG 1199
P21CDO-S1       CGGAAAATATAACGGGGCTAAAATGAGTACCGAAGCTTTAGACTTAGTTTTACTAAGTGG 1199
P21CDO-S2       CGGAAAATATAACGGGGCTAAAATGAGTACCGAAGCTTTAGACTTAGTTTTACTAAGTGG 1200
P21CDO-S4       CGGAAAATATAACGGGGCTAAAATGAGTACCGAAGCTTTAGACTTAGTTTTACTAAGTGG 1200
P24CDO-S2       CGGAAAATATAACGGGGCTAAAATGAGTACCGAAGCTTTAGACTTAGTTTTACTAAGTGG 1200
P24CDO-S3       CGGAAAATATAACGGGGCTAAAATGAGTACCGAAGCTTTAGACTTAGTTTTACTAAGTGG 1200
P24CDO-S4       CGGAAAATATAACGGGGCTAAAATGAGTACCGAAGCTTTAGACTTAGTTTTACTAAGTGG 1200
UNSW1           CGGAAAATATAACGGGGCTAAAATGAGTACCGAAGCTTTAGACTTAGTTTTACTAAGTGG 1200
UNSW2           CGGAAAATATAACGGGGCTAAAATGAGTACCGAAGCTTTAGACTTAGTTTTACTAAGTGG 1200
UNSW3           CGGAAAATATAACGGGGCTAAAATGAGTACCGAAGCTTTAGACTTAGTTTTACTAAGTGG 1200
UNSWCD          CGGAAAATATAACGGGGCTAAAATGAGTACCGAAGCTTTAGACTTAGTTTTACTAAGTGG 1197
UNSWCS          CGGAAAATATAACGGGGCTAAAATGAGTACCGAAGCTTTAGACTTAGTTTTACTAAGTGG 1200
                *********************************************** ********** *
```

Supplementary Figure S2

```
ATCC33237      TAGGAGAGCGTTGTATTTGCGTTGAAGGTATACCGGTAAGGAGTGCTGGAGCGAATACAA 1259
ATCC51562      TAGGAGAGCGTTGTATTTGCATTGAAGGTATACCGGTAAGGAGTGCTGGAGCGAATACAA 1256
H1O1           TAGGAGAGCGTTGTATTTGCGTCGAAGGTATACCGGTAAGGAGTGCTGGAGCGAATACAA 1260
H10O-S1        TAGGAGAGCGTTGTATTTGCGTCGAAGGTATACCGGTAAGGAGTGCTGGAGCGAATACAA 1256
H17O-S1        TAGGAGAGCGTTGTATTTGCGTTGAAGGTGTACCGGTAAGGAGTGCTGGAGCGAATACAA 1260
H21O-S3        TAGGAGAGCGTTGTATTTGCGTTGAAGGTGTACCGGTAAGGAGTGCTGGAGCGAATACAA 1260
P3UCO1         TAGGAGAGCGTTGTATTTGCGTTGAAGGTATACCGGTAAGGAGTGCTGGAGTGAATACAA 1259
P3UCB1         TAGGAGAGCGTTGTATTTGCGTTGAAGGTATACCGGTAAGGAGTGCTGGAGTGAATACAA 1259
P9CDO-S1       TAGGAGAGCGTTGTATTTGCGTTGAAGGTATACCGGTAAGGAGTGCTGGAGCGAATACAA 1256
P20CDO-S4      TAGGAGAGCGTTGTATTTGCGTTGAAGGTATACCGGTAAGGAGTGCTGGAGCGAATACAA 1259
13826          TAGGAGAGCGTTGTATTTGCGTTGAAGGTATACCGGTAAGGAGTGCTGGAGCGAATACAA 1260
ATCC51561      TAGGAGAGCGTTGTATTTGCGTTGAAGGTATACCGGTAAGGAGTGCTGGAGCGAATACAA 1257
H3O1           TAGGAGAGCGTTGTATTTGCGTTGAAGGTATACCGGTAAGGAGTGCTGGAGCGAATACAA 1257
H7O-S1         TAGGAGAGCGTTGTATTTGCGTTGAAGGTATACCGGTAAGGAGTGCTGGAGCGAATACAA 1257
H9O-S1         TAGGAGAGCGTTGTATTTGCGTTGAAGGTATACCGGTAAGGAGTGCTGGAGCGAATACAA 1256
H9O-S2         TAGGAGAGCGTTGTATTTGCGTTGAAGGTATACCGGTAAGGAGTGCTGGAGCGAATACAA 1260
H11O-S1        TAGGAGAGCGTTGTATTTGCGTTGAAGGTATACCGGTAAGGAGTGCTGGAGCAAATACAA 1257
H14O-S1        TAGGAGAGCGTTGTATTTGCGTTGAAGGTATACCGGTAAGGAGTGCTGGAGCGAATACAA 1259
H16O-S1        TAGGAGAGCGTTGTATTTGCGTTGAAGGTATACCGGTAAGGAGTGCTGGAGCGAATACAA 1257
H20O-S1        TAGGAGAGCGTTGTATTTGCGTTGAAGGTATACCGGTAAGGAGTGCTGGAGCGAATACAA 1257
H21O-S1        TAGGAGAGCGTTGTATTTGCGTTGAAGGTATACCGGTAAGGAGTGCTGGAGCGAATACAA 1260
H21O-S2        TAGGAGAGCGTTGTATTTGCGTTGAAGGTATACCGGTAAGGAGTGCTGGAGCGAATACAA 1260
H21O-S5        TAGGAGAGCGTTGTATTTGCGTTGAAGGTATACCGGTAAGGAGTGCTGGAGCGAATACAA 1260
H22O-S1        TAGGAGAGCGTTGTATTTGCGTTGAAGGTATACCGGTAAGGAGTGCTGGAGCGAATACAA 1260
H23O-S1        TAGGAGAGCGTTGTATTTGCATTGAAGGTATACCGGTAAGGAGTGCTGGAGCGAATACAA 1259
P2CDO3         TAGGAGAGCGTTGTATTTGCGTTGAAGGTATACCGGTAAGGAGTGCTGGAGCGAATACAA 1260
P2CDO4         TAGGAGAGCGTTGTATTTGCGTTGAAGGTATACCGGTAAGGAGTGCTGGAGCGAATACAA 1260
P2CDO-S6       TAGGAGAGCGTTGTATTTGCGTTGAAGGTATACCGGTAAGGAGTGCTGGAGCGAATACAA 1260
P6CDO1         TAGGAGAGCGTTGTATTTGCGTTGAAGGTATACCGGTAAGGAGTGCTGGAGCGAATACAA 1257
P12CDO-S1      TAGGAGAGCGTTGTATTTGCGTTGAAGGTATACCGGTAAGGAGTGCTGGAGCGAATACAA 1257
P13UCO-S3      TAGGAGAGCGTTGTATTTGCATTGAAGGTATACCGGTAAGGAGTGCTGGAGCGAATACAA 1259
P15UCO-S2      TAGGAGAGCGTTGTATTTGCGTTGAAGGTATACCGGTAAGGAGTGCTGGAGCGAATACAA 1260
P16UCO-S1      TAGGAGAGCGTTGTATTTGCGTTGAAGGTATACCGGTAAGGAGTGCTGGAGCGAATACAA 1257
P16UCO-S2      TAGGAGAGCGTTGTATTTGCATTGAAGGTATACCGGTAAGGAGTGCTGGAGCGAATACAA 1256
P18CDO-S1      TAGGAGAGCGTTGTATTTGCGTTGAAGGTATACCGGTAAGGAGTGCTGGAGCGAATACAA 1257
P20CDO-S1      TAGGAGAGCGTTGTATTTGCGTTGAAGGTATACCGGTAAGGAGTGCTGGAGCGAATACAA 1259
P20CDO-S2      TAGGAGAGCGTTGTATTTGCGTTGAAGGTATACCGGTAAGGAGTGCTGGAGCGAATACAA 1260
P20CDO-S3      TAGGAGAGCGTTGTATTTGCGTTGAAGGTATACCGGTAAGGAGTGCTGGAGCGAATACAA 1259
P21CDO-S1      TAGGAGAGCGTTGTATTTGCGTTGAAGGTATACCGGTAAGGAGTGCTGGAGCGAATACAA 1259
P21CDO-S2      TAGGAGAGCGTTGTATTTGCGTTGAAGGTATACCGGTAAGGAGTGCTGGAGCGAATACAA 1260
P21CDO-S4      TAGGAGAGCGTTGTATTTGCGTTGAAGGTATACCGGTAAGGAGTGCTGGAGCGAATACAA 1260
P24CDO-S2      TAGGAGAGCGTTGTATTTGCGTTGAAGGTATACCGGTAAGGAGTGCTGGAGCGAATACAA 1260
P24CDO-S3      TAGGAGAGCGTTGTATTTGCGTTGAAGGTATACCGGTAAGGAGTGCTGGAGCGAATACAA 1260
P24CDO-S4      TAGGAGAGCGTTGTATTTGCGTTGAAGGTATACCGGTAAGGAGTGCTGGAGCGAATACAA 1260
UNSW1          TAGGAGAGCGTTGTATTTGCGTTGAAGGTATACCGGTAAGGAGTGCTGGAGCGAATACAA 1260
UNSW2          TAGGAGAGCGTTGTATTTGCGTTGAAGGTATACCGGTAAGGAGTGCTGGAGCGAATACAA 1260
UNSW3          TAGGAGAGCGTTGTATTTGCGTTGAAGGTATACCGGTAAGGAGTGCTGGAGCGAATACAA 1260
UNSWCD         TAGGAGAGCGTTGTATTTGCGTTGAAGGTATACCGGTAAGGAGTGCTGGAGCGAATACAA 1257
UNSWCS         TAGGAGAGCGTTGTATTTGCGTTGAAGGTATACCGGTAAGGAGTGCTGGAGCGAATACAA 1260
               ******************** * ****** *********************  *******
```

Supplementary Figure S2

```
ATCC33237      GTGAGCATGCAGGCATGAGTAGCGATAATTGGGGTGAGAATCCCCAACGCCGTAAACCCA 1319
ATCC51562      GTGAGCATGCAGGCATGAGTAGCGATAATTGGGGTGAGAATCCCCAACGCCGTAAACCCA 1316
H1O1           GTGAGCATGCAGGCATGAGTAGCGATAATTGGGGTGAGAATCCCCAACGCCGTAAACCCA 1320
H10O-S1        GTGAGCATGCAGGCATGAGTAGCGATAATTGGGGTGAGAATCCCCAACGCCGTAAACCCA 1316
H17O-S1        GTGAGCATGCAGGCATGAGTAGCGATAATTGGGGTGAGAATCCCCAACGCCGTAAACCCA 1320
H21O-S3        GTGAGCATGCAGGCATGAGTAGCGATAATTGGGGTGAGAATCCCCAACGCCGTAAACCCA 1320
P3UCO1         GTGAGCATGCAGGCATGAGTAGCGATAATTGGGGTGAGAATCCCCAACGCCGTAAACCCA 1319
P3UCB1         GTGAGCATGCAGGCATGAGTAGCGATAATTGGGGTGAGAATCCCCAACGCCGTAAACCCA 1319
P9CDO-S1       GTGAGCATGCAGGCATGAGTAGCGATAATTGGGGTGAGAATCCCCAACGCCGTAAACCCA 1316
P20CDO-S4      GTGAGCATGCAGGCATGAGTAGCGATAATTGGGGTGAGAATCCCCAACGCCGTAAACCCA 1319
13826          GTGAGCATGCAGGCATGAGTAGCGATAATTGGGGTGAGAATCCCCAACGCCGTAAACCCA 1320
ATCC51561      GTGAGCATGCAGGCATGAGTAGCGATAATTGGGGTGAGAATCCCCAACGCCGTAAACCCA 1317
H3O1           GTGAGCATGCAGGCATGAGTAGCGATAATTGGGGTGAGAATCCCCAACGCCGTAAACCCA 1317
H7O-S1         GTGAGCATGCAGGCATGAGTAGCGATAATTGGGGTGAGAATCCCCAACGCCGTAAACCCA 1317
H9O-S1         GTGAGCATGCAGGCATGAGTAGCGATAATTGGGGTGAGAATCCCCAACGCCGTAAACCCA 1316
H9O-S2         GTGAGCATGCAGGCATGAGTAGCGATAATTGGGGTGAGAATCCCCAACGCCGTAAACCCA 1320
H11O-S1        GTGAGCATGCAGGCATGAGTAGCGATAATTGGGGTGAGAATCCCCAACGCCGTAAACCCA 1317
H14O-S1        GTGAGCATGCAGGCATGAGTAGCGATAATTGGGGTGAGAATCCCCAACGCCGTAAACCCA 1319
H16O-S1        GTGAGCATGCAGGCATGAGTAGCGATAATTGGGGTGAGAATCCCCAACGCCGTAAACCCA 1317
H20O-S1        GTGAGCATGCAGGCATGAGTAGCGATAATTGGGGTGAGAATCCCCAACGCCGTAAACCCA 1317
H21O-S1        GTGAGCATGCAGGCATGAGTAGCGATAATTGGGGTGAGAATCCCCAACGCCGTAAACCCA 1320
H21O-S2        GTGAGCATGCAGGCATGAGTAGCGATAATTGGGGTGAGAATCCCCAACGCCGTAAACCCA 1320
H21O-S5        GTGAGCATGCAGGCATGAGTAGCGATAATTGGGGTGAGAATCCCCAACGCCGTAAACCCA 1320
H22O-S1        GTGAGCATGCAGGCATGAGTAGCGATAATTGGGGTGAGAATCCCCAACGCCGTAAACCCA 1320
H23O-S1        GTGAGCATGCAGGCATGAGTAGCGATAATTGGGGTGAGAATCCCCAACGCCGTAAACCCA 1319
P2CDO3         GTGAGCATGCAGGCATGAGTAGCGATAATTGGGGTGAGAATCCCCAACGCCGTAAACCCA 1320
P2CDO4         GTGAGCATGCAGGCATGAGTAGCGATAATTGGGGTGAGAATCCCCAACGCCGTAAACCCA 1320
P2CDO-S6       GTGAGCATGCAGGCATGAGTAGCGATAATTGGGGTGAGAATCCCCAACGCCGTAAACCCA 1320
P6CDO1         GTGAGCATGCAGGCATGAGTAGCGATAATTGGGGTGAGAATCCCCAACGCCGTAAACCCA 1317
P12CDO-S1      GTGAGCATGCAGGCATGAGTAGCGATAATTGGGGTGAGAATCCCCAACGCCGTAAACCCA 1317
P13UCO-S3      GTGAGCATGCAGGCATGAGTAGCGATAATTGGGGTGAGAATCCCCAACGCCGTAAACCCA 1319
P15UCO-S2      GTGAGCATGCAGGCATGAGTAGCGATAATTGGGGTGAGAATCCCCAACGCCGTAAACCCA 1320
P16UCO-S1      GTGAGCATGCAGGCATGAGTAGCGATAATTGGGGTGAGAATCCCCAACGCCGTAAACCCA 1317
P16UCO-S2      GTGAGCATGCAGGCATGAGTAGCGATAATTGGGGTGAGAATCCCCAACGCCGTAAACCCA 1316
P18CDO-S1      GTGAGCATGCAGGCATGAGTAGCGATAATTGGGGTGAGAATCCCCAACGCCGTAAACCCA 1317
P20CDO-S1      GTGAGCATGCAGGCATGAGTAGCGATAATTGGGGTGAGAATCCCCAACGCCGTAAACCCA 1319
P20CDO-S2      GTGAGCATGCAGGCATGAGTAGCGATAATTGGGGTGAGAATCCCCAACGCCGTAAACCCA 1320
P20CDO-S3      GTGAGCATGCAGGCATGAGTAGCGATAATTGGGGTGAGAATCCCCAACGCCGTAAACCCA 1319
P21CDO-S1      GTGAGCATGCAGGCATGAGTAGCGATAATTGGGGTGAGAATCCCCAACGCCGTAAACCCA 1319
P21CDO-S2      GTGAGCATGCAGGCATGAGTAGCGATAATTGGGGTGAGAATCCCCAACGCCGTAAACCCA 1320
P21CDO-S4      GTGAGCATGCAGGCATGAGTAGCGATAATTGGGGTGAGAATCCCCAACGCCGTAAACCCA 1320
P24CDO-S2      GTGAGCATGCAGGCATGAGTAGCGATAATTGGGGTGAGAATCCCCAACGCCGTAAACCCA 1320
P24CDO-S3      GTGAGCATGCAGGCATGAGTAGCGATAATTGGGGTGAGAATCCCCAACGCCGTAAACCCA 1320
P24CDO-S4      GTGAGCATGCAGGCATGAGTAGCGATAATTGGGGTGAGAATCCCCAACGCCGTAAACCCA 1320
UNSW1          GTGAGCATGCAGGCATGAGTAGCGATAATTGGGGTGAGAATCCCCAACGCCGTAAACCCA 1320
UNSW2          GTGAGCATGCAGGCATGAGTAGCGATAATTGGGGTGAGAATCCCCAACGCCGTAAACCCA 1320
UNSW3          GTGAGCATGCAGGCATGAGTAGCGATAATTGGGGTGAGAATCCCCAACGCCGTAAACCCA 1320
UNSWCD         GTGAGCATGCAGGCATGAGTAGCGATAATTGGGGTGAGAATCCCCAACGCCGTAAACCCA 1317
UNSWCS         GTGAGCATGCAGGCATGAGTAGCGATAATTGGGGTGAGAATCCCCAACGCCGTAAACCCA 1320
               ************************************************************
```

Supplementary Figure S2

CON23S_GS1_F

```
ATCC33237        AGGTTTCCTACGCGATGCTCGTCATCGTAGGGTTAGCCGGGTCCTAAGCAAAGTCCGAAA 1379
ATCC51562        AGGTTTCCTACGCGATGCTCGTCATCGTAGGGTTAGCCGGGTCCTAAGCAAAGTCCGAAA 1376
H1O1             AGGTTTCCTACGCGATGCTCGTCATCGTAGGGTTAGCCGGGTCCTAAGCAAAGTCCGAAA 1380
H10O-S1          AGGTTTCCTACGCGATGCTCGTCATCGTAGGGTTAGCCGGGTCCTAAGCAAAGTCCGAAA 1376
H17O-S1          AGGTTTCCTACGCGATGCTCGTCATCGTAGGGTTAGCCGGGTCCTAAGCAAAGTCCGAAA 1380
H21O-S3          AGGTTTCCTACGCGATGCTCGTCATCGTAGGGTTAGCCGGGTCCTAAGCAAAGTCCGAAA 1380
P3UCO1           AGGTTTCCTACGCGATGCTCGTCATCGTAGGGTTAGCCGGGTCCTAAGCAAAGTCCGAAA 1379
P3UCB1           AGGTTTCCTACGCGATGCTCGTCATCGTAGGGTTAGCCGGGTCCTAAGCAAAGTCCGAAA 1379
P9CDO-S1         AGGTTTCCTACGCGATGCTCGTCATCGTAGGGTTAGCCGGGTCCTAAGCAAAGTCCGAAA 1376
P20CDO-S4        AGGTTTCCTACGCGATGCTCGTCATCGTAGGGTTAGCCGGGTCCTAAGCAAAGTCCGAAA 1379
13826            AGGTTTCCTACGCGATGCTCGTCATCGTAGGGTTAGCCGGGTCCTAAGCAAAGTCCGAAA 1380
ATCC51561        AGGTTTCCTACGCGATGCTCGTCATCGTAGGGTTAGCCGGGTCCTAAGCAAAGTCCGAAA 1377
H3O1             AGGTTTCCTACGCGATGCTCGTCATCGTAGGGTTAGCCGGGTCCTAAGCAAAGTCCGAAA 1377
H7O-S1           AGGTTTCCTACGCGATGCTCGTCATCGTAGGGTTAGCCGGGTCCTAAGCAAAGTCCGAAA 1377
H9O-S1           AGGTTTCCTACGCGATGCTCGTCATCGTAGGGTTAGCCGGGTCCTAAGCAAAGTCCGAAA 1376
H9O-S2           AGGTTTCCTACGCGATGCTCGTCATCGTAGGGTTAGCCGGGTCCTAAGCAAAGTCCGAAA 1380
H11O-S1          AGGTTTCCTACGCGATGCTCGTCATCGTAGGGTTAGCCGGGTCCTAAGCAAAGTCCGAAA 1377
H14O-S1          AGGTTTCCTACGCGATGCTCGTCATCGTAGGGTTAGCCGGGTCCTAAGCAAAGTCCGAAA 1379
H16O-S1          AGGTTTCCTACGCGATGCTCGTCATCGTAGGGTTAGCCGGGTCCTAAGCAAAGTCCGAAA 1377
H20O-S1          AGGTTTCCTACGCGATGCTCGTCATCGTAGGGTTAGCCGGGTCCTAAGCAAAGTCCGAAA 1377
H21O-S1          AGGTTTCCTACGCGATGCTCGTCATCGTAGGGTTAGCCGGGTCCTAAGCAAAGTCCGAAA 1380
H21O-S2          AGGTTTCCTACGCGATGCTCGTCATCGTAGGGTTAGCCGGGTCCTAAGCAAAGTCCGAAA 1380
H21O-S5          AGGTTTCCTACGCGATGCTCGTCATCGTAGGGTTAGCCGGGTCCTAAGCAAAGTCCGAAA 1380
H22O-S1          AGGTTTCCTACGCGATGCTCGTCATCGTAGGGTTAGCCGGGTCCTAAGCAAAGTCCGAAA 1380
H23O-S1          AGGTTTCCTACGCGATGCTCGTCATCGTAGGGTTAGCCGGGTCCTAAGCAAAGTCCGAAA 1379
P2CDO3           AGGTTTCCTACGCGATGCTCGTCATCGTAGGGTTAGCCGGGTCCTAAGCAAAGTCCGAAA 1380
P2CDO4           AGGTTTCCTACGCGATGCTCGTCATCGTAGGGTTAGCCGGGTCCTAAGCAAAGTCCGAAA 1380
P2CDO-S6         AGGTTTCCTACGCGATGCTCGTCATCGTAGGGTTAGCCGGGTCCTAAGCAAAGTCCGAAA 1380
P6CDO1           AGGTTTCCTACGCGATGCTCGTCATCGTAGGGTTAGCCGGGTCCTAAGCAAAGTCCGAAA 1377
P12CDO-S1        AGGTTTCCTACGCGATGCTCGTCATCGTAGGGTTAGCCGGGTCCTAAGCAAAGTCCGAAA 1377
P13UCO-S3        AGGTTTCCTACGCGATGCTCGTCATCGTAGGGTTAGCCGGGTCCTAAGCAAAGTCCGAAA 1379
P15UCO-S2        AGGTTTCCTACGCGATGCTCGTCATCGTAGGGTTAGCCGGGTCCTAAGCAAAGTCCGAAA 1380
P16UCO-S1        AGGTTTCCTACGCGATGCTCGTCATCGTAGGGTTAGCCGGGTCCTAAGCAAAGTCCGAAA 1377
P16UCO-S2        AGGTTTCCTACGCGATGCTCGTCATCGTAGGGTTAGCCGGGTCCTAAGCAAAGTCCGAAA 1376
P18CDO-S1        AGGTTTCCTACGCGATGCTCGTCATCGTAGGGTTAGCCGGGTCCTAAGCAAAGTCCGAAA 1377
P20CDO-S1        AGGTTTCCTACGCGATGCTCGTCATCGTAGGGTTAGCCGGGTCCTAAGCAAAGTCCGAAA 1379
P20CDO-S2        AGGTTTCCTACGCGATGCTCGTCATCGTAGGGTTAGCCGGGTCCTAAGCAAAGTCCGAAA 1380
P20CDO-S3        AGGTTTCCTACGCGATGCTCGTCATCGTAGGGTTAGCCGGGTCCTAAGCAAAGTCCGAAA 1379
P21CDO-S1        AGGTTTCCTACGCGATGCTCGTCATCGTAGGGTTAGCCGGGTCCTAAGCAAAGTCCGAAA 1379
P21CDO-S2        AGGTTTCCTACGCGATGCTCGTCATCGTAGGGTTAGCCGGGTCCTAAGCAAAGTCCGAAA 1380
P21CDO-S4        AGGTTTCCTACGCGATGCTCGTCATCGTAGGGTTAGCCGGGTCCTAAGCAAAGTCCGAAA 1380
P24CDO-S2        AGGTTTCCTACGCGATGCTCGTCATCGTAGGGTTAGCCGGGTCCTAAGCAAAGTCCGAAA 1380
P24CDO-S3        AGGTTTCCTACGCGATGCTCGTCATCGTAGGGTTAGCCGGGTCCTAAGCAAAGTCCGAAA 1380
P24CDO-S4        AGGTTTCCTACGCGATGCTCGTCATCGTAGGGTTAGCCGGGTCCTAAGCAAAGTCCGAAA 1380
UNSW1            AGGTTTCCTACGCGATGCTCGTCATCGTAGGGTTAGCCGGGTCCTAAGCAAAGTCCGAAA 1380
UNSW2            AGGTTTCCTACGCGATGCTCGTCATCGTAGGGTTAGCCGGGTCCTAAGCAAAGTCCGAAA 1380
UNSW3            AGGTTTCCTACGCGATGCTCGTCATCGTAGGGTTAGCCGGGTCCTAAGCAAAGTCCGAAA 1380
UNSWCD           AGGTTTCCTACGCGATGCTCGTCATCGTAGGGTTAGCCGGGTCCTAAGCAAAGTCCGAAA 1377
UNSWCS           AGGTTTCCTACGCGATGCTCGTCATCGTAGGGTTAGCCGGGTCCTAAGCAAAGTCCGAAA 1380
                 ************************************************************
```

Supplementary Figure S2

```
ATCC33237       -GGGGTATGCGATGGAAAATTGGTTAATATTCCAATGCCAACTATAATGTGCGATGGAAG 1438
ATCC51562       -GGGGTATGCGATGGAAAATTGGTTAATATTCCAATGCCAACTATAATGTGCGATGGAAG 1435
H1O1            -GGGGTATGCGATGGAAAATTGGTTAATATTCCAATGCCAACTATAATGTGCGATGGAAG 1439
H10O-S1         -GGGGTATGCGATGGAAAATTGGTTAATATTCCAATGCCAACTATAATGTGCGATGGAAG 1435
H17O-S1         -GGGGTATGCGATGGAAAATTGGTTAATATTCCAATGCCAACTATAATGTGCGATGGAAG 1439
H21O-S3         -GGGGTATGCGATGGAAAATTGGTTAATATTCCAATGCCAACTATAATGTGCGATGGAAG 1439
P3UCO1          -GGGGTATGCGATGGAAAATTGGTTAATATTCCAATGCCAACTATAATGTGCGATGGAAG 1438
P3UCB1          -GGGGTATGCGATGGAAAATTGGTTAATATTCCAATGCCAACTATAATGTGCGATGGAAG 1438
P9CDO-S1        -GGGGTATGCGATGGAAAATTGGTTAATATTCCAATGCCAACTATAATGTGCGATGGAAG 1435
P20CDO-S4       -GGGGTATGCGATGGAAAATTGGTTAATATTCCAATGCCAACTATAATGTGCGATGGAAG 1438
13826           -GGGGTATGCGATGGAAAATTGGTTAATATTCCAATGCCAACTATAATGTGCGATGGAAG 1439
ATCC51561       -GGGGTATGCGATGGAAAATTGGTTAATATTCCAATGCCAACTATAATGTGCGATGGAAG 1436
H3O1            -GGGGTATGCGATGGAAAATTGGTTAATATTCCAATGCCAACTATAATGTGCGATGGAAG 1436
H7O-S1          -GGGGTATGCGATGGAAAATTGGTTAATATTCCAATGCCAACTATAATGTGCGATGGAAG 1436
H9O-S1          -GGGGTATGCGATGGAAAATTGGTTAATATTCCAATGCCAACTATAATGTGCGATGGAAG 1435
H9O-S2          -GGGGTATGCGATGGAAAATTGGTTAATATTCCAATGCCAACTATAATGTGCGATGGAAG 1439
H11O-S1         -GGGGTATGCGATGGAAAATTGGTTAATATTCCAATGCCAACTATAATGTGCGATGGAAG 1436
H14O-S1         -GGGGTATGCGATGGAAAATTGGTTAATATTCCAATGCCAACTATAATGTGCGATGGAAG 1438
H16O-S1         -GGGGTATGCGATGGAAAATTGGTTAATATTCCAATGCCAACTATAATGTGCGATGGAAG 1436
H20O-S1         -GGGGTATGCGATGGAAAATTGGTTAATATTCCAATGCCAACTATAATGTGCGATGGAAG 1436
H21O-S1         -GGGGTATGCGATGGAAAATTGGTTAATATTCCAATGCCAACTATAATGTGCGATGGAAG 1439
H21O-S2         -GGGGTATGCGATGGAAAATTGGTTAATATTCCAATGCCAACTATAATGTGCGATGGAAG 1439
H21O-S5         -GGGGTATGCGATGGAAAATTGGTTAATATTCCAATGCCAACTATAATGTGCGATGGAAG 1439
H22O-S1         -GGGGTATGCGATGGAAAATTGGTTAATATTCCAATGCCAACTATAATGTGCGATGGAAG 1439
H23O-S1         -GGGGTATGCGATGGAAAATTGGTTAATATTCCAATGCCAACTATAATGTGCGATGGAAG 1438
P2CDO3          -GGGGTATGCGATGGAAAATTGGTTAATATTCCAATGCCAACTATAATGTGCGATGGAAG 1439
P2CDO4          -GGGGTATGCGATGGAAAATTGGTTAATATTCCAATGCCAACTATAATGTGCGATGGAAG 1439
P2CDO-S6        -GGGGTATGCGATGGAAAATTGGTTAATATTCCAATGCCAACTATAATGTGCGATGGAAG 1439
P6CDO1          -GGGGTATGCGATGGAAAATTGGTTAATATTCCAATGCCAACTATAATGTGCGATGGAAG 1436
P12CDO-S1       -GGGGTATGCGATGGAAAATTGGTTAATATTCCAATGCCAACTATAATGTGCGATGGAAG 1436
P13UCO-S3       -GGGGTATGCGATGGAAAATTGGTTAATATTCCAATGCCAACTATAATGTGCGATGGAAG 1438
P15UCO-S2       -GGGGTATGCGATGGAAAATTGGTTAATATTCCAATGCCAACTATAATGTGCGATGGAAG 1439
P16UCO-S1       -GGGGTATGCGATGGAAAATTGGTTAATATTCCAATGCCAACTATAATGTGCGATGGAAG 1436
P16UCO-S2       -GGGGTATGCGATGGAAAATTGGTTAATATTCCAATGCCAACTATAATGTGCGATGGAAG 1435
P18CDO-S1       -GGGGTATGCGATGGAAAATTGGTTAATATTCCAATGCCAACTATAATGTGCGATGGAAG 1436
P20CDO-S1       -GGGGTATGCGATGGAAAATTGGTTAATATTCCAATGCCAACTATAATGTGCGATGGAAG 1438
P20CDO-S2       -GGGGTATGCGATGGAAAATTGGTTAATATTCCAATGCCAACTATAATGTGCGATGGAAG 1439
P20CDO-S3       -GGGGTATGCGATGGAAAATTGGTTAATATTCCAATGCCAACTATAATGTGCGATGGAAG 1438
P21CDO-S1       -GGGGTATGCGATGGAAAATTGGTTAATATTCCAATGCCAACTATAATGTGCGATGGAAG 1438
P21CDO-S2       -GGGGTATGCGATGGAAAATTGGTTAATATTCCAATGCCAACTATAATGTGCGATGGAAG 1439
P21CDO-S4       -GGGGTATGCGATGGAAAATTGGTTAATATTCCAATGCCAACTATAATGTGCGATGGAAG 1439
P24CDO-S2       -GGGGTATGCGATGGAAAATTGGTTAATATTCCAATGCCAACTATAATGTGCGATGGAAG 1439
P24CDO-S3       -GGGGTATGCGATGGAAAATTGGTTAATATTCCAATGCCAACTATAATGTGCGATGGAAG 1439
P24CDO-S4       -GGGGTATGCGATGGAAAATTGGTTAATATTCCAATGCCAACTATAATGTGCGATGGAAG 1439
UNSW1           -GGGGTATGCGATGGAAAATTGGTTAATATTCCAATGCCAACTATAATGTGCGATGGAAG 1439
UNSW2           -GGGGTATGCGATGGAAAATTGGTTAATATTCCAATGCCAACTATAATGTGCGATGGAAG 1439
UNSW3           -GGGGTATGCGATGGAAAATTGGTTAATATTCCAATGCCAACTATAATGTGCGATGGAAG 1439
UNSWCD          -GGGGTATGCGATGGAAAATTGGTTAATATTCCAATGCCAACTATAATGTGCGATGGAAG 1436
UNSWCS          GGGGGTATGCGATGGAAAATTGGTTAATATTCCAATGCCAACTATAATGTGCGATGGAAG 1440
                 ***********************************************************
```

Supplementary Figure S2

CON23S_GS2_F

```
ATCC33237    GACGCTTAGAGTTAAGCAAGCTAGCGGATGGTAGTGCTAGTCGAAAGGTGTAGGTTAAGA 1498
ATCC51562    GACGCTTAGAGTTAAGCAAGCTAGCGGATGGTAGTGCTAGTCGAAAGGTGTAGGTTAAGA 1495
H1O1         GACGCTTAGAGTTAAGCAAGCTAGCGGATGGTAGTGCTAGTCGAAAGGTGTAGGTTAAGA 1499
H10O-S1      GACGCTTAGAGTTAAGCAAGCTAGCGGATGGTAGTGCTAGTCGAAAGGTGTAGGTTAAGA 1495
H17O-S1      GACGCTTAGAGTTAAGCAAGCTAGCGGATGGTAGTGCTAGTCGAAAGGTGTAGGTTAAGA 1499
H21O-S3      GACGCTTAGAGTTAAGCAAGCTAGCGGATGGTAGTGCTAGTCGAAAGGTGTAGGTTAAGA 1499
P3UCO1       GACGCTTAGAGTTAAGCAAGCTAGCGGATGGTAGTGCTAGTCGAAAGGTGTAGGTTAAGA 1498
P3UCB1       GACGCTTAGAGTTAAGCAAGCTAGCGGATGGTAGTGCTAGTCGAAAGGTGTAGGTTAAGA 1498
P9CDO-S1     GACGCTTAGAGTTAAGCAAGCTAGCGGATGGTAGTGCTAGTCGAAAGGTGTAGGTTAAGA 1495
P20CDO-S4    GACGCTTAGAGTTAAGCAAGCTAGCGGATGGTAGTGCTAGTCGAAAGGTGTAGGTTAAGA 1498
13826        GACGCTTAGAGTTAGAGGAGCCAGCGGATGGTAGTGCTGGTCGAAAGGTGCAGGTTGGAG 1499
ATCC51561    GACGCTTAGAGTTAGAGGAGCCAGCGGATGGTAGTGCTGGTCGAAAGGTGCAGGTTGGAG 1496
H3O1         GACGCTTAGAGTTAGAGGAGCCAGCGGATGGTAGTGCTGGTCGAAAGGTGCAGGTTGGAG 1496
H7O-S1       GACGCTTAGAGTTAGAGGAGCCAGCGGATGGTAGTGCTGGTCGAAAGGTGCAGGTTGGAG 1496
H9O-S1       GACGCTTAGAGTTAGAGGAGCCAGCGGATGGTAGTGCTGGTCGAAAGGTGCAGGTTGGAG 1495
H9O-S2       GACGCTTAGAGTTAGAGGAGCCAGCGGATGGTAGTGCTGGTCGAAAGGTGCAGGTTGGAG 1499
H11O-S1      GACGCTTAGAGTTAGAGGAGCCAGCGGATGGTAGTGCTGGTCGAAAGGTGCAGGTTGGAG 1496
H14O-S1      GACGCTTAGAGTTAGAGGAGCCAGCGGATGGTAGTGCTGGTCGAAAGGTGCAGGTTGGAG 1498
H16O-S1      GACGCTTAGAGTTAGAGGAGCCAGCGGATGGTAGTGCTGGTCGAAAGGTGCAGGTTGGAG 1496
H20O-S1      GACGCTTAGAGTTAGAGGAGCCAGCGGATGGTAGTGCTGGTCGAAAGGTGCAGGTTGGAA 1496
H21O-S1      GACGCTTAGAGTTAGAGGAGCCAGCGGATGGTAGTGCTGGTCGAAAGGTGCAGGTTGGAG 1499
H21O-S2      GACGCTTAGAGTTAGAGGAGCCAGCGGATGGTAGTGCTGGTCGAAAGGTGCAGGTTGGAG 1499
H21O-S5      GACGCTTAGAGTTAGAGGAGCCAGCGGATGGTAGTGCTGGTCGAAAGGTGCAGGTTGGAG 1499
H22O-S1      GACGCTTAGAGTTAGAGGAGCCAGTGGATGGTA-TGCTGGTCGAAAGGCGCAGGTTGGAA 1498
H23O-S1      GACGCTTAGAGTTAGAGGAGCCAGCGGATGGTAGTGCTGGTCGAAAGGTGCAGGTTGGAG 1498
P2CDO3       GACGCTTAGAGTTAGAGGAGCCAGCGGATGGTAGTGCTGGTCGAAAGGTGCAGGTTGGAA 1499
P2CDO4       GACGCTTAGAGTTAGAGGAGCCAGCGGATGGTAGTGCTGGTCGAAAGGTGCAGGTTGGAG 1499
P2CDO-S6     GACGCTTAGAGTTAGAGGAGCCAGCGGATGGTAGTGCTGGTCGAAAGGTGCAGGTTGGAA 1499
P6CDO1       GACGCTTAGAGTTAGAGGAGCCAGCGGATGGTAGTGCTGGTCGAAAGGTGCAGGTTGGAG 1496
P12CDO-S1    GACGCTTAGAGTTAGAGGAGCCAGCGGATGGTAGTGCTGGTCGAAAGGTGCAGGTTGGAG 1496
P13UCO-S3    GACGCTTAGAGTTAGAGGAGCCAGCGGATGGTAGTGCTGGTCGAAAGGTGCAGGTTGGAG 1498
P15UCO-S2    GACGCTTAGAGTTAGAGGAGCCAGCGGATGGTAGTGCTGGTCGAAAGGTGCAGGTTGGAG 1499
P16UCO-S1    GACGCTTAGAGTTAGAGGAGCCAGCGGATGGTAGTGCTGGTCGAAAGGTGCAGGTTGGAG 1496
P16UCO-S2    GACGCTTAGAGTTAGAGGAGCCAGCGGATGGTAGTGCTGGTCGAAAGGTGCAGGTTGGAG 1495
P18CDO-S1    GACGCTTAGAGTTAGAGGAGCCAGCGGATGGTAGTGCTGGTCGAAAGGTGCAGGTTGGAG 1496
P20CDO-S1    GACGCTTAGAGTTAGAGGAGCCAGCGGATGGTAGTGCTGGTCGAAAGGTGCAGGTTGGAG 1498
P20CDO-S2    GACGCTTAGAGTTAGAGGAGCCAGCGGATGGTAGTGCTGGTCGAAAGGTGCAGGTTGGAG 1499
P20CDO-S3    GACGCTTAGAGTTAGAGGAGCCAGCGGATGGTAGTGCTGGTCGAAAGGTGCAGGTTGGAG 1498
P21CDO-S1    GACGCTTAGAGTTAGAGGAGCCAGCGGATGGTAGTGCTGGTCGAAAGGTGCAGGTTGGAG 1498
P21CDO-S2    GACGCTTAGAGTTAGAGGAGCCAGCGGATGGTAGTGCTGGTCGAAAGGTGCAGGTTGGAG 1499
P21CDO-S4    GACGCTTAGAGTTAGAGGAGCCAGCGGATGGTAGTGCTGGTCGAAAGGTGCAGGTTGGAG 1499
P24CDO-S2    GACGCTTAGAGTTAGAGGAGCCAGCGGATGGTAGTGCTGGTCGAAAGGTGCAGGTTGGAG 1499
P24CDO-S3    GACGCTTAGAGTTAGAGGAGCCAGCGGATGGTAGTGCTGGTCGAAAGGTGCAGGTTGGAG 1499
P24CDO-S4    GACGCTTAGAGTTAGAGGAGCCAGCGGATGGTAGTGCTGGTCGAAAGGTGCAGGTTGGAG 1499
UNSW1        GACGCTTAGAGTTAGAGGAGCCAGCGGATGGTAGTGCTGGTCGAAAGGTGCAGGTTGGAG 1499
UNSW2        GACGCTTAGAGTTAGAGGAGCCAGCGGATGGTAGTGCTGGTCGAAAGGTGCAGGTTGGAG 1499
UNSW3        GACGCTTAGAGTTAGAGGAGCCAGCGGATGGTAGTGCTGGTCGAAAGGTGCAGGTTGGAG 1499
UNSWCD       GACGCTTAGAGTTAGAGGAGCCAGCGGATGGTAGTGCTGGTCGAAAGGTGCAGGTTGGAG 1496
UNSWCS       GACGCTTAGAGTTAGAGGAGCCAGCGGATGGTAGTGCTGGTCGAAAGGTGCAGGTTGGAG 1500
             **************    *** ** ******** **** ********* * *****
```

Supplementary Figure S2

CON23S_GS2_R

```
ATCC33237     TCCAGGCAAATCCGGATCTTTTTAAGCCGAGACCCCACAGGCGTTTGAAGTTCTTCGGAA 1558
ATCC51562     TCCAGGCAAATCCGGATCTTTTTAAGCCGAGACCCCATAGGCGTTTGAAGTTCTTCGGAA 1555
H1O1          TCCAGGCAAATCCGGATCTTTTTAAGCCGAGACCCCACAGGCGTTTGAAGTTCTTCGGAA 1559
H10O-S1       TCCAGGCAAATCCGGATCTTTTTAAGCCGAGACCCCACAGGCGTTTGAAGTTCTTCGGAA 1555
H17O-S1       TCCAGGCAAATCCGGATCTTTTTAAGCCGAGACCCCATAGGCGTTTGAAGTTCTTCGGAA 1559
H21O-S3       TCCAGGCAAATCCGGATCTTTTTAAGCCGAGACCCCATAGGCGTTTGAAGTTCTTCGGAA 1559
P3UCO1        TCCAGGCAAATCCGGATCTTTTTAAGCCGAGACCCCACAGGCGTTTGAAGTTCTTCGGAA 1558
P3UCB1        TCCAGGCAAATCCGGATCTTTTTAAGCCGAGACCCCACAGGCGTTTGAAGTTCTTCGGAA 1558
P9CDO-S1      TCCAGGCAAATCCGGATCTTTTTAAGCCGAGATCCCACAGGCGTTTGAAGTTCTTCGGAA 1555
P20CDO-S4     TCCAGGCAAATCCGGATCTTTTTAAGCCGAGACCCCACAGGCGTTTGAAGTTCTTCGGAA 1558
13826         TTCAGGCAAATCCGAACTCCTTTAAGCCGAGGCCTTACAGGTGCGTGAAGTTCTTCGGAA 1559
ATCC51561     TTCAGGCAAATCCGAGCTCCTTTAAGCCGAGGCCTTACAGGTGCGTGAAGTTCTTCGGAA 1556
H3O1          TTCAGGCAAATCCGAACTCCTTTAAGCCGAGGCCTTACAGGTGCGTGAAGTTCTTCGGAA 1556
H7O-S1        TTCAGGCAAATCCGAACTCCTTTAAGCCGAGGCCTTACAGGTGCGTGAAGTTCTTCGGAA 1556
H9O-S1        TTCAGGCAAATCCGAACTCCTTTAAGCCGAGGCCTTATAGGTGCGTGAAGTTCTTCGGAA 1555
H9O-S2        TTCAGGCAAATCCGAGCTCCTTTAAGCCGAGGCCTTACAGGTGCGTGAAGTTCTTCGGAA 1559
H11O-S1       TTCAGGCAAATCCGAACTCCTTTAAGCCGAGGCCTTACAGGTGCGTGAAGTTCTTCGGAA 1556
H14O-S1       TTCAGGCAAATCCGAACTCCTTTAAGCCGAGGCCTTACAGGTGCGTGAAGTTCTTCGGAA 1558
H16O-S1       TTCAGGCAAATCCGAACTCCTTTAAGCCGAGGCCTTACAGGTGCGTGAAGTTCTTCGGAA 1556
H20O-S1       TTCAGGCAAATCCGAATTCCTTTAAGCCGAGGCCTTACAGGTGCGTGAAGTTCTTCGGAA 1556
H21O-S1       TTCAGGCAAATCCGAACTCCTTTAAGCCGAGGCCTTACAGGTACGTGAAATTCTTCGGAA 1559
H21O-S2       TTCAGGCAAATCCGAACTCCTTTAAGCCGAGGCCTTACAGGTGCGTGAAGTTCTTCGGAA 1559
H21O-S5       TTCAGGCAAATCCGAACTCCTTTAAGCCGAGGCCTTACAGGTGCGTGAAGTTCTTCGGAA 1559
H22O-S1       TTCAGGCAAATCCGAATTCCTTTAAGCCGAGGCCCTACAGGTGCGTGAAATTCTTCGGAA 1558
H23O-S1       TTCAGGCAAATCCGAACTCCTTTAATCCGAGGCCTTACAGGTGCGTGAAGTTCTTCGGAA 1558
P2CDO3        TTCAGGCAAATCCGAATTCCTTTAAGCCGAGGCCTTACAGGTGCGTGAAGTTCTTCGGAA 1559
P2CDO4        TTCAGGTAAATCCGAACTCCTTTAAGCCGAGGCCTTACAGGTACGTGAAGTTTTTCGGAA 1559
P2CDO-S6      TTCAGGCAAATCCGAATTCCTTTAAGCCGAGGCCTTACAGGTGCGTGAAGTTCTTCGGAA 1559
P6CDO1        TTCAGGCAAATCCGAACTCCTTTAAGCCGAGGCCTTACAGGTGCGTGAAGTTCTTCGGAA 1556
P12CDO-S1     TTCAGGCAAATCCGAACTCCTTTAAGCCGAGGCCTTACAGGTGCGTGAAGTTCTTCGGAA 1556
P13UCO-S3     TTCAGGCAAATCCGAACTCCTTTAATCCGAGGCCTTACAGGTGCGTGAAGTTCTTCGGAA 1558
P15UCO-S2     TTCAGGCAAATCCGAACTCCTTTAAGCCGAGGCCTTACAGGTGCGTGAAGTTCTTCGGAA 1559
P16UCO-S1     TTCAGGCAAATCCGAACTCCTTTAAGCCGAGGCCTTACAGGTGCGTGAAGTTCTTCGGAA 1556
P16UCO-S2     TTCAGGCAAATCCGAACTCCTTTAATCCGAGGCCTTACAGGTGCGTGAAGTTCTTCGGAA 1555
P18CDO-S1     TTCAGGCAAATCCGAACTCCTTTAAGCCGAGGCCTTACAGGTGCGTGAAGTTCTTCGGAA 1556
P20CDO-S1     TTCAGGCAAATCCGAACTCCTTTAAGCCGAGGCCTTACAGGTGCGTGAAGTTCTTCGGAA 1558
P20CDO-S2     TTCAGGCAAATCCGAACTCCTTTAAGCCGAGGCCTTACAGGTGCGTGAAGTTCTTCGGAA 1559
P20CDO-S3     TTCAGGCAAATCCGAACTCCTTTAAGCCGAGGCCTTACAGGTGCGTGAAGTTCTTCGGAA 1558
P21CDO-S1     TTCAGGCAAATCCGAACTCCTTTAAGCCGAGGCCTTACAGGTGCGTGAAGTTCTTCGGAA 1558
P21CDO-S2     TTCAGGCAAATCCGAACTCCTTTGAGCCGAGGCCTTACAGGTGCGTGAAGTTCTTCGGAA 1559
P21CDO-S4     TTCAGGCAAATCCGAACTCCTTTAAGCCGAGGCCTTATAGGTGCGTGAAGTTCTTCGGAA 1559
P24CDO-S2     TTCAGGCAAATCCGAACTCCTTTAAGCCGAGGCCTTACAGGTGCGTGAAGTTCTTCGGAA 1559
P24CDO-S3     TTCAGGCAAATCCGAACTCCTTTAAGCCGAGGCCTTACAGGTGCGTGAAGTTCTTCGGAA 1559
P24CDO-S4     TTCAGGCAAATCCGAACTCCTTTAAGCCGAGGCCTTACAGGTGCGTGAAGTTCTTCGGAA 1559
UNSW1         TTCAGGCAAATCCGAACTCCTTTAAGCCGAGGCCTTACAGGTGCGTGAAGTTCTTCGGAA 1559
UNSW2         TTCAGGCAAATCCGAACTCCTTTAAGCCGAGGCCTTACAGGTGCGTGAAGTTCTTCGGAA 1559
UNSW3         TTCAGGCAAATCCGAACTCCTTTAAGCCGAGGCCTTACAGGTGCGTGAAGTTCTTCGGAA 1559
UNSWCD        TTCAGGCAAATCCGAACTCCTTTAAGCCGAGGCCTTACAGGTGCGTGAAGTTCTTCGGAA 1556
UNSWCS        TTCAGGCAAATCCGAACTCCTTTAAGCCGAGGCCTTACAGGTGCGTGAAGTTCTTCGGAA 1560
              * **** *******      *** * *****  *  * ***    **** ** *******
```

Supplementary Figure S2

CON1

```
ATCC33237       TGGATAGCGAATTGCCGATACTGTCGAGCCAAGAAAAGTTTCTAAGTTTAGTTATAGTTG 1618
ATCC51562       TGGATAGCGAATTGCCGATACTGTCGAGCCAAGAAAAGTTTCTAAGTTTAGTTATAGTTG 1615
H1O1            TGGATAGCGAATTGCTGATACTGTCGAGCCAAGAAAAGTTTCTAAGTTTAGTTATAGTTG 1619
H10O-S1         TGGATAGCGAATTGCCGATACTGTCGAGCCAAGAAAAGTTTCTAAGTTTAGTTATAGTTG 1615
H17O-S1         TGGATAGCGAATTGCCGATACTGTCGAGCCAAGAAAAGTTTCTAAGTTTAGTTATAGTTG 1619
H21O-S3         TGGATAGCGAATTGCCGATACTGTCGAGCCAAGAAAAGTTTCTAAGTTTAGTTATAGTTG 1619
P3UCO1          TGGATAGCGAATTGCTGATACTGTCGAGCCAAGAAAAGTTTCTAAGTTTAGTTATAGTTG 1618
P3UCB1          TGGATAGCGAATTGCTGATACTGTCGAGCCAAGAAAAGTTTCTAAGTTTAGTTATAGTTG 1618
P9CDO-S1        CGGATAGCGAATTGCCGATACTGTCGAGCCAAGAAAAGTTTCTAAGTTTAGTTATAGTTG 1615
P20CDO-S4       TGGATAGCGAATTGCCGATACTGTCGAGCCAAGAAAAGTTTCTAAGTTTAGTTATAGTTG 1618
13826           TGAATCGTAAATCCTTGATACTGTCGAGCCAAGAAAAGTTTCTAAGTTTAGTTATAGTTG 1619
ATCC51561       TGAATCGTAAATCCTTGATACTGTCGAGCCAAGAAAAGTTTCTAAGTTTAGTTATAGTTG 1616
H3O1            TGAATCGTAAATCCTTGATACTGTCGAGCCAAGAAAAGTTTCTAAGTTTAGTTATAGTTG 1616
H7O-S1          TGAATCGTAAATCCTTGATACTGTCGAGCCAAGAAAAGTTTCTAAGTTTAGTTATAGTTG 1616
H9O-S1          TGAATCGTAAATCCTTGATACTGTCGAGCCAAGAAAAGTTTCTAAGTTTAGTTATAGTTG 1615
H9O-S2          TGAATCGTAAATCCTTGATACTGTCGAGCCAAGAAAAGTTTCTAAGTTTAGTTATAGTTG 1619
H11O-S1         TGAATCGTAAATCCTTGATACTGTCGAGCCAAGAAAAGTTTCTAAGTTTAGTTATAGTTG 1616
H14O-S1         TGAATCGTAAATCCTTGATACTGTCGAGCCAAGAAAAGTTTCTAAGTTTAGTTATAGTTG 1618
H16O-S1         TGAATCGTAAATCCTTTATACTGTCGAGCCAAGAAAAGTTTCTAAGTTTAGTTATAGTTG 1616
H20O-S1         TGAATCGTAAATCCTTGATACTGTCGAGCCAAGAAAAGTTTCTAAGTTTAGTTATAGTTG 1616
H21O-S1         TGAATCGTAAATCCTTGATACTGTCGAGCCAAGAAAAGTTTCTAAGTTTAGTTATAGTTG 1619
H21O-S2         TGAATCGTAAATCCTTGATACTGTCGAGCCAAGAAAAGTTTCTAAGTTTAGTTATAGTTG 1619
H21O-S5         TGAATCGTAAATCCTTGATACTGTCGAGCCAAGAAAAGTTTCTAAGTTTAGTTATAGTTG 1619
H22O-S1         TAGATCGTATATTCTTGATACTGTCGAGCCAAGAAAAGTTTCTAAGTTTAGTTATAGTTG 1618
H23O-S1         TGAATCGTAAATCCTTGATACTGTCGAGCCAAGAAAAGTTTCTAAGTTTAGTTATAGTTG 1618
P2CDO3          TGAATCGTAAATCCTTGATACTGTCGAGCCAAGAAAAGTTTCTAAGTTTAGTTATAGTTG 1619
P2CDO4          TGAATCGTAAATCCTTGATACTGTCGAGCCAAGAAAAGTTTCTAAGTTTAGTTATAGTTG 1619
P2CDO-S6        TGAATCGTAAATCCTTGATACTGTCGAGCCAAGAAAAGTTTCTAAGTTTAGTTATAGTTG 1619
P6CDO1          TGAATCGTAAATCCTTGATACTGTCGAGCCAAGAAAAGTTTCTAAGTTTAGTTATAGTTG 1616
P12CDO-S1       TGAATCGTAAATCCTTGATACTGTCGAGCCAAGAAAAGTTTCTAAGTTTAGTTATAGTTG 1616
P13UCO-S3       TGAATCGTAAATCCTTGATACTGTCGAGCCAAGAAAAGTTTCTAAGTTTAGTTATAGTTG 1618
P15UCO-S2       TGAATCGTAAATCCTTTATACTGTCGAGCCAAGAAAAGTTTCTAAGTTTAGTTATAGTTG 1619
P16UCO-S1       TGAATCGTAAATCCTTTATACTGTCGAGCCAAGAAAAGTTTCTAAGTTTAGTTATAGTTG 1616
P16UCO-S2       TGAATCGTAAATCCTTGATACTGTCGAGCCAAGAAAAGTTTCTAAGTTTAGTTATAGTTG 1615
P18CDO-S1       TGAATCGTAAATCCTTGATACTGTCGAGCCAAGAAAAGTTTCTAAGTTTAGTTATAGTTG 1616
P20CDO-S1       TGAATCGTAAATCCTTGATACTGTCGAGCCAAGAAAAGTTTCTAAGTTTAGTTATAGTTG 1618
P20CDO-S2       TGAATCGTAAATCCTTGATACTGTCGAGCCAAGAAAAGTTTCTAAGTTTAGTTATAGTTG 1619
P20CDO-S3       TGAATCGTAAATCCTTGATACTGTCGAGCCAAGAAAAGTTTCTAAGTTTAGTTATAGTTG 1618
P21CDO-S1       TGAATCGTAAATCCTTGATACTGTCGAGCCAAGAAAAGTTTCTAAGTTTAGTTATAGTTG 1618
P21CDO-S2       TGAATCGTAAATCCTTGATACTGTCGAGCCAAGAAAAGTTTCTAAGTTTAGTTATAGTTG 1619
P21CDO-S4       TGAATCGTAAATCCTTGATACTGTCGAGCCAAGAAAAGTTTCTAAGTTTAGTTATAGTTG 1619
P24CDO-S2       TGAATCGTAAATCCTTGATACTGTCGAGCCAAGAAAAGTTTCTAAGTTTAGTTATAGTTG 1619
P24CDO-S3       TGAATCGTAAATCCTTGATACTGTCGAGCCAAGAAAAGTTTCTAAGTTTAGTTATAGTTG 1619
P24CDO-S4       TGAATCGTAAATCCTTGATACTGTCGAGCCAAGAAAAGTTTCTAAGTTTAGTTATAGTTG 1619
UNSW1           TGAATCGTAAATCCTTGATACTGTCGAGCCAAGAAAAGTTTCTAAGTTTAGTTATAGTTG 1619
UNSW2           TGAATCGTAAATCCTTTATACTGTCGAGCCAAGAAAAGTTTCTAAGTTTAGTTATAGTTG 1619
UNSW3           TGAATCGTAAATCCTTGATACTGTCGAGCCAAGAAAAGTTTCTAAGTTTAGTTATAGTTG 1619
UNSWCD          TGAATCGTAAATCCTTGATACTGTCGAGCCAAGAAAAGTTTCTAAGTTTAGTTATAGTTG 1616
UNSWCS          TGAATCGTAAATCCTTGATACTGTCGAGCCAAGAAAAGTTTCTAAGTTTAGTTATAGTTG 1620
                   ** *   **     *******************************************
```

Supplementary Figure S2

```
ATCC33237      CCCGTACCGTAAACCGACACAGGTGGGTGGGATGAGTATTCTAAGGCGCGTGGAAGAACT 1678
ATCC51562      CCCGTACCGTAAACCGACACAGGTGGGTGGGATGAGTATTCTAAGGCGCGTGGAAGAACT 1675
H1O1           CCCGTACCGTAAACCGACACAGGTGGGTGGGATGAGTATTCTAAGGCGCGTGGAAGAACT 1679
H10O-S1        CCCGTACCGTAAACCGACACAGGTGGGTGGGATGAGTATTCTAAGGCGCGTGGAAGAACT 1675
H17O-S1        CCCGTACCGTAAACCGACACAGGTGGGTGGGATGAGTATTCTAAGGCGCGTGGAAGAACT 1679
H21O-S3        CCCGTACCGTAAACCGACACAGGTGGGTGGGATGAGTATTCTAAGGCGCGTGGAAGAACT 1679
P3UCO1         CCCGTACCGTAAACCGACACAGGTGGGTGGGATGAGTATTCTAAGGCGCGTGGAAGAACT 1678
P3UCB1         CCCGTACCGTAAACCGACACAGGTGGGTGGGATGAGTATTCTAAGGCGCGTGGAAGAACT 1678
P9CDO-S1       CCCGTACCGTAAACCGACACAGGTGGGTGGGATGAGTATTCTAAGGCGCGTGGAAGAACT 1675
P20CDO-S4      CCCGTACCGTAAACCGACACAGGTGGGTGGGATGAGTATTCTAAGGCGCGTGGAAGAACT 1678
13826          CCCGTACCGTAAACCGACACAGGTGGGTGGGATGAGTATTCTAAGGCGCGTGGAAGAACT 1679
ATCC51561      CCCGTACCGTAAACCGACACAGGTGGGTGGGATGAGTATTCTAAGGCGCGTGGAAGAACT 1676
H3O1           CCCGTACCGTAAACCGACACAGGTGGGTGGGATGAGTATTCTAAGGCGCGTGGAAGAACT 1676
H7O-S1         CCCGTACCGTAAACCGACACAGGTGGGTGGGATGAGTATTCTAAGGCGCGTGGAAGAACT 1676
H9O-S1         CCCGTACCGTAAACCGACACAGGTGGGTGGGATGAGTATTCTAAGGCGCGTGGAAGAACT 1675
H9O-S2         CCCGTACCGTAAACCGACACAGGTGGGTGGGATGAGTATTCTAAGGCGCGTGGAAGAACT 1679
H11O-S1        CCCGTACCGTAAACCGACACAGGTGGGTGGGATGAGTATTCTAAGGCGCGTGGAAGAACT 1676
H14O-S1        CCCGTACCGTAAACCGACACAGGTGGGTGGGATGAGTATTCTAAGGCGCGTGGAAGAACT 1678
H16O-S1        CCCGTACCGTAAACCGACACAGGTGGGTGGGATGAGTATTCTAAGGCGCGTGGAAGAACT 1676
H20O-S1        CCCGTACCGTAAACCGACACAGGTGGGTGGGATGAGTATTCTAAGGCGCGTGGAAGAACT 1676
H21O-S1        CCCGTACCGTAAACCGACACAGGTGGGTGGGATGAGTATTCTAAGGCGCGTGGAAGAACT 1679
H21O-S2        CCCGTACCGTAAACCGACACAGGTGGGTGGGATGAGTATTCTAAGGCGCGTGGAAGAACT 1679
H21O-S5        CCCGTACCGTAAACCGACACAGGTGGGTGGGATGAGTATTCTAAGGCGCGTGGAAGAACT 1679
H22O-S1        CCCGTACCGTAAACCGACACAGGTGGGTGGGATGAGTATTCTAAGGCGCGTGGAAGAACT 1678
H23O-S1        CCCGTACCGTAAACCGACACAGGTGGGTGGGATGAGTATTCTAAGGCGCGTGGAAGAACT 1678
P2CDO3         CCCGTACCATAAACCGACACAGGTGGGTGGGATGAGTATTCTAAGGCGCGTGGAAGAACT 1679
P2CDO4         CCCGTACCGTAAACCGACACAGGTGGGTGGGATGAGTATTCTAAGGCGCGTGGAAGAACT 1679
P2CDO-S6       CCCGTACCATAAACCGACACAGGTGGGTGGGATGAGTATTCTAAGGCGCGTGGAAGAACT 1679
P6CDO1         CCCGTACCGTAAACCGACACAGGTGGGTGGGATGAGTATTCTAAGGCGCGTGGAAGAACT 1676
P12CDO-S1      CCCGTACCGTAAACCGACACAGGTGGGTGGGATGAGTATTCTAAGGCGCGTGGAAGAACT 1676
P13UCO-S3      CCCGTACCGTAAACCGACACAGGTGGGTGGGATGAGTATTCTAAGGCGCGTGGAAGAACT 1678
P15UCO-S2      CCCGTACCGTAAACCGACACAGGTGGGTGGGATGAGTATTCTAAGGCGCGTGGAAGAACT 1679
P16UCO-S1      CCCGTACCGTAAACCGACACAGGTGGGTGGGATGAGTATTCTAAGGCGCGTGGAAGAACT 1676
P16UCO-S2      CCCGTACCGTAAACCGACACAGGTGGGTGGGATGAGTATTCTAAGGCGCGTGGAAGAACT 1675
P18CDO-S1      CCCGTACCGTAAACCGACACAGGTGGGTGGGATGAGTATTCTAAGGCGCGTGGAAGAACT 1676
P20CDO-S1      CCCGTACCGTAAACCGACACAGGTGGGTGGGATGAGTATTCTAAGGCGCGTGGAAGAACT 1678
P20CDO-S2      CCCGTACCGTAAACCGACACAGGTGGGTGGGATGAGTATTCTAAGGCGCGTGGAAGAACT 1679
P20CDO-S3      CCCGTACCGTAAACCGACACAGGTGGGTGGGATGAGTATTCTAAGGCGCGTGGAAGAACT 1678
P21CDO-S1      CCCGTACCGTAAACCGACACAGGTGGGTGGGATGAGTATTCTAAGGCGCGTGGAAGAACT 1678
P21CDO-S2      CCCGTACCGTAAACCGACACAGGTGGGTGGGATGAGTATTCTAAGGCGCGTGGAAGAACT 1679
P21CDO-S4      CCCGTACCGTAAACCGACACAGGTGGGTGGGATGAGTATTCTAAGGCGCGTGGAAGAACT 1679
P24CDO-S2      CCCGTACCGTAAACCGACACAGGTGGGTGGGATGAGTATTCTAAGGCGCGTGGAAGAACT 1679
P24CDO-S3      CCCGTACCGTAAACCGACACAGGTGGGTGGGATGAGTATTCTAAGGCGCGTGGAAGAACT 1679
P24CDO-S4      CCCGTACCGTAAACCGACACAGGTGGGTGGGATGAGTATTCTAAGGCGCGTGGAAGAACT 1679
UNSW1          CCCGTACCGTAAACCGACACAGGTGGGTGGGATGAGTATTCTAAGGCGCGTGGAAGAACT 1679
UNSW2          CCCGTACCGTAAACCGACACAGGTGGGTGGGATGAGTATTCTAAGGCGCGTGGAAGAACT 1679
UNSW3          CCCGTACCGTAAACCGACACAGGTGGGTGGGATGAGTATTCTAAGGCGCGTGGAAGAACT 1679
UNSWCD         CCCGTACCGTAAACCGACACAGGTGGGTGGGATGAGTATTCTAAGGCGCGTGGAAGAACT 1676
UNSWCS         CCCGTACCGTAAACCGACACAGGTGGGTGGGATGAGTATTCTAAGGCGCGTGGAAGAACT 1680
               ******** ***************************************************
```

Supplementary Figure S2

```
ATCC33237      CTCTTCAAGGAACTCTGCAAAATAGCACCGTATCTTCGGTATAAGGTGTGCCTAACTTTG 1738
ATCC51562      CTCTTCAAGGAACTCTGCAAAATAGCACCGTATCTTCGGTATAAGGTGTGCCTAACTTTG 1735
H1O1           CTCTTCAAGGAACTCTGCAAAATAGCACCGTATCTTCGGTATAAGGTGTGCCTAACTTTG 1739
H10O-S1        CTCTTCAAGGAACTCTGCAAAATAGCACCGTATCTTCGGTATAAGGTGTGCCTAACTTTG 1735
H17O-S1        CTCTTCAAGGAACTCTGCAAAATAGCACCGTATCTTCGGTATAAGGTGTGCCTAACTTTG 1739
H21O-S3        CTCTTCAAGGAACTCTGCAAAATAGCACCGTATCTTCGGTATAAGGTGTGCCTAACTTTG 1739
P3UCO1         CTCTTCAAGGAACTCTGCAAAATAGCACCGTATCTTCGGTATAAGGTGTGCCTAACTTTG 1738
P3UCB1         CTCTTCAAGGAACTCTGCAAAATAGCACCGTATCTTCGGTATAAGGTGTGCCTAACTTTG 1738
P9CDO-S1       CTCTTCAAGGAACTCTGCAAAATAGCACCGTATCTTCGGTATAAGGTGTGCCTAACTTTG 1735
P20CDO-S4      CTCTTCAAGGAACTCTGCAAAATAGCACCGTATCTTCGGTATAAGGTGTGCCTAACTTTG 1738
13826          CTCTTCAAGGAACTCTGCAAAATAGCACCGTATCTTCGGTATAAGGTGTGCCTAACTTTG 1739
ATCC51561      CTCTTCAAGGAACTCTGCAAAATAGCACCGTATCTTCGGTATAAGGTGTGCCTAACTTTG 1736
H3O1           CTCTTCAAGGAACTCTGCAAAATAGCACCGTATCTTCGGTATAAGGTGTGCCTAACTTTG 1736
H7O-S1         CTCTTCAAGGAACTCTGCAAAATAGCACCGTATCTTCGGTATAAGGTGTGCCTAACTTTG 1736
H9O-S1         CTCTTCAAGGAACTCTGCAAAATAGCACCGTATCTTCGGTATAAGGTGTGCCTAACTTTG 1735
H9O-S2         CTCTTCAAGGAACTCTGCAAAATAGCACCGTATCTTCGGTATAAGGTGTGCCTAACTTTG 1739
H11O-S1        CTCTTCAAGGAACTCTGCAAAATAGCACCGTATCTTCGGTATAAGGTGTGCCTAACTTTG 1736
H14O-S1        CTCTTCAAGGAACTCTGCAAAATAGCACCGTATCTTCGGTATAAGGTGTGCCTAACTTTG 1738
H16O-S1        CTCTTCAAGGAACTCTGCAAAATAGCACCGTATCTTCGGTATAAGGTGTGCCTAACTTTG 1736
H20O-S1        CTCTTCAAGGAACTCTGCAAAATAGCACCGTATCTTCGGTATAAGGTGTGCCTAACTTTG 1736
H21O-S1        CTCTTCAAGGAACTCTGCAAAATAGCACCGTATCTTCGGTATAAGGTGTGCCTAACTTTG 1739
H21O-S2        CTCTTCAAGGAACTCTGCAAAATAGCACCGTATCTTCGGTATAAGGTGTGCCTAACTTTG 1739
H21O-S5        CTCTTCAAGGAACTCTGCAAAATAGCACCGTATCTTCGGTATAAGGTGTGCCTAACTTTG 1739
H22O-S1        CTCTTCAAGGAACTCTGCAAAATAGCACCGTATCTTCGGTATAAGGTGTGCCTAACTTTG 1738
H23O-S1        CTCTTCAAGGAACTCTGCAAAATAGCACCGTATCTTCGGTATAAGGTGTGCCTAACTTTG 1738
P2CDO3         CTCTTCAAGGAACTCTGCAAAATAGCACCGTATCTTCGGTATAAGGTGTGCCTAACTTTG 1739
P2CDO4         CTCTTCAAGGAACTCTGCAAAATAGCACCGTATCTTCGGTATAAGGTGTGCCTAACTTTG 1739
P2CDO-S6       CTCTTCAAGGAACTCTGCAAAATAGCACCGTATCTTCGGTATAAGGTGTGCCTAACTTTG 1739
P6CDO1         CTCTTCAAGGAACTCTGCAAAATAGCACCGTATCTTCGGTATAAGGTGTGCCTAACTTTG 1736
P12CDO-S1      CTCTTCAAGGAACTCTGCAAAATAGCACCGTATCTTCGGTATAAGGTGTGCCTAACTTTG 1736
P13UCO-S3      CTCTTCAAGGAACTCTGCAAAATAGCACCGTATCTTCGGTATAAGGTGTGCCTAACTTTG 1738
P15UCO-S2      CTCTTCAAGGAACTCTGCAAAATAGCACCGTATCTTCGGTATAAGGTGTGCCTAACTTTG 1739
P16UCO-S1      CTCTTCAAGGAACTCTGCAAAATAGCACCGTATCTTCGGTATAAGGTGTGCCTAACTTTG 1736
P16UCO-S2      CTCTTCAAGGAACTCTGCAAAATAGCACCGTATCTTCGGTATAAGGTGTGCCTAACTTTG 1735
P18CDO-S1      CTCTTCAAGGAACTCTGCAAAATAGCACCGTATCTTCGGTATAAGGTGTGCCTAACTTTG 1736
P20CDO-S1      CTCTTCAAGGAACTCTGCAAAATAGCACCGTATCTTCGGTATAAGGTGTGCCTAACTTTG 1738
P20CDO-S2      CTCTTCAAGGAACTCTGCAAAATAGCACCGTATCTTCGGTATAAGGTGTGCCTAACTTTG 1739
P20CDO-S3      CTCTTCAAGGAACTCTGCAAAATAGCACCGTATCTTCGGTATAAGGTGTGCCTAACTTTG 1738
P21CDO-S1      CTCTTCAAGGAACTCTGCAAAATAGCACCGTATCTTCGGTATAAGGTGTGCCTAACTTTG 1738
P21CDO-S2      CTCTTCAAGGAACTCTGCAAAATAGCACCGTATCTTCGGTATAAGGTGTGCCTAACTTTG 1739
P21CDO-S4      CTCTTCAAGGAACTCTGCAAAATAGCACCGTATCTTCGGTATAAGGTGTGCCTAACTTTG 1739
P24CDO-S2      CTCTTCAAGGAACTCTGCAAAATAGCACCGTATCTTCGGTATAAGGTGTGCCTAACTTTG 1739
P24CDO-S3      CTCTTCAAGGAACTCTGCAAAATAGCACCGTATCTTCGGTATAAGGTGTGCCTAACTTTG 1739
P24CDO-S4      CTCTTCAAGGAACTCTGCAAAATAGCACCGTATCTTCGGTATAAGGTGTGCCTAACTTTG 1739
UNSW1          CTCTTCAAGGAACTCTGCAAAATAGCACCGTATCTTCGGTATAAGGTGTGCCTAACTTTG 1739
UNSW2          CTCTTCAAGGAACTCTGCAAAATAGCACCGTATCTTCGGTATAAGGTGTGCCTAACTTTG 1739
UNSW3          CTCTTCAAGGAACTCTGCAAAATAGCACCGTATCTTCGGTATAAGGTGTGCCTAACTTTG 1739
UNSWCD         CTCTTCAAGGAACTCTGCAAAATAGCACCGTATCTTCGGTATAAGGTGTGCCTAACTTTG 1736
UNSWCS         CTCTTCAAGGAACTCTGCAAAATAGCACCGTATCTTCGGTATAAGGTGTGCCTAACTTTG 1740
               ************************************************************
```

Supplementary Figure S2

```
ATCC33237       TGAAGGATTTACTCCGTAAGCATTGAAGGTTACAACAAAGAGTCCCTCCCGACTGTTTAC 1798
ATCC51562       TGAAGGATTTACTCCGTAAGCATTGAAGGTTACAACAAAGAGTCCCTCCCGACTGTTTAC 1795
H1O1            TGAAGGATTTACTCCGTAAGCATTGAAGGTTACAACAAAGAGTCCCTCCCGACTGTTTAC 1799
H10O-S1         TGAAGGATTTACTCCGTAAGCATTGAAGGTTACAACAAAGAGTCCCTCCCGACTGTTTAC 1795
H17O-S1         TTAAGGATTTACTCCGTAAGCATTGAAGGTTACAACAAAGAGTCCCTCCCGACTGTTTAC 1799
H21O-S3         TTAAGGATTTACTCCGTAAGCATTGAAGGTTACAACAAAGAGTCCCTCCCGACTGTTTAC 1799
P3UCO1          TGAAGGATTTACTCCGTAAGCATTGAAGGTTACAACAAAGAGTCCCTCCCGACTGTTTAC 1798
P3UCB1          TGAAGGATTTACTCCGTAAGCATTGAAGGTTACAACAAAGAGTCCCTCCCGACTGTTTAC 1798
P9CDO-S1        TGAAGGATTTACTCCGTAAGCATTGAAGGTTACAACAAAGAGTCCCTCCCGACTGTTTAC 1795
P20CDO-S4       TGAAGGATTTACTCCGTAAGCATTGAAGGTTACAACAAAGAGTCCCTCCCGACTGTTTAC 1798
13826           TGAAGGATTTACTCCGTAAGCATTGAAGGTTACAACAAAGAGTCCCTCCCGACTGTTTAC 1799
ATCC51561       TGAAGGATTTACTCCGTAAGCATTGAAGGTTACAACAAAGAGTCCCTCCCGACTGTTTAC 1796
H3O1            TGAAGGATTTACTCCGTAAGCATTGAAGGTTACAACAAAGAGTCCCTCCCGACTGTTTAC 1796
H7O-S1          TGAAGGATTTACTCCGTAAGCATTGAAGGTTACAACAAAGAGTCCCTCCCGACTGTTTAC 1796
H9O-S1          TGAAGGATTTACTCCGTAAGCATTGAAGGTTACAACAAAGAGTCCCTCCCGACTGTTTAC 1795
H9O-S2          TGAAGGATTTACTCCGTAAGCATTGAAGGTTACAACAAAGAGTCCCTCCCGACTGTTTAC 1799
H11O-S1         TGAAGGATTTACTCCGTAAGCATTGAAGGTTACAACAAAGAGTCCCTCCCGACTGTTTAC 1796
H14O-S1         TGAAGGATTTACTCCGTAAGCATTGAAGGTTACAACAAAGAGTCCCTCCCGACTGTTTAC 1798
H16O-S1         TGAAGGATTTACTCCGTAAGCATTGAAGGTTACAACAAAGAGTCCCTCCCGACTGTTTAC 1796
H20O-S1         TGAAGGATTTACTCCGTAAGCATTGAAGGTTACAACAAAGAGTCCCTCCCGACTGTTTAC 1796
H21O-S1         TGAAGGATTTACTCCGTAAGCATTGAAGGTTACAACAAAGAGTCCCTCCCGACTGTTTAC 1799
H21O-S2         TGAAGGATTTACTCCGTAAGCATTGAAGGTTACAACAAAGAGTCCCTCCCGACTGTTTAC 1799
H21O-S5         TGAAGGATTTACTCCGTAAGCATTGAAGGTTACAACAAAGAGTCCCTCCCGACTGTTTAC 1799
H22O-S1         TGAAGGATTTACTCCGTAAGCATTGAAGGTTACAACAAAGAGTCCCTCCCGACTGTTTAC 1798
H23O-S1         TGAAGGATTTACTCCGTAAGCATTGAAGGTTACAACAAAGAGTCCCTCCCGACTGTTTAC 1798
P2CDO3          TGAAGGATTTACTCCGTAAGCATTGAAGGTTACAACAAAGAGTCCCTCCCGACTGTTTAC 1799
P2CDO4          TGAAGGATTTACTCCGTAAGCATTGAAGGTTACAACAAAGAGTCCCTCCCGACTGTTTAC 1799
P2CDO-S6        TGAAGGATTTACTCCGTAAGCATTGAAGGTTACAACAAAGAGTCCCTCCCGACTGTTTAC 1799
P6CDO1          TGAAGGATTTACTCCGTAAGCATTGAAGGTTACAACAAAGAGTCCCTCCCGACTGTTTAC 1796
P12CDO-S1       TGAAGGATTTACTCCGTAAGCATTGAAGGTTACAACAAAGAGTCCCTCCCGACTGTTTAC 1796
P13UCO-S3       TGAAGGATTTACTCCGTAAGCATTGAAGGTTACAACAAAGAGTCCCTCCCGACTGTTTAC 1798
P15UCO-S2       TGAAGGATTTACTCCGTAAGCATTGAAGGTTACAACAAAGAGTCCCTCCCGACTGTTTAC 1799
P16UCO-S1       TGAAGGATTTACTCCGTAAGCATTGAAGGTTACAACAAAGAGTCCCTCCCGACTGTTTAC 1796
P16UCO-S2       TGAAGGATTTACTCCGTAAGCATTGAAGGTTACAACAAAGAGTCCCTCCCGACTGTTTAC 1795
P18CDO-S1       TGAAGGATTTACTCCGTAAGCATTGAAGGTTACAACAAAGAGTCCCTCCCGACTGTTTAC 1796
P20CDO-S1       TGAAGGATTTACTCCGTAAGCATTGAAGGTTACAACAAAGAGTCCCTCCCGACTGTTTAC 1798
P20CDO-S2       TGAAGGATTTACTCCGTAAGCATTGAAGGTTACAACAAAGAGTCCCTCCCGACTGTTTAC 1799
P20CDO-S3       TGAAGGATTTACTCCGTAAGCATTGAAGGTTACAACAAAGAGTCCCTCCCGACTGTTTAC 1798
P21CDO-S1       TGAAGGATTTACTCCGTAAGCATTGAAGGTTACAACAAAGAGTCCCTCCCGACTGTTTAC 1798
P21CDO-S2       TGAAGGATTTACTCCATAAGCATTGAAGGTTACAACAAAGAGTCCCTCCCGACTGTTTAC 1799
P21CDO-S4       TGAAGGATTTACTCCGTAAGCATTGAAGGTTACAACAAAGAGTCCCTCCCGACTGTTTAC 1799
P24CDO-S2       TGAAGGATTTACTCCATAAGCATTGAAGGTTACAACAAAGAGTCCCTCCCGACTGTTTAC 1799
P24CDO-S3       TGAAGGATTTACTCCATAAGCATTGAAGGTTACAACAAAGAGTCCCTCCCGACTGTTTAC 1799
P24CDO-S4       TGAAGGATTTACTCCATAAGCATTGAAGGTTACAACAAAGAGTCCCTCCCGACTGTTTAC 1799
UNSW1           TGAAGGATTTACTCCGTAAGCATTGAAGGTTACAACAAAGAGTCCCTCCCGACTGTTTAC 1799
UNSW2           TGAAGGATTTACTCCGTAAGCATTGAAGGTTACAACAAAGAGTCCCTCCCGACTGTTTAC 1799
UNSW3           TGAAGGATTTACTCCGTAAGCATTGAAGGTTACAACAAAGAGTCCCTCCCGACTGTTTAC 1799
UNSWCD          TGAAGGATTTACTCCGTAAGCATTGAAGGTTACAACAAAGAGTCCCTCCCGACTGTTTAC 1796
UNSWCS          TGAAGGATTTACTCCGTAAGCATTGAAGGTTACAACAAAGAGTCCCTCCCGACTGTTTAC 1800
                * ************* ********************************************
```

Supplementary Figure S2

```
ATCC33237        CAAAAACACAGCACTCTGCTAACTCGTAAGAGGATGTATAGGGTGTGACGCCTGCCCGGT 1858
ATCC51562        CAAAAACACAGCACTCTGCTAACTCGTAAGAGGATGTATAGGGTGTGACGCCTGCCCGGT 1855
H1O1             CAAAAACACAGCACTCTGCTAACTCGTAAGAGGATGTATAGGGTGTGACGCCTGCCCGGT 1859
H10O-S1          CAAAAACACAGCACTCTGCTAACTCGTAAGAGGATGTATAGGGTGTGACGCCTGCCCGGT 1855
H17O-S1          CAAAAACACAGCACTCTGCTAACTCGTAAGAGGATGTATAGGGTGTGACGCCTGCCCGGT 1859
H21O-S3          CAAAAACACAGCACTCTGCTAACTCGTAAGAGGATGTATAGGGTGTGACGCCTGCCCGGT 1859
P3UCO1           CAAAAACACAGCACTCTGCTAACTCGTAAGAGGATGTATAGGGTGTGACGCCTGCCCGGT 1858
P3UCB1           CAAAAACACAGCACTCTGCTAACTCGTAAGAGGATGTATAGGGTGTGACGCCTGCCCGGT 1858
P9CDO-S1         CAAAAACACAGCACTCTGCTAACTCGTAAGAGGATGTATAGGGTGTGACGCCTGCCCGGT 1855
P20CDO-S4        CAAAAACACAGCACTCTGCTAACTCGTAAGAGGATGTATAGGGTGTGACGCCTGCCCGGT 1858
13826            CAAAAACACAGCACTCTGCTAACTCGTAAGAGGATGTATAGGGTGTGACGCCTGCCCGGT 1859
ATCC51561        CAAAAACACAGCACTCTGCTAACTCGTAAGAGGATGTATAGGGTGTGACGCCTGCCCGGT 1856
H3O1             CAAAAACACAGCACTCTGCTAACTCGTAAGAGGATGTATAGGGTGTGACGCCTGCCCGGT 1856
H7O-S1           CAAAAACACAGCACTCTGCTAACTCGTAAGAGGATGTATAGGGTGTGACGCCTGCCCGGT 1856
H9O-S1           CAAAAACACAGCACTCTGCTAACTCGTAAGAGGATGTATAGGGTGTGACGCCTGCCCGGT 1855
H9O-S2           CAAAAACACAGCACTCTGCTAACTCGTAAGAGGATGTATAGGGTGTGACGCCTGCCCGGT 1859
H11O-S1          CAAAAACACAGCACTCTGCTAACTCGTAAGAGGATGTATAGGGTGTGACGCCTGCCCGGT 1856
H14O-S1          CAAAAACACAGCACTCTGCTAACTCGTAAGAGGATGTATAGGGTGTGACGCCTGCCCGGT 1858
H16O-S1          CAAAAACACAGCACTCTGCTAACTCGTAAGAGGATGTATAGGGTGTGACGCCTGCCCGGT 1856
H20O-S1          CAAAAACACAGCACTCTGCTAACTCGTAAGAGGATGTATAGGGTGTGACGCCTGCCCGGT 1856
H21O-S1          CAAAAACACAGCACTCTGCTAACTCGTAAGAGGATGTATAGGGTGTGACGCCTGCCCGGT 1859
H21O-S2          CAAAAACACAGCACTCTGCTAACTCGTAAGAGGATGTATAGGGTGTGACGCCTGCCCGGT 1859
H21O-S5          CAAAAACACAGCACTCTGCTAACTCGTAAGAGGATGTATAGGGTGTGACGCCTGCCCGGT 1859
H22O-S1          CAAAAACACAGCACTCTGCTAACTCGTAAGAGGATGTATAGGGTGTGACGCCTGCCCGGT 1858
H23O-S1          CAAAAACACAGCACTCTGCTAACTCGTAAGAGGATGTATAGGGTGTGACGCCTGCCCGGT 1858
P2CDO3           CAAAAACACAGCACTCTGCTAACTCGTAAGAGGATGTATAGGGTGTGACGCCTGCCCGGT 1859
P2CDO4           CAAAAACACAGCACTCTGCTAACTCGTAAGAGGATGTATAGGGTGTGACGCCTGCCCGGT 1859
P2CDO-S6         CAAAAACACAGCACTCTGCTAACTCGTAAGAGGATGTATAGGGTGTGACGCCTGCCCGGT 1859
P6CDO1           CAAAAACACAGCACTCTGCTAACTCGTAAGAGGATGTATAGGGTGTGACGCCTGCCCGGT 1856
P12CDO-S1        CAAAAACACAGCACTCTGCTAACTCGTAAGAGGATGTATAGGGTGTGACGCCTGCCCGGT 1856
P13UCO-S3        CAAAAACACAGCACTCTGCTAACTCGTAAGAGGATGTATAGGGTGTGACGCCTGCCCGGT 1858
P15UCO-S2        CAAAAACACAGCACTCTGCTAACTCGTAAGAGGATGTATAGGGTGTGACGCCTGCCCGGT 1859
P16UCO-S1        CAAAAACACAGCACTCTGCTAACTCGTAAGAGGATGTATAGGGTGTGACGCCTGCCCGGT 1856
P16UCO-S2        CAAAAACACAGCACTCTGCTAACTCGTAAGAGGATGTATAGGGTGTGACGCCTGCCCGGT 1855
P18CDO-S1        CAAAAACACAGCACTCTGCTAACTCGTAAGAGGATGTATAGGGTGTGACGCCTGCCCGGT 1856
P20CDO-S1        CAAAAACACAGCACTCTGCTAACTCGTAAGAGGATGTATAGGGTGTGACGCCTGCCCGGT 1858
P20CDO-S2        CAAAAACACAGCACTCTGCTAACTCGTAAGAGGATGTATAGGGTGTGACGCCTGCCCGGT 1859
P20CDO-S3        CAAAAACACAGCACTCTGCTAACTCGTAAGAGGATGTATAGGGTGTGACGCCTGCCCGGT 1858
P21CDO-S1        CAAAAACACAGCACTCTGCTAACTCGTAAGAGGATGTATAGGGTGTGACGCCTGCCCGGT 1858
P21CDO-S2        CAAAAACACAGCACTCTGCTAACTCGTAAGAGGATGTATAGGGTGTGACGCCTGCCCGGT 1859
P21CDO-S4        CAAAAACACAGCACTCTGCTAACTCGTAAGAGGATGTATAGGGTGTGACGCCTGCCCGGT 1859
P24CDO-S2        CAAAAACACAGCACTCTGCTAACTCGTAAGAGGATGTATAGGGTGTGACGCCTGCCCGGT 1859
P24CDO-S3        CAAAAACACAGCACTCTGCTAACTCGTAAGAGGATGTATAGGGTGTGACGCCTGCCCGGT 1859
P24CDO-S4        CAAAAACACAGCACTCTGCTAACTCGTAAGAGGATGTATAGGGTGTGACGCCTGCCCGGT 1859
UNSW1            CAAAAACACAGCACTCTGCTAACTCGTAAGAGGATGTATAGGGTGTGACGCCTGCCCGGT 1859
UNSW2            CAAAAACACAGCACTCTGCTAACTCGTAAGAGGATGTATAGGGTGTGACGCCTGCCCGGT 1859
UNSW3            CAAAAACACAGCACTCTGCTAACTCGTAAGAGGATGTATAGGGTGTGACGCCTGCCCGGT 1859
UNSWCD           CAAAAACACAGCACTCTGCTAACTCGTAAGAGGATGTATAGGGTGTGACGCCTGCCCGGT 1856
UNSWCS           CAAAAACACAGCACTCTGCTAACTCGTAAGAGGATGTATAGGGTGTGACGCCTGCCCGGT 1860
                 ************************************************************
```

Supplementary Figure S2

```
ATCC33237       GCTCGAAGGTTAATTGATGACGTTAGCTCTGCGAAGCGTTTGATCGAAGCCCGAGTAAAC 1918
ATCC51562       GCTCGAAGGTTAATTGATGACGTTAGCTCTGCGAAGCGTTTGATCGAAGCCCGAGTAAAC 1915
H1O1            GCTCGAAGGTTAATTGATGACGTTAGCTCTGCGAAGCGTTTGATCGAAGCCCGAGTAAAC 1919
H10O-S1         GCTCGAAGGTTAATTGATGACGTTAGCTCTGCGAAGCGTTTGATCGAAGCCCGAGTAAAC 1915
H17O-S1         GCTCGAAGGTTAATTGATGACGTTAGCTCTGCGAAGCGTTTGATCGAAGCCCGAGTAAAC 1919
H21O-S3         GCTCGAAGGTTAATTGATGACGTTAGCTCTGCGAAGCGTTTGATCGAAGCCCGAGTAAAC 1919
P3UCO1          GCTCGAAGGTTAATTGATGACGTTAGCTCTGCGAAGCGTTTGATCGAAGCCCGAGTAAAC 1918
P3UCB1          GCTCGAAGGTTAATTGATGACGTTAGCTCTGCGAAGCGTTTGATCGAAGCCCGAGTAAAC 1918
P9CDO-S1        GCTCGAAGGTTAATTGATGACGTTAGCTCTGCGAAGCGTTTGATCGAAGCCCGAGTAAAC 1915
P20CDO-S4       GCTCGAAGGTTAATTGATGACGTTAGCTCTGCGAAGCGTTTGATCGAAGCCCGAGTAAAC 1918
13826           GCTCGAAGGTTAATTGATGACGTTAGCTCTGCGAAGCGTTTGATCGAAGCCCGAGTAAAC 1919
ATCC51561       GCTCGAAGGTTAATTGATGACGTTAGCTCTGCGAAGCGTTTGATCGAAGCCCGAGTAAAC 1916
H3O1            GCTCGAAGGTTAATTGATGACGTTAGCTCTGCGAAGCGTTTGATCGAAGCCCGAGTAAAC 1916
H7O-S1          GCTCGAAGGTTAATTGATGACGTTAGCTCTGCGAAGCGTTTGATCGAAGCCCGAGTAAAC 1916
H9O-S1          GCTCGAAGGTTAATTGATGACGTTAGCTCTGCGAAGCGTTTGATCGAAGCCCGAGTAAAC 1915
H9O-S2          GCTCGAAGGTTAATTGATGACGTTAGCTCTGCGAAGCGTTTGATCGAAGCCCGAGTAAAC 1919
H11O-S1         GCTCGAAGGTTAATTGATGACGTTAGCTCTGCGAAGCGTTTGATCGAAGCCCGAGTAAAC 1916
H14O-S1         GCTCGAAGGTTAATTGATGACGTTAGCTCTGCGAAGCGTTTGATCGAAGCCCGAGTAAAC 1918
H16O-S1         GCTCGAAGGTTAATTGATGACGTTAGCTCTGCGAAGCGTTTGATCGAAGCCCGAGTAAAC 1916
H20O-S1         GCTCGAAGGTTAATTGATGACGTTAGCTCTGCGAAGCGTTTGATCGAAGCCCGAGTAAAC 1916
H21O-S1         GCTCGAAGGTTAATTGATGACGTTAGCTCTGCGAAGCGTTTGATCGAAGCCCGAGTAAAC 1919
H21O-S2         GCTCGAAGGTTAATTGATGACGTTAGCTCTGCGAAGCGTTTGATCGAAGCCCGAGTAAAC 1919
H21O-S5         GCTCGAAGGTTAATTGATGACGTTAGCTCTGCGAAGCGTTTGATCGAAGCCCGAGTAAAC 1919
H22O-S1         GCTCGAAGGTTAATTGATGACGTTAGCTCTGCGAAGCGTTTGATCGAAGCCCGAGTAAAC 1918
H23O-S1         GCTCGAAGGTTAATTGATGATGTTAGCTCTGCGAAGCGTTTGATCGAAGCCCGAGTAAAC 1918
P2CDO3          GCTCGAAGGTTAATTGATGACGTTAGCTCTGCGAAGCGTTTGATCGAAGCCCGAGTAAAC 1919
P2CDO4          GCTCGAAGGTTAATTGATGACGTTAGCTCTGCGAAGCGTTTGATCGAAGCCCGAGTAAAC 1919
P2CDO-S6        GCTCGAAGGTTAATTGATGACGTTAGCTCTGCGAAGCGTTTGATCGAAGCCCGAGTAAAC 1919
P6CDO1          GCTCGAAGGTTAATTGATGACGTTAGCTCTGCGAAGCGTTTGATCGAAGCCCGAGTAAAC 1916
P12CDO-S1       GCTCGAAGGTTAATTGATGACGTTAGCTCTGCGAAGCGTTTGATCGAAGCCCGAGTAAAC 1916
P13UCO-S3       GCTCGAAGGTTAATTGATGATGTTAGCTCTGCGAAGCGTTTGATCGAAGCCCGAGTAAAC 1918
P15UCO-S2       GCTCGAAGGTTAATTGATGACGTTAGCTCTGCGAAGCGTTTGATCGAAGCCCGAGTAAAC 1919
P16UCO-S1       GCTCGAAGGTTAATTGATGACGTTAGCTCTGCGAAGCGTTTGATCGAAGCCCGAGTAAAC 1916
P16UCO-S2       GCTCGAAGGTTAATTGATGATGTTAGCTCTGCGAAGCGTTTGATCGAAGCCCGAGTAAAC 1915
P18CDO-S1       GCTCGAAGGTTAATTGATGACGTTAGCTCTGCGAAGCGTTTGATCGAAGCCCGAGTAAAC 1916
P20CDO-S1       GCTCGAAGGTTAATTGATGACGTTAGCTCTGCGAAGCGTTTGATCGAAGCCCGAGTAAAC 1918
P20CDO-S2       GCTCGAAGGTTAATTGATGACGTTAGCTCTGCGAAGCGTTTGATCGAAGCCCGAGTAAAC 1919
P20CDO-S3       GCTCGAAGGTTAATTGATGACGTTAGCTCTGCGAAGCGTTTGATCGAAGCCCGAGTAAAC 1918
P21CDO-S1       GCTCGAAGGTTAATTGATGACGTTAGCTCTGCGAAGCGTTTGATCGAAGCCCGAGTAAAC 1918
P21CDO-S2       GCTCGAAGGTTAATTGATGACGTTAGCTCTGCGAAGCGTTTGATCGAAGCCCGAGTAAAC 1919
P21CDO-S4       GCTCGAAGGTTAATTGATGACGTTAGCTCTGCGAAGCGTTTGATCGAAGCCCGAGTAAAC 1919
P24CDO-S2       GCTCGAAGGTTAATTGATGACGTTAGCTCTGCGAAGCGTTTGATCGAAGCCCGAGTAAAC 1919
P24CDO-S3       GCTCGAAGGTTAATTGATGACGTTAGCTCTGCGAAGCGTTTGATCGAAGCCCGAGTAAAC 1919
P24CDO-S4       GCTCGAAGGTTAATTGATGACGTTAGCTCTGCGAAGCGTTTGATCGAAGCCCGAGTAAAC 1919
UNSW1           GCTCGAAGGTTAATTGATGACGTTAGCTCTGCGAAGCGTTTGATCGAAGCCCGAGTAAAC 1919
UNSW2           GCTCGAAGGTTAATTGATGACGTTAGCTCTGCGAAGCGTTTGATCGAAGCCCGAGTAAAC 1919
UNSW3           GCTCGAAGGTTAATTGATGACGTTAGCTCTGCGAAGCGTTTGATCGAAGCCCGAGTAAAC 1919
UNSWCD          GCTCGAAGGTTAATTGATGACGTTAGCTCTGCGAAGCGTTTGATCGAAGCCCGAGTAAAC 1916
UNSWCS          GCTCGAAGGTTAATTGATGACGTTAGCTCTGCGAAGCGTTTGATCGAAGCCCGAGTAAAC 1920
                ******************** ***************************************
```

Supplementary Figure S2

```
ATCC33237      GGCGGCCGTAACTATAACGGTCCTAAGGTAGCGAAATTCCTTGTCGATTAAATATCGACC 1978
ATCC51562      GGCGGCCGTAACTATAACGGTCCTAAGGTAGCGAAATTCCTTGTCGATTAAATATCGACC 1975
H1O1           GGCGGCCGTAACTATAACGGTCCTAAGGTAGCGAAATTCCTTGTCGATTAAATATCGACC 1979
H10O-S1        GGCGGCCGTAACTATAACGGTCCTAAGGTAGCGAAATTCCTTGTCGATTAAATATCGACC 1975
H17O-S1        GGCGGCCGTAACTATAACGGTCCTAAGGTAGCGAAATTCCTTGTCGATTAAATATCGACC 1979
H21O-S3        GGCGGCCGTAACTATAACGGTCCTAAGGTAGCGAAATTCCTTGTCGATTAAATATCGACC 1979
P3UCO1         GGCGGCCGTAACTATAACGGTCCTAAGGTAGCGAAATTCCTTGTCGATTAAATATCGACC 1978
P3UCB1         GGCGGCCGTAACTATAACGGTCCTAAGGTAGCGAAATTCCTTGTCGATTAAATATCGACC 1978
P9CDO-S1       GGCGGCCGTAACTATAACGGTCCTAAGGTAGCGAAATTCCTTGTCGATTAAATATCGACC 1975
P20CDO-S4      GGCGGCCGTAACTATAACGGTCCTAAGGTAGCGAAATTCCTTGTCGATTAAATATCGACC 1978
13826          GGCGGCCGTAACTATAACGGTCCTAAGGTAGCGAAATTCCTTGTCGATTAAATATCGACC 1979
ATCC51561      GGCGGCCGTAACTATAACGGTCCTAAGGTAGCGAAATTCCTTGTCGATTAAATATCGACC 1976
H3O1           GGCGGCCGTAACTATAACGGTCCTAAGGTAGCGAAATTCCTTGTCGATTAAATATCGACC 1976
H7O-S1         GGCGGCCGTAACTATAACGGTCCTAAGGTAGCGAAATTCCTTGTCGATTAAATATCGACC 1976
H9O-S1         GGCGGCCGTAACTATAACGGTCCTAAGGTAGCGAAATTCCTTGTCGATTAAATATCGACC 1975
H9O-S2         GGCGGCCGTAACTATAACGGTCCTAAGGTAGCGAAATTCCTTGTCGATTAAATATCGACC 1979
H11O-S1        GGCGGCCGTAACTATAACGGTCCTAAGGTAGCGAAATTCCTTGTCGATTAAATATCGACC 1976
H14O-S1        GGCGGCCGTAACTATAACGGTCCTAAGGTAGCGAAATTCCTTGTCGATTAAATATCGACC 1978
H16O-S1        GGCGGCCGTAACTATAACGGTCCTAAGGTAGCGAAATTCCTTGTCGATTAAATATCGACC 1976
H20O-S1        GGCGGCCGTAACTATAACGGTCCTAAGGTAGCGAAATTCCTTGTCGATTAAATATCGACC 1976
H21O-S1        GGCGGCCGTAACTATAACGGTCCTAAGGTAGCGAAATTCCTTGTCGATTAAATATCGACC 1979
H21O-S2        GGCGGCCGTAACTATAACGGTCCTAAGGTAGCGAAATTCCTTGTCGATTAAATATCGACC 1979
H21O-S5        GGCGGCCGTAACTATAACGGTCCTAAGGTAGCGAAATTCCTTGTCGATTAAATATCGACC 1979
H22O-S1        GGCGGCCGTAACTATAACGGTCCTAAGGTAGCGAAATTCCTTGTCGATTAAATATCGACC 1978
H23O-S1        GGCGGCCGTAACTATAACGGTCCTAAGGTAGCGAAATTCCTTGTCGATTAAATATCGACC 1978
P2CDO3         GGCGGCCGTAACTATAACGGTCCTAAGGTAGCGAAATTCCTTGTCGATTAAATATCGACC 1979
P2CDO4         GGCGGCCGTAACTATAACGGTCCTAAGGTAGCGAAATTCCTTGTCGATTAAATATCGACC 1979
P2CDO-S6       GGCGGCCGTAACTATAACGGTCCTAAGGTAGCGAAATTCCTTGTCGATTAAATATCGACC 1979
P6CDO1         GGCGGCCGTAACTATAACGGTCCTAAGGTAGCGAAATTCCTTGTCGATTAAATATCGACC 1976
P12CDO-S1      GGCGGCCGTAACTATAACGGTCCTAAGGTAGCGAAATTCCTTGTCGATTAAATATCGACC 1976
P13UCO-S3      GGCGGCCGTAACTATAACGGTCCTAAGGTAGCGAAATTCCTTGTCGATTAAATATCGACC 1978
P15UCO-S2      GGCGGCCGTAACTATAACGGTCCTAAGGTAGCGAAATTCCTTGTCGATTAAATATCGACC 1979
P16UCO-S1      GGCGGCCGTAACTATAACGGTCCTAAGGTAGCGAAATTCCTTGTCGATTAAATATCGACC 1976
P16UCO-S2      GGCGGCCGTAACTATAACGGTCCTAAGGTAGCGAAATTCCTTGTCGATTAAATATCGACC 1975
P18CDO-S1      GGCGGCCGTAACTATAACGGTCCTAAGGTAGCGAAATTCCTTGTCGATTAAATATCGACC 1976
P20CDO-S1      GGCGGCCGTAACTATAACGGTCCTAAGGTAGCGAAATTCCTTGTCGATTAAATATCGACC 1978
P20CDO-S2      GGCGGCCGTAACTATAACGGTCCTAAGGTAGCGAAATTCCTTGTCGATTAAATATCGACC 1979
P20CDO-S3      GGCGGCCGTAACTATAACGGTCCTAAGGTAGCGAAATTCCTTGTCGATTAAATATCGACC 1978
P21CDO-S1      GGCGGCCGTAACTATAACGGTCCTAAGGTAGCGAAATTCCTTGTCGATTAAATATCGACC 1978
P21CDO-S2      GGCGGCCGTAACTATAACGGTCCTAAGGTAGCGAAATTCCTTGTCGATTAAATATCGACC 1979
P21CDO-S4      GGCGGCCGTAACTATAACGGTCCTAAGGTAGCGAAATTCCTTGTCGATTAAATATCGACC 1979
P24CDO-S2      GGCGGCCGTAACTATAACGGTCCTAAGGTAGCGAAATTCCTTGTCGATTAAATATCGACC 1979
P24CDO-S3      GGCGGCCGTAACTATAACGGTCCTAAGGTAGCGAAATTCCTTGTCGATTAAATATCGACC 1979
P24CDO-S4      GGCGGCCGTAACTATAACGGTCCTAAGGTAGCGAAATTCCTTGTCGATTAAATATCGACC 1979
UNSW1          GGCGGCCGTAACTATAACGGTCCTAAGGTAGCGAAATTCCTTGTCGATTAAATATCGACC 1979
UNSW2          GGCGGCCGTAACTATAACGGTCCTAAGGTAGCGAAATTCCTTGTCGATTAAATATCGACC 1979
UNSW3          GGCGGCCGTAACTATAACGGTCCTAAGGTAGCGAAATTCCTTGTCGATTAAATATCGACC 1979
UNSWCD         GGCGGCCGTAACTATAACGGTCCTAAGGTAGCGAAATTCCTTGTCGATTAAATATCGACC 1976
UNSWCS         GGCGGCCGTAACTATAACGGTCCTAAGGTAGCGAAATTCCTTGTCGATTAAATATCGACC 1980
               ************************************************************
```

Supplementary Figure S2

```
ATCC33237        TGCATGAATGGCGTAACGAGATGGGAGCTGTCTCGAAGAGGGATCCAGTGAAATTGTAGT 2038
ATCC51562        TGCATGAATGGCGTAACGAGATGGGAGCTGTCTCGAAGAGGGATCCAGTGAAATTGTAGT 2035
H1O1             TGCATGAATGGCGTAACGAGATGGGAGCTGTCTCGAAGAGGGATCCAGTGAAATTGTAGT 2039
H10O-S1          TGCATGAATGGCGTAACGAGATGGGAGCTGTCTCGAAGAGGGATCCAGTGAAATTGTAGT 2035
H17O-S1          TGCATGAATGGCGTAACGAGATGGGAGCTGTCTCGAAGAGGGATCCAGTGAAATTGTAGT 2039
H21O-S3          TGCATGAATGGCGTAACGAGATGGGAGCTGTCTCGAAGAGGGATCCAGTGAAATTGTAGT 2039
P3UCO1           TGCATGAATGGCGTAACGAGATGGGAGCTGTCTCGAAGAGGGATCCAGTGAAATTGTAGT 2038
P3UCB1           TGCATGAATGGCGTAACGAGATGGGAGCTGTCTCGAAGAGGGATCCAGTGAAATTGTAGT 2038
P9CDO-S1         TGCATGAATGGCGTAACGAGATGGGAGCTGTCTCGAAGAGGGATCCAGTGAAATTGTAGT 2035
P20CDO-S4        TGCATGAATGGCGTAACGAGATGGGAGCTGTCTCGAAGAGGGATCCAGTGAAATTGTAGT 2038
13826            TGCATGAATGGCGTAACGAGATGGGAGCTGTCTCGAAGAGGGATCCAGTGAAATTGTAGT 2039
ATCC51561        TGCATGAATGGCGTAACGAGATGGGAGCTGTCTCGAAGAGGGATCCAGTGAAATTGTAGT 2036
H3O1             TGCATGAATGGCGTAACGAGATGGGAGCTGTCTCGAAGAGGGATCCAGTGAAATTGTAGT 2036
H7O-S1           TGCATGAATGGCGTAACGAGATGGGAGCTGTCTCGAAGAGGGATCCAGTGAAATTGTAGT 2036
H9O-S1           TGCATGAATGGCGTAACGAGATGGGAGCTGTCTCGAAGAGGGATCCAGTGAAATTGTAGT 2035
H9O-S2           TGCATGAATGGCGTAACGAGATGGGAGCTGTCTCGAAGAGGGATCCAGTGAAATTGTAGT 2039
H11O-S1          TGCATGAATGGCGTAACGAGATGGGAGCTGTCTCGAAGAGGGATCCAGTGAAATTGTAGT 2036
H14O-S1          TGCATGAATGGCGTAACGAGATGGGAGCTGTCTCGAAGAGGGATCCAGTGAAATTGTAGT 2038
H16O-S1          TGCATGAATGGCGTAACGAGATGGGAGCTGTCTCGAAGAGGGATCCAGTGAAATTGTAGT 2036
H20O-S1          TGCATGAATGGCGTAACGAGATGGGAGCTGTCTCGAAGAGGGATCCAGTGAAATTGTAGT 2036
H21O-S1          TGCATGAATGGCGTAACGAGATGGGAGCTGTCTCGAAGAGGGATCCAGTGAAATTGTAGT 2039
H21O-S2          TGCATGAATGGCGTAACGAGATGGGAGCTGTCTCGAAGAGGGATCCAGTGAAATTGTAGT 2039
H21O-S5          TGCATGAATGGCGTAACGAGATGGGAGCTGTCTCGAAGAGGGATCCAGTGAAATTGTAGT 2039
H22O-S1          TGCATGAATGGCGTAACGAGATGGGAGCTGTCTCGAAGAGGGATCCAGTGAAATTGTAGT 2038
H23O-S1          TGCATGAATGGCGTAACGAGATGGGAGCTGTCTCGAAGAGGGATCCAGTGAAATTGTAGT 2038
P2CDO3           TGCATGAATGGCGTAACGAGATGGGAGCTGTCTCGAAGAGGGATCCAGTGAAATTGTAGT 2039
P2CDO4           TGCATGAATGGCGTAACGAGATGGGAGCTGTCTCGAAGAGGGATCCAGTGAAATTGTAGT 2039
P2CDO-S6         TGCATGAATGGCGTAACGAGATGGGAGCTGTCTCGAAGAGGGATCCAGTGAAATTGTAGT 2039
P6CDO1           TGCATGAATGGCGTAACGAGATGGGAGCTGTCTCGAAGAGGGATCCAGTGAAATTGTAGT 2036
P12CDO-S1        TGCATGAATGGCGTAACGAGATGGGAGCTGTCTCGAAGAGGGATCCAGTGAAATTGTAGT 2036
P13UCO-S3        TGCATGAATGGCGTAACGAGATGGGAGCTGTCTCGAAGAGGGATCCAGTGAAATTGTAGT 2038
P15UCO-S2        TGCATGAATGGCGTAACGAGATGGGAGCTGTCTCGAAGAGGGATCCAGTGAAATTGTAGT 2039
P16UCO-S1        TGCATGAATGGCGTAACGAGATGGGAGCTGTCTCGAAGAGGGATCCAGTGAAATTGTAGT 2036
P16UCO-S2        TGCATGAATGGCGTAACGAGATGGGAGCTGTCTCGAAGAGGGATCCAGTGAAATTGTAGT 2035
P18CDO-S1        TGCATGAATGGCGTAACGAGATGGGAGCTGTCTCGAAGAGGGATCCAGTGAAATTGTAGT 2036
P20CDO-S1        TGCATGAATGGCGTAACGAGATGGGAGCTGTCTCGAAGAGGGATCCAGTGAAATTGTAGT 2038
P20CDO-S2        TGCATGAATGGCGTAACGAGATGGGAGCTGTCTCGAAGAGGGATCCAGTGAAATTGTAGT 2039
P20CDO-S3        TGCATGAATGGCGTAACGAGATGGGAGCTGTCTCGAAGAGGGATCCAGTGAAATTGTAGT 2038
P21CDO-S1        TGCATGAATGGCGTAACGAGATGGGAGCTGTCTCGAAGAGGGATCCAGTGAAATTGTAGT 2038
P21CDO-S2        TGCATGAATGGCGTAACGAGATGGGAGCTGTCTCGAAGAGGGATCCAGTGAAATTGTAGT 2039
P21CDO-S4        TGCATGAATGGCGTAACGAGATGGGAGCTGTCTCGAAGAGGGATCCAGTGAAATTGTAGT 2039
P24CDO-S2        TGCATGAATGGCGTAACGAGATGGGAGCTGTCTCGAAGAGGGATCCAGTGAAATTGTAGT 2039
P24CDO-S3        TGCATGAATGGCGTAACGAGATGGGAGCTGTCTCGAAGAGGGATCCAGTGAAATTGTAGT 2039
P24CDO-S4        TGCATGAATGGCGTAACGAGATGGGAGCTGTCTCGAAGAGGGATCCAGTGAAATTGTAGT 2039
UNSW1            TGCATGAATGGCGTAACGAGATGGGAGCTGTCTCGAAGAGGGATCCAGTGAAATTGTAGT 2039
UNSW2            TGCATGAATGGCGTAACGAGATGGGAGCTGTCTCGAAGAGGGATCCAGTGAAATTGTAGT 2039
UNSW3            TGCATGAATGGCGTAACGAGATGGGAGCTGTCTCGAAGAGGGATCCAGTGAAATTGTAGT 2039
UNSWCD           TGCATGAATGGCGTAACGAGATGGGAGCTGTCTCGAAGAGGGATCCAGTGAAATTGTAGT 2036
UNSWCS           TGCATGAATGGCGTAACGAGATGGGAGCTGTCTCGAAGAGGGATCCAGTGAAATTGTAGT 2040
                 ************************************************************
```

Supplementary Figure S2

```
ATCC33237       GGAGGTGAAAATTCCTCCTACCCGCGGCAAGACGGAAAGACCCCGTGGACCTTTACTACA 2098
ATCC51562       GGAGGTGAAAATTCCTCCTACCCGCGGCAAGACGGAAAGACCCCGTGGACCTTTACTACA 2095
H1O1            GGAGGTGAAAATTCCTCCTACCCGCGGCAAGACGGAAAGACCCCGTGGACCTTTACTACA 2099
H10O-S1         GGAGGTGAAAATTCCTCCTACCCGCGGCAAGACGGAAAGACCCCGTGGACCTTTACTACA 2095
H17O-S1         GGAGGTGAAAATTCCTCCTACCCGCGGCAAGACGGAAAGACCCCGTGGACCTTTACTACA 2099
H21O-S3         GGAGGTGAAAATTCCTCCTACCCGCGGCAAGACGGAAAGACCCCGTGGACCTTTACTACA 2099
P3UCO1          GGAGGTGAAAATTCCTCCTACCCGCGGCAAGACGGAAAGACCCCGTGGACCTTTACTACA 2098
P3UCB1          GGAGGTGAAAATTCCTCCTACCCGCGGCAAGACGGAAAGACCCCGTGGACCTTTACTACA 2098
P9CDO-S1        GGAGGTGAAAATTCCTCCTACCCGCGGCAAGACGGAAAGACCCCGTGGACCTTTACTACA 2095
P20CDO-S4       GGAGGTGAAAATTCCTCCTACCCGCGGCAAGACGGAAAGACCCCGTGGACCTTTACTACA 2098
13826           GGAGGTGAAAATTCCTCCTACCCGCGGCAAGACGGAAAGACCCCGTGGACCTTTACTACA 2099
ATCC51561       GGAGGTGAAAATTCCTCCTACCCGCGGCAAGACGGAAAGACCCCGTGGACCTTTACTACA 2096
H3O1            GGAGGTGAAAATTCCTCCTACCCGCGGCAAGACGGAAAGACCCCGTGGACCTTTACTACA 2096
H7O-S1          GGAGGTGAAAATTCCTCCTACCCGCGGCAAGACGGAAAGACCCCGTGGACCTTTACTACA 2096
H9O-S1          GGAGGTGAAAATTCCTCCTACCCGCGGCAAGACGGAAAGACCCCGTGGACCTTTACTACA 2095
H9O-S2          GGAGGTGAAAATTCCTCCTACCCGCGGCAAGACGGAAAGACCCCGTGGACCTTTACTACA 2099
H11O-S1         GGAGGTGAAAATTCCTCCTACCCGCGGCAAGACGGAAAGACCCCGTGGACCTTTACTACA 2096
H14O-S1         GGAGGTGAAAATTCCTCCTACCCGCGGCAAGACGGAAAGACCCCGTGGACCTTTACTACA 2098
H16O-S1         GGAGGTGAAAATTCCTCCTACCCGCGGCAAGACGGAAAGACCCCGTGGACCTTTACTACA 2096
H20O-S1         GGAGGTGAAAATTCCTCCTACCCGCGGCAAGACGGAAAGACCCCGTGGACCTTTACTACA 2096
H21O-S1         GGAGGTGAAAATTCCTCCTACCCGCGGCAAGACGGAAAGACCCCGTGGACCTTTACTACA 2099
H21O-S2         GGAGGTGAAAATTCCTCCTACCCGCGGCAAGACGGAAAGACCCCGTGGACCTTTACTACA 2099
H21O-S5         GGAGGTGAAAATTCCTCCTACCCGCGGCAAGACGGAAAGACCCCGTGGACCTTTACTACA 2099
H22O-S1         GGAGGTGAAAATTCCTCCTACCCGCGGCAAGACGGAAAGACCCCGTGGACCTTTACTACA 2098
H23O-S1         GGAGGTGAAAATTCCTCCTACCCGCGGCAAGACGGAAAGACCCCGTGGACCTTTACTACA 2098
P2CDO3          GGAGGTGAAAATTCCTCCTACCCGCGGCAAGACGGAAAGACCCCGTGGACCTTTACTACA 2099
P2CDO4          GGAGGTGAAAATTCCTCCTACCCGCGGCAAGACGGAAAGACCCCGTGGACCTTTACTACA 2099
P2CDO-S6        GGAGGTGAAAATTCCTCCTACCCGCGGCAAGACGGAAAGACCCCGTGGACCTTTACTACA 2099
P6CDO1          GGAGGTGAAAATTCCTCCTACCCGCGGCAAGACGGAAAGACCCCGTGGACCTTTACTACA 2096
P12CDO-S1       GGAGGTGAAAATTCCTCCTACCCGCGGCAAGACGGAAAGACCCCGTGGACCTTTACTACA 2096
P13UCO-S3       GGAGGTGAAAATTCCTCCTACCCGCGGCAAGACGGAAAGACCCCGTGGACCTTTACTACA 2098
P15UCO-S2       GGAGGTGAAAATTCCTCCTACCCGCGGCAAGACGGAAAGACCCCGTGGACCTTTACTACA 2099
P16UCO-S1       GGAGGTGAAAATTCCTCCTACCCGCGGCAAGACGGAAAGACCCCGTGGACCTTTACTACA 2096
P16UCO-S2       GGAGGTGAAAATTCCTCCTACCCGCGGCAAGACGGAAAGACCCCGTGGACCTTTACTACA 2095
P18CDO-S1       GGAGGTGAAAATTCCTCCTACCCGCGGCAAGACGGAAAGACCCCGTGGACCTTTACTACA 2096
P20CDO-S1       GGAGGTGAAAATTCCTCCTACCCGCGGCAAGACGGAAAGACCCCGTGGACCTTTACTACA 2098
P20CDO-S2       GGAGGTGAAAATTCCTCCTACCCGCGGCAAGACGGAAAGACCCCGTGGACCTTTACTACA 2099
P20CDO-S3       GGAGGTGAAAATTCCTCCTACCCGCGGCAAGACGGAAAGACCCCGTGGACCTTTACTACA 2098
P21CDO-S1       GGAGGTGAAAATTCCTCCTACCCGCGGCAAGACGGAAAGACCCCGTGGACCTTTACTACA 2098
P21CDO-S2       GGAGGTGAAAATTCCTCCTACCCGCGGCAAGACGGAAAGACCCCGTGGACCTTTACTACA 2099
P21CDO-S4       GGAGGTGAAAATTCCTCCTACCCGCGGCAAGACGGAAAGACCCCGTGGACCTTTACTACA 2099
P24CDO-S2       GGAGGTGAAAATTCCTCCTACCCGCGGCAAGACGGAAAGACCCCGTGGACCTTTACTACA 2099
P24CDO-S3       GGAGGTGAAAATTCCTCCTACCCGCGGCAAGACGGAAAGACCCCGTGGACCTTTACTACA 2099
P24CDO-S4       GGAGGTGAAAATTCCTCCTACCCGCGGCAAGACGGAAAGACCCCGTGGACCTTTACTACA 2099
UNSW1           GGAGGTGAAAATTCCTCCTACCCGCGGCAAGACGGAAAGACCCCGTGGACCTTTACTACA 2099
UNSW2           GGAGGTGAAAATTCCTCCTACCCGCGGCAAGACGGAAAGACCCCGTGGACCTTTACTACA 2099
UNSW3           GGAGGTGAAAATTCCTCCTACCCGCGGCAAGACGGAAAGACCCCGTGGACCTTTACTACA 2099
UNSWCD          GGAGGTGAAAATTCCTCCTACCCGCGGCAAGACGGAAAGACCCCGTGGACCTTTACTACA 2096
UNSWCS          GGAGGTGAAAATTCCTCCTACCCGCGGCAAGACGGAAAGACCCCGTGGACCTTTACTACA 2100
                ************************************************************
```

Supplementary Figure S2

```
ATCC33237       GCTTGACACTGCTATTGGGATAAAAATGTGCAGGATAGGTGGGAGGCTTTGATCTATAGA 2158
ATCC51562       GCTTGACACTGCTATTGGGATAAAAATGTGCAGGATAGGTGGGAGGCTTTGATCCATAGA 2155
H1O1            GCTTGACACTGCTATTGGGATAAAAATGTGCAGGATAGGTGGGAGGCTTTGATCCATAGA 2159
H10O-S1         GCTTGACACTGCTATTGGGATAAAAATGTGCAGGATAGGTGGGAGGCTTTGATCCATAGA 2155
H17O-S1         GCTTGACACTGCTATTGGGATAAAAATGTGCAGGATAGGTGGGAGGCTTTGATCCATAGA 2159
H21O-S3         GCTTGACACTGCTATTGGGATAAAAATGTGCAGGATAGGTGGGAGGCTTTGATCCATAGA 2159
P3UCO1          GCTTGACACTGCTATTGGGATAAAAATGTGCAGGATAGGTGGGAGGCTTTGATCCATAGA 2158
P3UCB1          GCTTGACACTGCTATTGGGATAAAAATGTGCAGGATAGGTGGGAGGCTTTGATCCATAGA 2158
P9CDO-S1        GCTTGACACTGCTATTGGGATAAAAATGTGCAGGATAGGTGGGAGGCTTTGATCCATAGA 2155
P20CDO-S4       GCTTGACACTGCTATTGGGATAAAAATGTGCAGGATAGGTGGGAGGCTTTGATCCATAGA 2158
13826           GCTTGACACTGCTATTGGGATAAAAATGTGCAGGATAGGCGGGAGGCTTTGATCCATAGA 2159
ATCC51561       GCTTGACACTGCTATTGGGATAAAAATGTGCAGGATAGGCGGGAGGCTTTGATCCATAGA 2156
H3O1            GCTTGACACTGCTATTGGGATAAAAATGTGCAGGATAGGCGGGAGGCTTTGATCCATAGA 2156
H7O-S1          GCTTGACACTGCTATTGGGATAAAAATGTGCAGGATAGGCGGGAGGCTTTGATCCATAGA 2156
H9O-S1          GCTTGACACTGCTATTGGGATAAAAATGTGCAGGATAGGCGGGAGGCTTTGATCCATAGA 2155
H9O-S2          GCTTGACACTGCTATTGGGATAAAAATGTGCAGGATAGGCGGGAGGCTTTGATCCATAGA 2159
H11O-S1         GCTTGACACTGCTATTGGGATAAAAATGTGCAGGATAGGCGGGAGGCTTTGATCCATAGA 2156
H14O-S1         GCTTGACACTGCTATTGGGATAAAAATGTGCAGGATAGGCGGGAGGCTTTGATCCATAGA 2158
H16O-S1         GCTTGACACTGCTATTGGGATAAAAATGTGCAGGATAGGCGGGAGGCTTTGATCCATAGA 2156
H20O-S1         GCTTGACACTGCTATTGGGATAAAAATGTGCAGGATAGGCGGGAGGCTTTGATCCATAGA 2156
H21O-S1         GCTTGACACTGCTATTGGGATAAAAATGTGCAGGATAGGCGGGAGGCTTTGATCCATAGA 2159
H21O-S2         GCTTGACACTGCTATTGGGATAAAAATGTGCAGGATAGGCGGGAGGCTTTGATCCATAGA 2159
H21O-S5         GCTTGACACTGCTATTGGGATAAAAATGTGCAGGATAGGCGGGAGGCTTTGATCCATAGA 2159
H22O-S1         GCTTGACACTGCTATTGGGATAAAAATGTGCAGGATAGGCGGGAGGCTTTGATCCATAGA 2158
H23O-S1         GCTTGACACTGCTATTGGGATAAAAATGTGCAGGATAGGCGGGAGGCTTTGATCCATAGA 2158
P2CDO3          GCTTGACACTGCTATTGGGATAAAAATGTGCAGGATAGGCGGGAGGCTTTGATCCATAGA 2159
P2CDO4          GCTTGACACTGCTATTGGGATAAAAATGTGCAGGATAGGCGGGAGGCTTTGATCCATAGA 2159
P2CDO-S6        GCTTGACACTGCTATTGGGATAAAAATGTGCAGGATAGGCGGGAGGCTTTGATCCATAGA 2159
P6CDO1          GCTTGACACTGCTATTGGGATAAAAATGTGCAGGATAGGCGGGAGGCTTTGATCCATAGA 2156
P12CDO-S1       GCTTGACACTGCTATTGGGATAAAAATGTGCAGGATAGGCGGGAGGCTTTGATCCATAGA 2156
P13UCO-S3       GCTTGACACTGCTATTGGGATAAAAATGTGCAGGATAGGCGGGAGGCTTTGATCCATAGA 2158
P15UCO-S2       GCTTGACACTGCTATTGGGATAAAAATGTGCAGGATAGGCGGGAGGCTTTGATCCATAGA 2159
P16UCO-S1       GCTTGACACTGCTATTGGGATAAAAATGTGCAGGATAGGCGGGAGGCTTTGATCCATAGA 2156
P16UCO-S2       GCTTGACACTGCTATTGGGATAAAAATGTGCAGGATAGGCGGGAGGCTTTGATCCATAGA 2155
P18CDO-S1       GCTTGACACTGCTATTGGGATAAAAATGTGCAGGATAGGCGGGAGGCTTTGATCCATAGA 2156
P20CDO-S1       GCTTGACACTGCTATTGGGATAAAAATGTGCAGGATAGGCGGGAGGCTTTGATCCATAGA 2158
P20CDO-S2       GCTTGACACTGCTATTGGGATAAAAATGTGCAGGATAGGCGGGAGGCTTTGATCCATAGA 2159
P20CDO-S3       GCTTGACACTGCTATTGGGATAAAAATGTGCAGGATAGGCGGGAGGCTTTGATCCATAGA 2158
P21CDO-S1       GCTTGACACTGCTATTGGGATAAAAATGTGCAGGATAGGCGGGAGGCTTTGATCCATAGA 2158
P21CDO-S2       GCTTGACACTGCTATTGGGATAAAAATGTGCAGGATAGGCGGGAGGCTTTGATCCATAGA 2159
P21CDO-S4       GCTTGACACTGCTATTGGGATAAAAATGTGCAGGATAGGCGGGAGGCTTTGATCCATAGA 2159
P24CDO-S2       GCTTGACACTGCTATTGGGATAAAAATGTGCAGGATAGGCGGGAGGCTTTGATCCATAGA 2159
P24CDO-S3       GCTTGACACTGCTATTGGGATAAAAATGTGCAGGATAGGCGGGAGGCTTTGATCCATAGA 2159
P24CDO-S4       GCTTGACACTGCTATTGGGATAAAAATGTGCAGGATAGGCGGGAGGCTTTGATCCATAGA 2159
UNSW1           GCTTGACACTGCTATTGGGATAAAAATGTGCAGGATAGGCGGGAGGCTTTGATCCATAGA 2159
UNSW2           GCTTGACACTGCTATTGGGATAAAAATGTGCAGGATAGGCGGGAGGCTTTGATCCATAGA 2159
UNSW3           GCTTGACACTGCTATTGGGATAAAAATGTGCAGGATAGGCGGGAGGCTTTGATCCATAGA 2159
UNSWCD          GCTTGACACTGCTATTGGGATAAAAATGTGCAGGATAGGCGGGAGGCTTTGATCCATAGA 2156
UNSWCS          GCTTGACACTGCTATTGGGATAAAAATGTGCAGGATAGGCGGGAGGCTTTGATCCATAGA 2160
                *************************************** ************** *****
```

Supplementary Figure S2

```
ATCC33237      CGCCAGTTTATGGTGAGCCGTTGTTGAGATACCACTCTTTTTTATTCTGATAGCTAACTA 2218
ATCC51562      CGCCAGTTTATGGTGAGCCATTGTTGAGATACCACTCTTTTTTATTCTGATAGCTAACTA 2215
H1O1           CGCCAGTTTATGGTGAGCCGTTGTTGAGATACCACTCTTTTTTATTCTGATAGCTAACTA 2219
H10O-S1        CGCCAGTTTATGGTGAGCCATTGTTGAGATACCACTCTTTTTTATTCTGATAGCTAACTA 2215
H17O-S1        CGCCAGTTTATGGTGAGCCGTTGTTGAGATACCACTCTTTTTTATTCTGATAGCTAACTA 2219
H21O-S3        CGCCAGTTTATGGTGAGCCGTTGTTGAGATACCACTCTTTTTTATTCTGATAGCTAACTA 2219
P3UCO1         CGCCAGTTTATGGTGAGCCATTGTTGAGATACCACTCTTTTTTATTCTGATAGCTAACTA 2218
P3UCB1         CGCCAGTTTATGGTGAGCCATTGTTGAGATACCACTCTTTTTTATTCTGATAGCTAACTA 2218
P9CDO-S1       CGCCAGTTTATGGTGAGCCATTGTTGAGATACCACTCTTTTTTATTCTGATAGCTAACTA 2215
P20CDO-S4      CGCCAGTTTATGGTGAGCCGTTGTTGAGATACCACTCTTTTTTATTCTGATAGCTAACTA 2218
13826          CGCCAGTTTATGGTGAGCCATTGTTGAGATACCGCTCTTTTTTATTCTGATAGCTAACTA 2219
ATCC51561      CGCCAGTTTATGGTGAGCCATTGTTGAGATACCGCTCTTTTTTATTCTGATAGCTAACTA 2216
H3O1           CGCCAGTTTATGGTGAGCCATTGTTGAGATACCGCTCTTTTTTATTCTGATAGCTAACTA 2216
H7O-S1         CGCCAGTTTATGGTGAGCCATTGTTGAGATACCGCTCTTTTTTATTCTGATAGCTAACTA 2216
H9O-S1         CGCCAGTTTATGGTGAGCCATTGTTGAGATACCGCTCTTTTTTATTCTGATAGCTAACTA 2215
H9O-S2         CGCCAGTTTATGGTGAGCCATTGTTGAGATACCGCTCTTTTTTATTCTGATAGCTAACTA 2219
H11O-S1        CGCCAGTTTATGGTGAGCCATTGTTGAGATACCGCTCTTTTTTATTCTGATAGCTAACTA 2216
H14O-S1        CGCCAGTTTATGGTGAGCCATTGTTGAGATACCGCTCTTTTTTATTCTGATAGCTAACTA 2218
H16O-S1        CGCCAGTTTATGGTGAGCCATTGTTGAGATACCGCTCTTTTTTATTCTGATAGCTAACTA 2216
H20O-S1        CGCCAGTTTATGGTGAGCCATTGTTGAGATACCGCTCTTTTTTATTCTGATAGCTAACTA 2216
H21O-S1        CGCCAGTTTATGGTGAGCCATTGTTGAGATACCGCTCTTTTTTATTCTGATAGCTAACTA 2219
H21O-S2        CGCCAGTTTATGGTGAGCCATTGTTGAGATACCGCTCTTTTTTATTCTGATAGCTAACTA 2219
H21O-S5        CGCCAGTTTATGGTGAGCCATTGTTGAGATACCGCTCTTTTTTATTCTGATAGCTAACTA 2219
H22O-S1        CGCCAGTTTATGGTGAGCCATTGTTGAGATACCGCTCTTTTTTATTCTGATAGCTAACTA 2218
H23O-S1        CGCCAGTTTATGGTGAGCCATTGTTGAGATACCGCTCTTTTTTATTCTGATAGCTAACTA 2218
P2CDO3         CGCCAGTTTATGGTGAGCCATTGTTGAGATACCGCTCTTTTTTATTCTGATAGCTAACTA 2219
P2CDO4         CGCCAGTTTATGGTGAGCCATTGTTGAGATACCGCTCTTTTTTATTCTGATAGCTAACTA 2219
P2CDO-S6       CGCCAGTTTATGGTGAGCCATTGTTGAGATACCGCTCTTTTTTATTCTGATAGCTAACTA 2219
P6CDO1         CGCCAGTTTATGGTGAGCCATTGTTGAGATACCGCTCTTTTTTATTCTGATAGCTAACTA 2216
P12CDO-S1      CGCCAGTTTATGGTGAGCCATTGTTGAGATACCGCTCTTTTTTATTCTGATAGCTAACTA 2216
P13UCO-S3      CGCCAGTTTATGGTGAGCCATTGTTGAGATACCGCTCTTTTTTATTCTGATAGCTAACTA 2218
P15UCO-S2      CGCCAGTTTATGGTGAGCCATTGTTGAGATACCGCTCTTTTTTATTCTGATAGCTAACTA 2219
P16UCO-S1      CGCCAGTTTATGGTGAGCCATTGTTGAGATACCGCTCTTTTTTATTCTGATAGCTAACTA 2216
P16UCO-S2      CGCCAGTTTATGGTGAGCCATTGTTGAGATACCGCTCTTTTTTATTCTGATAGCTAACTA 2215
P18CDO-S1      CGCCAGTTTATGGTGAGCCATTGTTGAGATACCGCTCTTTTTTATTCTGATAGCTAACTA 2216
P20CDO-S1      CGTCAGTTTATGGTGAGCCATTGTTGAGATACCGCTCTTTTTTATTCTGATAGCTAACTA 2218
P20CDO-S2      CGCCAGTTTATGGTGAGCCATTGTTGAGATACCGCTCTTTTTTATTCTGATAGCTAACTA 2219
P20CDO-S3      CGTCAGTTTATGGTGAGCCATTGTTGAGATACCGCTCTTTTTTATTCTGATAGCTAACTA 2218
P21CDO-S1      CGCCAGTTTATGGTGAGCCATTGTTGAGATACCGCTCTTTTTTATTCTGATAGCTAACTA 2218
P21CDO-S2      CGCCAGTTTATGGTGAGCCATTGTTGAGATACCGCTCTTTTTTATTCTGATAGCTAACTA 2219
P21CDO-S4      CGCCAGTTTATGGTGAGCCATTGTTGAGATACCGCTCTTTTTTATTCTGATAGCTAACTA 2219
P24CDO-S2      CGCCAGTTTATGGTGAGCCATTGTTGAGATACCGCTCTTTTTTATTCTGATAGCTAACTA 2219
P24CDO-S3      CGCCAGTTTATGGTGAGCCATTGTTGAGATACCGCTCTTTTTTATTCTGATAGCTAACTA 2219
P24CDO-S4      CGCCAGTTTATGGTGAGCCATTGTTGAGATACCGCTCTTTTTTATTCTGATAGCTAACTA 2219
UNSW1          CGCCAGTTTATGGTGAGCCATTGTTGAGATACCGCTCTTTTTTATTCTGATAGCTAACTA 2219
UNSW2          CGCCAGTTTATGGTGAGCCATTGTTGAGATACCGCTCTTTTTTATTCTGATAGCTAACTA 2219
UNSW3          CGCCAGTTTATGGTGAGCCATTGTTGAGATACCGCTCTTTTTTATTCTGATAGCTAACTA 2219
UNSWCD         CGCCAGTTTATGGTGAGCCATTGTTGAGATACCGCTCTTTTTTATTCTGATAGCTAACTA 2216
UNSWCS         CGCCAGTTTATGGTGAGCCATTGTTGAGATACCGCTCTTTTTTATTCTGATAGCTAACTA 2220
               ** **************** ************* **************************
```

Supplementary Figure S2

```
ATCC33237       GCTTGAGTTATCCTCAAGTAGGACAATGTCTGGTGGGTAGTTTGACTGGGGCGGTCGCCT 2278
ATCC51562       GCTTGAGTTATCCTCAAGTAGGACAATGTCTGGTGGGTAGTTTGACTGGGGCGGTCGCCT 2275
H1O1            GCTTGAGTTATCCTCAAGTAGGACAATGTCTGGTGGGTAGTTTGACTGGGGCGGTCGCCT 2279
H10O-S1         GCTTGAGTTATCCTCAAGTAGGACAATGTCTGGTGGGTAGTTTGACTGGGGCGGTCGCCT 2275
H17O-S1         GCTTGAGTTATCCTCAAGTAGGACAATGTCTGGTGGGTAGTTTGACTGGGGCGGTCGCCT 2279
H21O-S3         GCTTGAGTTATCCTCAAGTAGGACAATGTCTGGTGGGTAGTTTGACTGGGGCGGTCGCCT 2279
P3UCO1          GCTTGAGTTATCCTCAAGTAGGACAATGTCTGGTGGGTAGTTTGACTGGGGCGGTCGCCT 2278
P3UCB1          GCTTGAGTTATCCTCAAGTAGGACAATGTCTGGTGGGTAGTTTGACTGGGGCGGTCGCCT 2278
P9CDO-S1        GCTTGAGTTATCCTCAAGTAGGACAATGTCTGGTGGGTAGTTTGACTGGGGCGGTCGCCT 2275
P20CDO-S4       GCTTGAGTTATCCTCAAGTAGGACAATGTCTGGTGGGTAGTTTGACTGGGGCGGTCGCCT 2278
13826           GCTTGAGTTATCCTCAAGTAGGACAATGTCTGGTGGGTAGTTTGACTGGGGCGGTCGCCT 2279
ATCC51561       GCTTGAGTTATCCTCAAGTAGGACAATGTCTGGTGGGTAGTTTGACTGGGGCGGTCGCCT 2276
H3O1            GCTTGAGTTATCCTCAAGTAGGACAATGTCTGGTGGGTAGTTTGACTGGGGCGGTCGCCT 2276
H7O-S1          GCTTGAGTTATCCTCAAGTAGGACAATGTCTGGTGGGTAGTTTGACTGGGGCGGTCGCCT 2276
H9O-S1          GCTTGAGTTATCCTCAAGTAGGACAATGTCTGGTGGGTAGTTTGACTGGGGCGGTCGCCT 2275
H9O-S2          GCTTGAGTTATCCTCAAGTAGGACAATGTCTGGTGGGTAGTTTGACTGGGGCGGTCGCCT 2279
H11O-S1         GCTTGAGTTATCCTCAAGTAGGACAATGTCTGGTGGGTAGTTTGACTGGGGCGGTCGCCT 2276
H14O-S1         GCTTGAGTTATCCTCAAGTAGGACAATGTCTGGTGGGTAGTTTGACTGGGGCGGTCGCCT 2278
H16O-S1         GCTTGAGTTATCCTCAAGTAGGACAATGTCTGGTGGGTAGTTTGACTGGGGCGGTCGCCT 2276
H20O-S1         GCTTGAGTTATCCTCAAGTAGGACAATGTCTGGTGGGTAGTTTGACTGGGGCGGTCGCCT 2276
H21O-S1         GCTTGAGTTATCCTCAAGTAGGACAATGTCTGGTGGGTAGTTTGACTGGGGCGGTCGCCT 2279
H21O-S2         GCTTGAGTTATCCTCAAGTAGGACAATGTCTGGTGGGTAGTTTGACTGGGGCGGTCGCCT 2279
H21O-S5         GCTTGAGTTATCCTCAAGTAGGACAATGTCTGGTGGGTAGTTTGACTGGGGCGGTCGCCT 2279
H22O-S1         GCTTGAGTTATCCTCAAGTAGGACAATGTCTGGTGGGTAGTTTGACTGGGGCGGTCGCCT 2278
H23O-S1         GCTTGAGTTATCCTCAAGTAGGACAATGTCTGGTGGGTAGTTTGACTGGGGCGGTCGCCT 2278
P2CDO3          GCTTGAGTTATCCTCAAGTAGGACAATGTCTGGTGGGTAGTTTGACTGGGGCGGTCGCCT 2279
P2CDO4          GCTTGAGTTATCCTCAAGTAGGACAATGTCTGGTGGGTAGTTTGACTGGGGCGGTCGCCT 2279
P2CDO-S6        GCTTGAGTTATCCTCAAGTAGGACAATGTCTGGTGGGTAGTTTGACTGGGGCGGTCGCCT 2279
P6CDO1          GCTTGAGTTATCCTCAAGTAGGACAATGTCTGGTGGGTAGTTTGACTGGGGCGGTCGCCT 2276
P12CDO-S1       GCTTGAGTTATCCTCAAGTAGGACAATGTCTGGTGGGTAGTTTGACTGGGGCGGTCGCCT 2276
P13UCO-S3       GCTTGAGTTATCCTCAAGTAGGACAATGTCTGGTGGGTAGTTTGACTGGGGCGGTCGCCT 2278
P15UCO-S2       GCTTGAGTTATCCTCAAGTAGGACAATGTCTGGTGGGTAGTTTGACTGGGGCGGTCGCCT 2279
P16UCO-S1       GCTTGAGTTATCCTCAAGTAGGACAATGTCTGGTGGGTAGTTTGACTGGGGCGGTCGCCT 2276
P16UCO-S2       GCTTGAGTTATCCTCAAGTAGGACAATGTCTGGTGGGTAGTTTGACTGGGGCGGTCGCCT 2275
P18CDO-S1       GCTTGAGTTATCCTCAAGTAGGACAATGTCTGGTGGGTAGTTTGACTGGGGCGGTCGCCT 2276
P20CDO-S1       GCTTGAGTTATCCTCAAGTAGGACAATGTCTGGTGGGTAGTTTGACTGGGGCGGTCGCCT 2278
P20CDO-S2       GCTTGAGTTATCCTCAAGTAGGACAATGTCTGGTGGGTAGTTTGACTGGGGCGGTCGCCT 2279
P20CDO-S3       GCTTGAGTTATCCTCAAGTAGGACAATGTCTGGTGGGTAGTTTGACTGGGGCGGTCGCCT 2278
P21CDO-S1       GCTTGAGTTATCCTCAAGTAGGACAATGTCTGGTGGGTAGTTTGACTGGGGCGGTCGCCT 2278
P21CDO-S2       GCTTGAGTTATCCTCAAGTAGGACAATGTCTGGTGGGTAGTTTGACTGGGGCGGTCGCCT 2279
P21CDO-S4       GCTTGAGTTATCCTCAAGTAGGACAATGTCTGGTGGGTAGTTTGACTGGGGCGGTCGCCT 2279
P24CDO-S2       GCTTGAGTTATCCTCAAGTAGGACAATGTCTGGTGGGTAGTTTGACTGGGGCGGTCGCCT 2279
P24CDO-S3       GCTTGAGTTATCCTCAAGTAGGACAATGTCTGGTGGGTAGTTTGACTGGGGCGGTCGCCT 2279
P24CDO-S4       GCTTGAGTTATCCTCAAGTAGGACAATGTCTGGTGGGTAGTTTGACTGGGGCGGTCGCCT 2279
UNSW1           GCTTGAGTTATCCTCAAGTAGGACAATGTCTGGTGGGTAGTTTGACTGGGGCGGTCGCCT 2279
UNSW2           GCTTGAGTTATCCTCAAGTAGGACAATGTCTGGTGGGTAGTTTGACTGGGGCGGTCGCCT 2279
UNSW3           GCTTGAGTTATCCTCAAGTAGGACAATGTCTGGTGGGTAGTTTGACTGGGGCGGTCGCCT 2279
UNSWCD          GCTTGAGTTATCCTCAAGTAGGACAATGTCTGGTGGGTAGTTTGACTGGGGCGGTCGCCT 2276
UNSWCS          GCTTGAGTTATCCTCAAGTAGGACAATGTCTGGTGGGTAGTTTGACTGGGGCGGTCGCCT 2280
                ************************************************************
```

Supplementary Figure S2

ATCC33237	CCCAAAATGTAACGGAGGCTTACAAAGGTTGGCTCAGAACGGTTGGAAATCGTTCGCAGA	2338
ATCC51562	CCCAAAATGTAACGGAGGCTTACAAAGGTTGGCTCAGAACGGTTGGAAATCGTTCGTAGA	2335
H1O1	CCCAAAATGTAACGGAGGCTTACAAAGGTTGGCTCAGAACGGTTGGAAATCGTTCGTAGA	2339
H10O-S1	CCCAAAATGTAACGGAGGCTTACAAAGGTTGGCTCAGAACGGTTGGAAATCGTTCGTAGA	2335
H17O-S1	CCCAAAATGTAACGGAGGCTTACAAAGGTTGGCTCAGAACGGTTGGAAATCGTTCGTAGA	2339
H21O-S3	CCCAAAATGTAACGGAGGCTTACAAAGGTTGGCTCAGAACGGTTGGAAATCGTTCGTAGA	2339
P3UCO1	CCCAAAATGTAACGGAGGCTTACAAAGGTTGGCTCAGAACGGTTGGAAATCGTTCGTAGA	2338
P3UCB1	CCCAAAATGTAACGGAGGCTTACAAAGGTTGGCTCAGAACGGTTGGAAATCGTTCGTAGA	2338
P9CDO-S1	CCCAAAATGTAACGGAGGCTTACAAAGGTTGGCTCAGAACGGTTGGAAATCGTTCGTAGA	2335
P20CDO-S4	CCCAAAATGTAACGGAGGCTTACAAAGGTTGGCTCAGAACGGTTGGAAATCGTTCGTAGA	2338
13826	CCCAAAATGTAACGGAGGCTTACAAAGGTTGGCTCAGAACGGTTGGAAATCGTTCGTAGA	2339
ATCC51561	CCCAAAATGTAACGGAGGCTTACAAAGGTTGGCTCAGAACGGTTGGAAATCGTTCGTAGA	2336
H3O1	CCCAAAATGTAACGGAGGCTTACAAAGGTTGGCTCAGAACGGTTGGAAATCGTTCGTAGA	2336
H7O-S1	CCCAAAATGTAACGGAGGCTTACAAAGGTTGGCTCAGAACGGTTGGAAATCGTTCGTAGA	2336
H9O-S1	CCCAAAATGTAACGGAGGCTTACAAAGGTTGGCTCAGAACGGTTGGAAATCGTTCGTAGA	2335
H9O-S2	CCCAAAATGTAACGGAGGCTTACAAAGGTTGGCTCAGAACGGTTGGAAATCGTTCGTAGA	2339
H11O-S1	CCCAAAATGTAACGGAGGCTTACAAAGGTTGGCTCAGAACGGTTGGAAATCGTTCGTAGA	2336
H14O-S1	CCCAAAATGTAACGGAGGCTTACAAAGGTTGGCTCAGAACGGTTGGAAATCGTTCGTAGA	2338
H16O-S1	CCCAAAATGTAACGGAGGCTTACAAAGGTTGGCTCAGAACGGTTGGAAATCGTTCGTAGA	2336
H20O-S1	CCCAAAATGTAACGGAGGCTTACAAAGGTTGGCTCAGAACGGTTGGAAATCGTTCGTAGA	2336
H21O-S1	CCCAAAATGTAACGGAGGCTTACAAAGGTTGGCTCAGAACGGTTGGAAATCGTTCGTAGA	2339
H21O-S2	CCCAAAATGTAACGGAGGCTTACAAAGGTTGGCTCAGAACGGTTGGAAATCGTTCGTAGA	2339
H21O-S5	CCCAAAATGTAACGGAGGCTTACAAAGGTTGGCTCAGAACGGTTGGAAATCGTTCGTAGA	2339
H22O-S1	CCCAAAATGTAACGGAGGCTTACAAAGGTTGGCTCAGAACGGTTGGAAATCGTTCGTAGA	2338
H23O-S1	CCCAAAATGTAACGGAGGCTTACAAAGGTTGGCTCAGAACGGTTGGAAATCGTTCGTAGA	2338
P2CDO3	CCCAAAATGTAACGGAGGCTTACAAAGGTTGGCTCAGAACGGTTGGAAATCGTTCGTAGA	2339
P2CDO4	CCCAAAATGTAACGGAGGCTTACAAAGGTTGGCTCAGAACGGTTGGAAATCGTTCGTAGA	2339
P2CDO-S6	CCCAAAATGTAACGGAGGCTTACAAAGGTTGGCTCAGAACGGTTGGAAATCGTTCGTAGA	2339
P6CDO1	CCCAAAATGTAACGGAGGCTTACAAAGGTTGGCTCAGAACGGTTGGAAATCGTTCGTAGA	2336
P12CDO-S1	CCCAAAATGTAACGGAGGCTTACAAAGGTTGGCTCAGAACGGTTGGAAATCGTTCGTAGA	2336
P13UCO-S3	CCCAAAATGTAACGGAGGCTTACAAAGGTTGGCTCAGAACGGTTGGAAATCGTTCGTAGA	2338
P15UCO-S2	CCCAAAATGTAACGGAGGCTTACAAAGGTTGGCTCAGAACGGTTGGAAATCGTTCGTAGA	2339
P16UCO-S1	CCCAAAATGTAACGGAGGCTTACAAAGGTTGGCTCAGAACGGTTGGAAATCGTTCGTAGA	2336
P16UCO-S2	CCCAAAATGTAACGGAGGCTTACAAAGGTTGGCTCAGAACGGTTGGAAATCGTTCGTAGA	2335
P18CDO-S1	CCCAAAATGTAACGGAGGCTTACAAAGGTTGGCTCAGAACGGTTGGAAATCGTTCGTAGA	2336
P20CDO-S1	CCCAAAATGTAACGGAGGCTTACAAAGGTTGGCTCAGAACGGTTGGAAATCGTTCGTAGA	2338
P20CDO-S2	CCCAAAATGTAACGGAGGCTTACAAAGGTTGGCTCAGAACGGTTGGAAATCGTTCGTAGA	2339
P20CDO-S3	CCCAAAATGTAACGGAGGCTTACAAAGGTTGGCTCAGAACGGTTGGAAATCGTTCGTAGA	2338
P21CDO-S1	CCCAAAATGTAACGGAGGCTTACAAAGGTTGGCTCAGAACGGTTGGAAATCGTTCGTAGA	2338
P21CDO-S2	CCCAAAATGTAACGGAGGCTTACAAAGGTTGGCTCAGAACGGTTGGAAATCGTTCGTAGA	2339
P21CDO-S4	CCCAAAATGTAACGGAGGCTTACAAAGGTTGGCTCAGAACGGTTGGAAATCGTTCGTAGA	2339
P24CDO-S2	CCCAAAATGTAACGGAGGCTTACAAAGGTTGGCTCAGAACGGTTGGAAATCGTTCGTAGA	2339
P24CDO-S3	CCCAAAATGTAACGGAGGCTTACAAAGGTTGGCTCAGAACGGTTGGAAATCGTTCGTAGA	2339
P24CDO-S4	CCCAAAATGTAACGGAGGCTTACAAAGGTTGGCTCAGAACGGTTGGAAATCGTTCGTAGA	2339
UNSW1	CCCAAAATGTAACGGAGGCTTACAAAGGTTGGCTCAGAACGGTTGGAAATCGTTCGTAGA	2339
UNSW2	CCCAAAATGTAACGGAGGCTTACAAAGGTTGGCTCAGAACGGTTGGAAATCGTTCGTAGA	2339
UNSW3	CCCAAAATGTAACGGAGGCTTACAAAGGTTGGCTCAGAACGGTTGGAAATCGTTCGTAGA	2339
UNSWCD	CCCAAAATGTAACGGAGGCTTACAAAGGTTGGCTCAGAACGGTTGGAAATCGTTCGTAGA	2336
UNSWCS	CCCAAAATGTAACGGAGGCTTACAAAGGTTGGCTCAGAACGGTTGGAAATCGTTCGTAGA	2340
	** ***	

Supplementary Figure S2

```
ATCC33237       GTATAAAGGCAAAAGCCAGCTTAACTGCGAGACATACACGTCAAGCAGGGACGAAAGTCG 2398
ATCC51562       GTATAAAGGCATAAGCCAGCTTAACTGCGAGACATACACGTCAAGCAGAGACGAAAGTCG 2395
H1O1            GTATAAAGGCATAAGCCAGCTTAACTGCGAGACATACACGTCAAGCAGAGACGAAAGTCG 2399
H10O-S1         GTATAAAGGCATAAGCCAGCTTAACTGCGAGACATACACGTCAAGCAGAGACGAAAGTCG 2395
H17O-S1         GTATAAAGGCATAAGCCAGCTTAACTGCAAGACATACACGTCAAGCAGAGACGAAAGTCG 2399
H21O-S3         GTATAAAGGCATAAGCCAGCTTAACTGCAAGACATACACGTCAAGCAGAGACGAAAGTCG 2399
P3UCO1          GTATAAAGGCATAAGCCAGCTTAACTGCGAGACATACACGTCAAGCAGAGACGAAAGTCG 2398
P3UCB1          GTATAAAGGCATAAGCCAGCTTAACTGCGAGACATACACGTCAAGCAGAGACGAAAGTCG 2398
P9CDO-S1        GTATAAAGGCAAAAGCCAGCTTAACTGCGAGACATACACGTCAAGCAGAGACGAAAGTCG 2395
P20CDO-S4       GTATAAAGGCATAAGCCAGCTTAACTGCGAGACATACACGTCAAGCAGAGACGAAAGTCG 2398
13826           GTATAAAGGCATAAGCCAGCTTAACTGCGAGACATACACGTCAAGCAGAGACGAAAGTCG 2399
ATCC51561       GTATAAAGGCATAAGCCAGCTTAACTGCGAGACATACACGTCAAGCAGAGACGAAAGTCG 2396
H3O1            GTATAAAGGCATAAGCCAGCTTAACTGCGAGACATACACGTCAAGCAGAGACGAAAGTCG 2396
H7O-S1          GTATAAAGGCATAAGCCAGCTTAACTGCGAGACATACACGTCAAGCAGAGACGAAAGTCG 2396
H9O-S1          GTATAAAGGCATAAGCCAGCTTAACTGCGAGACATACACGTCAAGCAGAGACGAAAGTCG 2395
H9O-S2          GTATAAAGGCATAAGCCAGCTTAACTGCGAGACATACACGTCAAGCAGAGACGAAAGTCG 2399
H11O-S1         GTATAAAGGCATAAGCCAGCTTAACTGCGAGACATACACGTCAAGCAGAGACGAAAGTCG 2396
H14O-S1         GTATAAAGGCATAAGCCAGCTTAACTGCGAGACATACACGTCAAGCAGAGACGAAAGTCG 2398
H16O-S1         GTATAAAGGCATAAGCCAGCTTAACTGCGAGACATACACGTCAAGCAGAGACGAAAGTCG 2396
H20O-S1         GTATAAAGGCATAAGCCAGCTTAACTGCGAGACATACACGTCAAGCAGAGACGAAAGTCG 2396
H21O-S1         GTATAAAGGCATAAGCCAGCTTAACTGCGAGACATACACGTCAAGCAGAGACGAAAGTCG 2399
H21O-S2         GTATAAAGGCATAAGCCAGCTTAACTGCGAGACATACACGTCAAGCAGAGACGAAAGTCG 2399
H21O-S5         GTATAAAGGCATAAGCCAGCTTAACTGCGAGACATACACGTCAAGCAGAGACGAAAGTCG 2399
H22O-S1         GTATAAAGGCATAAGCCAGCTTAACTGCGAGACATACACGTCAAGCAGAGACGAAAGTCG 2398
H23O-S1         GTATAAAGGCATAAGCCAGCTTAACTGCGAGACATACACGTCAAGCAGAGACGAAAGTCG 2398
P2CDO3          GTATAAAGGCATAAGCCAGCTTAACTGCGAGACATACACGTCAAGCAGAGACGAAAGTCG 2399
P2CDO4          GTATAAAGGCATAAGCCAGCTTAACTGCGAGACATACACGTCAAGCAGAGACGAAAGTCG 2399
P2CDO-S6        GTATAAAGGCATAAGCCAGCTTAACTGCGAGACATACACGTCAAGCAGAGACGAAAGTCG 2399
P6CDO1          GTATAAAGGCATAAGCCAGCTTAACTGCGAGACATACACGTCAAGCAGAGACGAAAGTCG 2396
P12CDO-S1       GTATAAAGGCATAAGCCAGCTTAACTGCGAGACATACACGTCAAGCAGAGACGAAAGTCG 2396
P13UCO-S3       GTATAAAGGCATAAGCCAGCTTAACTGCGAGACATACACGTCAAGCAGAGACGAAAGTCG 2398
P15UCO-S2       GTATAAAGGCATAAGCCAGCTTAACTGCGAGACATACACGTCAAGCAGAGACGAAAGTCG 2399
P16UCO-S1       GTATAAAGGCATAAGCCAGCTTAACTGCGAGACATACACGTCAAGCAGAGACGAAAGTCG 2396
P16UCO-S2       GTATAAAGGCATAAGCCAGCTTAACTGCGAGACATACACGTCAAGCAGAGACGAAAGTCG 2395
P18CDO-S1       GTATAAAGGCATAAGCCAGCTTAACTGCGAGACATACACGTCAAGCAGAGACGAAAGTCG 2396
P20CDO-S1       GTATAAAGGCATAAGCCAGCTTAACTGCGAGACATACACGTCAAGCAGAGACGAAAGTCG 2398
P20CDO-S2       GTATAAAGGCATAAGCCAGCTTAACTGCGAGACATACACGTCAAGCAGAGACGAAAGTCG 2399
P20CDO-S3       GTATAAAGGCATAAGCCAGCTTAACTGCGAGACATACACGTCAAGCAGAGACGAAAGTCG 2398
P21CDO-S1       GTATAAAGGCATAAGCCAGCTTAACTGCGAGACATACACGTCAAGCAGAGACGAAAGTCG 2398
P21CDO-S2       GTATAAAGGCATAAGCCAGCTTAACTGCGAGACATACACGTCAAGCAGAGACGAAAGTCG 2399
P21CDO-S4       GTATAAAGGCATAAGCCAGCTTAACTGCGAGACATACACGTCAAGCAGAGACGAAAGTCG 2399
P24CDO-S2       GTATAAAGGCATAAGCCAGCTTAACTGCGAGACATACACGTCAAGCAGAGACGAAAGTCG 2399
P24CDO-S3       GTATAAAGGCATAAGCCAGCTTAACTGCGAGACATACACGTCAAGCAGAGACGAAAGTCG 2399
P24CDO-S4       GTATAAAGGCATAAGCCAGCTTAACTGCGAGACATACACGTCAAGCAGAGACGAAAGTCG 2399
UNSW1           GTATAAAGGCATAAGCCAGCTTAACTGCGAGACATACACGTCAAGCAGAGACGAAAGTCG 2399
UNSW2           GTATAAAGGCATAAGCCAGCTTAACTGCGAGACATACACGTCAAGCAGAGACGAAAGTCG 2399
UNSW3           GTATAAAGGCATAAGCCAGCTTAACTGCGAGACATACACGTCAAGCAGAGACGAAAGTCG 2399
UNSWCD          GTATAAAGGCATAAGCCAGCTTAACTGCGAGACATACACGTCAAGCAGAGACGAAAGTCG 2396
UNSWCS          GTATAAAGGCATAAGCCAGCTTAACTGCGAGACATACACGTCAAGCAGAGACGAAAGTCG 2400
                *********** **************** ******************* **********
```

Supplementary Figure S2

ATCC33237	GTCTTAGTGATCCGGTGGTTCTGTGTGGAAGGGCCATCGCTCAAAGGATAAAAGGTACCC	2458
ATCC51562	GTCTTAGTGATCCGGTGGTTCTGTGTGGAAGGGCCATCGCTCAAAGGATAAAAGGTACCC	2455
H1O1	GTCTTAGTGATCCGGTGGTTCTGTGTGGAAGGGCCATCGCTCAAAGGATAAAAGGTACCC	2459
H10O-S1	GTCTTAGTGATCCGGTGGTTCTGTGTGGAAGGGCCATCGCTCAAAGGATAAAAGGTACCC	2455
H17O-S1	GTCTTAGTGATCCGGTGGTTCTGTGTGGAAGGGCCATCGCTCAAAGGATAAAAGGTACCC	2459
H21O-S3	GTCTTAGTGATCCGGTGGTTCTGTGTGGAAGGGCCATCGCTCAAAGGATAAAAGGTACCC	2459
P3UCO1	GTCTTAGTGATCCGGTGGTTCTGTGTGGAAGGGCCATCGCTCAAAGGATAAAAGGTACCC	2458
P3UCB1	GTCTTAGTGATCCGGTGGTTCTGTGTGGAAGGGCCATCGCTCAAAGGATAAAAGGTACCC	2458
P9CDO-S1	GTCTTAGTGATCCGGTGGTTCTGTGTGGAAGGGCCATCGCTCAAAGGATAAAAGGTACCC	2455
P20CDO-S4	GTCTTAGTGATCCGGTGGTTCTGTGTGGAAGGGCCATCGCTCAAAGGATAAAAGGTACCC	2458
13826	GTCTTAGTGATCCGGTGGTTCTGTGTGGAAGGGCCATCGCTCAAAGGATAAAAGGTACCC	2459
ATCC51561	GTCTTAGTGATCCGGTGGTTCTGTGTGGAAGGGCCATCGCTCAAAGGATAAAAGGTACCC	2456
H3O1	GTCTTAGTGATCCGGTGGTTCTGTGTGGAAGGGCCATCGCTCAAAGGATAAAAGGTACCC	2456
H7O-S1	GTCTTAGTGATCCGGTGGTTCTGTGTGGAAGGGCCATCGCTCAAAGGATAAAAGGTACCC	2456
H9O-S1	GTCTTAGTGATCCGGTGGTTCTGTGTGGAAGGGCCATCGCTCAAAGGATAAAAGGTACCC	2455
H9O-S2	GTCTTAGTGATCCGGTGGTTCTGTGTGGAAGGGCCATCGCTCAAAGGATAAAAGGTACCC	2459
H11O-S1	GTCTTAGTGATCCGGTGGTTCTGTGTGGAAGGGCCATCGCTCAAAGGATAAAAGGTACCC	2456
H14O-S1	GTCTTAGTGATCCGGTGGTTCTGTGTGGAAGGGCCATCGCTCAAAGGATAAAAGGTACCC	2458
H16O-S1	GTCTTAGTGATCCGGTGGTTCTGTGTGGAAGGGCCATCGCTCAAAGGATAAAAGGTACCC	2456
H20O-S1	GTCTTAGTGATCCGGTGGTTCTGTGTGGAAGGGCCATCGCTCAAAGGATAAAAGGTACCC	2456
H21O-S1	GTCTTAGTGATCCGGTGGTTCTGTGTGGAAGGGCCATCGCTCAAAGGATAAAAGGTACCC	2459
H21O-S2	GTCTTAGTGATCCGGTGGTTCTGTGTGGAAGGGCCATCGCTCAAAGGATAAAAGGTACCC	2459
H21O-S5	GTCTTAGTGATCCGGTGGTTCTGTGTGGAAGGGCCATCGCTCAAAGGATAAAAGGTACCC	2459
H22O-S1	GTCTTAGTGATCCGGTGGTTCTGTGTGGAAGGGCCATCGCTCAAAGGATAAAAGGTACCC	2458
H23O-S1	GTCTTAGTGATCCGGTGGTTCTGTGTGGAAGGGCCATCGCTCAAAGGATAAAAGGTACCC	2458
P2CDO3	GTCTTAGTGATCCGGTGGTTCTGTGTGGAAGGGCCATCGCTCAAAGGATAAAAGGTACCC	2459
P2CDO4	GTCTTAGTGATCCGGTGGTTCTGTGTGGAAGGGCCATCGCTCAAAGGATAAAAGGTACCC	2459
P2CDO-S6	GTCTTAGTGATCCGGTGGTTCTGTGTGGAAGGGCCATCGCTCAAAGGATAAAAGGTACCC	2459
P6CDO1	GTCTTAGTGATCCGGTGGTTCTGTGTGGAAGGGCCATCGCTCAAAGGATAAAAGGTACCC	2456
P12CDO-S1	GTCTTAGTGATCCGGTGGTTCTGTGTGGAAGGGCCATCGCTCAAAGGATAAAAGGTACCC	2456
P13UCO-S3	GTCTTAGTGATCCGGTGGTTCTGTGTGGAAGGGCCATCGCTCAAAGGATAAAAGGTACCC	2458
P15UCO-S2	GTCTTAGTGATCCGGTGGTTCTGTGTGGAAGGGCCATCGCTCAAAGGATAAAAGGTACCC	2459
P16UCO-S1	GTCTTAGTGATCCGGTGGTTCTGTGTGGAAGGGCCATCGCTCAAAGGATAAAAGGTACCC	2456
P16UCO-S2	GTCTTAGTGATCCGGTGGTTCTGTGTGGAAGGGCCATCGCTCAAAGGATAAAAGGTACCC	2455
P18CDO-S1	GTCTTAGTGATCCGGTGGTTCTGTGTGGAAGGGCCATCGCTCAAAGGATAAAAGGTACCC	2456
P20CDO-S1	GTCTTAGTGATCCGGTGGTTCTGTGTGGAAGGGCCATCGCTCAAAGGATAAAAGGTACCC	2458
P20CDO-S2	GTCTTAGTGATCCGGTGGTTCTGTGTGGAAGGGCCATCGCTCAAAGGATAAAAGGTACCC	2459
P20CDO-S3	GTCTTAGTGATCCGGTGGTTCTGTGTGGAAGGGCCATCGCTCAAAGGATAAAAGGTACCC	2458
P21CDO-S1	GTCTTAGTGATCCGGTGGTTCTGTGTGGAAGGGCCATCGCTCAAAGGATAAAAGGTACCC	2458
P21CDO-S2	GTCTTAGTGATCCGGTGGTTCTGTGTGGAAGGGCCATCGCTCAAAGGATAAAAGGTACCC	2459
P21CDO-S4	GTCTTAGTGATCCGGTGGTTCTGTGTGGAAGGGCCATCGCTCAAAGGATAAAAGGTACCC	2459
P24CDO-S2	GTCTTAGTGATCCGGTGGTTCTGTGTGGAAGGGCCATCGCTCAAAGGATAAAAGGTACCC	2459
P24CDO-S3	GTCTTAGTGATCCGGTGGTTCTGTGTGGAAGGGCCATCGCTCAAAGGATAAAAGGTACCC	2459
P24CDO-S4	GTCTTAGTGATCCGGTGGTTCTGTGTGGAAGGGCCATCGCTCAAAGGATAAAAGGTACCC	2459
UNSW1	GTCTTAGTGATCCGGTGGTTCTGTGTGGAAGGGCCATCGCTCAAAGGATAAAAGGTACCC	2459
UNSW2	GTCTTAGTGATCCGGTGGTTCTGTGTGGAAGGGCCATCGCTCAAAGGATAAAAGGTACCC	2459
UNSW3	GTCTTAGTGATCCGGTGGTTCTGTGTGGAAGGGCCATCGCTCAAAGGATAAAAGGTACCC	2459
UNSWCD	GTCTTAGTGATCCGGTGGTTCTGTGTGGAAGGGCCATCGCTCAAAGGATAAAAGGTACCC	2456
UNSWCS	GTCTTAGTGATCCGGTGGTTCTGTGTGGAAGGGCCATCGCTCAAAGGATAAAAGGTACCC	2460
	**	

Supplementary Figure S2

```
ATCC33237       CGGGGATAACAGGCTGATCTCCCCCAAGAGCTCACATCGACGGGGAGGTTTGGCACCTCG 2518
ATCC51562       CGGGGATAACAGGCTGATCTCCCCCAAGAGCTCACATCGACGGGGAGGTTTGGCACCTCG 2515
H1O1            CGGGGATAACAGGCTGATCTCCCCCAAGAGCTCACATCGACGGGGAGGTTTGGCACCTCG 2519
H10O-S1         CGGGGATAACAGGCTGATCTCCCCCAAGAGCTCACATCGACGGGGAGGTTTGGCACCTCG 2515
H17O-S1         CGGGGATAACAGGCTGATCTCCCCCAAGAGCTCACATCGACGGGGAGGTTTGGCACCTCG 2519
H21O-S3         CGGGGATAACAGGCTGATCTCCCCCAAGAGCTCACATCGACGGGGAGGTTTGGCACCTCG 2519
P3UCO1          CGGGGATAACAGGCTGATCTCCCCCAAGAGCTCACATCGACGGGGAGGTTTGGCACCTCG 2518
P3UCB1          CGGGGATAACAGGCTGATCTCCCCCAAGAGCTCACATCGACGGGGAGGTTTGGCACCTCG 2518
P9CDO-S1        CGGGGATAACAGGCTGATCTCCCCCAAGAGCTCACATCGACGGGGAGGTTTGGCACCTCG 2515
P20CDO-S4       CGGGGATAACAGGCTGATCTCCCCCAAGAGCTCACATCGACGGGGAGGTTTGGCACCTCG 2518
13826           CGGGGATAACAGGCTGATCTCCCCCAAGAGCTCACATCGACGGGGAGGTTTGGCACCTCG 2519
ATCC51561       CGGGGATAACAGGCTGATCTCCCCCAAGAGCTCACATCGACGGGGAGGTTTGGCACCTCG 2516
H3O1            CGGGGATAACAGGCTGATCTCCCCCAAGAGCTCACATCGACGGGGAGGTTTGGCACCTCG 2516
H7O-S1          CGGGGATAACAGGCTGATCTCCCCCAAGAGCTCACATCGACGGGGAGGTTTGGCACCTCG 2516
H9O-S1          CGGGGATAACAGGCTGATCTCCCCCAAGAGCTCACATCGACGGGGAGGTTTGGCACCTCG 2515
H9O-S2          CGGGGATAACAGGCTGATCTCCCCCAAGAGCTCACATCGACGGGGAGGTTTGGCACCTCG 2519
H11O-S1         CGGGGATAACAGGCTGATCTCCCCCAAGAGCTCACATCGACGGGGAGGTTTGGCACCTCG 2516
H14O-S1         CGGGGATAACAGGCTGATCTCCCCCAAGAGCTCACATCGACGGGGAGGTTTGGCACCTCG 2518
H16O-S1         CGGGGATAACAGGCTGATCTCCCCCAAGAGCTCACATCGACGGGGAGGTTTGGCACCTCG 2516
H20O-S1         CGGGGATAACAGGCTGATCTCCCCCAAGAGCTCACATCGACGGGGAGGTTTGGCACCTCG 2516
H21O-S1         CGGGGATAACAGGCTGATCTCCCCCAAGAGCTCACATCGACGGGGAGGTTTGGCACCTCG 2519
H21O-S2         CGGGGATAACAGGCTGATCTCCCCCAAGAGCTCACATCGACGGGGAGGTTTGGCACCTCG 2519
H21O-S5         CGGGGATAACAGGCTGATCTCCCCCAAGAGCTCACATCGACGGGGAGGTTTGGCACCTCG 2519
H22O-S1         CGGGGATAACAGGCTGATCTCCCCCAAGAGCTCACATCGACGGGGAGGTTTGGCACCTCG 2518
H23O-S1         CGGGGATAACAGGCTGATCTCCCCCAAGAGCTCACATCGACGGGGAGGTTTGGCACCTCG 2518
P2CDO3          CGGGGATAACAGGCTGATCTCCCCCAAGAGCTCACATCGACGGGGAGGTTTGGCACCTCG 2519
P2CDO4          CGGGGATAACAGGCTGATCTCCCCCAAGAGCTCACATCGACGGGGAGGTTTGGCACCTCG 2519
P2CDO-S6        CGGGGATAACAGGCTGATCTCCCCCAAGAGCTCACATCGACGGGGAGGTTTGGCACCTCG 2519
P6CDO1          CGGGGATAACAGGCTGATCTCCCCCAAGAGCTCACATCGACGGGGAGGTTTGGCACCTCG 2516
P12CDO-S1       CGGGGATAACAGGCTGATCTCCCCCAAGAGCTCACATCGACGGGGAGGTTTGGCACCTCG 2516
P13UCO-S3       CGGGGATAACAGGCTGATCTCCCCCAAGAGCTCACATCGACGGGGAGGTTTGGCACCTCG 2518
P15UCO-S2       CGGGGATAACAGGCTGATCTCCCCCAAGAGCTCACATCGACGGGGAGGTTTGGCACCTCG 2519
P16UCO-S1       CGGGGATAACAGGCTGATCTCCCCCAAGAGCTCACATCGACGGGGAGGTTTGGCACCTCG 2516
P16UCO-S2       CGGGGATAACAGGCTGATCTCCCCCAAGAGCTCACATCGACGGGGAGGTTTGGCACCTCG 2515
P18CDO-S1       CGGGGATAACAGGCTGATCTCCCCCAAGAGCTCACATCGACGGGGAGGTTTGGCACCTCG 2516
P20CDO-S1       CGGGGATAACAGGCTGATCTCCCCCAAGAGCTCACATCGACGGGGAGGTTTGGCACCTCG 2518
P20CDO-S2       CGGGGATAACAGGCTGATCTCCCCCAAGAGCTCACATCGACGGGGAGGTTTGGCACCTCG 2519
P20CDO-S3       CGGGGATAACAGGCTGATCTCCCCCAAGAGCTCACATCGACGGGGAGGTTTGGCACCTCG 2518
P21CDO-S1       CGGGGATAACAGGCTGATCTCCCCCAAGAGCTCACATCGACGGGGAGGTTTGGCACCTCG 2518
P21CDO-S2       CGGGGATAACAGGCTGATCTCCCCCAAGAGCTCACATCGACGGGGAGGTTTGGCACCTCG 2519
P21CDO-S4       CGGGGATAACAGGCTGATCTCCCCCAAGAGCTCACATCGACGGGGAGGTTTGGCACCTCG 2519
P24CDO-S2       CGGGGATAACAGGCTGATCTCCCCCAAGAGCTCACATCGACGGGGAGGTTTGGCACCTCG 2519
P24CDO-S3       CGGGGATAACAGGCTGATCTCCCCCAAGAGCTCACATCGACGGGGAGGTTTGGCACCTCG 2519
P24CDO-S4       CGGGGATAACAGGCTGATCTCCCCCAAGAGCTCACATCGACGGGGAGGTTTGGCACCTCG 2519
UNSW1           CGGGGATAACAGGCTGATCTCCCCCAAGAGCTCACATCGACGGGGAGGTTTGGCACCTCG 2519
UNSW2           CGGGGATAACAGGCTGATCTCCCCCAAGAGCTCACATCGACGGGGAGGTTTGGCACCTCG 2519
UNSW3           CGGGGATAACAGGCTGATCTCCCCCAAGAGCTCACATCGACGGGGAGGTTTGGCACCTCG 2519
UNSWCD          CGGGGATAACAGGCTGATCTCCCCCAAGAGCTCACATCGACGGGGAGGTTTGGCACCTCG 2516
UNSWCS          CGGGGATAACAGGCTGATCTCCCCCAAGAGCTCACATCGACGGGGAGGTTTGGCACCTCG 2520
                ************************************************************
```

Supplementary Figure S2

```
ATCC33237       ATGTCGGCTCATCGCATCCTGGGGCTGGAGCAGGTCCCAAGGGTATGGCTGTTCGCCATT 2578
ATCC51562       ATGTCGGCTCATCGCATCCTGGGGCTGGAGCAGGTCCCAAGGGTATGGCTGTTCGCCATT 2575
H1O1            ATGTCGGCTCATCGCATCCTGGGGCTGGAGCAGGTCCCAAGGGTATGGCTGTTCGCCATT 2579
H10O-S1         ATGTCGGCTCATCGCATCCTGGGGCTGGAGCAGGTCCCAAGGGTATGGCTGTTCGCCATT 2575
H17O-S1         ATGTCGGCTCATCGCATCCTGGGGCTGGAGCAGGTCCCAAGGGTATGGCTGTTCGCCATT 2579
H21O-S3         ATGTCGGCTCATCGCATCCTGGGGCTGGAGCAGGTCCCAAGGGTATGGCTGTTCGCCATT 2579
P3UCO1          ATGTCGGCTCATCGCATCCTGGGGCTGGAGCAGGTCCCAAGGGTATGGCTGTTCGCCATT 2578
P3UCB1          ATGTCGGCTCATCGCATCCTGGGGCTGGAGCAGGTCCCAAGGGTATGGCTGTTCGCCATT 2578
P9CDO-S1        ATGTCGGCTCATCGCATCCTGGGGCTGGAGCAGGTCCCAAGGGTATGGCTGTTCGCCATT 2575
P20CDO-S4       ATGTCGGCTCATCGCATCCTGGGGCTGGAGCAGGTCCCAAGGGTATGGCTGTTCGCCATT 2578
13826           ATGTCGGCTCATCGCATCCTGGGGCTGGAGCAGGTCCCAAGGGTATGGCTGTTCGCCATT 2579
ATCC51561       ATGTCGGCTCATCGCATCCTGGGGCTGGAGCAGGTCCCAAGGGTATGGCTGTTCGCCATT 2576
H3O1            ATGTCGGCTCATCGCATCCTGGGGCTGGAGCAGGTCCCAAGGGTATGGCTGTTCGCCATT 2576
H7O-S1          ATGTCGGCTCATCGCATCCTGGGGCTGGAGCAGGTCCCAAGGGTATGGCTGTTCGCCATT 2576
H9O-S1          ATGTCGGCTCATCGCATCCTGGGGCTGGAGCAGGTCCCAAGGGTATGGCTGTTCGCCATT 2575
H9O-S2          ATGTCGGCTCATCGCATCCTGGGGCTGGAGCAGGTCCCAAGGGTATGGCTGTTCGCCATT 2579
H11O-S1         ATGTCGGCTCATCGCATCCTGGGGCTGGAGCAGGTCCCAAGGGTATGGCTGTTCGCCATT 2576
H14O-S1         ATGTCGGCTCATCGCATCCTGGGGCTGGAGCAGGTCCCAAGGGTATGGCTGTTCGCCATT 2578
H16O-S1         ATGTCGGCTCATCGCATCCTGGGGCTGGAGCAGGTCCCAAGGGTATGGCTGTTCGCCATT 2576
H20O-S1         ATGTCGGCTCATCGCATCCTGGGGCTGGAGCAGGTCCCAAGGGTATGGCTGTTCGCCATT 2576
H21O-S1         ATGTCGGCTCATCGCATCCTGGGGCTGGAGCAGGTCCCAAGGGTATGGCTGTTCGCCATT 2579
H21O-S2         ATGTCGGCTCATCGCATCCTGGGGCTGGAGCAGGTCCCAAGGGTATGGCTGTTCGCCATT 2579
H21O-S5         ATGTCGGCTCATCGCATCCTGGGGCTGGAGCAGGTCCCAAGGGTATGGCTGTTCGCCATT 2579
H22O-S1         ATGTCGGCTCATCGCATCCTGGGGCTGGAGCAGGTCCCAAGGGTATGGCTGTTCGCCATT 2578
H23O-S1         ATGTCGGCTCATCGCATCCTGGGGCTGGAGCAGGTCCCAAGGGTATGGCTGTTCGCCATT 2578
P2CDO3          ATGTCGGCTCATCGCATCCTGGGGCTGGAGCAGGTCCCAAGGGTATGGCTGTTCGCCATT 2579
P2CDO4          ATGTCGGCTCATCGCATCCTGGGGCTGGAGCAGGTCCCAAGGGTATGGCTGTTCGCCATT 2579
P2CDO-S6        ATGTCGGCTCATCGCATCCTGGGGCTGGAGCAGGTCCCAAGGGTATGGCTGTTCGCCATT 2579
P6CDO1          ATGTCGGCTCATCGCATCCTGGGGCTGGAGCAGGTCCCAAGGGTATGGCTGTTCGCCATT 2576
P12CDO-S1       ATGTCGGCTCATCGCATCCTGGGGCTGGAGCAGGTCCCAAGGGTATGGCTGTTCGCCATT 2576
P13UCO-S3       ATGTCGGCTCATCGCATCCTGGGGCTGGAGCAGGTCCCAAGGGTATGGCTGTTCGCCATT 2578
P15UCO-S2       ATGTCGGCTCATCGCATCCTGGGGCTGGAGCAGGTCCCAAGGGTATGGCTGTTCGCCATT 2579
P16UCO-S1       ATGTCGGCTCATCGCATCCTGGGGCTGGAGCAGGTCCCAAGGGTATGGCTGTTCGCCATT 2576
P16UCO-S2       ATGTCGGCTCATCGCATCCTGGGGCTGGAGCAGGTCCCAAGGGTATGGCTGTTCGCCATT 2575
P18CDO-S1       ATGTCGGCTCATCGCATCCTGGGGCTGGAGCAGGTCCCAAGGGTATGGCTGTTCGCCATT 2576
P20CDO-S1       ATGTCGGCTCATCGCATCCTGGGGCTGGAGCAGGTCCCAAGGGTATGGCTGTTCGCCATT 2578
P20CDO-S2       ATGTCGGCTCATCGCATCCTGGGGCTGGAGCAGGTCCCAAGGGTATGGCTGTTCGCCATT 2579
P20CDO-S3       ATGTCGGCTCATCGCATCCTGGGGCTGGAGCAGGTCCCAAGGGTATGGCTGTTCGCCATT 2578
P21CDO-S1       ATGTCGGCTCATCGCATCCTGGGGCTGGAGCAGGTCCCAAGGGTATGGCTGTTCGCCATT 2578
P21CDO-S2       ATGTCGGCTCATCGCATCCTGGGGCTGGAGCAGGTCCCAAGGGTATGGCTGTTCGCCATT 2579
P21CDO-S4       ATGTCGGCTCATCGCATCCTGGGGCTGGAGCAGGTCCCAAGGGTATGGCTGTTCGCCATT 2579
P24CDO-S2       ATGTCGGCTCATCGCATCCTGGGGCTGGAGCAGGTCCCAAGGGTATGGCTGTTCGCCATT 2579
P24CDO-S3       ATGTCGGCTCATCGCATCCTGGGGCTGGAGCAGGTCCCAAGGGTATGGCTGTTCGCCATT 2579
P24CDO-S4       ATGTCGGCTCATCGCATCCTGGGGCTGGAGCAGGTCCCAAGGGTATGGCTGTTCGCCATT 2579
UNSW1           ATGTCGGCTCATCGCATCCTGGGGCTGGAGCAGGTCCCAAGGGTATGGCTGTTCGCCATT 2579
UNSW2           ATGTCGGCTCATCGCATCCTGGGGCTGGAGCAGGTCCCAAGGGTATGGCTGTTCGCCATT 2579
UNSW3           ATGTCGGCTCATCGCATCCTGGGGCTGGAGCAGGTCCCAAGGGTATGGCTGTTCGCCATT 2579
UNSWCD          ATGTCGGCTCATCGCATCCTGGGGCTGGAGCAGGTCCCAAGGGTATGGCTGTTCGCCATT 2576
UNSWCS          ATGTCGGCTCATCGCATCCTGGGGCTGGAGCAGGTCCCAAGGGTATGGCTGTTCGCCATT 2580
                ************************************************************
```

Supplementary Figure S2

```
ATCC33237       TAAAGCGGTACGCGAGCTGGGTTCAGAACGTCGTGAGACAGTTCGGTCCC--TATCTGCC 2636
ATCC51562       TAAAGCGGTACGCGAGCTGGGTTCAGAACGTCGTGAGACAGTTCGGTCCC--TATCTGCC 2633
H1O1            TAAAGCGGTACGCGAGCTGGGTTCAGAACGTCGTGAGACAGTTCGGTCCC--TATCTGCC 2637
H10O-S1         TAAAGCGGTACGCGAGCTGGGTTCAGAACGTCGTGAGACAGTTCGGTCCC--TATCTGCC 2633
H17O-S1         TAAAGCGGTACGCGAGCTGGGTTCAGAACGTCGTGAGACAGTTCGGTCCC--TATCTGCC 2637
H21O-S3         TAAAGCGGTACGCGAGCTGGGTTCAGAACGTCGTGAGACAGTTCGGTCCC--TATCTGCC 2637
P3UCO1          TAAAGCGGTACGCGAGCTGGGTTCAGAACGTCGTGAGACAGTTCGGTCCC--TATCTGCC 2636
P3UCB1          TAAAGCGGTACGCGAGCTGGGTTCAGAACGTCGTGAGACAGTTCGGTCCC--TATCTGCC 2636
P9CDO-S1        TAAAGCGGTACGCGAGCTGGGTTCAGAACGTCGTGAGACAGTTCGGTCCC--TATCTGCC 2633
P20CDO-S4       TAAAGCGGTACGCGAGCTGGGTTCAGAACGTCGTGAGACAGTTCGGTCCC--TATCTGCC 2636
13826           TAAAGCGGTACGCGAGCTGGGTTCAGAACGTCGTGAGACAGTTCGGTCCC--TATCTGCC 2637
ATCC51561       TAAAGCGGTACGCGAGCTGGGTTCAGAACGTCGTGAGACAGTTCGGTCCCATAGTCTGCC 2636
H3O1            TAAAGCGGTACGCGAGCTGGGTTCAGAACGTCGTGAGACAGTTCGGTCCC--TATCTGCC 2634
H7O-S1          TAAAGCGGTACGCGAGCTGGGTTCAGAACGTCGTGAGACAGTTCGGTCCC--TATCTGCC 2634
H9O-S1          TAAAGCGGTACGCGAGCTGGGTTCAGAACGTCGTGAGACAGTTCGGTCCC--TATCTGCC 2633
H9O-S2          TAAAGCGGTACGCGAGCTGGGTTCAGAACGTCGTGAGACAGTTCGGTCCC--TATCTGCC 2637
H11O-S1         TAAAGCGGTACGCGAGCTGGGTTCAGAACGTCGTGAGACAGTTCGGTCCC--TATCTGCC 2634
H14O-S1         TAAAGCGGTACGCGAGCTGGGTTCAGAACGTCGTGAGACAGTTCGGTCCC--TATCTGCC 2636
H16O-S1         TAAAGCGGTACGCGAGCTGGGTTCAGAACGTCGTGAGACAGTTCGGTCCC--TATCTGCC 2634
H20O-S1         TAAAGCGGTACGCGAGCTGGGTTCAGAACGTCGTGAGACAGTTCGGTCCC--TATCTGCC 2634
H21O-S1         TAAAGCGGTACGCGAGCTGGGTTCAGAACGTCGTGAGACAGTTCGGTCCC--TATCTGCC 2637
H21O-S2         TAAAGCGGTACGCGAGCTGGGTTCAGAACGTCGTGAGACAGTTCGGTCCC--TATCTGCC 2637
H21O-S5         TAAAGCGGTACGCGAGCTGGGTTCAGAACGTCGTGAGACAGTTCGGTCCC--TATCTGCC 2637
H22O-S1         TAAAGCGGTACGCGAGCTGGGTTCAGAACGTCGTGAGACAGTTCGGTCCC--TATCTGCC 2636
H23O-S1         TAAAGCGGTACGCGAGCTGGGTTCAGAACGTCGTGAGACAGTTCGGTCCC--TATCTGCC 2636
P2CDO3          TAAAGCGGTACGCGAGCTGGGTTCAGAACGTCGTGAGACAGTTCGGTCCC--TATCTGCC 2637
P2CDO4          TAAAGCGGTACGCGAGCTGGGTTCAGAACGTCGTGAGACAGTTCGGTCCC--TATCTGCC 2637
P2CDO-S6        TAAAGCGGTACGCGAGCTGGGTTCAGAACGTCGTGAGACAGTTCGGTCCC--TATCTGCC 2637
P6CDO1          TAAAGCGGTACGCGAGCTGGGTTCAGAACGTCGTGAGACAGTTCGGTCCC--TATCTGCC 2634
P12CDO-S1       TAAAGCGGTACGCGAGCTGGGTTCAGAACGTCGTGAGACAGTTCGGTCCC--TATCTGCC 2634
P13UCO-S3       TAAAGCGGTACGCGAGCTGGGTTCAGAACGTCGTGAGACAGTTCGGTCCC--TATCTGCC 2636
P15UCO-S2       TAAAGCGGTACGCGAGCTGGGTTCAGAACGTCGTGAGACAGTTCGGTCCC--TATCTGCC 2637
P16UCO-S1       TAAAGCGGTACGCGAGCTGGGTTCAGAACGTCGTGAGACAGTTCGGTCCC--TATCTGCC 2634
P16UCO-S2       TAAAGCGGTACGCGAGCTGGGTTCAGAACGTCGTGAGACAGTTCGGTCCC--TATCTGCC 2633
P18CDO-S1       TAAAGCGGTACGCGAGCTGGGTTCAGAACGTCGTGAGACAGTTCGGTCCC--TATCTGCC 2634
P20CDO-S1       TAAAGCGGTACGCGAGCTGGGTTCAGAACGTCGTGAGACAGTTCGGTCCC--TATCTGCC 2636
P20CDO-S2       TAAAGCGGTACGCGAGCTGGGTTCAGAACGTCGTGAGACAGTTCGGTCCC--TATCTGCC 2637
P20CDO-S3       TAAAGCGGTACGCGAGCTGGGTTCAGAACGTCGTGAGACAGTTCGGTCCC--TATCTGCC 2636
P21CDO-S1       TAAAGCGGTACGCGAGCTGGGTTCAGAACGTCGTGAGACAGTTCGGTCCC--TATCTGCC 2636
P21CDO-S2       TAAAGCGGTACGCGAGCTGGGTTCAGAACGTCGTGAGACAGTTCGGTCCC--TATCTGCC 2637
P21CDO-S4       TAAAGCGGTACGCGAGCTGGGTTCAGAACGTCGTGAGACAGTTCGGTCCC--TATCTGCC 2637
P24CDO-S2       TAAAGCGGTACGCGAGCTGGGTTCAGAACGTCGTGAGACAGTTCGGTCCC--TATCTGCC 2637
P24CDO-S3       TAAAGCGGTACGCGAGCTGGGTTCAGAACGTCGTGAGACAGTTCGGTCCC--TATCTGCC 2637
P24CDO-S4       TAAAGCGGTACGCGAGCTGGGTTCAGAACGTCGTGAGACAGTTCGGTCCC--TATCTGCC 2637
UNSW1           TAAAGCGGTACGCGAGCTGGGTTCAGAACGTCGTGAGACAGTTCGGTCCC--TATCTGCC 2637
UNSW2           TAAAGCGGTACGCGAGCTGGGTTCAGAACGTCGTGAGACAGTTCGGTCCC--TATCTGCC 2637
UNSW3           TAAAGCGGTACGCGAGCTGGGTTCAGAACGTCGTGAGACAGTTCGGTCCC--TATCTGCC 2637
UNSWCD          TAAAGCGGTACGCGAGCTGGGTTCAGAACGTCGTGAGACAGTTCGGTCCC--TATCTGCC 2634
UNSWCS          TAAAGCGGTACGCGAGCTGGGTTCAGAACGTCGTGAGACAGTTCGGTCCC--TATCTGCC 2638
                **************************************************    ******
```

Supplementary Figure S2

```
ATCC33237       GTGGGCGTAAGAAGATTGAGGAGAGTTGACCCTAGTACGAGAGGACCGGGTCGAACCAAC 2696
ATCC51562       GTGGGCGCAAGAAGATTGAGGAGAGTTGACCCTAGTACGAGAGGACCGGGTCGAACCAAC 2693
H1O1            GTGGGCGCAAGAAGATTGAGGAGAGTTGACCCTAGTACGAGAGGACCGGGTCGAACCAAC 2697
H10O-S1         GTGGGCGTAAGAAGATTGAGGAGAGTTGACCCTAGTACGAGAGGACCGGGTCGAACCAAC 2693
H17O-S1         GTGGGCGTAAGAAGATTGAGGAGAGTTGACCCTAGTACGAGAGGACCGGGTCGAACCAAC 2697
H21O-S3         GTGGGCGTAAGAAGATTGAGGAGAGTTGACCCTAGTACGAGAGGACCGGGTCGAACCAAC 2697
P3UCO1          GTGGGCGCAAGAAGATTGAGGAGAGTTGACCCTAGTACGAGAGGACCGGGTCGAACCAAC 2696
P3UCB1          GTGGGCGCAAGAAGATTGAGGAGAGTTGACCCTAGTACGAGAGGACCGGGTCGAACCAAC 2696
P9CDO-S1        GTGGGCGTAAGAAGATTGAGGAGAGTTGACCCTAGTACGAGAGGACCGGGTCGAACCAAC 2693
P20CDO-S4       GTGGGCGTAAGAAGATTGAGGAGAGTTGACCCTAGTACGAGAGGACCGGGTCGAACCAAC 2696
13826           GTGGGCGCAAGAAGATTGAGGAGAGTTGACCCTAGTACGAGAGGACCGGGTCGAACCAAC 2697
ATCC51561       GTGGGCGCAAGAAGATTGAGGAGAG----------------------------------- 2661
H3O1            GTGGGCGCAAGAAGATTGAGGAGAGTTGACCCTAGTACGAGAGGACCGGGTCGAACCAAC 2694
H7O-S1          GTGGGCGCAAGAAGATTGAGGAGAGTTGACCCTAGTACGAGAGGACCGGGTCGAACCAAC 2694
H9O-S1          GTGGGCGCAAGAAGATTGAGGAGAGTTGACCCTAGTACGAGAGGACCGGGTCGAACCAAC 2693
H9O-S2          GTGGGCGCAAGAAGATTGAGGAGAGTTGACCCTAGTACGAGAGGACCGGGTCGAACCAAC 2697
H11O-S1         GTGGGCGCAAGAAGATTGAGGAGAGTTGACCCTAGTACGAGAGGACCGGGTCGAACCAAC 2694
H14O-S1         GTGGGCGCAAGAAGATTGAGGAGAGTTGACCCTAGTACGAGAGGACCGGGTCGAACCAAC 2696
H16O-S1         GTGGGCGCAAGAAGATTGAGGAGAGTTGACCCTAGTACGAGAGGACCGGGTCGAACCAAC 2694
H20O-S1         GTGGGCGCAAGAAGATTGAGGAGAGTTGACCCTAGTACGAGAGGACCGGGTCGAACCAAC 2694
H21O-S1         GTGGGCGCAAGAAGATTGAGGAGAGTTGACCCTAGTACGAGAGGACCGGGTCGAACCAAC 2697
H21O-S2         GTGGGCGCAAGAAGATTGAGGAGAGTTGACCCTAGTACGAGAGGACCGGGTCGAACCAAC 2697
H21O-S5         GTGGGCGCAAGAAGATTGAGGAGAGTTGACCCTAGTACGAGAGGACCGGGTCGAACCAAC 2697
H22O-S1         GTGGGCGCAAGAAGATTGAGGAGAGTTGACCCTAGTACGAGAGGACCGGGTCGAACCAAC 2696
H23O-S1         GTGGGCGCAAGAAGATTGAGGAGAGTTGACCCTAGTACGAGAGGACCGGGTCGAACCAAC 2696
P2CDO3          GTGGGCGTAAGAAGATTGAGGAGAGTTGACCCTAGTACGAGAGGACCGGGTCGAACCAAC 2697
P2CDO4          GTGGGCGCAAGAAGATTGAGGAGAGTTGACCCTAGTACGAGAGGACCGGGTCGAACCAAC 2697
P2CDO-S6        GTGGGCGTAAGAAGATTGAGGAGAGTTGACCCTAGTACGAGAGGACCGGGTCGAACCAAC 2697
P6CDO1          GTGGGCGCAAGAAGATTGAGGAGAGTTGACCCTAGTACGAGAGGACCGGGTCGAACCAAC 2694
P12CDO-S1       GTGGGCGCAAGAAGATTGAGGAGAGTTGACCCTAGTACGAGAGGACCGGGTCGAACCAAC 2694
P13UCO-S3       GTGGGCGCAAGAAGATTGAGGAGAGTTGACCCTAGTACGAGAGGACCGGGTCGAACCAAC 2696
P15UCO-S2       GTGGGCGCAAGAAGATTGAGGAGAGTTGACCCTAGTACGAGAGGACCGGGTCGAACCAAC 2697
P16UCO-S1       GTGGGCGCAAGAAGATTGAGGAGAGTTGACCCTAGTACGAGAGGACCGGGTCGAACCAAC 2694
P16UCO-S2       GTGGGCGCAAGAAGATTGAGGAGAGTTGACCCTAGTACGAGAGGACCGGGTCGAACCAAC 2693
P18CDO-S1       GTGGGCGTAAGAAGATTGAGGAGAGTTGACCCTAGTACGAGAGGACCGGGTCGAACCAAC 2694
P20CDO-S1       GTGGGCGCAAGAAGATTGAGGAGAGTTGACCCTAGTACGAGAGGACCGGGTCGAACCAAC 2696
P20CDO-S2       GTGGGCGCAAGAAGATTGAGGAGAGTTGACCCTAGTACGAGAGGACCGGGTCGAACCAAC 2697
P20CDO-S3       GTGGGCGCAAGAAGATTGAGGAGAGTTGACCCTAGTACGAGAGGACCGGGTCGAACCAAC 2696
P21CDO-S1       GTGGGCGCAAGAAGATTGAGGAGAGTTGACCCTAGTACGAGAGGACCGGGTCGAACCAAC 2696
P21CDO-S2       GTGGGCGCAAGAAGATTGAGGAGAGTTGACCCTAGTACGAGAGGACCGGGTCGAACCAAC 2697
P21CDO-S4       GTGGGCGCAAGAAGATTGAGGAGAGTTGACCCTAGTACGAGAGGACCGGGTCGAACCAAC 2697
P24CDO-S2       GTGGGCGCAAGAAGATTGAGGAGAGTTGACCCTAGTACGAGAGGACCGGGTCGAACCAAC 2697
P24CDO-S3       GTGGGCGCAAGAAGATTGAGGAGAGTTGACCCTAGTACGAGAGGACCGGGTCGAACCAAC 2697
P24CDO-S4       GTGGGCGCAAGAAGATTGAGGAGAGTTGACCCTAGTACGAGAGGACCGGGTCGAACCAAC 2697
UNSW1           GTGGGCGCAAGAAGATTGAGGAGAGTTGACCCTAGTACGAGAGGACCGGGTCGAACCAAC 2697
UNSW2           GTGGGCGCAAGAAGATTGAGGAGAGTTGACCCTAGTACGAGAGGACCGGGTCGAACCAAC 2697
UNSW3           GTGGGCGCAAGAAGATTGAGGAGAGTTGACCCTAGTACGAGAGGACCGGGTCGAACCAAC 2697
UNSWCD          GTGGGCGCAAGAAGATTGAGGAGAGTTGACCCTAGTACGAGAGGACCGGGTCGAACCAAC 2694
UNSWCS          GTGGGCGCAAGAAGATTGAGGAGAGTTGACCCTAGTACGAGAGGACCGGGTCGAACCAAC 2698
                ******* *****************
```

Supplementary Figure S2

```
ATCC33237       CACTGGTGTACGAGTTGTTCTGCCAAGAGCACCGCTCGGTAGCTATGTTGGGATGTGATA 2756
ATCC51562       CACTGGTGTACGAGTTGTTCTGCCAAGAGC------------------------------ 2723
H1O1            CACTGGTGTACGAGTTGTTCTGCCAAGAGCACCGCTCGGTAGCTATGTTGGGATGTGATA 2757
H10O-S1         CACTGGTGTACGAGTTGTTCTGCCAAGAGCACCGCTCGGTAGCTATGTTGGGATGTGATA 2753
H17O-S1         CACTGGTGTACGAGTTGTTCTGCCAAGAGCACCGCTCGGTAGCTATGTTGGGATGTGATA 2757
H21O-S3         CACTGGTGTACGAGTTGTTCTGCCAAGAGCACCGCTCGGTAGCTATGTTGGGATGTGATA 2757
P3UCO1          CACTGGTGTACGAGTTGTTCTGCCAAGGGCACCGCTCGGTAGCTATGTTGGGATGTGATA 2756
P3UCB1          CACTGGTGTACGAGTTGTTCTGCCAAGGGCACCGCTCGGTAGCTATGTTGGGATGTGATA 2756
P9CDO-S1        CACTGGTGTACGAGTTGTTCTGCCAAGAGCACCGCTCGGTAGCTATGTTGGGATGTGATA 2753
P20CDO-S4       CACTGGTGTACGAGTTGTTCTGCCAAGAGCACCGCTCGGTAGCTATGTTGGGATGTGATA 2756
13826           CACTGGTGTACGAGTTGTCCTGCCAAGGGCACCGCTCGGTAGCTATGTTGGGATGTGATA 2757
ATCC51561       ------------------------------------------------------------
H3O1            CACTGGTGTACGAGTTGTCCTGCCAAGGGCACCGCTCGGTAGCTATGTTGGGATGTGATA 2754
H7O-S1          CACTGGTGTACGAGTTGTCCTGCCAAGGGCACCGCTCGGTAGCTATGTTGGGATGTGATA 2754
H9O-S1          CACTGGTGTACGAGTTGTCCTGCCAAGGGCACCGCTCGGTAGCTATGTTGGGATGTGATA 2753
H9O-S2          CACTGGTGTACGAGTTGTCCTGCCAAGGGCACCGCTCGGTAGCTATGTTGGGATGTGATA 2757
H11O-S1         CACTGGTGTACGAGTTGTCCTGCCAAGGGCACCGCTCGGTAGCTATGTTGGGATGTGATA 2754
H14O-S1         CACTGGTGTACGAGTTGTCCTGCCAAGGGCACCGCTCGGTAGCTATGTTGGGATGTGATA 2756
H16O-S1         CACTGGTGTACGAGTTGTCCTGCCAAGGGCACCGCTCGGTAGCTATGTTGGGATGTGATA 2754
H20O-S1         CACTGGTGTACGAGTTGTCCTGCCAAGGGCACCGCTCGGTAGCTATGTTGGGATGTGATA 2754
H21O-S1         CACTGGTGTACGAGTTGTCCTGCCAAGGGCACCGCTCGGTAGCTATGTTGGGATGTGATA 2757
H21O-S2         CACTGGTGTACGAGTTGTCCTGCCAAGGGCACCGCTCGGTAGCTATGTTGGGATGTGATA 2757
H21O-S5         CACTGGTGTACGAGTTGTCCTGCCAAGGGCACCGCTCGGTAGCTATGTTGGGATGTGATA 2757
H22O-S1         CACTGGTGTACGAGTTGTCCTGCCAAGGGCACCGCTCGGTAGCTATGTTGGGATGTGATA 2756
H23O-S1         CACTGGTGTACGAGTTGTCCTGCCAAGGGCACCGCTCGGTAGCTATGTTGGGATGTGATA 2756
P2CDO3          CACTGGTGTACGAGTTGTCCTGCCAAGGGCACCGCTCGGTAGCTATGTTGGGATGTGATA 2757
P2CDO4          CACTGGTGTACGAGTTGTCCTGCCAAGGGCACCGCTCGGTAGCTATGTTGGGATGTGATA 2757
P2CDO-S6        CACTGGTGTACGAGTTGTCCTGCCAAGGGCACCGCTCGGTAGCTATGTTGGGATGTGATA 2757
P6CDO1          CACTGGTGTACGAGTTGTCCTGCCAAGGGCACCGCTCGGTAGCTATGTTGGGATGTGATA 2754
P12CDO-S1       CACTGGTGTACGAGTTGTCCTGCCAAGGGCACCGCTCGGTAGCTATGTTGGGATGTGATA 2754
P13UCO-S3       CACTGGTGTACGAGTTGTCCTGCCAAGGGCACCGCTCGGTAGCTATGTTGGGATGTGATA 2756
P15UCO-S2       CACTGGTGTACGAGTTGTCCTGCCAAGGGCACCGCTCGGTAGCTATGTTGGGATGTGATA 2757
P16UCO-S1       CACTGGTGTACGAGTTGTCCTGCCAAGGGCACCGCTCGGTAGCTATGTTGGGATGTGATA 2754
P16UCO-S2       CACTGGTGTACGAGTTGTCCTGCCAAGGGCACCGCTCGGTAGCTATGTTGGGATGTGATA 2753
P18CDO-S1       CACTGGTGTACGAGTTGTCCTGCCAAGGGCACCGCTCGGTAGCTATGTTGGGATGTGATA 2754
P20CDO-S1       CACTGGTGTACGAGTTGTCCTGCCAAGGGCACCGCTCGGTAGCTATGTTGGGATGTGATA 2756
P20CDO-S2       CACTGGTGTACGAGTTGTCCTGCCAAGGGCACCGCTCGGTAGCTATGTTGGGATGTGATA 2757
P20CDO-S3       CACTGGTGTACGAGTTGTCCTGCCAAGGGCACCGCTCGGTAGCTATGTTGGGATGTGATA 2756
P21CDO-S1       CACTGGTGTACGAGTTGTCCTGCCAAGGGCACCGCTCGGTAGCTATGTTGGGATGTGATA 2756
P21CDO-S2       CACTGGTGTACGAGTTGTCCTGCCAAGGGCACCGCTCGGTAGCTATGTTGGGATGTGATA 2757
P21CDO-S4       CACTGGTGTACGAGTTGTCCTGCCAAGGGCACCGCTCGGTAGCTATGTTGGGATGTGATA 2757
P24CDO-S2       CACTGGTGTACGAGTTGTCCTGCCAAGGGCACCGCTCGGTAGCTATGTTGGGATGTGATA 2757
P24CDO-S3       CACTGGTGTACGAGTTGTCCTGCCAAGGGCACCGCTCGGTAGCTATGTTGGGATGTGATA 2757
P24CDO-S4       CACTGGTGTACGAGTTGTCCTGCCAAGGGCACCGCTCGGTAGCTATGTTGGGATGTGATA 2757
UNSW1           CACTGGTGTACGAGTTGTCCTGCCAAGGGCACCGCTCGGTAGCTATGTTGGGATGTGATA 2757
UNSW2           CACTGGTGTACGAGTTGTCCTGCCAAGGGCACCGCTCGGTAGCTATGTTGGGATGTGATA 2757
UNSW3           CACTGGTGTACGAGTTGTCCTGCCAAGGGCACCGCTCGGTAGCTATGTTGGGATGTGATA 2757
UNSWCD          CACTGG------------------------------------------------------ 2700
UNSWCS          CACTGGTGTACGAGTTGTCCTGCCAAGGGCACCGCTCGGTAGCTATGTTGGGATGTGATA 2758
```

Supplementary Figure S2

ATCC33237	ACTGCTGAAAGCATCTAAGCAGGAAGCCAACTCCAAGATGAATCTTCTTTTAAGAGCTCA	2816
ATCC51562	--	
H1O1	ACTGCTGAAAGCATCTAAGCAGGAAGCCAACTCCAAGATGAATCTTCTTTTAAGAGCTCA	2817
H10O-S1	ACTGCTGAAAGCATCTAAGCAGGAAGCCAACTCCAAGATGAATCTTCTTTTAAGAGCTCA	2813
H17O-S1	ACTGCTGAAAGCATCTAAGCAGGAAGCCAACTCCAAGATGAATCTTCTTTTAAGAGCTCA	2817
H21O-S3	ACTGCTGAAAGCATCTAAGCAGGAAGCCAACTCCAAGATGAATCTTCTTTTAAGAGCTCA	2817
P3UCO1	ACTGCTGAAAGCATCTAAGCAGGAAGCCAACTCCAAGATGAATCTTCTTTTAAGAGCTCA	2816
P3UCB1	ACTGCTGAAAGCATCTAAGCAGGAAGCCAACTCCAAGATGAATCTTCTTTTAAGAGCTCA	2816
P9CDO-S1	ACTGCTGAAAGCATCTAAGCAGGAAGCCAACTCCAAGATGAATCTTCTTTTAAGAGCTCA	2813
P20CDO-S4	ACTGCTGAAAGCATCTAAGCAGGAAGCCAACTCCAAGATGAATCTTCTTTTAAGAGCTCA	2816
13826	ACTGCTGAAAGCATCTAAGCAGGAAGCCAACTCCAAGATGAATCTTCTTTTAAGAGCTCA	2817
ATCC51561	--	
H3O1	ACTGCTGAAAGCATCTAAGCAGGAAGCCAACTCCAAGATGAATCTTCTTTTAAGAGCTCA	2814
H7O-S1	ACTGCTGAAAGCATCTAAGCAGGAAGCCAACTCCAAGATGAATCTTCTTTTAAGAGCTCA	2814
H9O-S1	ACTGCTGAAAGCATCTAAGCAGGAAGCCAACTCCAAGATGAATCTTCTTTTAAGAGCTCA	2813
H9O-S2	ACTGCTGAAAGCATCTAAGCAGGAAGCCAACTCCAAGATGAATCTTCTTTTAAGAGCTCA	2817
H11O-S1	ACTGCTGAAAGCATCTAAGCAGGAAGCCAACTCCAAGATGAATCTTCTTTTAAGAGCTCA	2814
H14O-S1	ACTGCTGAAAGCATCTAAGCAGGAAGCCAACTCCAAGATGAATCTTCTTTTAAGAGCTCA	2816
H16O-S1	ACTGCTGAAAGCATCTAAGCAGGAAGCCAACTCCAAGATGAATCTTCTTTTAAGAGCTCA	2814
H20O-S1	ACTGCTGAAAGCATCTAAGCAGGAAGCCAACTCCAAGATGAATCTTCTTTTAAGAGCTCA	2814
H21O-S1	ACTGCTGAAAGCATCTAAGCAGGAAGCCAACTCCAAGATGAATCTTCTTTTAAGAGCTCA	2817
H21O-S2	ACTGCTGAAAGCATCTAAGCAGGAAGCCAACTCCAAGATGAATCTTCTTTTAAGAGCTCA	2817
H21O-S5	ACTGCTGAAAGCATCTAAGCAGGAAGCCAACTCCAAGATGAATCTTCTTTTAAGAGCTCA	2817
H22O-S1	ACTGCTGAAAGCATCTAAGCAGGAAGCCAACTCCAAGATGAATCTTCTTTTAAGAGCTCA	2816
H23O-S1	ACTGCTGAAAGCATCTAAGCAGGAAGCCAACTCCAAGATGAATCTTCTTTTAAGAGCTCA	2816
P2CDO3	ACTGCTGAAAGCATCTAAGCAGGAAGCCAACTCCAAGATGAATCTTCTTTTAAGAGCTCA	2817
P2CDO4	ACTGCTGAAAGCATCTAAGCAGGAAGCCAACTCCAAGATGAATCTTCTTTTAAGAGCTCA	2817
P2CDO-S6	ACTGCTGAAAGCATCTAAGCAGGAAGCCAACTCCAAGATGAATCTTCTTTTAAGAGCTCA	2817
P6CDO1	ACTGCTGAAAGCATCTAAGCAGGAAGCCAACTCCAAGATGAATCTTCTTTTAAGAGCTCA	2814
P12CDO-S1	ACTGCTGAAAGCATCTAAGCAGGAAGCCAACTCCAAGATGAATCTTCTTTTAAGAGCTCA	2814
P13UCO-S3	ACTGCTGAAAGCATCTAAGCAGGAAGCCAACTCCAAGATGAATCTTCTTTTAAGAGCTCA	2816
P15UCO-S2	ACTGCTGAAAGCATCTAAGCAGGAAGCCAACTCCAAGATGAATCTTCTTTTAAGAGCTCA	2817
P16UCO-S1	ACTGCTGAAAGCATCTAAGCAGGAAGCCAACTCCAAGATGAATCTTCTTTTAAGAGCTCA	2814
P16UCO-S2	ACTGCTGAAAGCATCTAAGCAGGAAGCCAACTCCAAGATGAATCTTCTTTTAAGAGCTCA	2813
P18CDO-S1	ACTGCTGAAAGCATCTAAGCAGGAAGCCAACTCCAAGATGAATCTTCTTTTAAGAGCTCA	2814
P20CDO-S1	ACTGCTGAAAGCATCTAAGCAGGAAGCCAACTCCAAGATGAATCTTCTTTTAAGAGCTCA	2816
P20CDO-S2	ACTGCTGAAAGCATCTAAGCAGGAAGCCAACTCCAAGATGAATCTTCTTTTAAGAGCTCA	2817
P20CDO-S3	ACTGCTGAAAGCATCTAAGCAGGAAGCCAACTCCAAGATGAATCTTCTTTTAAGAGCTCA	2816
P21CDO-S1	ACTGCTGAAAGCATCTAAGCAGGAAGCCAACTCCAAGATGAATCTTCTTTTAAGAGCTCA	2816
P21CDO-S2	ACTGCTGAAAGCATCTAAGCAGGAAGCCAACTCCAAGATGAATCTTCTTTTAAGAGCTCA	2817
P21CDO-S4	ACTGCTGAAAGCATCTAAGCAGGAAGCCAACTCCAAGATGAATCTTCTTTTAAGAGCTCA	2817
P24CDO-S2	ACTGCTGAAAGCATCTAAGCAGGAAGCCAACTCCAAGATGAATCTTCTTTTAAGAGCTCA	2817
P24CDO-S3	ACTGCTGAAAGCATCTAAGCAGGAAGCCAACTCCAAGATGAATCTTCTTTTAAGAGCTCA	2817
P24CDO-S4	ACTGCTGAAAGCATCTAAGCAGGAAGCCAACTCCAAGATGAATCTTCTTTTAAGAGCTCA	2817
UNSW1	ACTGCTGAAAGCATCTAAGCAGGAAGCCAACTCCAAGATGAATCTTCTTTTAAGAGCTCA	2817
UNSW2	ACTGCTGAAAGCATCTAAGCAGGAAGCCAACTCCAAGATGAATCTTCTTTTAAGAGCTCA	2817
UNSW3	ACTGCTGAAAGCATCTAAGCAGGAAGCCAACTCCAAGATGAATCTTCTTTTAAGAGCTCA	2817
UNSWCD	--	
UNSWCS	ACTGCTGAAAGCATCTAAGCAGGAAGCCAACTCCAAGATGAATCTTCTTTTAAGAGCTCA	2818

Supplementary Figure S2

```
ATCC33237       TATAGACTATGTGTTTGATAGGCTGGGTGTGTAATGGATGAAAGTCCTTTAGCTGACCAG 2876
ATCC51562       ------------------------------------------------------------
H1O1            TATAGACTATGTGTTTGATAGGCTGGGTGTGTAATGGATGAAAGTCCTTTAGCTGACCAG 2877
H10O-S1         TATAGACTATGTGTTTGATAGGCTGGGTGTGTAATGGATGAAAGTCCTTTAGCTGACCAG 2873
H17O-S1         TATAGACTATGTGTTTGATAGGCTGGGTGTGTAATGGATGAAAGTCCTTTAGCTGACCAG 2877
H21O-S3         TATAGACTATGTGTTTGATAGGCTGGGTGTGTAATGGATGAAAGTCCTTTAGCTGACCAG 2877
P3UCO1          TATAGACTATGTGTTTGATAGGCTGGGTGTGTAATGGATGAAAGTCCTTTAGCTGACCAG 2876
P3UCB1          TATAGACTATGTGTTTGATAGGCTGGGTGTGTAATGGATGAAAGTCCTTTAGCTGACCAG 2876
P9CDO-S1        TATAGACTATGTGTTTGATAGGCTGGGTGTGTAATGGATGAAAGTCCTTTAGCTGACCAG 2873
P20CDO-S4       TATAGACTATGTGTTTGATAGGCTGGGTGTGTAATGGATGAAAGTCCTTTAGCTGACCAG 2876
13826           TATAGACTATGTGTTTGATAGGCTGGGTGTGTAATGGATGAAAGTCCTTTAGCTGACCAG 2877
ATCC51561       ------------------------------------------------------------
H3O1            TATAGACTATGTGTTTGATAGGCTGGGTGTGTAATGGATGAAAGTCCTTTAGCTGACCAG 2874
H7O-S1          TATAGACTATGTGTTTGATAGGCTGGGTGTGTAATGGATGAAAGTCCTTTAGCTGACCAG 2874
H9O-S1          TATAGACTATGTGTTTGATAGGCTGGGTGTGTAATGGATGAAAGTCCTTTAGCTGACCAG 2873
H9O-S2          TATAGACTATGTGTTTGATAGGCTGGGTGTGTAATGGATGAAAGTCCTTTAGCTGACCAG 2877
H11O-S1         TATAGACTATGTGTTTGATAGGCTGGGTGTGTAATGGATGAAAGTCCTTTAGCTGACCAG 2874
H14O-S1         TATAGACTATGTGTTTGATAGGCTGGGTGTGTAATGGATGAAAGTCCTTTAGCTGACCAG 2876
H16O-S1         TATAGACTATGTGTTTGATAGGCTGGGTGTGTAATGGATGAAAGTCCTTTAGCTGACCAG 2874
H20O-S1         TATAGACTATGTGTTTGATAGGCTGGGTGTGTAATGGATGAAAGTCCTTTAGCTGACCAG 2874
H21O-S1         TATAGACTATGTGTTTGATAGGCTGGGTGTGTAATGGATGAAAGTCCTTTAGCTGACCAG 2877
H21O-S2         TATAGACTATGTGTTTGATAGGCTGGGTGTGTAATGGATGAAAGTCCTTTAGCTGACCAG 2877
H21O-S5         TATAGACTATGTGTTTGATAGGCTGGGTGTGTAATGGATGAAAGTCCTTTAGCTGACCAG 2877
H22O-S1         TATAGACTATGTGTTTGATAGGCTGGGTGTGTAATGGATGAAAGTCCTTTAGCTGACCAG 2876
H23O-S1         TATAGACTATGTGTTTGATAGGCTGGGTGTGTAATGGATGAAAGTCCTTTAGCTGACCAG 2876
P2CDO3          TATAGACTATGTGTTTGATAGGCTGGGTGTGTAATGGATGAAAGTCCTTTAGCTGACCAG 2877
P2CDO4          TATAGACTATGTGTTTGATAGGCTGGGTGTGTAATGGATGAAAGTCCTTTAGCTGACCAG 2877
P2CDO-S6        TATAGACTATGTGTTTGATAGGCTGGGTGTGTAATGGATGAAAGTCCTTTAGCTGACCAG 2877
P6CDO1          TATAGACTATGTGTTTGATAGGCTGGGTGTGTAATGGATGAAAGTCCTTTAGCTGACCAG 2874
P12CDO-S1       TATAGACTATGTGTTTGATAGGCTGGGTGTGTAATGGATGAAAGTCCTTTAGCTGACCAG 2874
P13UCO-S3       TATAGACTATGTGTTTGATAGGCTGGGTGTGTAATGGATGAAAGTCCTTTAGCTGACCAG 2876
P15UCO-S2       TATAGACTATGTGTTTGATAGGCTGGGTGTGTAATGGATGAAAGTCCTTTAGCTGACCAG 2877
P16UCO-S1       TATAGACTATGTGTTTGATAGGCTGGGTGTGTAATGGATGAAAGTCCTTTAGCTGACCAG 2874
P16UCO-S2       TATAGACTATGTGTTTGATAGGCTGGGTGTGTAATGGATGAAAGTCCTTTAGCTGACCAG 2873
P18CDO-S1       TATAGACTATGTGTTTGATAGGCTGGGTGTGTAATGGATGAAAGTCCTTTAGCTGACCAG 2874
P20CDO-S1       TATAGACTATGTGTTTGATAGGCTGGGTGTGTAATGGATGAAAGTCCTTTAGCTGACCAG 2876
P20CDO-S2       TATAGACTATGTGTTTGATAGGCTGGGTGTGTAATGGATGAAAGTCCTTTAGCTGACCAG 2877
P20CDO-S3       TATAGACTATGTGTTTGATAGGCTGGGTGTGTAATGGATGAAAGTCCTTTAGCTGACCAG 2876
P21CDO-S1       TATAGACTATGTGTTTGATAGGCTGGGTGTGTAATGGATGAAAGTCCTTTAGCTGACCAG 2876
P21CDO-S2       TATAGACTATGTGTTTGATAGGCTGGGTGTGTAATGGATGAAAGTCCTTTAGCTGACCAG 2877
P21CDO-S4       TATAGACTATGTGTTTGATAGGCTGGGTGTGTAATGGATGAAAGTCCTTTAGCTGACCAG 2877
P24CDO-S2       TATAGACTATGTGTTTGATAGGCTGGGTGTGTAATGGATGAAAGTCCTTTAGCTGACCAG 2877
P24CDO-S3       TATAGACTATGTGTTTGATAGGCTGGGTGTGTAATGGATGAAAGTCCTTTAGCTGACCAG 2877
P24CDO-S4       TATAGACTATGTGTTTGATAGGCTGGGTGTGTAATGGATGAAAGTCCTTTAGCTGACCAG 2877
UNSW1           TATAGACTATGTGTTTGATAGGCTGGGTGTGTAATGGATGAAAGTCCTTTAGCTGACCAG 2877
UNSW2           TATAGACTATGTGTTTGATAGGCTGGGTGTGTAATGGATGAAAGTCCTTTAGCTGACCAG 2877
UNSW3           TATAGACTATGTGTTTGATAGGCTGGGTGTGTAATGGATGAAAGTCCTTTAGCTGACCAG 2877
UNSWCD          ------------------------------------------------------------
UNSWCS          TATAGACTATGTGTTTGATAGGCTGGGTGTGTAATGGATGAAAGTCCTTTAGCTGACCAG 2878
```

Supplementary Figure S2

ATCC33237	TACTAATAGCTCGTCTGCTTATCTTT	2902
ATCC51562	--------------------------	
H1O1	TACTAATAGCTCGTCTGCTTATCTTT	2903
H10O-S1	TACTAATAGCTCGTCTGCTTATCTTT	2899
H17O-S1	TACTAATAGCTCGTCTGCTTATCTTT	2903
H21O-S3	TACTAATAGCTCGTCTGCTTATCTTT	2903
P3UCO1	TACTAATAGCTCGTCTGCTTATCTTT	2902
P3UCB1	TACTAATAGCTCGTCTGCTTATCTTT	2902
P9CDO-S1	TACTAATAGCTCGTCTGCTTATCTTT	2899
P20CDO-S4	TACTAATAGCTCGTCTGCTTATCTT-	2901
13826	TACTAATAGCTCGTCTGCTTATCTTT	2903
ATCC51561	--------------------------	
H3O1	TACTAATAGCTCGTCTGCTTATCTTT	2900
H7O-S1	TACTAATAGCTCGTCTGCTTATCTTT	2900
H9O-S1	TACTAATAGCTCGTCTGCTTATCTTT	2899
H9O-S2	TACTAATAGCTCGTCTGCTTATCTTT	2903
H11O-S1	TACTAATAGCTCGTCTGCTTATCTTT	2900
H14O-S1	TACTAATAGCTCGTCTGCTTATCTTT	2902
H16O-S1	TACTAATAGCTCGTCTGCTTATCTTT	2900
H20O-S1	TACTAATAGCTCGTCTGCTTATCTTT	2900
H21O-S1	TACTAATAGCTCGTCTGCTTATCTTT	2903
H21O-S2	TACTAATAGCTCGTCTGCTTATCTTT	2903
H21O-S5	TACTAATAGCTCGTCTGCTTATCTTT	2903
H22O-S1	TACTAATAGCTCGTCTGCTTATCTTT	2902
H23O-S1	TACTAATAGCTCGTCTGCTTATCTTT	2902
P2CDO3	TACTAATAGCTCGTCTGCTTATCTTT	2903
P2CDO4	TACTAATAGCTCGTCTGCTTATCTTT	2903
P2CDO-S6	TACTAATAGCTCGTCTGCTTATCTTT	2903
P6CDO1	TACTAATAGCTCGTCTGCTTATCTTT	2900
P12CDO-S1	TACTAATAGCTCGTCTGCTTATCTTT	2900
P13UCO-S3	TACTAATAGCTCGTCTGCTTATCTTT	2902
P15UCO-S2	TACTAATAGCTCGTCTGCTTATCTTT	2903
P16UCO-S1	TACTAATAGCTCGTCTGCTTATCTTT	2900
P16UCO-S2	TACTAATAGCTCGTCTGCTTATCTTT	2899
P18CDO-S1	TACTAATAGCTCGTCTGCTTATCTTT	2900
P20CDO-S1	TACTAATAGCTCGTCTGCTTATCTTT	2902
P20CDO-S2	TACTAATAGCTCGTCTGCTTATCTTT	2903
P20CDO-S3	TACTAATAGCTCGTCTGCTTATCTTT	2902
P21CDO-S1	TACTAATAGCTCGTCTGCTTATCTTT	2902
P21CDO-S2	TACTAATAGCTCGTCTGCTTATCTTT	2903
P21CDO-S4	TACTAATAGCTCGTCTGCTTATCTTT	2903
P24CDO-S2	TACTAATAGCTCGTCTGCTTATCTTT	2903
P24CDO-S3	TACTAATAGCTCGTCTGCTTATCTTT	2903
P24CDO-S4	TACTAATAGCTCGTCTGCTTATCTTT	2903
UNSW1	TACTAATAGCTCGTCTGCTTATCTTT	2903
UNSW2	TACTAATAGCTCGTCTGCTTATCTTT	2903
UNSW3	TACTAATAGCTCGTCTGCTTATCTTT	2903
UNSWCD	--------------------------	
UNSWCS	TACTAATAGCTCGTCTGCTTATCTTT	2904
